# Early-life factors shaping the gut microbiota of Common buzzard nestlings

**DOI:** 10.1186/s42523-024-00313-8

**Published:** 2024-05-14

**Authors:** Hugo Pereira, Nayden Chakarov, Joseph I. Hoffman, Tony Rinaud, Meinolf Ottensmann, Kai-Philipp Gladow, Busche Tobias, Barbara A. Caspers, Öncü Maraci, Oliver Krüger

**Affiliations:** 1 Department of Animal Behaviour, Bielefeld University, Konsequenz 45, 33615 Bielefeld, NRW Germany; 2 Department of Evolutionary Population Genetics, Bielefeld University, Konsequenz 45, 33615 Bielefeld, NRW Germany; 3 Department of Behavioural Ecology, Bielefeld University, Konsequenz 45, 33615 Bielefeld, NRW Germany; 4 Medical School East Westphalia-Lippe & Center for Biotechnology (CeBiTec), Bielefeld University, Universitätsstraße 27, 33615 Bielefeld, NRW Germany; 5 Joint Institute for Individualisation in a Changing Environment (JICE), Bielefeld University and University of Münster, Konsequenz 45, 33615 Bielefeld, NRW Germany; 6 British Antarctic Survey, High Cross, Madingley Road, Cambridge, CB3 OET UK

**Keywords:** Bacterial microbiota, Eukaryotic microbiota, Early-life stages, 16S rRNA gene, 28S rRNA gene, Longitudinal study, Common buzzard

## Abstract

**Background:**

Exploring the dynamics of gut microbiome colonisation during early-life stages is important for understanding the potential impact of microbes on host development and fitness. Evidence from model organisms suggests a crucial early-life phase when shifts in gut microbiota can lead to immune dysregulation and reduced host condition. However, our understanding of gut microbiota colonisation in long-lived vertebrates, especially during early development, remains limited. We therefore used a wild population of common buzzard nestlings (*Buteo buteo*) to investigate connections between the early-life gut microbiota colonisation, environmental and host factors.

**Results:**

We targeted both bacterial and eukaryotic microbiota using the 16S and 28S rRNA genes. We sampled the individuals during early developmental stages in a longitudinal design. Our data revealed that age significantly affected microbial diversity and composition. Nest environment was a notable predictor of microbiota composition, with particularly eukaryotic communities differing between habitats occupied by the hosts. Nestling condition and infection with the blood parasite *Leucocytozoon* predicted microbial community composition.

**Conclusion:**

Our findings emphasise the importance of studying microbiome dynamics to capture changes occurring during ontogeny. They highlight the role of microbial communities in reflecting host health and the importance of the nest environment for the developing nestling microbiome. Overall, this study contributes to understanding the complex interplay between microbial communities, host factors, and environmental variables, and sheds light on the ecological processes governing gut microbial colonisation during early-life stages.

**Supplementary Information:**

The online version contains supplementary material available at 10.1186/s42523-024-00313-8.

## Introduction

The gut microbiome constitutes a diverse assemblage of microorganisms encompassing bacteria, archaea, viruses, and microbial eukaryotes, which collectively exert significant influences on various host processes [59, 94]. Numerous studies have demonstrated the importance of gut microbial communities in regulating physiological functions such as digestion, absorption, metabolism, and immune response, fostering host health across diverse animal species [105].

A fundamental aspect of studying animal microbiota communities involves understanding how they are acquired and how these communities evolve over time. A comprehensive study of humans conducted by Martino et al [74] demonstrated a sequence of transformations in gut microbial diversity and composition throughout different life stages. The process commences with initial colonisation by pioneer species at birth, followed by the emergence of body site-specific microbial communities (”primary succession”). Over time, these communities grow in complexity until they reach a stable structure (”secondary succession”). Ultimately, as the host organism senesces, the community undergoes a final transformation marked by a decline in diversity (”third succession”).

In avian species, the chick gastrointestinal tract is initially colonised by various transient species, with bacterial communities gradually transitioning into a more stable adult state [37]. Avian embryos develop within a closed and essentially sterile environment (the egg); gut microbial colonisation commences shortly after birth and is influenced by interactions among early-life environment, parental microbial transmission, and diet [26, 42, 54]. This early establishment phase, aptly captured by the “nidobiome” concept, encompasses the collective impacts of parents, the nest, and nestlings on the initial microbiome assembly [24, 26, 36]. This concept considers their interactions over time, as well as the inherent feedback mechanisms between hosts, their microbiome, and the surrounding environment [24]. Disruptions of these early-life microbiome assembly processes can adversely affect the host [55, 99] by influencing immune system development [41, 54], and diminishing resistance to parasitic infections [7, 78]. Understanding the factors influencing avian gut microbial composition in early life is therefore of great importance due to the potential long-term impacts of the gut microbiota on host development and body condition [55, 77].

The relationship between the gut microbiome and host condition is complex, exhibiting variation across different host taxa [42, 52, 55]. While many studies have shown that the gut microbiome undergoes dynamic changes throughout development, important questions persist regarding its predictive role in host health [105, 106]. Specifically, little is known about the developmental stages at which the microbiome may influence host condition [33, 114]. In general, higher gut microbial diversity has been associated with improved host health [95, 101] whereas low diversity has been linked to disease states [111]. A diverse gut microbiota exhibits a broader spectrum of metabolic capabilities, enhances immune signalling and competition against harmful pathogens [17, 29, 79]. It is also possible that high diversity might be related to a state of dysbiosis and thus a reduction in diversity might be indicative of a return to a state of homeostasis [30, 49]. On the other hand, emerging evidence suggests that the composition of the gut microbiota, rather than diversity alone, may have an even greater impact on host health [118].

In addition to host and environmental factors, parasite infection can trigger changes in the host microbiome [67, 70, 112]. For example, Madlala et al [67] showed that *Eimeria spp.* infection disrupts the gut environment increasing susceptibility to diseases that significantly threaten the health and productivity of chickens. On the other hand, *Plasmodium* infection appears not to markedly alter microbial diversity and composition in canaries (*Serinus canaria domestica*), Hawaiian honeycreepers (subfamily *Hemithraupinae*) and Eurasian tree sparrows (*Passer montanus*), although correlations with specific bacterial taxa were identified [7, 78, 91].

Despite this body of knowledge, the majority of gut microbiome research focuses exclusively on bacteria, the dominant taxonomic group in vertebrate gut ecosystems [47]. Relatively few studies have investigated the structure and diversity of other microbial kingdoms within the gut microbiome, such as viruses, archaea and microbial eukaryotes, and the underlying drivers of such variation [12, 104, 119]. Nonetheless, mounting evidence suggests that host-associated eukaryotic communities, particularly fungal communities, contribute to host health by participating in vital host processes. For example, within the gut, eukaryotic species play pivotal roles in digestion [120] and the development and modulation of the host immune system [122]. While most of the evidence supporting the importance of gut eukaryotic communities on host health comes from captive and model organisms [122], scarce research has explored patterns of eukaryotic variation in wild populations [104, 116, 119].

Here, we aim to elucidate the factors that shape the early-life gut microbiota of common buzzard (*Buteo buteo*) nestlings and the effects of the gut microbiota on body condition, focusing on both bacterial and eukaryotic components of the microbiome. On average, breeding pairs of common buzzards typically have brood sizes of approximately two nestlings [57] and there is a dominance hierarchy within the brood, with older nestlings usually being stronger at competing for food resources [39]. During their approximately five-week nestling period ($\approx$ 35 days), common buzzard nestlings rely entirely on parental care and provisioning, followed by a post-nestling phase where fledglings remain in the nest vicinity, receiving parental care and being provisioned even after they are fully fledged ($\approx$ 47 days) [110]. We collected data at two distinct time points during the nestling period of each nestling. Our dataset includes comprehensive information on environmental factors, such as nest sharing, habitat type, and sampling year, as well as key host factors including age, sex, rank (dominance hierarchy within the brood), blood parasite infection status, weight, and wing length. Additionally, we collected microbiome samples via cloacal swabs to thoroughly assess the microbial diversity and composition. We adopt a multimarker approach utilising both the conventional 16S rRNA gene and the less explored 28S rRNA gene. This strategy aims not only to identify bacteria but also to capture diverse eukaryotic communities beyond fungal populations [56], thereby contributing to a deeper understanding of the broader eukaryotic microbiome.

We hypothesise that factors such as nest sharing, age, and body condition, play pivotal roles in shaping the diversity and composition of the gut microbiome in common buzzard nestlings. Concerning microbiome diversity, two plausible scenarios emerge: 1. An increase in diversity with increasing age, suggesting that maturing individuals acquire an increasingly diverse microbiome, enabling them to take advantage of diverse functional capabilities provided by a broader array of microorganisms [17]; 2. As individuals mature, diversity declines, indicating a gradual transition to a more stable community, after a rapid and uncontrolled colonisation which occurred shortly after hatching [30]. By unravelling the complex relationships among microbial communities, host and environmental factors, we aim to shed light on the mechanisms driving gut microbial colonisation in the first stages of life.

## Methods

### Study area and sample collection

This study was conducted within a designated 300 km^2^ study area located in North Rhine-Westphalia, Germany (8^∘^ 25′ E and 52^∘^ 06′ N). For a detailed description of the study system see Krüger [57] and Jonker et al [50]. A total of 117 common buzzard nestlings were sampled, with 107 individuals being sampled in 2020 and 10 individuals being sampled in 2021. Out of these, 5 individuals were sampled once, while the majority were sampled twice, and one individual was sampled three times. Individuals were sampled from 54 nests with a brood size per nest of approximately 2.17 ± 0.853 nestlings. The sampling was distributed across three different habitats: North, South, and Teuto (Teutoburg Forest), as described in Krüger [57] and Jonker et al [50] (Appendix A Table S1).

Morphometric measurements were recorded during each sampling event, including body weight (to the nearest 5 g) and wing length (to the nearest mm). Individuals were re-sampled on average nine days after the initial sampling (mean ± s.d. = 8.7 ± 5.23 days). The average age at the first sampling point was 19 days (mean ± s.d. = 19.3 ± 5.29 days), while the average age at the second sampling point was 28 days (mean ± s.d. = 27.8 ± 5.16 days). Due to the inherent difficulty of precisely determining nestling ages without the daily disturbance of nests, it was not possible to sample individuals at the exact same ages.

Nestling age estimation (after first sampling) was done by employing a sex-specific polynomial regression model on wing length. This model was constructed based on growth curve data published by Bijlmsa [13]. Body condition index (BCI) was calculated by obtaining the residuals from a logarithmic regression of weight on wing length, while accounting for sex.

Blood samples (500–1000 $\upmu$l) were collected from the ulnar vein. A small drop of blood was used to prepare blood smears and the remaining volume was placed into 96% ethanol tubes and stored at −20 ^∘^C. Sex determination followed the standard protocol described by Fridolfsson and Ellegren [40], while infection status with the haemosporidian blood parasite *Leucocytozoon* was determined using the procedure outlined in Chakarov et al [25].

For gut microbiota analysis, adult birds were swabbed in the field by inserting the entire head of the swab into the cloaca and swabbing the inside for approximately 5 s in circular motions. The swab was then transferred to a tube containing RNAlater (Sigma- Aldrich, R0901), which was stored in dry ice and subsequently transferred to long-term storage at −80 ^∘^C. Environmental controls were obtained by swabbing various surfaces around the working station. Additionally, blank swabs were collected as negative controls to account for potential contamination during the sampling process.

### DNA extraction from cloacal swabs

DNA extraction from cloacal swabs was performed using a modified phenol-chloroform protocol. Initially, the swabs were centrifuged (10 min at 13000 rpm) and the RNAlater was removed. Subsequently, the swabs were re-suspended in an extraction buffer and subjected to mechanical lysis using 3 mm stainless steel beads (15 min, 50 Hz; TissueLyser LT; Qiagen, Hilden, Germany). Following centrifugation, proteinase K was added, and the samples were incubated at 56 ^∘^C for 2 h. We then used a modified phenol-chloroform procedure to purify the DNA. Adjustments in reagent amounts and centrifugation times were made to accommodate the low biomass of the samples (see Appendix B).

### Library preparation

For gene library preparation the ”Illumina 16S Metagenomic Library Preparation Guide” (15044223 Rev.B) was followed. A multi-marker approach was employed, targeting the V4 region of the 16S ribosomal RNA (rRNA) using primers 515F (Parada) [81] and 806R (Apprill) [3]. Additionally, to enhance taxonomic coverage for eukaryotes and to prevent bacterial read contamination [56], the D8-D9 region of the 28S rRNA was targeted using the primers GA20F[6]/RM9Rb[66]. This approach allows for a comprehensive assessment of the eukaryotic microbiota, encompassing not only fungal communities, as with the classic ITS2 (Internal Transcribed Spacer 2), but also other microbial eukaryotes typically targeted by 18S rRNA [56, 75, 120].

Polymerase chain reactions (PCR) were performed were performed separately for each marker in a 25 $\upmu$l reaction volume, containing 5 $\upmu$l DNA, 12.5 $\upmu$l KAPA HiFi HotStart ReadyMix (KAPA Biosystems, MA, United States), 1 $\upmu$l of each primer (1 $\upmu$M), and 6 $\upmu$l of PCR-grade water. The PCR amplification conditions comprised an initial denaturation step at 95 ^∘^C for 3 min, followed by 30 cycles of denaturation at 95 ^∘^C for 30 s, annealing at 55 ^∘^C for 16 S rRNA and 60 ^∘^C for 28 S rRNA for 25 s, extension at 72 ^∘^C for 40 s, and a final extension step of 5 min at 72 ^∘^C. The presence and size of amplicons were verified using a 2% agarose gel. Subsequently, PCR products were purified using the Agencourt AMpure XP PCR purification system (Beckman Coulter, Brea, CA, United States), following the manufacturer’s instructions. To increase amplicon concentration, a second PCR was performed using the purified PCR products from the first PCR (PCR-1). The same PCR conditions were applied, except for reducing the annealing time to 20 s. The PCR-1 and PCR-2 products were pooled and subjected to another round of purification using the Agencourt AMpure XP PCR purification system.

The purified PCR products were sent to the CeBiTec sequencing centre at Bielefeld University for subsequent processing. Index-PCR was performed using Illumina Nextera XT V2 index kits. Quantification of sequencing libraries was conducted using the Fragment Analyzer (Agilent). Following quantification, the libraries were diluted and equimolarly pooled. The pooled amplicon libraries were then sequenced on a Illumina MiSeq platform (0.4% MiSeq run), applying the protocol for 2$\times$300 bp paired-end reads. In addition to the 235 biological samples, the final library pool comprised of two replicates of the $ZymoBIOMICS^{TM}$ Microbial Community Standard (D6300, Irvine, CA, USA), two $ZymoBIOMICS^{TM}$ Microbial Community DNA Standard (D6305), four environmental controls, five extraction blanks, and four PCR negatives. Negative controls were included to monitor potential contamination throughout the entire procedure, while positive controls served for posterior workflow validation.

### 16S rRNA sequence data processing

Demultiplexed Illumina sequence data were imported into QIIME2 (Quantitative Insights Into Microbial Ecology 2, version 2022.11 [16]. Quality assessment of the reads was performed by visualising quality plots. To filter out low-quality bases and infer Amplicon Sequencing Variants (ASVs), the Divisive Amplicon Denoising Algorithm pipeline (DADA2) was employed [22]. The forward and reverse sequences were truncated at 253 and 185 base pairs, respectively, with 20 base pairs trimmed from the 5’ end of the reads. Taxonomy was assigned to the ASVs using a naive Bayes taxonomic classifier trained on the SILVA SSU 138.1 database [86]. The classifier was built and trained using the REference Sequence annotation and CuRatIon Pipeline plugin (RESCRIPt) [89].

The data were imported into R version 4.2.2 [87] using the qiime2R package version 0.99.6 [15]. Negative controls (environmental, extraction, and PCR negatives) were included in the analysis. 578 sequence contaminants were identified and removed using the decontam package version 1.18 [35]. The ”prevalence” method, with a probability threshold of 0.1, was applied for contaminant removal. Data was imported back into QIIME2 and ASVs assigned to Mitochondria, Chloroplast, Vertebrata, Eukaryota, and unassigned taxa were filtered out using QIIME2. Only taxa present in more than one sample and samples containing a minimum frequency of 500 reads were retained for further analysis. The remaining ASVs were aligned using MAFFT [51] as implemented in the q2- alignment plugin. The aligned sequences were then used to construct a phylogeny with FastTree [85], as implemented in the q2-phylogeny plugin. To assess the performance of the pipeline, the q2-quality-control plugin was used. This allowed us to evaluate the accuracy with which the expected taxonomic composition, derived from the community standards, was reconstructed (The results of the microbial community standards analysis are presented in Appendix C). To visualise microbial community composition, taxa-bar plots were generated exclusively for the core taxa, defined as taxa present in at least 70% of the samples. (Detailed scripts and intermediate results can be found in Appendix D.)

### 28S rRNA sequence data processing

Demultiplexed Illumina sequence data were imported into R version 4.2.2. The data underwent initial processing using Cutadapt version 4.4 [72] to identify and remove locus-specific primers from both reads. Quality assessment was performed through visualising quality plots.

ASV inference was conducted using DADA2, based on the pipeline outlined in Callahan et al [23]. After quality assessment, the sequences were trimmed to eliminate low quality regions: forward reads were truncated to 230 base pairs, and reverse reads to 185 base pairs. Short sequences below 100 base pairs were removed. Paired-end sequences were merged. A high percentage of reads failed to merge due to the larger fragment resulting in a low base-pair overlap. In order to maximise data utilisation, the unmerged paired-end sequences were concatenated. Chimeras were subsequently removed, and the data were imported into QIIME2. A primer-region specific classifier was built and trained using RESCRIPt. Taxonomy was assigned using the naive Bayes taxonomic classifier trained on the SILVA LSU 138.1 database. The processed data was then imported back into R. The decontam pipeline was applied, 218 ASV contaminants were removed using the ”prevalence” method with a probability threshold of 0.1.

Similar to the 16 S rRNA analysis, taxonomy-based filtering (host reads, Mitochondria, Chloroplast, Vertebra and unassigned reads were removed), removal of unique features, and filtering of samples with fewer than 500 reads were performed in QIIME2. ASVs were aligned using MAFFT as implemented in the q2-alignment plugin, and phylogeny was constructed using FastTree through the q2-phylogeny plugin.

The performance of the pipeline was evaluated using the q2-quality-control plugin (Appendix C) and taxa-bar plots (core taxa only) were generated to visualise microbial community composition. (Detailed scripts and intermediate results can be found in Appendix D.)

### Alpha diversity

In Qiime2, rarefaction curves were generated using the q2-diversity-alpha-rarefaction plugin to assess sequencing depth and sample coverage. After inspection, the 16S rRNA dataset was rarefied at 4000 reads, while the 28S rRNA dataset was rarefied at 2000 reads (Appendix A Fig. S1–S3). Two measures of alpha diversity, the Shannon diversity index [97] and Faith’s Phylogenetic Diversity (Faith PD) [38], were calculated using the q2-diversity-alpha plugin. Correlations among the variables studied were assessed using the Pearson correlation coefficient as part of the correlation package version 0.8.4 in R [69]. To investigate individual differences in the gut microbiome, linear mixed models (LMMs) with a Gaussian distribution and identity link were performed using the lmer function from the lme4 package [9] in R. The significance of factors included in the models was tested using analysis of variance (ANOVA). This analysis aimed to examine the relationship between gut microbiota diversity, age and body condition index (BCI), while accounting for differences in habitat, sex, year, hatching sequence (rank), and *Leucocytozoon* infection. To incorporate the longitudinal nature of the dataset and the fact that individuals belonged to the same/different nests, Nest ID and Individual ID were included as nested random effects (Individual ID nested within Nest ID) as follows:

$$\begin{aligned}{} & {} \text {Microbiota diversity }\sim Age + BCI + Rank + Habitat + Sex + Year \\{} & {} \quad + Infection + (1|Nest ID/Individual ID) \end{aligned}$$

The significance of the fixed effects was assessed at $\alpha$ < 0.05. Significance of the random effects was tested using the ‘ranova” function from the lmerTest package [58]. Marginal and conditional R^2^ values were calculated using the MuMIn package [8]. Assumptions of normality and homogeneity of variance of the residuals were assessed through visual inspection of plots using the performance package [65] and furthermore analysed with the Shapiro-Wilk test. When necessary, the data were normality transformed. (For complete scripts and intermediate results, refer to Appendix E)

### Beta diversity

Microbiota community analysis was performed using R version 4.2.2. The unrarified dataset, excluding samples with fewer than 4000 reads for 16S rRNA and 2000 reads for 28S rRNA, was subjected to Cumulative Sum Scaling (CSS) normalisation [83] using the metagenomeSeq package version 1.30.0 [82] to account for uneven sequence coverage. Bray-Curtis dissimilarities (BC) [19] and weighted UniFrac distances (WU) [64] were then computed using the phyloseq package [76]. Our dataset violated some of the underlying assumptions of PERMANOVA, namely the exchangeability assumption under the null hypothesis (independence of variables) and the challenge of assessing homogeneity of variances with continuous variables [2]. We therefore adopted a Bayesian framework to model pairwise (dyadic) values, described by Raulo et al [88]. The analysis pipeline described at Analysing-dyadic-data-with-brms was implemented. Bayesian regression models were fitted using the brms package [21]. brms allows for the incorporation of random effect structures to account for dependence in dyadic data and repeated sampling [20]. The models included all pairwise sample comparisons except within-sample comparisons. BC and WU were used as response variables; matrices of nest sharing, habitat, sex, and year (all coded as 0/1 for different/same), age differences ($\Delta$Age), BCI differences ($\Delta$BCI) and infection status combination (II: infected-infected; INi: infected/non-infected; NiNi: non-infected/non-infected) were included as fixed effects. Continuous variables were either naturally scaled or transformed to range from 0 to 1. To control for data dependency resulting from pairwise comparisons and repeat samples per individual, two multi-membership random effects were included in the model: one capturing the individuals in each dyad (ID A + ID B) and another capturing the samples in each dyad (Sample A + Sample B):

$$\begin{aligned}{} & {} \text {Microbiota composition }\sim \Delta Age + \Delta BCI + Habitat(similarity) + Sex(similarity) \\{} & {} \quad + Nest(sharing) + Year(similarity) \\{} & {} \quad + Infection(combination) + (1|mm(sampleA,sampleB)) +(1|mm(IDA,IDB)) \end{aligned}$$

(Complete scripts and intermediate results in Appendix G)

### Differential abundance analysis

To identify key ASVs driving the observed patterns of alpha and beta diversity, we performed a differential abundance analysis (DAA) using an Analysis of Compositions of Microbiomes with Bias Correction 2 (ANCOM-BC2) implemented in the R package ANCOMBC version 2.0.2 [61, 62]. ANCOM-BC2 allows for model fitting to the analysis with the ”fix formula=” option: Age, BCI, Rank, Habitat, Sex, Year, and Infection were specified as fixed effects. Unlike most DAA methods, ANCOM-BC2 also enables control for random effects with the option ”rand formula”. Again a nested random effect to account for Nest ID and Individual ID was fitted to the analysis (1|NestID/Individual ID). As part of ANCOM-BC2, the Holm-Bonferroni method was employed to correct P values for multiple testing [44]. A significance cutoff of $p_{\text {adj}}$ < 0.05 was used. Default parameter settings were applied, and DAA was performed at ASV level. (Detailed scripts and intermediate results can be found in Appendix H)

## Results

### Gut microbiota composition

**Fig. 1:**
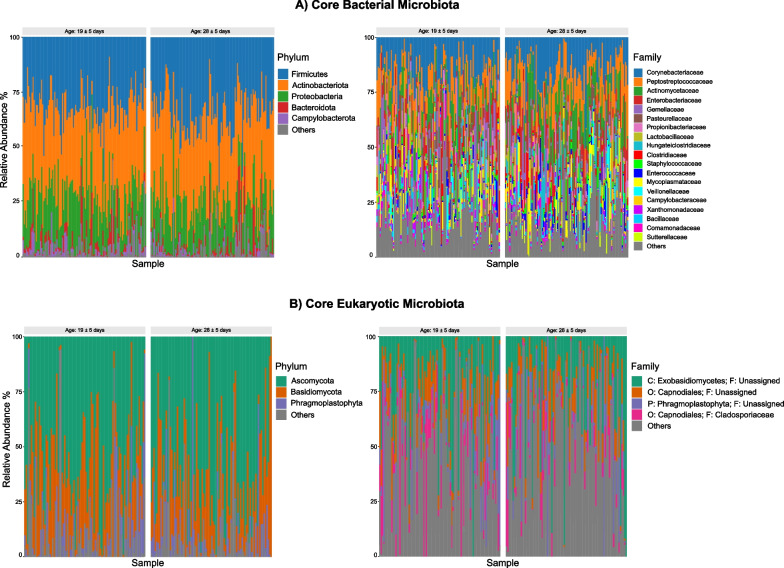
Relative abundances (as percentages) of: **A** core bacterial gut microbiota phyla and families and **B** core eukaryotic gut microbiota phyla and families in common buzzard nestlings. Each individual is represented at two different sampling ages. Core taxa are defined as microbial taxa present in at least 70% of the samples. *Note*: In Figure 1B, **P** refers to Phylum, **C** - Classe, **O** - Order and **F** - Family

After quality control and filtering steps, the 16S rRNA dataset consisted of 117 individuals (66 males; 51 females) with a total of 230 samples (mean = 2 samples per individual) from 54 different nests. On average 22,268 ± 8,764.14 reads per sample were obtained, resulting in the identification of 2,078 ASVs (amplicon sequencing variants) (Appendix A Table S3). Two microbial kingdoms were identified, Bacteria (99.7%, SD = 0.24%) and Archaea (0.03%, SD = 0.24%). A total of 25 bacterial phyla were detected, of which five phyla were identified as core taxa: *Firmicutes* (39.3%, SD = 10.9%), *Actinobacteria* (33.1%, SD = 10.1%), *Proteobacteria* (19.1%, SD = 8.4%), *Bacteroidota* (3.4%, SD = 6.0%), and *Campylobacterota* (2.1%, SD = 3.5%). 219 bacterial families were identified, 19 of which were classified as core taxa. Among these families, the most abundant ones were: *Corynebacteriaceae* (15.9%, SD = 9.3%), *Peptostreptococcaceae* (12.0%, SD = 8.5%), *Actinomycetaceae* (9.5%, SD = 9.1%), *Enterobacteriaceae* (6.5%, SD = 7.3%), and *Gemellaceae* (6.1%, SD = 10.7%) (Fig. 1A).

The 28S rRNA final dataset included 109 individuals (64 males; 45 females) and a total of 180 samples, representing 54 nests. The average number of reads per sample were 8,212 ± 4,231.45, resulting in the identification of 1770 ASVs (Appendix A Table S5). A total of 22 eukaryotic phyla were identified, with *Ascomycota* (57.1%, SD = 24.6%), *Basidiomycota* (25.4%, SD = 20.9%), and *Phragmoplastophyta* (7.3%, SD = 13.9%) being the core phyla. The majority of the core taxa could not be identified at family level. Among the 94 families, *Cladosporiaceae *(8.9%, SD = 17.7%) and unassigned families belonging to the class *Exobasidiomycetes *(23.8%, SD = 22.9%), order *Capnodiales* (11.4%, SD = 9.1%), phylum *Phragmoplastophyta* (8.9%, SD = 17.7%), were identified as the core taxa (Fig. 1B).

### Factors driving gut microbiota alpha diversity

**Fig. 2:**
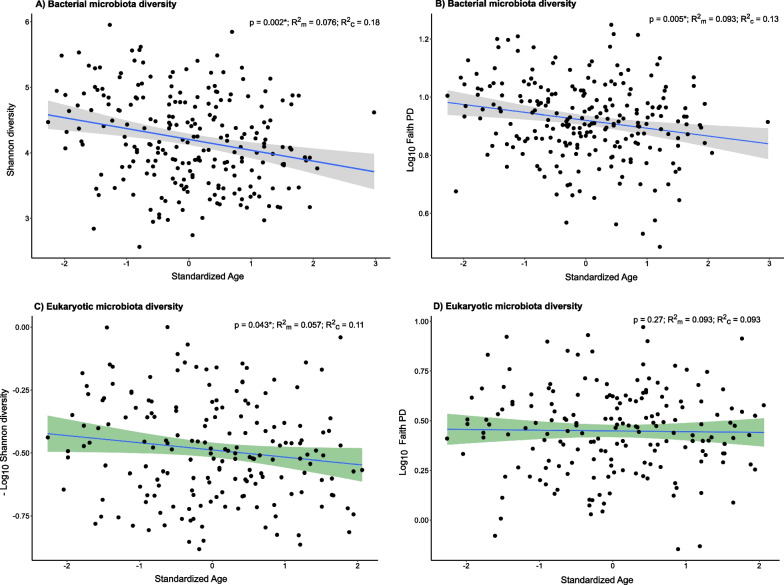
Effect of age on microbiome alpha diversity as obtained from a LMM. *p*-Values are shown for each diversity metric along with the variance explained by the fixed effects ($R^2_{\text {m}}$) and the total variance explained by the model ($R^2_{\text {c}}$) **A** Bacterial Shannon diversity. **B** Bacterial Faith PD. **C** Eukaryotic Shannon diversity. **D** Eukaryotic Faith PD

Age was the only factor that explained gut bacterial diversity. Both Shannon and Faith phylogenetic diversity decreased with age (Shannon: $\beta$ = $-$0.17, $\chi ^2$ = 9.76, p = 0.002; Faith PD: $\beta$ = $-$0.03, $\chi ^2$ = 7.82, p = 0.005) (Fig. 2A and B; Appendix A Table S8 & S9). The fixed effects of the model accounted for only a small portion of the diversity variation, with approximately 10% of the variability explained by the random effects ($LMM_{\text {Shannon}}$: $R^2_{\text {marginal}}$=0.075, $R^2_{\text {conditional}}$=0.176; $LMM_{\text {Faith PD}}$: $R^2_{\text {marginal}}$=0.092, $R^2_{\text {conditional}}$=0.128). However, the analysis of the significance of random effects revealed no evidence for variation driven by the grouping factors (Table 1). Similar patterns were found for eukaryotic microbial diversity. Shannon diversity decreased with age ($\beta$ = $-$0.036, $\chi ^2$ = 4.08, p = 0.043), while no evidence was found for Faith PD ($\beta$ = $-$0.020, $\chi ^2$ = 1.24, p = 0.23; Fig. 2C and D; Appendix A Table S10 & S11). Additionally, no significant evidence for variation driven by random effects was detected (Table 2). Evidence of an effect of habitat on Faith PD was found, but after adjusting for multiple comparisons with Benjamini-Hochberg method, habitat did not remain statistically significant ($p_{\text {adj}}$ > 0.05; Appendix A Table S12). Overall, our results suggest that age is the primary factor influencing gut microbiota diversity in common buzzard nestlings.

**Table 1:** Analysis of significance of the random effects (“ranova”) with bacterial alpha diversity as response variable

	LogLik	AIC	LRT	*p*-Value
*Shannon diversity*				
Individual ID: Nest ID	−237.86	501.71	0	1
Nest ID	−239.56	505.12	3.41	0.065
*Faith PD*				
Individual ID: Nest ID	112.74	−199.49	0	1
Nest ID	112.45	−198.91	0.58	0.45

LogLik = log-likelihood for the model, AIC = akaike information criterion, LRT = likelihood ratio test, *p*-value = error probability

**Table 2:** Analysis of significance of the random effects (”ranova”) with eukaryotic alpha diversity as response variable

	LogLik	AIC	LRT	p-value
*Shannon diversity*				
Individual ID: Nest ID	14.43	−2.87	0	1
Nest ID	14.15	−2.289	0.58	0.45
*Faith PD*				
Individual ID: Nest ID	5.00	15.99	0	1
Nest ID	5.00	15.99	0	1

LogLik = Log-likelihood for the model, AIC = Akaike information criterion, LRT = Likelihood ratio test, *p*-Value = error probability

### Gut microbiota beta diversity

**Fig. 3:**
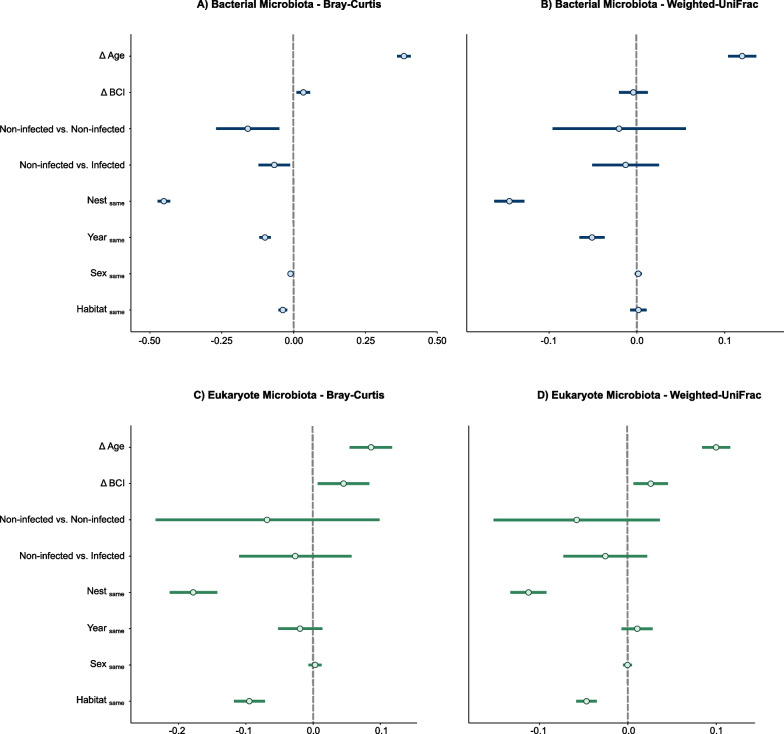
Effect size estimates (points) and corresponding 95% credible intervals (horizontal lines) derived from Bayesian regression (brms) models with pairwise Bray-Curtis dissimilarities and Weighted UniFrac distances as response variables. All predictors correspond to pairwise comparisons: nest sharing, habitat, sex, and year (different/same), $\Delta$Age (age differences), $\Delta$BCI (body condition index differences) and infection status (infected-infected; infected/non infected; non-infected/non-infected). A variable significantly predicts microbiota dissimilarity/distance when the credible intervals do not overlap zero

We found a substantial influence of age difference and nest sharing (among compared pairs) on the bacterial community structure (beta diversity). Bacterial microbiota composition became more distinct as the age difference between individuals increased ($\mu$ = 0.38,CI [0.36, 0.41]). Conversely, dissimilarity decreased among individuals from the same nest ($\mu _{\text {Same Nest}}$ = $-$0.45, CI [$-$0.48, $-$0.43]; Fig. 3A). In addition to age differences and nest sharing, we found evidence for the effects of $\Delta$BCI ($\mu$ = 0.03, CI [0.01, 0.06]), year ($\mu _{\text {Same year}}$ = $-$0.1, CI [$-$0.12, $-$0.08]) and habitat similarity ($\mu _{\text {Same Habitat}}$ = $-$0.04, CI [$-$0.05, $-$0.02]) on bacterial composition, albeit less pronounced (Fig. 3A). Notably, individuals with similar BCI, individuals from the same year, and occupying the same habitat tended to exhibit similar bacterial microbiota compositions. Sex similarity ($\mu _{\text {Same sex}}$ = $-$0.01, CI [$-$0.02, 0]) did not explain beta diversity (Fig. 3A). Blood parasite-infected individuals exhibited greater dissimilarities among themselves than did non-infected individuals among them ($\mu _{\text {Ni-Ni}}$ = $-$0.1, CI [$-$0.29, $-$0.03]). Moreover, within infected individuals dissimilarities were higher than dissimilarities between non-infected/infected pairs ($\mu _{\text {Ni-I}}$ = $-$0.1, CI [$-$0.13, $-$0.001]) (Fig. 3A). When considering phylogenetic relationships among bacterial communities (WU), credible intervals indicated an effect of age difference ($\mu$ = 0.12, CI [0.10, 0.14]), nest sharing ($\mu _{\text {Same Nest}}$ = $-$0.15, CI [$-$0.17, $-$0.13]), and year similarity ($\mu _{\text {Same year}}$ = $-$0.05, CI [$-$0.07, $-$0.03]) (Fig. 3B).

Similar but less pronounced trends were observed in the eukaryotic microbiota. In terms of Bray-Curtis dissimilarity, nest sharing ($\mu _{\text {Same Nest}}$ = $-$0.18, CI [$-$0.22, $-$0.14]) had the largest impact on compositional differences; $\Delta$age ($\mu$ = 0.09, CI [0.05, 0.12]), $\Delta$BCI ($\mu$ = 0.05, CI [0.0006, 0.09]), and habitat similarity exhibited weaker effects (Fig. 3C). When considering phylogenetic distances, nest sharing ($\mu _{\text {Same Nest}}$ = $-$0.11, CI [$-$0.14, $-$0.09]) and $\Delta$age ($\mu$ = 0.10, CI [0.08, 0.12]) remained as primary contributors to compositional differences, with $\Delta$BCI and habitat similarity ($\mu _{\text {Same Habitat}}$ = $-$0.09, CI [$-$0.12, $-$0.07]) showing weaker effects (Fig. 3D). No effects of year, sex, or blood parasite infection infection were observed (Fig. 3C and D). Complete Bayesian model diagnostics and results are available in the supplementary information, Appendix A Tables S13 to S16.

### Age-associated differential abundant taxa

**Fig. 4:**
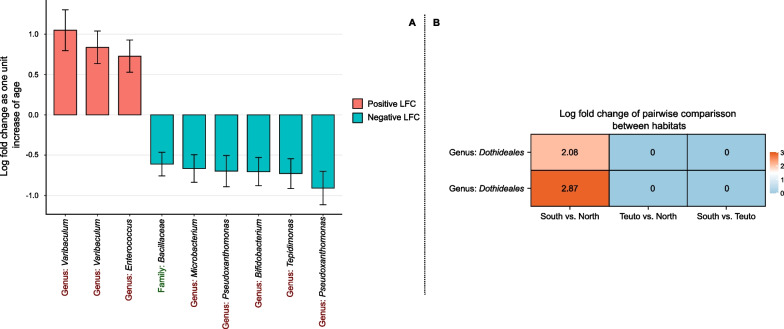
Differential abundance analysis using ANCOM-BC2. **A** Log fold changes (LFC) of deferentially abundant bacterial taxa with increasing age. **B** Habitat pairwise comparisons showing log-fold changes of deferentially abundant eukaryotic taxa

We identified nine differential abundant bacterial ASVs associated with age ($p_{\text {adj}}$ < 0.05; Appendix A Table S17). Among these, six ASVs displayed a considerable log-fold decrease with age and belonged to the genera *Microbacterium*, *Pseudoxanthomonas*, *Bifidobacterium*, and *Tepidomonas*, while one ASV was identified only at the family level, *Bacillaceae*. Conversely, three ASVs demonstrated a log-fold increase with age and were assigned with the genera *Varibaculum* and *Enterococcus* (Fig. 4A). No eukaryotic ASVs showed differential variation with age; however, two ASVs, both belonging to the order *Dothideales*, exhibited differential abundance between northern and southern habitats (Fig. 4B). No differential abundant taxa were found associated with any of the other studied variables.

## Discussion

Gut microbial communities acquired during early-life stages significantly impact host condition by influencing metabolism [26, 42] and the development of the nervous and immune systems [41, 52]. Investigating the driving factors behind the colonisation of the gut is therefore essential for gaining a comprehensive understanding of the broader implications of the gut microbiota on the health and fitness of the host. In this study, we analysed longitudinal data from a wild population of common buzzards nestlings and explored relationships among the gut microbiome and environmental and host factors. We found that age shapes both bacterial and eukaryotic gut microbiota diversity and composition, with the nesting environment playing a crucial role in shaping microbiota composition. Nestling condition affects gut microbial composition, while habitat influences both bacterial and eukaryotic communities. Additionally, we observed associations between *Leucocytozoon* infection and bacterial beta diversity, although no such differences were detected in the eukaryotic microbiota.

### Gut microbiota diversity is affected by nestling age

Our study revealed strong evidence for an influence of age on bacterial gut microbiota diversity, as evidenced by declines in both Shannon and Faith PD indices with increasing age (Fig. 2A and B). This suggests that, over time, certain bacterial taxa may become more dominant as nestlings age, leading to a reduction in overall diversity (competitive exclusion) [1, 29]. These dominant taxa also exhibit closer phylogenetic relationships, indicating potential specialisation or competitive advantages that drive their prevalence. While certain raptor studies have not found significant age-related effects [45, 107, 123], our findings align with studies on other species [34, 48, 115], suggesting a diverse gut microbiota in newborns due to rapid colonisation after hatching.

Maraci et al [71] reported fluctuating changes in gut microbiota diversity during ontogeny in zebra finches (*Taeniopygia guttata*) and Bengalese finches (*Lonchura striata domestica*). Our results from the differential abundance analysis reveal that some specific taxa exhibit a log-fold increase with age, while others decrease in abundance (Fig. 4A), providing additional support for these dynamic shifts in relation to age. These findings emphasise the importance of sampling an adequately extended time-frame to effectively capture variations in the gut microbiota diversity. Similarly, eukaryotic taxa showed a decline in Shannon diversity with age, although Faith PD showed no detectable effects (Fig. 2C and D). Unlike Shannon diversity, Faith PD is not weighted on abundance, which means that increasingly dominant taxa, responsible for reducing Shannon diversity, do not exert the same influence on Faith PD [38, 97]. These findings align with results from West et al [115], who documented a decline in mycobiota diversity with age in kākāpō chicks, mirroring the pattern previously identified in their bacterial microbiota study [115].

As birds typically hatch with little microbial diversity, this rapid initial colonisation most likely comes from the parents, either by direct contact or by food provisioning and passive exposure to the nesting environment [24, 109]. Buzzard nestlings fledge at around 35 days old [110]. During this period, parental care and presence (mainly by females) decrease gradually: from days 0–8 after hatching, active brooding occurs also outside of feeding sessions; from days 9–30, there is a decline in female presence and care, and by around 25–30 days, female care resembles that of males [46]. The decrease in microbiota diversity during this period may be related to reduced parental contact and dietary shifts [32, 43]. Empirical data suggests no major dietary shifts in the common buzzard, there are however shifts in feeding practices from direct provisioning to depositing prey in the nest resulting in nestlings transitioning from consuming individual pieces of prey to whole prey items. Additional work would be needed to test the impact of changing feeding habits on the development of the gut microbiota of buzzard chicks. Additionally, maturation of the immune system and other physiological systems can drive microbial selection leading to the observed patterns of decreasing diversity with age [108, 122].

### Age difference and nest similarity driving gut compositional differences

Age differences and nest similarity exhibited the most pronounced effects on beta diversity. We observed that compositional dissimilarities and phylogenetic distances increase with age difference and between distinct nests, a trend consistently evident in both bacterial and eukaryotic diversity (Fig. 3). Studies by Worsley et al [118, 119] found that age primarily impacted bacterial compositional differences. Similarly, in humans and other primates, compositional differences linked to age have been consistently observed [74, 102, 104]. Coupled with the identification of specific taxa showing increased abundance while others decrease with age (Fig. 4A), it is plausible to hypothesize that these changes may correspond to changing functional requirements across the developmental stages of buzzard nestlings [84, 90].

A plethora of studies have reported robust links between the nesting environment and chick gut microbiota [26, 28, 36, 98]. Our findings align with the ”nidobiome” concept, which integrates the collective effects of parents, nest conditions, and nestling interactions during microbiome assembly and their contribution over the developmental stages [24].

### Nestling condition shapes gut microbial composition but not diversity

Differences in nestling body condition impacted gut microbial composition, with both bacterial and eukaryotic communities exhibiting increased similarity among nestlings with similar body condition (Fig. 3). However, we did not detect individually significantly abundant taxa, indicating that compositional changes are relatively small and are distributed across the entire microbial community rather than being concentrated on a small number of taxa. Alterations in the microbiota community can potentially result in dysbiosis, which may have adverse effects on the host’s health [63]. This could explain the observed increased dissimilarities among nestlings with poor body condition compared to those in better health. However, an alternative plausible mechanism would be that changes in microbiota composition are a consequence of variation in individual body condition, considering that factors such as anatomy, physiology, and the immune system play pivotal roles in shaping gut microbial communities [31, 42]. The relationship between gut microbial composition and host body condition is intricate and varies across host species [42, 55]. Certain microbial taxa can either enhance or diminish host condition, depending on their interactions with the current diet, host immunity and other members of the microbiota [93]. A study conducted on the steppe buzzard (*Buteo buteo vulpinus*) showed no correlation between body condition and microbiota community composition, but it revealed a significant increase in the relative abundance of the genus *Escherichia-Shigella* with decreasing body condition [107]. Studies highlighting connections between eukaryotic gut microbiota and health primarily focus on economically important species [103], with a scarcity of direct connections observed in the wild. Nevertheless, research on the Seychelles warbler revealed an association between fungal microbiota and differential survival [119]. Our results add to a limited body of evidence suggesting that not only bacterial but also eukaryotic gut microbial communities are associated with host body condition.

In contrast with microbiota composition, no discernible connection was found between body condition and gut microbiota diversity. Gut microbiota diversity is influenced by a multitude of variables and these complex interactions may obscure direct links between body condition and microbiota diversity [42, 52, 105]. Furthermore, the potential time lag between changes in body condition and alterations in the gut microbiota might also contribute to the observed lack of association [33].

### Habitat and year affects microbiota composition

Habitat similarity also affected gut microbiota composition with individuals from the same habitat sharing similar compositions (Fig. 3). Interestingly, this effect was more pronounced within the eukaryotic communities, resulting in substantial impacts on both dissimilarity and phylogenetic measurements (Fig. 3C and D). The study site encompasses three distinct habitat types: the Teutoburg Forest (Teuto), a low mountain region where Norway Spruce (*Picea abies*), Beech (*Fagus sylvatica*), and Oak (*Quercus robur and Q. petraea*) are dominant; the northern habitat characterised by a prevalence of Beech and Oak; and the southern habitat marked by the dominance of Pine (*Pinus silvestris*) and sandy soils [50, 57]. It is plausible that variation in tree and soil types contributes to the observed differences in gut microbiota composition, possibly reflecting diverse ecological niches that influence microbial transmission dynamics [42, 105]. Variation in tree and soil types likely contribute to the observed differences in gut microbiota composition. This suggests that eukaryotic microbiota can be acquired both directly from the environment and indirectly through prey items (particularly the common vole, *Microtus arvalis*). Indeed fungi often have more specialised substrate requirements compared to bacteria [27]. Different fungal species may have specific host plants or environmental substrates they prefer, leading to a stronger association with certain habitats. In contrast, bacteria might be more generalist and thus adaptable to diverse habitats. Notably, core microbiota taxa such as *Exobasidiomycetes* species have been linked to abnormal plant tissue outgrowths [10], and *Cladosporiaceae* species are commonly found in soil and plant materials [11]. Supporting the influence of habitat differences on eukaryotic microbiota, we also observed differential abundance of *Dothideales* between Northern and Southern habitats. *Dothideales* species have strong affinities with conifer trees, particularly *Pinus* species [14], which are dominant in the South habitat.

Year similarity also had an influence on bacterial microbiota composition, with no discernible effects observed within the eukaryotic communities. Seasonal variations, presumably (linked to fluctuations in food availability and weather conditions) [42] and ecological drift (random changes in the frequency of different microbial taxa) likely contribute to these differences [92, 96].

Despite the effects on microbiota composition, sampling year and habitat showed no impact on gut microbiota diversity. Generally, annual variation in prey availability and divergent environmental factors can also contribute to fluctuations in gut microbial alpha diversity [45, 53, 104]. It is, nevertheless, important to acknowledge the limitations of our sample set. Only a small percentage of individuals were sampled in 2021 (10%) compared to 2020, and similarly, few individuals were sampled from the South (10%) and Teuto (<5%) habitats compared to the North habitat. Consequently, this reduces statistical power and our capacity to make inferences about detected effects, while also potentially limiting our ability to detect differences.

### Gut microbiota and *Leucocytozoon* infection

Severe malaria is caused by *Plasmodium* blood parasites, while related *Leucocytozoon* parasites can induce similar conditions in a wide range of avian hosts [5]. Despite a high prevalence (55%) of *Leucocytozoon* infection in buzzard nestlings previously found on this study site, research suggests low pathogenicity and minimal impacts on host condition [117]. Nevertheless, signatures of infection were found in haematological and blood chemistry profiles [117].

Our analysis revealed evidence that bacterial beta diversity, rather than alpha diversity, varied with infection status (Fig. 3A). Infected individuals displayed greater compositional dissimilarity among themselves than compared to non-infected nestlings. Furthermore, the compositional differences do not appear to be influenced by individually differential abundant taxa, but rather by overall changes across the community. No correlation was found between eukaryotic gut microbiota and infection status (Fig. 3C and D). In the context of infection, the host’s immune response can affect the gut microbiota composition; conversely, alterations in the microbiota following infection might signal the immune system to trigger a response [41, 60]. Mateos-Hernández et al [75] showed that altering the gut microbiota can enhance protection against avian aspergillosis by modulating anti-$\alpha$-Gal immunity. In the context of infection, the host’s immune response can significantly impact the gut microbiota composition, potentially leading to changes in the host microbiota [41].

Differences in beta diversity between bacterial and eukaryotic microbiota may arise from differential interactions with mucosal immunity (e.g. recognition mechanisms; immune cell responses), as well as differential microbiota-driven [41] signalling of the immune response [4, 18, 60]. Additionally, the higher abundance and broader range of functions performed by bacterial taxa might make small changes easier to detect [100].

While recognizing existing connections between gut microbiome and blood parasite infection [113], the mechanisms behind these relationships, especially among bird species, remain largely unknown. Although many studies find no correlations with alpha or beta diversity metrics, subtle links with specific taxa have been detected [7, 78, 91]. Further studies, particularly with controlled experimental designs, will be necessary to untangle the complex effects of parasite infection alongside other variables, while here we aim to shed light on population dynamics in their natural habitat.

### Sex and rank does not impact the gut microbiota

Neither bacterial nor eukaryotic alpha and beta diversity differed between sexes. There is no sex dimorphism in nestling common buzzards and sex-related differences in gut microbial diversity may become more apparent in adulthood, as the birds mature and experience hormonal changes associated with sexual development [42, 119].

Rank exhibited no influence on nestling gut microbiota diversity. Similar to sex, this could be attributed to the relatively short time frame of this study, which may not have allowed hierarchical differences among chicks to become evident. The nesting environment appears to have a homogenising effect on microbiota diversity across the brood, surpassing other potential factors like dominance rank in its influence [26, 36].

### Gut microbiota profile

There is a scarcity of studies investigating the microbiota composition of raptor species. Nevertheless, our findings align with a handful of existing studies, which show that *Firmicutes*, *Proteobacteria*, and *Actinobacteriota* (Fig. 1A) are among the most abundant bacterial phyla in raptor gut microbiota [73, 80, 107, 121]. Notably, while studies focusing on raptors lack descriptions of the eukaryotic microbiota, our results are in accordance with research conducted in other bird species that also revealed that the two major fungal phyla, *Ascomycota* and *Basidiomycota*, are predominant in the gut microbiota [68, 116, 119].

Our findings show that the core eukaryotic microbiota is predominantly dominated by fungal communities as the core microbiota (Fig. 1B). However, we did identify the presence of other eukaryotes, such as *Arthropoda* (4.8%, SD = 12.8%, most likely derived from diet, although not typically consumed by common buzzards), *Apicomplexa* (4.8%, SD = 12.8%), and *Annelida* (0.6%, SD = 3.0%), albeit in smaller abundances (Appendix A Table S6). These results show the utility of the 28S rRNA marker for studying the gut microbiome, while also highlighting the challenges in fully exploring the less abundant eukaryotic communities in the gut.

## Conclusion

We present a study into the gut microbiota of wild common buzzard nestlings, encompassing both bacterial and eukaryotic components. Using a longitudinal approach, we established that age is a critical factor shaping both bacterial and eukaryotic gut microbiota diversity and composition. These age-related shifts underscore the importance of capturing an adequate time frame that can help to disentangle the temporal dynamics of gut microbiota development. Moreover, our study highlights the importance of the nesting environment, with the ”nidobiome” concept aptly summarising the combined effects of parents, nest, and nestlings on microbiome assembly and interactions. Nestling condition emerges as a determinant of gut microbial composition, reflecting host health, and habitat plays a role in shaping not only bacterial but also eukaryotic communities. While we also observed a correlation between *Leucocytozoon* infection and bacterial beta diversity, no such differences were detected in the eukaryotic microbiota. These different outcomes may result from distinct interactions between the mucosal immune system, bacteria and eukaryotes.

## Supplementary information

Supplementary tables and figures can be found in Appendix  A

### Supplementary Information

Below is the link to the electronic supplementary material.

Supplementary file1 (PDF 514 kb)

Supplementary file1 (PDF 24 kb)

Supplementary file1 (PDF 257 kb)

Supplementary file1 (PDF 1093 kb)

Supplementary file1 (PDF 1408 kb)

Supplementary file1 (PDF 175 kb)

Supplementary file1 (PDF 13894 kb)

Supplementary file1 (PDF 216 kb)

## Data Availability

All 16S and 28S rRNA raw reads have been submitted to the European Nucleotide Archive repository, Project ID: PRJEB70791. The scripts and metadata to reproduce all analyses can be accessed via the GitHub repository: https://github.com/hugoeira/Gut-microbiota-of-buzzard-nestlings.

## Code available

Supplementary materials and can be accessed via the GitHub repository: https://github.com/hugoeira/gut-microbiota-of-buzzard-nestlings.
