# Supplementary material for: Early-life factors shaping the gut microbiota of Common buzzard nestlings
Source: Anim Microbiome. 2024 May 14;6:27. doi: 10.1186/s42523-024-00313-8 (PMC11092241; doi:10.1186/s42523-024-00313-8)
Supplement: Supplementary file 1 — Supplementary file1 (PDF 514 kb) [file 42523_2024_313_MOESM1_ESM.pdf]

Supplementary tables and figures

Table of contents

Supplementary tables and figures
- Table of contents
- Table S1. Summary of number of individuals per variable studied
  - Table S1.1 Age differences per individual between first and second sampling points
- Table S2. Summary of ASV table before filtering steps: 16s rRNA
  - Tabel S2.1. Summary of reads per sample before filtering steps: 16s rRNA data set
- Table S3. Summary of final ASV table: 16s rRNA
  - Tabel S3.1. Summary of final reads per sample: 16s rRNA data set
- Table S4. Summary of ASV table before filtering steps: 28s rRNA
  - Tabel S4.1. Summary of reads per sample before filtering steps: 28s rRNA data set
- Table S5. Summary of final ASV table: 28s rRNA
  - Table S5.1. Summary of final reads per sample: 28s rRNA data set
- Table S6. 16s rRNA ASVs relative abundances at Phylum level
  - Table S6.1 16s rRNA ASVs relative abundances at Family level
- Table S7. 28s rRNA ASVs relative abundances at Phylum level
  - Table S7.1 28s rRNA top 20 ASVs relative abundances at Family level
- Table S8. 16s rRNA Shannon diversity index LMM fit
- Table S9. 16s rRNA Faith PD LMM fit
- Table S10. 28s rRNA Shannon diversity index LMM fit
- Table S11. 28s rRNA Faith PD LMM fit
- Table S12. Multiple Comparisons of Means (Tukey Contrasts) for the habitat variable
- Table S13. 16s rRNA Bayesian model results for Bray-Curtis dissimilarities
- Table S14. 16s rRNA Bayesian model results for Weighted-UniFrac distances
- Table S15. 28s rRNA Bayesian model results for Bray-Curtis dissimilarities
- Table S16. 28s rRNA Bayesian model results for Weighted-UniFrac
- Table S17. Differential abundance analysis results for the age variable
- Figure S1. 16S rRNA rarefaction curves
- Figure S2. 28S rRNA rarefaction curves (sampling depth 20000)
- Figure S3. 28S rRNA rarefaction curves (sampling depth 2000)

Table S1. Summary of number of individuals per variable studied

	N individuals	N samples	Males	Females	2020	2021	Nests
**16s rRNA dataset**	117	230	66	51	107	10	54
**28s rRNA dataset**	109	180	64	45	100	6	54
	**Habitat North**	**Habitat South**	**Habitat Teuto**	**Rank 1**	**Rank 2**	**Rank 3**	**Rank 4**
**16s rRNA dataset**	101	12	4	54	45	17	1
**28s rRNA dataset**	94	12	3	51	40	17	1
	**Infected T1**	**Non-Infected T1**	**Infected T2**	**Non-Infected T2**			
**16s rRNA dataset**	54	57	76	40			
**28s rRNA dataset**	43	47	58	30			

Table S1.1 Age differences per individual between first and second sampling points

ID	Habitat	Nest	Year	Sex	Age difference
3121001	north	1374	2020	F	8
3121003	north	1914	2020	M	8
3121004	north	1914	2020	F	7
3121005	north	1914	2020	F	7
3121006	north	1156	2020	F	10
3121007	north	1156	2020	F	9
3121008	north	2165B	2020	F	9
3121009	north	2165B	2020	F	9
3121010	north	2165B	2020	F	9
3121011	north	2364C	2020	M	9
3121012	north	2364C	2020	F	8
3121013	north	2364C	2020	M	6
3121014	north	2257E1	2020	F	9
3121015	north	2257E1	2020	M	8
3121018	north	1427	2020	M	8
3121019	north	1427	2020	M	6
3121020	north	2289C	2020	F	10
3121021	north	2289C	2020	M	8
3121028	north	2347C	2020	F	8
3121040	south	1817	2020	M	9
3121041	south	1817	2020	F	10
3121045	north	681	2020	F	8
3121051	north	342	2020	M	8
3121052	north	342	2020	F	9
3121053	north	342	2020	F	8
3121054	north	585	2020	F	7
3121055	north	585	2020	F	7
3121056	north	585	2020	M	6
3121057	north	778E	2020	M	7
3121058	north	778E	2020	F	8
3121061	north	2239A	2020	M	8
3121062	north	2239A	2020	F	8
3121063	north	1541	2020	M	9
3121066	north	2235B	2020	F	9
3121067	north	2235B	2020	M	8
3121068	north	2235B	2020	F	8
3121072	north	2331C	2020	F	8

ID	Habitat	Nest	Year	Sex	Age difference
3121073	north	2331C	2020	M	8
3121074	north	2331C	2020	M	6
3121075	north	1611	2020	M	8
3121076	north	1611	2020	M	7
3121077	north	2261E1	2020	M	7
3121078	north	2261E1	2020	M	8
3121079	north	2263E1	2020	M	7
3121080	north	2263E1	2020	F	6
3121088	north	1103E	2020	F	7
3121089	north	1103E	2020	F	7
3121091	north	2259A	2020	F	7
3121092	north	2259A	2020	M	8
3121093	north	2259A	2020	F	8
3121094	north	2259A	2020	M	9
3121096	north	2265E	2020	F	9
3121098	north	2265E	2020	M	10
3121101	north	1102E	2020	F	9
3121102	north	1102E	2020	F	9
3121108	north	2016E	2020	F	10
3121109	north	2016E	2020	M	9
3121121	north	273	2020	M	8
3121136	teuto	2048E	2020	M	13
3121138	north	1860	2020	M	7
3121141	north	2263A	2020	M	9
3121142	north	2263A	2020	M	8
3121200	south	1810	2020	M	8
3121208	south	3061B	2020	M	8
3121209	south	3061B	2020	M	7
3121210	south	21392	2020	M	7
3121211	south	21392	2020	M	6
3121212	south	2264D	2020	M	6
3121226	north	1934	2020	F	8
3121227	north	1934	2020	M	8
3121228	north	1988E	2020	F	7
3121229	north	1988E	2020	M	8
3121254	north	1196	2020	F	15
3121257	south	1810	2020	M	8
3121263	teuto	1847	2020	F	7
3121264	teuto	1847	2020	M	7
3121274	north	2196B	2020	M	8

ID	Habitat	Nest	Year	Sex	Age difference
3121275	north	2196B	2020	M	8
3121280	north	1124E	2020	M	7
3121404	north	1104E	2021	M	11
3121405	north	1104E	2021	M	11
3121407	north	4051E	2021	M	11
3121408	north	4051E	2021	M	12
3121409	north	2011E	2021	F	11
3121410	north	2011E	2021	M	12
3121421	teuto	4028C	2021	M	12
3121429	north	2260A	2021	M	3
3121431	north	2260A	2021	F	2
3419275	north	2260E	2020	M	14
3419276	north	2260E	2020	M	14
3419277	north	2260E	2020	F	14
3419278	north	20771	2020	M	10
3419279	north	20771	2020	M	10
3419280	north	20771	2020	F	11
3419281	north	1500	2020	F	8
3419282	north	1500	2020	M	8
3419283	north	1500	2020	M	8
3419284	north	2202B	2020	F	10
3419285	north	2202B	2020	F	10
3419286	north	1231E	2020	M	8
3419287	north	1231E	2020	M	7
3419288	north	1231E	2020	F	8
3419289	north	1268E	2020	M	9
3419290	north	1268E	2020	M	9
3419291	north	1268E	2020	M	9
3419292	south	2188A	2020	M	17
3419293	south	2188A	2020	F	16
3419294	south	3017B	2020	M	18
3419297	north	201	2020	F	8
3419298	north	201	2020	M	10
3419299	north	201	2020	F	9
3419300	north	1374	2020	M	7
3121430	north	2260A	2021	F	3
3121064	north	1541	2020	F	10
3121097	north	2265E	2020	F	10
3121103	north	1102E	2020	M	9

Table S2. Summary of ASV table before filtering steps: 16s rRNA

	Sample
**Number of samples**	252
**Number of features (ASVs)**	10,065
**Total frequency**	6,319,664

Tabel S2.1. Summary of reads per sample before filtering steps: 16s rRNA data set

	Frequency
**Minimum frequency**	0
**1st quartile**	19,110.25
**Median frequency**	25,091.50
**3rd quartile**	31,849.25
**Maximum frequency**	50,886.00
**Mean frequency**	25,078.03

Table S3. Summary of final ASV table: 16s rRNA

	Sample
**Number of samples**	230
**Number of features (ASVs)**	2,078
**Total frequency**	5,121,868

Tabel S3.1. Summary of final reads per sample: 16s rRNA data set

	Frequency
**Minimum frequency**	4,295.00
**1st quartile**	16,586.25
**Median frequency**	22,162.00
**3rd quartile**	27,903.50
**Maximum frequency**	43,074.00
**Mean frequency**	22,268.99

Table S4. Summary of ASV table before filtering steps: 28s rRNA

	Sample
**Number of samples**	252
**Number of features (ASVs)**	14,143
**Total frequency**	2,710,821

Tabel S4.1. Summary of reads per sample before filtering steps: 28s rRNA data set

	Frequency
**Minimum frequency**	0
**1st quartile**	6,773.50
**Median frequency**	9,894.00
**3rd quartile**	15,024.00
**Maximum frequency**	33,905.00
**Mean frequency**	10,757.23

Table S5. Summary of final ASV table: 28s rRNA

	Sample
**Number of samples**	180
**Number of features (ASVs)**	1,770
**Total frequency**	1,478,279

Table S5.1. Summary of final reads per sample: 28s rRNA data set

	Frequency
**Minimum frequency**	2,036.00
**1st quartile**	5,388.25
**Median frequency**	7,138.50
**3rd quartile**	9,985.75
**Maximum frequency**	33,249.00
**Mean frequency**	8,212.66

Table S6. 16s rRNA ASVs relative abundances at Phylum level

Phylum	Mean abundance (%)	SD (%)
Firmicutes	39.3	10.9
Actinobacteriota	33.1	10.1
Proteobacteria	19.1	8.4
Bacteroidota	3.4	6
Campylobacterota	2.2	3.5

Table S6.1 16s rRNA ASVs relative abundances at Family level

Family	Mean abundance (%)	SD (%)
Corynebacteriaceae	15.9	9.3
Peptostreptococcaceae	11.9	8.5
Actinomycetaceae	9.4	9
Enterobacteriaceae	6.4	7.3
Gemellaceae	6.1	10.7
Pasteurellaceae	3.8	6.5
Propionibacteriaceae	3.2	4.1
Lactobacillaceae	2.7	4.5
Hungateiclostridiaceae	2.6	5.5
Clostridiaceae	2.2	4.7
Staphylococcaceae	2.2	3
Enterococcaceae	2.2	3.3
Mycoplasmataceae	2.2	5.3
Veillonellaceae	2.1	4.6
Campylobacteraceae	2	3.3
Xanthomonadaceae	1.6	2.2
Bacillaceae	1.6	2
Comamonadaceae	1.5	1.9
Sutterellaceae	1.3	3

Table S7. 28s rRNA ASVs relative abundances at Phylum level

Phylum	Mean abundance (%)	SD (%)
Ascomycota	57.1	24.6
Basidiomycota	25.4	20.9
Phragmoplastophyta	7.3	13.9
Arthropoda	4.8	12.8
Apicomplexa	2.1	12.4
Annelida	0.6	3
Discicristata	0.6	7.5
Chytridiomycota	0.5	2.4
Mucoromycota	0.5	2.1
Ciliophora	0.3	1.4
Nematozoa	0.2	1.5
Eukaryota	0.1	0.9
Klebsormidiophyceae	0.1	0.8
Chlorophyta	0.1	0.5
Amoebozoa	0.1	0.6
Cercozoa	0.1	0.3

Phylum	Mean abundance (%)	SD (%)
Peronosporomycetes	0.1	0.5

Table S7.1 28s rRNA top 20 ASVs relative abundances at Family level

Family	Mean_abundance (%)	SD (%)
C_Exobasidiomycetes;F_Unassigned	23.8	22.9
O_Capnodiales;F_Unassigned	11.4	9.1
P_Phragmoplastophyta;F_Unassigned	8.9	17.7
Cladosporiaceae	8.7	14.7
Debaryomycetaceae	6.9	16.9
O_Dothideales;F_Unassigned	5.9	12.5
O_Diaporthales;F_Unassigned	3.3	8.3
Mycosphaerellaceae	2.5	5.3
O_Diptera;F_Unassigned	2.4	12.2
O_Helotiales;F_Unassigned	2.2	4.1
O_Tremellales;F_Unassigned	1.9	4.9
O_Pleosporales;F_Unassigned	1.7	5.7
O_Cryptosporida;F_Unassigned	1.4	10.8
O_Hypocreales;F_Unassigned	1.1	5.3
Pichiaceae	1.1	6.5
Acari;F_Unassigned	1	4.4
Aspergillaceae	1	6.4
Pleosporaceae	1	4
Eimeriorina	0.9	7

P: Phylum; C: Classe; O: Order: F: Family

Table S8. 16s rRNA Shannon diversity index LMM fit

**Random effects:**			
**Groups**	**Name**	**Variance**	**Std.Dev.**
**Individual ID : Nest ID**	(Intercept)	0	0
**Nest ID**	(Intercept)	0.04932	0.2221
**Residual**		0.4071	0.638
**Fixed effects:**			
	**Estimate**	**Std. Error**	**t value**
**(Intercept)**	4.3068106	0.1058171	40.701
**Std_age**	-0.1652697	0.0529112	-3.124
**Std_BCI**	0.0001679	0.051195	0.003

**Rank(2)**	-0.0925053	0.096437	-0.959
**Rank(3)**	-0.1613338	0.1423461	-1.133
**Rank(4)**	0.2139437	0.507752	0.421
**Sex(M)**	-0.1144732	0.0963586	-1.188
**Year(2021)**	0.0683771	0.1981118	0.345
**Habitat (South)**	-0.1657539	0.1744792	-0.95
**Habitat (Teuto)**	-0.2976098	0.2869742	-1.037
**Infection-status (infected)**	0.078734	0.1036844	0.759
**Analysis of Deviance Table (ANOVA)**			
Response: shannon_entropy	**Chisq**	**Df**	**Pr(>Chisq)**
**Std_age**	**9.7564**	**1**	**0.001787 ** **
**Std_BCI**	0	1	0.997383
**Rank**	2.0552	3	0.561028
**Sex**	1.4113	1	0.234836
**Year**	0.1191	1	0.729986
**Habitat**	1.76	2	0.414783
**Infection-status**	0.5766	1	0.447636

Table S9. 16s rRNA Faith PD LMM fit

**Random effects:**			
**Groups**	**Name**	**Variance**	**Std.Dev.**
**Individual ID : Nest ID**	(Intercept)	0	0
**Nest ID**	(Intercept)	0.0006666	0.02582
**Residual**		0.016499	0.12845
**Fixed effects:**			
	**Estimate**	**Std. Error**	**t value**
**(Intercept)**	0.9407717	0.0199731	47.102
**Std_age**	-0.0285771	0.0102204	-2.796
**Std_BCI**	-0.00676	0.0097165	-0.696
**Rank(2)**	-0.0189343	0.0192559	-0.983
**Rank(3)**	-0.0154191	0.0279228	-0.552
**Rank(4)**	0.23475	0.0988584	2.375
**Sex(M)**	-0.0360834	0.01867	-1.933
**Year(2021)**	-0.0134265	0.0354902	-0.378
**Habitat (South)**	-0.002979	0.0315251	-0.094
**Habitat (Teuto)**	0.0008674	0.0526736	0.016

**Infection-status (infected)**	0.0146748	0.0200224	0.733
**Analysis of Deviance Table (ANOVA)**			
Response: log_faith	**Chisq**	**Df**	**Pr(>Chisq)**
**Std_age**	**7.8181**	**1**	**0.005173 ****
**Std_BCI**	0.484	1	0.486605
**Rank**	7.562	3	0.055986 .
**Sex**	3.7353	1	0.053274 .
**Year**	0.1431	1	0.705197
**Habitat**	0.0098	2	0.995125
**Infection-status**	0.5372	1	0.463609

Table S10. 28s rRNA Shannon diversity index LMM fit

**Random effects:**			
**Groups**	**Name**	**Variance**	**Std.Dev.**
**Individual ID : Nest ID**	(Intercept)	2.03E-09	4.51E-05
**Nest ID**	(Intercept)	2.24E-03	4.73E-02
**Residual**	3.75E-02	1.94E-01	
**Fixed effects:**			
	**Estimate**	**Std.Error**	**t value**
**(Intercept)**	-0.520591	0.034071	-15.28
**Std_age**	-0.035594	0.017628	-2.019
**Std_BCI**	-0.004052	0.016615	-0.244
**Sex(M)**	0.016542	0.032398	0.511
**Rank(2)**	-0.004258	0.033979	-0.125
**Rank(3)**	0.029871	0.045397	0.658
**Rank(4)**	0.21061	0.152755	1.379
**Habitat (South)**	0.068654	0.053602	1.281
**Habitat (Teuto)**	0.112459	0.108764	1.034
**Year(2021)**	0.003454	0.061376	0.056
**Infection-status (infected)**	0.019645	0.034963	0.562
**Analysis of Deviance Table (ANOVA)**			
	**Chisq**	**Df**	**Pr(>Chis)**
**Std_age**	**4.0769**	**1**	**0.04347 ***
**Std_BCI**	0.0595	1	0.80735
**Sex**	0.2607	1	0.60965

**Rank**	2.3064	3	0.51129
**Habitat**	2.5173	2	0.28404
**Year**	0.0032	1	0.95512
**Infection-status**	0.3157	1	0.57421

Table S11. 28s rRNA Faith PD LMM fit

**Random effects:**			
**Groups**	**Name**	**Variance**	**Std.Dev.**
**Individual ID : Nest ID**	(Intercept)	0	0
**Nest ID**	(Intercept)	0	0
**Residual**		0.04422	0.2103
**Fixed effects:**			
	**Estimate**	**Std. Error**	**t value**
**(Intercept)**	0.403635	0.035173	11.476
**Std_age**	-0.020558	0.018479	-1.113
**Std_BCI**	-0.019744	0.017132	-1.152
**Rank(2)**	0.007209	0.036431	0.198
**Rank(3)**	-0.014333	0.048106	-0.298
**Rank(4)**	0.315343	0.158692	1.987
**Sex(M)**	0.008349	0.033891	0.246
**Year(2021)**	0.004901	0.060742	0.081
**Habitat (South)**	0.094678	0.053631	1.765
**Habitat (Teuto)**	0.219712	0.113001	1.944
**Infection-status (infected)**	0.043	0.036659	1.173
**Analysis of Deviance Table (ANOVA)**			
	**Chisq**	**Df**	**Pr(>Chisq)**
**Std_age**	1.2377	1	0.26591
**Std_BCI**	1.3281	1	0.24914
**Rank**	4.3856	3	0.22272
**Sex**	0.0607	1	0.8054
**Year**	0.0065	1	0.93569
**Habitat**	**6.3988**	**2**	**0.04079 ***
**Infection-status**	1.3759	1	0.24081

Table S12. Multiple Comparisons of Means (Tukey Contrasts) for the habitat variable

Linear Hypotheses:				
	**Estimate**	**Std. Error**	**z value**	**Pr(>|z|)**
**south - north == 0**	0.09468	0.05363	1.765	0.116
**teuto - north == 0**	0.21971	0.113	1.944	0.116
**teuto - south == 0**	0.12503	0.12123	1.031	0.302

(Adjusted p values reported -- BH method)

Table S13. 16s rRNA Bayesian model results for Bray-Curtis dissimilarities

**Group-Level Effects:**							
**mmIDAIDB (Number of levels: 117)**							
	**Estimate**	**Est.Error**	**l-95% CI**	**u-95% CI**	**Rhat**	**Bulk_ESS**	**Tail_ESS**
**sd(Intercept)**	0.25	0.05	0.13	0.34	1	2990	2652
**mmsampleAsampleB (Number of levels: 226)**							
	**Estimate**	**Est.Error**	**l-95% CI**	**u-95% CI**	**Rhat**	**Bulk_ESS**	**Tail_ESS**
**sd(Intercept)**	0.4	0.03	0.35	0.47	1	5918	8058
**Population-Level Effects**							
	**Estimate**	**Est.Error**	**l-95% CI**	**u-95% CI**	**Rhat**	**Bulk_ESS**	**Tail_ESS**
**Intercept**	1.06	0.05	0.97	1.15	1	35066	30616
**Age_difference**	0.38	0.01	0.36	0.41	1	58577	25412
**BCI_difference**	0.03	0.01	0.01	0.06	1	59493	25555
	Frequency		---------------------	:-------:		**Minimum frequency**	0
**Infection-status-combination (Ni-I)**	-0.07	0.03	-0.13	-0.001	1	21753	26841
**Nest-sharing (same nest)**	-0.45	0.01	-0.48	-0.43	1	59044	27921
**Year-similarity (same year)**	-0.1	0.01	-0.12	-0.08	1	59803	26444
**Sex-similarity (same sex)**	-0.01	0	-0.02	0	1	58233	26359
**Habitat-similarity (same habitat)**	-0.04	0.01	-0.05	-0.02	1	55160	26715
**Family Specific Parameters**							
	**Estimate**	**Est.Error**	**l-95% CI**	**u-95% CI**	**Rhat**	**Bulk_ESS**	**Tail_ESS**
**phi**	60.34	0.53	59.3	61.37	1	55129	25683

Ni-Ni: pairwise comparisons within non-infected individuals
Ni-I: pairwise comparisons between infected and non-infected individuals
Age_difference: between compared pairs of individuals
BCI_difference: body condition differences between compared pairs of individuals
Nest-sharing: pairwise comparisons between individuls of same/diferent nests
Sex-similarity: pairwise comparisons between individuals of same/different sex
Habitat-similarity: pairwise comparisons between individuals of same/different habitat

Table S14. 16s rRNA Bayesian model results for Weighted-UniFrac distances

**Group-Level Effects:**							
**mmIDAIDB (Number of levels: 117)**							
	**Estimate**	**Est.Error**	**l-95% CI**	**u-95% CI**	**Rhat**	**Bulk_ESS**	**Tail_ESS**
**sd(Intercept)**	0.12	0.05	0.01	0.2	1	1090	1738
**mmsampleAsampleB (Number of levels: 226)**							
	**Estimate**	**Est.Error**	**l-95% CI**	**u-95% CI**	**Rhat**	**Bulk_ESS**	**Tail_ESS**
**sd(Intercept)**	0.3	0.02	0.26	0.34	1	2450	7741
**Population-Level Effects**							
	**Estimate**	**Est.Error**	**l-95% CI**	**u-95% CI**	**Rhat**	**Bulk_ESS**	**Tail_ESS**
**Intercept**	-2.45	0.03	-2.51	-2.38	1	17900	25545
**Age_difference**	0.12	0.01	0.1	0.14	1	67396	27034
**BCI_difference**	0	0.01	-0.02	0.02	1	66325	26781
**Infection-status-combination (Ni-Ni)**	-0.02	0.05	-0.11	0.07	1	10353	17451
**Infection-status-combination (Ni-I)**	-0.01	0.02	-0.06	0.03	1	10461	17112
**Nest-sharing (same nest)**	-0.15	0.01	-0.17	-0.13	1	66733	28983
**Sex-similarity (same sex)**	0	0	0	0.01	1	60472	27160
**Year-similarity (same year)**	-0.05	0.01	-0.07	-0.03	1	69858	29156
**Habitat-similarity (same habitat)**	0	0.01	-0.01	0.01	1	66748	28899
**Family Specific Parameters**							
	**Estimate**	**Est.Error**	**l-95% CI**	**u-95% CI**	**Rhat**	**Bulk_ESS**	**Tail_ESS**
**phi**	358.85	3.16	352.67	365.09	1	64829	27654

Ni-Ni: pairwise comparisons within non-infected individuals
Ni-I: pairwise comparisons between infected and non-infected individuals
Age_difference: between compared pairs of individuals
BCI_difference: body condition differences between compared pairs of individuals
Nest-sharing: pairwise comparisons between individuls of same/diferent nests
Sex-similarity: pairwise comparisons between individuals of same/different sex
Habitat-similarity: pairwise comparisons between individuals of same/different habitat

Table S15. 28s rRNA Bayesian model results for Bray-Curtis dissimilarities

**Group-Level Effects:**							
**mmIDAIDB (Number of levels: 117)**							
	**Estimate**	**Est.Error**	**l-95% CI**	**u-95% CI**	**Rhat**	**Bulk_ESS**	**Tail_ESS**
**sd(Intercept)**	0.16	0.1	0.01	0.36	1.01	783	2381
**mmsampleAsampleB (Number of levels: 226)**							
	**Estimate**	**Est.Error**	**l-95% CI**	**u-95% CI**	**Rhat**	**Bulk_ESS**	**Tail_ESS**
**sd(Intercept)**	0.65	0.04	0.57	0.73	1	3000	6849
**Population-Level Effects**							
	**Estimate**	**Est.Error**	**l-95% CI**	**u-95% CI**	**Rhat**	**Bulk_ESS**	**Tail_ESS**
**Intercept**	1.6	0.07	1.45	1.74	1	10560	19316
**Age_difference**	0.09	0.02	0.05	0.12	1	80195	27859
**BCI_difference**	0.05	0.02	0.0006	0.09	1	85005	26947
**Infection-status-combination (Ni-Ni)**	-0.07	0.1	-0.27	0.13	1	8718	15364
**Infection-status-combination (Ni-I)**	-0.03	0.05	-0.13	0.07	1	8771	15815
**Nest-sharing (same nest)**	-0.18	0.02	-0.22	-0.14	1	79725	29045
**Sex-similarity (same sex)**	0	0.01	-0.01	0.01	1	78734	26540
**Year-similarity (same year)**	-0.02	0.02	-0.06	0.02	1	72869	31147
**Habitat-similarity (same habitat)**	-0.09	0.01	-0.12	-0.07	1	71270	28517
**Family Specific Parameters**							
	**Estimate**	**Est.Error**	**l-95% CI**	**u-95% CI**	**Rhat**	**Bulk_ESS**	**Tail_ESS**
**phi**	47.1	0.52	46.09	48.12	1	81005	27166
**zoi**	0.01	0	0	0.01	1	74765	26599
**coi**	0.99	0.01	0.96	1	1	46936	21236

Ni-Ni: pairwise comparisons within non-infected individuals
Ni-I: pairwise comparisons between infected and non-infected individuals
Age_difference: between compared pairs of individuals
BCI_difference: body condition differences between compared pairs of individuals
Nest-sharing: pairwise comparisons between individuls of same/diferent nests
Sex-similarity: pairwise comparisons between individuals of same/different sex
Habitat-similarity: pairwise comparisons between individuals of same/different habitat

Table S16. 28s rRNA Bayesian model results for Weighted-UniFrac

**Group-Level Effects:**							
**mmIDAIDB (Number of levels: 117)**							
	**Estimate**	**Est.Error**	**l-95% CI**	**u-95% CI**	**Rhat**	**Bulk_ESS**	**Tail_ESS**
**sd(Intercept)**	0.2	0.06	0.05	0.3	1	2473	2493

**mmsampleAsampleB (Number of levels: 226)**							
	**Estimate**	**Est.Error**	**l-95% CI**	**u-95% CI**	**Rhat**	**Bulk_ESS**	**Tail_ESS**
**sd(Intercept)**	0.3	0.03	0.25	0.37	1	3590	5450
**Population-Level Effects**							
	**Estimate**	**Est.Error**	**l-95% CI**	**u-95% CI**	**Rhat**	**Bulk_ESS**	**Tail_ESS**
**Intercept**	-1.93	0.04	-2.01	-1.85	1	22627	27001
**Age_difference**	0.1	0.01	0.08	0.12	1	94893	27585
**BCI_difference**	0.03	0.01	0.003	0.05	1	93067	26189
**Infection-status-combination (Ni-Ni)**	-0.06	0.06	-0.17	0.05	1	11669	20499
**Infection-status-combination (Ni-I)**	-0.03	0.03	-0.08	0.03	1	11780	21192
**Nest-sharing (same nest)**	-0.11	0.01	-0.14	-0.09	1	88616	27856
**Sex-similarity (same sex)**	0	0	-0.01	0.01	1	89743	26096
**Year-similarity (same year)**	0.01	0.01	-0.01	0.03	1	90232	28801
**Habitat-similarity (same habitat)**	-0.05	0.01	-0.06	-0.03	1	92715	27700
**Family Specific Parameters**							
	**Estimate**	**Est.Error**	**l-95% CI**	**u-95% CI**	**Rhat**	**Bulk_ESS**	**Tail_ESS**
**phi**	254.78	2.81	249.27	260.3	1	86114	27141

Ni-Ni: pairwise comparisons within non-infected individuals
Ni-I: pairwise comparisons between infected and non-infected individuals
Age_difference: between compared pairs of individuals
BCI_difference: body condition differences between compared pairs of individuals
Nest-sharing: pairwise comparisons between individuls of same/diferent nests
Sex-similarity: pairwise comparisons between individuals of same/different sex
Habitat-similarity: pairwise comparisons between individuals of same/different habitat

Table S17. Differential abundance analysis results for the age variable

taxon	lfc_std_age	se_std_age	W_std_age	p_std_age	q_std_age	diff_std_age
**51ad0deb6331cb77f17cde5970059539**	**0.73**	**0.20**	**3.65**	**0.00**	**0.04**	**TRUE**
**eaf0ffd15882c0410c527effbe35f17e**	**-0.61**	**0.15**	**-4.17**	**0.00**	**0.00**	**TRUE**
**c287db728494cd6027afb0494a103927**	**-0.70**	**0.18**	**-4.01**	**0.00**	**0.01**	**TRUE**
**799f6ae8d556107ff50a68b663e4b663**	**-0.66**	**0.17**	**-3.86**	**0.00**	**0.02**	**TRUE**
**af9942270500e33a4c495ffa5dbc42eb**	**1.05**	**0.25**	**4.14**	**0.00**	**0.01**	**TRUE**
**2f08c5a0c0fecd2166cafb8e15e50202**	**0.84**	**0.20**	**4.12**	**0.00**	**0.01**	**TRUE**
**92e1de7ddb7eed4bd11841dac5c32fb1**	**-0.73**	**0.18**	**-3.96**	**0.00**	**0.01**	**TRUE**
**fb26c90c0b2afc91efe361f689f6b1ca**	**-0.70**	**0.19**	**-3.59**	**0.00**	**0.05**	**TRUE**
**6428019f5f4085ec42c0dc69e2cb7d47**	**-0.91**	**0.21**	**-4.39**	**0.00**	**0.00**	**TRUE**
ffb22bfc8bc47653d64e89eea274109a	0.28	0.22	1.25	0.21	1.00	FALSE
9d082e93ecdbd3a84c42c327b3fafdf7	0.29	0.19	1.51	0.13	1.00	FALSE
a35382145f53b151ee5e8817fb1b52d7	-0.09	0.21	-0.42	0.67	1.00	FALSE

taxon	lfc_std_age	se_std_age	W_std_age	p_std_age	q_std_age	diff_std_age
fe4f6690aac33269018bc47a9af040a9	-0.18	0.18	-1.01	0.31	1.00	FALSE
9389cc5e24fcb8fd40794cda2f4a20f0	0.08	0.13	0.65	0.52	1.00	FALSE
d0315996449ce0da72db4769499ffc95	0.11	0.12	0.89	0.38	1.00	FALSE
a8b6786cd18ffb7901b88116577271aa	-0.13	0.13	-1.05	0.29	1.00	FALSE
8f1a669676c0d61416d77f22346e2fbb	0.03	0.13	0.23	0.82	1.00	FALSE
e3fa155c1e8877fc644ad253e3fa22d3	-0.17	0.14	-1.18	0.24	1.00	FALSE
ed9ce2c8f767dca8fff4de6517a9ecd3	0.11	0.14	0.75	0.45	1.00	FALSE
aaa2143e01a4db36e72d91542511a1f8	0.13	0.14	0.91	0.36	1.00	FALSE
aa60af114c31ddf84a911ca6c07f9504	0.12	0.24	0.49	0.62	1.00	FALSE
818168954d374144da5fd036a0db976b	0.58	0.21	2.73	0.01	0.82	FALSE
3965162f4f4ad6821a06a2db830e7798	0.10	0.16	0.64	0.52	1.00	FALSE
c4fb2d3d6543341e8eed5871e028bf5a	0.10	0.14	0.68	0.49	1.00	FALSE
986daa9057630c895fa5f9c724578811	-0.37	0.16	-2.31	0.02	1.00	FALSE
429528823edb55fa5e24e1f20b322a57	0.18	0.13	1.37	0.17	1.00	FALSE
e922586081ebfd3c1131765980752500	-0.21	0.16	-1.37	0.17	1.00	FALSE
64e1a83810f5e8b23458a6f4b5f7631a	-0.19	0.15	-1.24	0.22	1.00	FALSE
d52dcbc1c0b35a049d6d36a60058c7a2	-0.54	0.18	-2.98	0.00	0.38	FALSE
e0cd2b466ebcfee038f05d27ad4819e4	-0.50	0.20	-2.52	0.01	1.00	FALSE
8a5b42547e126c079f0fc3fb28ba7017	-0.64	0.21	-3.05	0.00	0.31	FALSE
e4029522eb431d33da5ec11ce7201d76	0.07	0.18	0.40	0.69	1.00	FALSE
27fab6bee595d199a1d442f5056cb73d	0.01	0.14	0.04	0.97	1.00	FALSE
4d031dcebe39427cf5ce0610c37486af	-0.03	0.16	-0.18	0.86	1.00	FALSE
3d1cb42e515a27ab9d45615c90fcccd0	0.07	0.14	0.54	0.59	1.00	FALSE
933741b049f15ce5873d1af45d5d5e76	-0.10	0.19	-0.54	0.59	1.00	FALSE
de33bca81e3fbf518db3a2a54cd1f670	0.01	0.15	0.06	0.95	1.00	FALSE
04a33fe4662d8127c50d7680fd86a46f	-0.01	0.17	-0.09	0.93	1.00	FALSE
c3c6b35a7e7fd0a56aab3c29bd47737b	0.15	0.21	0.72	0.47	1.00	FALSE
18af7b7f2b61429936fcd63a453fcefd	-0.04	0.20	-0.22	0.83	1.00	FALSE
3b5b70e9dc7a857f2418795334569e17	-0.05	0.17	-0.29	0.77	1.00	FALSE
8e3955d4651137b0e17e8b2ef44f350f	0.06	0.12	0.48	0.63	1.00	FALSE
a2397abbce9d4a1bbf9d45e8c61edbf3	-0.03	0.23	-0.13	0.90	1.00	FALSE
2df7c666a73180820243b056ffb9410c	-0.27	0.17	-1.57	0.12	1.00	FALSE
d74690bce352c8b27257ffa241a3195f	-0.29	0.17	-1.74	0.08	1.00	FALSE
db6059a25d4ae6b88b9cdaaee1755364	-0.26	0.15	-1.70	0.09	1.00	FALSE
28e35c19f6ecc799b2850bd7cd87f040	-0.52	0.19	-2.74	0.01	0.80	FALSE
b2fba622f1768d4de9fe5a1b3049dbdc	-0.28	0.17	-1.65	0.10	1.00	FALSE
2fc6278cc2ae17ba5c33696317ab41e3	-0.64	0.19	-3.40	0.00	0.09	FALSE
af9aed73a4594c97439b5fe134124eaa	-0.19	0.14	-1.32	0.19	1.00	FALSE
c6af88842367b82c02ad03bce6f0dc4d	-0.22	0.15	-1.50	0.13	1.00	FALSE
59b76cca1802de6de750a71d6efa539b	0.08	0.16	0.51	0.61	1.00	FALSE

taxon	lfc_std_age	se_std_age	W_std_age	p_std_age	q_std_age	diff_std_age
82c6e0b45313500067ade65513677e25	0.11	0.18	0.59	0.55	1.00	FALSE
2ef382de7f184f5f0de0d4c6671ade0c	0.24	0.16	1.54	0.12	1.00	FALSE
726ba575fcaf24a99c7c60dabb79467b	-0.10	0.15	-0.65	0.51	1.00	FALSE
53701e36ae5f9b24a4987eeccd3f339c	-0.10	0.15	-0.68	0.50	1.00	FALSE
c188f2cd65cb93dda1d97a43214082a4	0.06	0.14	0.43	0.67	1.00	FALSE
0cf002cdeee557701d01a4cf7ab3b6c4	-0.17	0.19	-0.93	0.35	1.00	FALSE
104a667d2c05316173b49b6efebd04c4	-0.01	0.12	-0.09	0.93	1.00	FALSE
665a6d094e3f79306959fc3c71de4d7c	0.58	0.19	2.99	0.00	0.38	FALSE
b91de9dc64b0cad22a1a742a4e977b8b	-0.13	0.17	-0.73	0.46	1.00	FALSE
30b45cd5d162409d648eeec5565994f2	0.17	0.18	0.90	0.37	1.00	FALSE
0c643396b2fee14edbe6c92c02f3b652	0.20	0.18	1.16	0.25	1.00	FALSE
169ac541300fa14716452c712a804d95	-0.05	0.17	-0.29	0.77	1.00	FALSE
8bcfcb7500d1809304711c60e85fb77b	0.57	0.18	3.09	0.00	0.28	FALSE
a6e89b000e29b74daac65af2d4fa8c40	-0.19	0.13	-1.44	0.15	1.00	FALSE
d3c15b4e05f175b66cb625a5070c0912	-0.16	0.14	-1.16	0.24	1.00	FALSE
c752ea1974df441eb9d13dc8a17cc986	-0.19	0.15	-1.25	0.21	1.00	FALSE
4e269cbdd41f328dabf501e891c0c3ce	-0.08	0.16	-0.48	0.63	1.00	FALSE
a1fd41728079b9a502437e42327f0ea4	-0.08	0.14	-0.53	0.59	1.00	FALSE
667840ea3c1028901d9bb696a5908215	-0.09	0.14	-0.63	0.53	1.00	FALSE
776f39c6ffbc1df0a88680a9d841ddf1	-0.08	0.15	-0.53	0.59	1.00	FALSE
7cd9cb3dbb38f6ac4fd84cce9304a057	-0.14	0.22	-0.64	0.52	1.00	FALSE
1a911d47249569b7e8164849ee8a7828	-0.02	0.13	-0.16	0.87	1.00	FALSE
a1a8d7f8950a58b57f5a9349809094c2	-0.08	0.13	-0.64	0.52	1.00	FALSE
ffd49d08dcde4adc1dc64e14f6d014a5	-0.01	0.14	-0.04	0.97	1.00	FALSE
42fa8f52b5c1c4a110469218cd2ca8d6	0.06	0.19	0.31	0.76	1.00	FALSE
f2a7d05134f1bfccf0ca786b9c8b2c58	-0.07	0.14	-0.52	0.60	1.00	FALSE
db99ba353ea8f395feb76013b4855445	0.21	0.22	0.97	0.33	1.00	FALSE
7e0d656b5371d9c447f5ba0a6589cf5c	-0.20	0.18	-1.12	0.26	1.00	FALSE
d443ad925116af4a55f7a48c91eb8d05	-0.01	0.13	-0.10	0.92	1.00	FALSE
82cbfddad510359d13346b8fa16e5a91	0.15	0.21	0.71	0.48	1.00	FALSE
3f8f174448b386f58fc09cb6bc3151f3	-0.32	0.21	-1.51	0.13	1.00	FALSE
6e9c989a894b939f105838c8f3445104	-0.00	0.13	-0.02	0.98	1.00	FALSE
6861b32a4e186d13e250ca8013b81200	-0.06	0.17	-0.32	0.75	1.00	FALSE
ebb881e05aedb9f1d522aba44aabf072	-0.11	0.21	-0.52	0.60	1.00	FALSE
fe71e0bb0d7b6f30c2a33bcbbbaa41fd	0.15	0.15	1.05	0.29	1.00	FALSE
95e33362e84f4b23ad351929ca0c92fc	-0.29	0.21	-1.41	0.16	1.00	FALSE
17595d01170169e5ffc2d7e1bcfaa840	-0.18	0.16	-1.11	0.27	1.00	FALSE
1a98ff4f7e42b825bd257e038465432f	-0.03	0.16	-0.21	0.84	1.00	FALSE
d3300abd8f0d5934b5a53edde25f15c1	-0.01	0.14	-0.04	0.97	1.00	FALSE
a90fe630a0792bbfa2063b6d90190bac	-0.10	0.17	-0.58	0.56	1.00	FALSE

taxon	lfc_std_age	se_std_age	W_std_age	p_std_age	q_std_age	diff_std_age
63ee4320a568cbe73cef58ad2313fc31	-0.28	0.14	-2.05	0.04	1.00	FALSE
a3e8fe6bf4c29c95a9a07a4ed3fa3835	-0.04	0.13	-0.30	0.76	1.00	FALSE
fbe681bb9d21244b516cdfdb7f230b69	-0.27	0.16	-1.71	0.09	1.00	FALSE
5844b28b946965e1307531118791a69c	-0.04	0.17	-0.23	0.82	1.00	FALSE
5ba4fcb429b90dd97bd16830b246e048	-0.15	0.12	-1.31	0.19	1.00	FALSE
614ed52e6ffa758afc0d07d6fef8a830	-0.03	0.14	-0.18	0.85	1.00	FALSE
f7958f380e6bd72231e3d9df973e554b	0.06	0.14	0.38	0.70	1.00	FALSE
dc4d31ce51866ecfe8802ed6d716364a	-0.14	0.13	-1.07	0.29	1.00	FALSE
9783f65ac7c7175949c4fd41841e2214	-0.07	0.16	-0.47	0.64	1.00	FALSE
3cbb35bbce6f506113e5bd7e9800c4e8	0.00	0.14	0.02	0.99	1.00	FALSE
af15c97a8bbfe7ecb05af4baca1ac2fc	-0.03	0.13	-0.20	0.84	1.00	FALSE
998bcad9b75de667179dd567e029bbf9	0.02	0.14	0.17	0.87	1.00	FALSE
dee9b9994fb3e29b8dbb5df07d458f4b	-0.15	0.15	-1.03	0.30	1.00	FALSE
0aa415e2ba6a661bead2121028d9370a	0.06	0.13	0.47	0.64	1.00	FALSE
1e4445d0ec82478bbdad2c87cb4107b1	0.05	0.13	0.39	0.70	1.00	FALSE
801501015c0e5ff39e9dcbdd0d05b335	-0.42	0.18	-2.30	0.02	1.00	FALSE
554eb7bf7e5c55ad4a01f64a140edc99	-0.45	0.18	-2.54	0.01	1.00	FALSE
b84d4d4e10230419d47a1515b753dcf1	-0.04	0.13	-0.34	0.73	1.00	FALSE
2800bb10e27d18faa1115fa96aa35f9a	0.01	0.14	0.09	0.93	1.00	FALSE
ffa8408dd9bb380efbec99d5358dd971	-0.13	0.13	-0.99	0.32	1.00	FALSE
2d8094bb1d62362e4d86513d5411c32f	0.01	0.14	0.07	0.94	1.00	FALSE
61a3662d515e4b5abf9a30fe478fc2f3	-0.47	0.15	-3.03	0.00	0.33	FALSE
e62ff1bb2b1213ee296b46a4b6da9511	-0.26	0.18	-1.43	0.15	1.00	FALSE
86bad06167ece78433a16023f189940f	-0.22	0.13	-1.71	0.09	1.00	FALSE
4991b491dc839d74db77914343d5ef9e	-0.48	0.16	-3.03	0.00	0.33	FALSE
8e1feeb7e21c535f823589c5a72560b8	0.09	0.17	0.55	0.59	1.00	FALSE
d5c3ebe1364906df8ab61ffb29307e3d	-0.05	0.13	-0.37	0.71	1.00	FALSE
67b8f4dbe4b7e9c44500abd2faf351d1	-0.08	0.12	-0.64	0.52	1.00	FALSE
1f4663a1817c149865942c39cea67f42	0.78	0.23	3.45	0.00	0.08	FALSE
fa4158cdcb8a68c5df337cd0fc25dd1a	-0.01	0.15	-0.08	0.94	1.00	FALSE
492c9d99645d6866290affabd62e6c23	0.18	0.16	1.15	0.25	1.00	FALSE
5ab210b70bbb9d11943b629b9c1adea3	0.05	0.13	0.40	0.69	1.00	FALSE
1d1def08494fecb247f799c76fb29750	0.07	0.24	0.29	0.77	1.00	FALSE
abe3c653201e0f86f2d5c49f7ed32f75	-0.02	0.16	-0.10	0.92	1.00	FALSE
24e196c950338dc0dd38a9b38c84c1d6	0.07	0.14	0.51	0.61	1.00	FALSE
829ee0e126800d181ab4a09065174a4d	0.37	0.17	2.14	0.03	1.00	FALSE
5bfb87b61ec26b3347297c36d287f034	0.01	0.17	0.03	0.97	1.00	FALSE
c8fba175a590937b39493057330cd196	0.12	0.15	0.78	0.44	1.00	FALSE
b2fcbf2fdc0719fa654ba1c98fc0ce23	0.22	0.14	1.59	0.11	1.00	FALSE
67696afee40020e8783692136dc56cf9	0.01	0.14	0.09	0.93	1.00	FALSE

taxon	lfc_std_age	se_std_age	W_std_age	p_std_age	q_std_age	diff_std_age
00ee634ba50681f351bdeb78fa3dca36	-0.01	0.19	-0.07	0.95	1.00	FALSE
4c1b442d388d6489de50ac22de1e4c5d	-0.09	0.14	-0.65	0.52	1.00	FALSE
ca96c5417115dda3c2686fb42e695457	-0.02	0.18	-0.13	0.89	1.00	FALSE
2ed0216a8320b28d71f1f788daff92ab	-0.10	0.17	-0.55	0.58	1.00	FALSE
f36c161e4341ca7c0fd5d7615f736706	-0.16	0.17	-0.92	0.36	1.00	FALSE
393b4e817eebce91c46321c143a0d06d	0.10	0.16	0.63	0.53	1.00	FALSE
91c93208099c5254153fe250684deda0	-0.27	0.16	-1.73	0.08	1.00	FALSE
f95cd34b5bc49283f5af83b430341688	0.03	0.16	0.17	0.86	1.00	FALSE
713e55690cb976f5af2147acc45b8b39	-0.65	0.19	-3.41	0.00	0.09	FALSE
aa4de042e0c492a5f41355fe54ca2246	0.16	0.17	0.97	0.33	1.00	FALSE
9b88028c6a724b353607573926edbf82	0.05	0.14	0.37	0.71	1.00	FALSE
0ff04f60a629ab032309e3ec3162dfab	-0.02	0.16	-0.14	0.89	1.00	FALSE
c39f99ccf879cb1859aeb5d9e8b42cf8	-0.04	0.15	-0.30	0.76	1.00	FALSE
f93fbfab216a53cd11cdb9fd27765df4	-0.00	0.18	-0.02	0.98	1.00	FALSE
9868a3670f730be89cb79f7dfe9eb914	0.05	0.13	0.36	0.72	1.00	FALSE
9286c457c28a8bf4f2949a3c376a4399	-0.35	0.19	-1.84	0.07	1.00	FALSE
cc523fdc023fc1e056705eb8b1e69f56	-0.03	0.15	-0.18	0.86	1.00	FALSE
86f88dec7777f0258548b6bdb3ea53c9	-0.04	0.14	-0.27	0.79	1.00	FALSE

Figure S1. 16S rRNA rarefaction curves

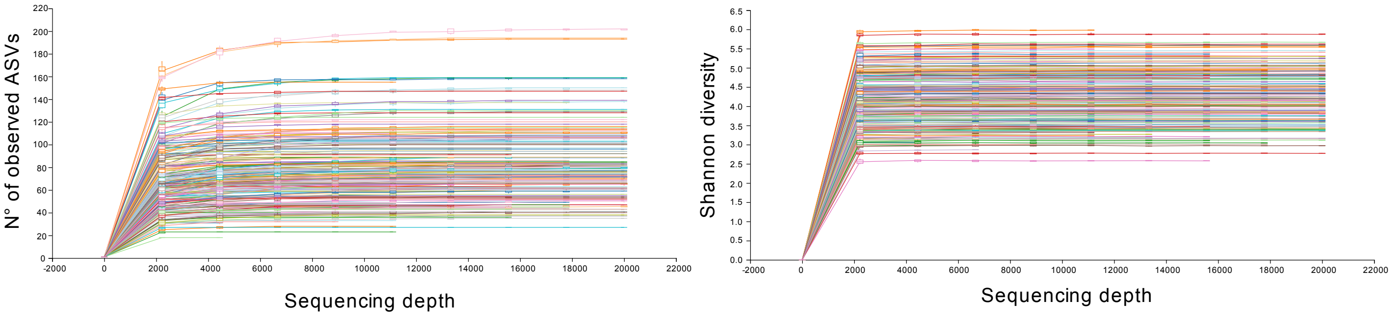

Rarefaction curves for two alpha diversity metrics: N° of observed ASVs and Shannon diversity. Diversity saturated in all samples at ~3000 reads per sample.

Figure S2. 28S rRNA rarefaction curves (sampling depth 20000)

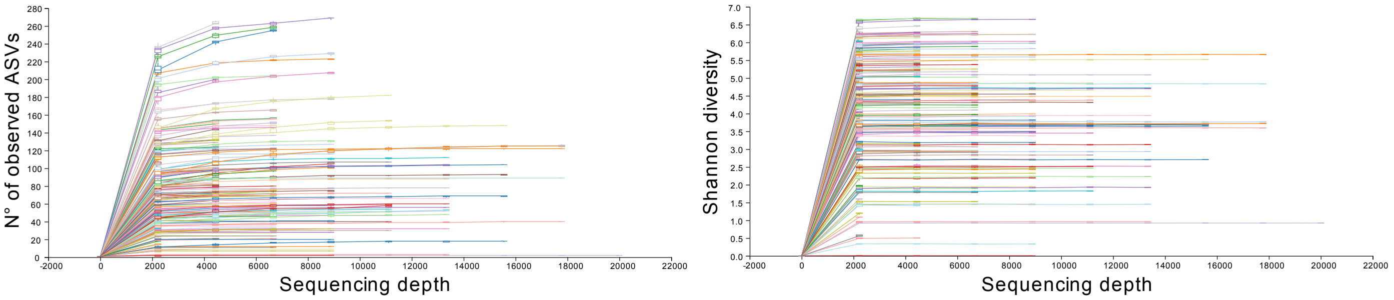

Figure S3. 28S rRNA rarefaction curves (sampling depth 2000)

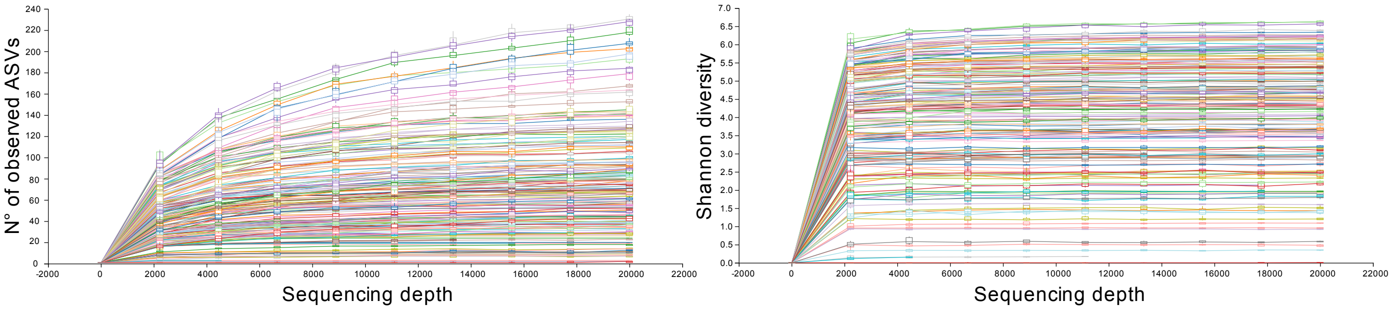

Rarefaction curves for two alpha diversity metrics: N° of observed ASVs and Shannon diversity. Diversity saturated in all samples at ~2000 reads per sample.
