# Supplementary material for: Early-life factors shaping the gut microbiota of Common buzzard nestlings
Source: Anim Microbiome. 2024 May 14;6:27. doi: 10.1186/s42523-024-00313-8 (PMC11092241; doi:10.1186/s42523-024-00313-8)
Supplement: Supplementary file 2 — Supplementary file1 (PDF 24 kb) [file 42523_2024_313_MOESM2_ESM.pdf]

# Phenol/Chloroform Extraction Protocol (Cloacal Swabs)

## Swabs in RNAlater initial lysis :

1. Vortex 30s. Centrifuge for 10 min at 13 000 rpm.
2. Remove the RNAlater (do not remove the swab).
3. Transfer the swab to a new 2 ml tube (optional).
4. Re-suspend the pellets and swab in 600/700 µl extraction buffer
5. Incubate for at 65°C for 10 min (in water-bath shaking on).
6. Bead beating for 15 min at max speed.
7. Add 20 µl Proteinase K (10 mg/ml).
8. Digest for 1/2h at 56°C.

## Phenol/Chloroform Extraction

1. a. Vortex for 5-10s;

   b. Add 250µl NaCl (5M) vortex;

   c. Add 700µl Chloroform-Isoamyl alcohol (24:1) and shake vigorously by hand.

   d. Centrifuge for 15 min at 13 000 rpm.

2. a. Transfer the clear (upper) phase into a new 1.5 ml Eppendorf tube (usually about 500µl);

   b. Add 1/10 volume of NaAc (50µl), mix well by shaking vigorously

   c. Add 0.6-0.7x volume of Isopropanol (350-390µl), mix well by shaking vigorously.

   d. Centrifuge for 20 min at 13 00 rpm.

3. a. Discard the supernatant (pay attention to the pellet);

   b. Add 500µl of EtOH 70%;

   c. Leave at room temperature for 10 min;

   d. Centrifuge for 10 min at 13 000 rpm

   e. Repeat this for 2-3 times.

4. Discard the supernatant (pay attention to the pellet). If there is any ethanol remaining, carefully remove it with a pipette (if the pellet is loose, remove all the ethanol with a pipette). Let the pellet air dry for aprox. 1-2h with the lid open. Or for 1h at 56°C

5. Add 70-80µl of Elution Buffer (TE). Elute the DNA overnight at room temperature or for 1h at 40°C.
