# Supplementary material for: Early-life factors shaping the gut microbiota of Common buzzard nestlings
Source: Anim Microbiome. 2024 May 14;6:27. doi: 10.1186/s42523-024-00313-8 (PMC11092241; doi:10.1186/s42523-024-00313-8)
Supplement: Supplementary file 3 — Supplementary file1 (PDF 257 kb) [file 42523_2024_313_MOESM3_ESM.pdf]

# Quality control - community standards workflow

## Table of Contents


**Quality control - community standards workflow**
- Table of Contents

**A) 16S rRNA workflow**
- 1. Activate Qiime2
- 2. Import expected taxonomic composition of mock samples
- 2. Get the observed taxonomic composition of mock samples
  - 2.1 Import results after decontam pipeline
  - 2.2 Subset community standard samples (positive controls)
  - 2.3 Go through the filtering steps
  - 2.4 Inspect the taxonomic composition of the community standards (taxa bar plots only for positive controls)
- 3. Agglomerate taxa at species level
  - 3.1 Convert sequence counts into relative abundances
- 4. Compare observed and expected taxonomic composition of mock samples
- 5. Feature evaluation results
  - 5.1 Per-level accuracy
  - 5.2 False positives: misclassifications
  - 5.3 False positives: underclassifications
  - 5.4 False negatives

**B) 28S rRNA workflow**
- 1. Activate Qiime2
- 2. Import expected taxonomic composition of mock samples
- 2. Get the observed taxonomic composition of mock samples
  - 2.1 Import results after decontam pipeline
  - 2.2 Subset community standard samples (positive controls)
  - 2.3 Go through the filtering steps
  - 2.4 Inspect the taxonomic composition of the community standards (taxa bar plots only for positive controls)
- 3. Agglomerate taxa at species level
  - 3.1 Convert sequence counts into relative abundances
- 4. Compare observed and expected taxonomic composition of mock samples
- 5. Feature evaluation results
  - 5.1 Per-level accuracy
  - 5.2 False positives: misclassifications
  - 5.3 False positives: underclassifications
  - 5.4 False negatives


---

# A) 16S rRNA workflow

## 1. Activate Qiime2

```
# Activate base env
. ~/.bashrc

# Activate qiime2
conda activate qiime2-2022.11
```

## 2. Import expected taxonomic composition of mock samples

Composition of comunity standard available here: [https://www.zymoresearch.de/collections/zymobiomics-microbial-community-standards/products/zymobiomics-microbial-community-standard](https://www.zymoresearch.de/collections/zymobiomics-microbial-community-standards/products/zymobiomics-microbial-community-standard)

For the purpose of this analysis, eukaryote taxa were excluded from the community standards' composition.

```
biom convert -i mock_expected.tsv -o mock-expected.biom --table-type="OTU table" --to-hdf5

qiime tools import --input-path mock-expected.biom --type 'FeatureTable[RelativeFrequency]' --input-format
BIOMV210Format --output-path mock-expected.qza
```

## 2. Get the observed taxonomic composition of mock samples

### 2.1 Import results after decontam pipeline

```
qiime tools import --input-path table-nocontam.biom --type 'FeatureTable[Frequency]' --input-format
BIOMV100Format --output-path table-nocontam.qza
```

### 2.2 Subset community standard samples (positive controls)

```
qiime feature-table filter-samples --i-table table-nocontam.qza --m-metadata-file positive-samples.tsv --o-
filtered-table mock-observed.qza
```

### 2.3 Go through the filtering steps

```
#Filter taxonomy
qiime taxa filter-table --i-table mock-observed.qza --i-taxonomy taxonomy.qza --p-exclude
mitochondria,chloroplast,Unassigned,Vertebrata --p-include p_ --o-filtered-table mock-observed.qza

# Filter features present in less than 2 samples
qiime feature-table filter-features --i-table mock-observed.qza --p-min-samples 2 --o-filtered-table mock-
observed.qza
qiime feature-table summarize --i-table mock-observed.qza --o-visualization mock-observed.qzv --m-sample-
metadata-file buzzard_meta.tsv

# Filter samples with less than 500 reads
qiime feature-table filter-samples --i-table mock-observed.qza --p-min-frequency 500 --o-filtered-table mock-
observed.qza
```

### 2.4 Inspect the taxonomic composition of the community standards (taxa bar plots only for positive controls)

```
qiime taxa barplot --i-table mock-observed.qza --i-taxonomy taxonomy.qza --m-metadata-file buzzard_meta.tsv --o-visualization mock-observed-bar-plot.qzv
```

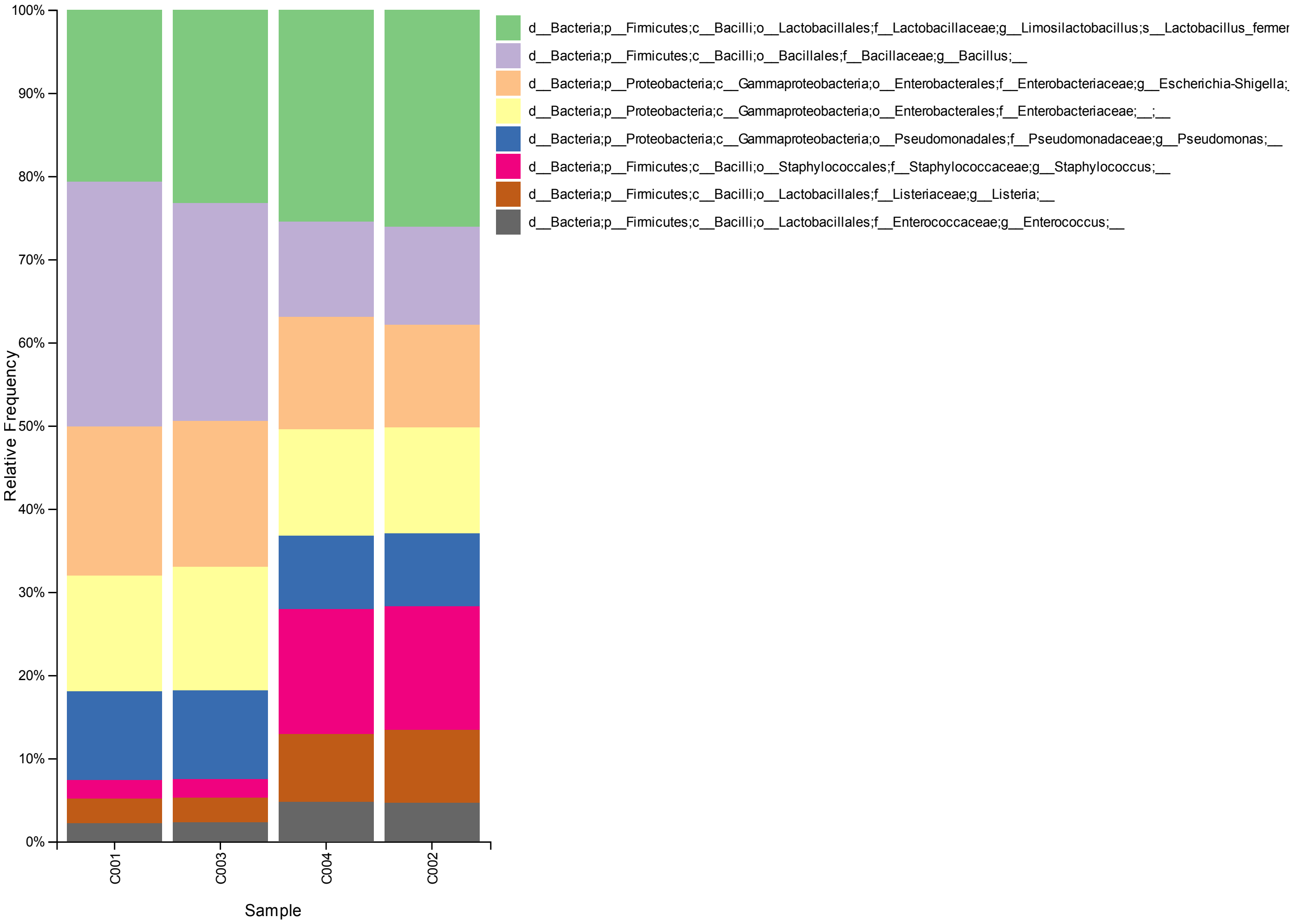


**C001 and C003** are replicates of the ZymoBIOMICS Microbial Community Standard (D6300) used for validation of the extraction method.

**C002 and C004** are replicates of the ZymoBIOMICS Microbial Community Standard (D6305) used for validation of the PCR procedure.

## 3. Agglomerate taxa at species level

```
qiime taxa collapse --i-table mock-observed.qza --i-taxonomy taxonomy.qza --p-level 7 --o-collapsed-table mock-observed-l7.qza
```

### 3.1 Convert sequence counts into relative abundances

```
qiime feature-table relative-frequency --i-table mock-observed-l7.qza --o-relative-frequency-table mock-observed-l7-rel.qza
```

## 4. Compare observed and expected taxonomic composition of mock samples

```
qiime quality-control evaluate-composition --i-expected-features mock-expected.qza --i-observed-features mock-observed-l7-rel.qza --o-visualization mock-comparison.qzv
```

## 5. Feature evaluation results

Taxon accuracy rate (**TAR**) and taxon detection rate (**TDR**) are used for qualitative compositional analyses of mock communities.

At a given taxonomic level, a classification is a:

**True positive (TP)**, if that taxon is both observed and expected.

**False positive (FP)**, if that taxon is observed but not expected.

**False negative (FN)**, if a taxon is expected but not observed.

These are used to calculate TAR and TDR as:

**TAR = TP/(TP + FP)** Fraction of observed taxa that were expected at level L.

**TDR = TP/(TP + FN)** Fraction of expected taxa that are observed at level L.

### 5.1 Per-level accuracy

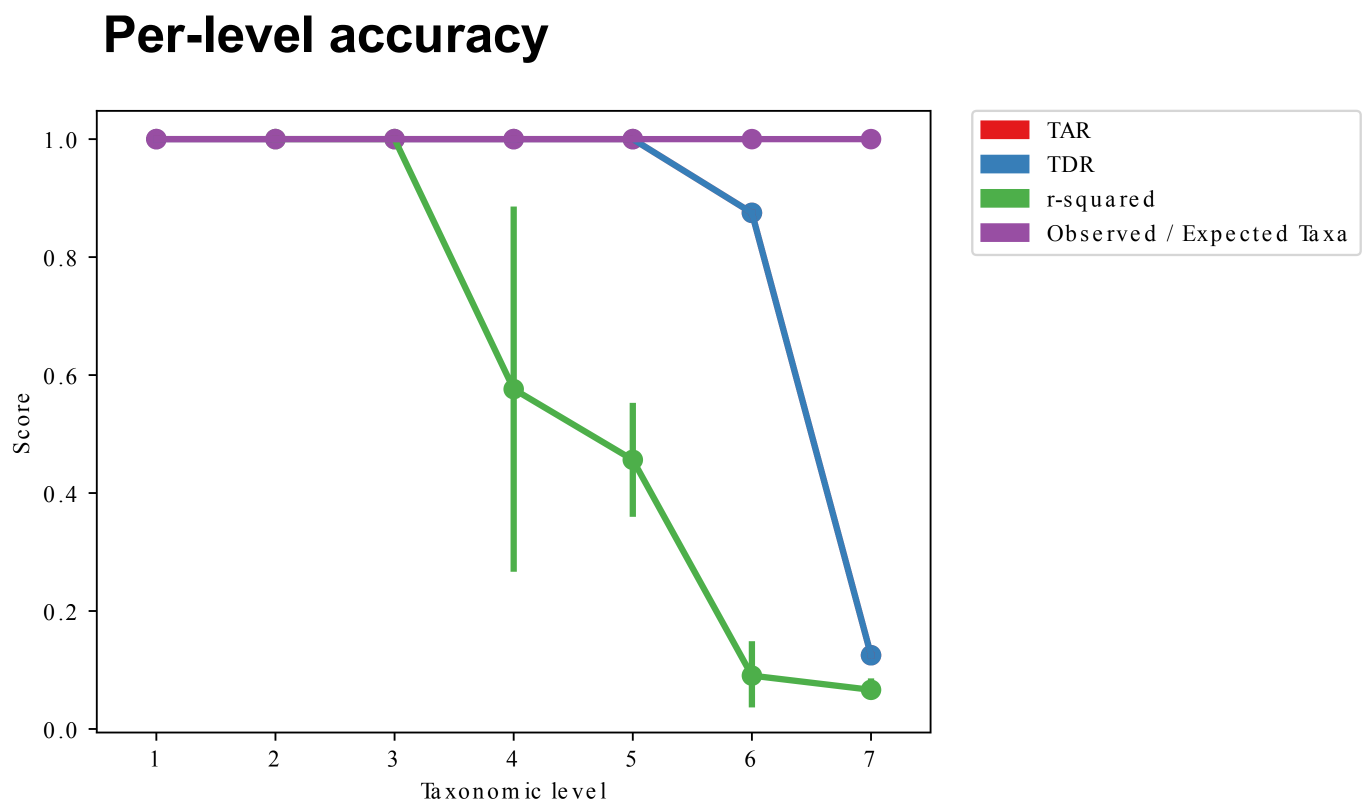


| sample | level | Observed Taxa | Observed / Expected Taxa | TAR | TDR | Slope | Intercept | r-value | P value | Std Err | Bray-Curtis | Jaccard | r-squared |
|---|---|---|---|---|---|---|---|---|---|---|---|---|---|
| C001 | 1 | 1 | 1 | 1 | 1 | | | | | | 0 | 0 | |
| C002 | 1 | 1 | 1 | 1 | 1 | | | | | | 0 | 0 | |

| sample | level | Observed Taxa | Observed / Expected Taxa | TAR | TDR | Slope | Intercept | r-value | P value | Std Err | Bray-Curtis | Jaccard | r-squared |
|---|---|---|---|---|---|---|---|---|---|---|---|---|---|
| C003 | 1 | 1 | 1 | 1 | 1 | | | | | | 0 | 0 | |
| C004 | 1 | 1 | 1 | 1 | 1 | | | | | | 0 | 0 | |
| C001 | 2 | 2 | 1 | 1 | 1 | 0.296724388 | 0.351637806 | 1 | 0 | 0 | 0.17792873 | 0 | 1 |
| C002 | 2 | 2 | 1 | 1 | 1 | 0.63862169 | 0.180689155 | 1 | 0 | 0 | 0.091428712 | 0 | 1 |
| C003 | 2 | 2 | 1 | 1 | 1 | 0.273625725 | 0.363187137 | 1 | 0 | 0 | 0.183772691 | 0 | 1 |
| C004 | 2 | 2 | 1 | 1 | 1 | 0.586240686 | 0.206879657 | 1 | 0 | 0 | 0.104681106 | 0 | 1 |
| C001 | 3 | 2 | 1 | 1 | 1 | 0.296724388 | 0.351637806 | 1 | 0 | 0 | 0.17792873 | 0 | 1 |
| C002 | 3 | 2 | 1 | 1 | 1 | 0.63862169 | 0.180689155 | 1 | 0 | 0 | 0.091428712 | 0 | 1 |
| C003 | 3 | 2 | 1 | 1 | 1 | 0.273625725 | 0.363187137 | 1 | 0 | 0 | 0.183772691 | 0 | 1 |
| C004 | 3 | 2 | 1 | 1 | 1 | 0.586240686 | 0.206879657 | 1 | 0 | 0 | 0.104681106 | 0 | 1 |
| C001 | 4 | 5 | 1 | 1 | 1 | 0.436888805 | 0.112622239 | 0.472250039 | 0.42187708 | 0.47080729 | 0.298227114 | 0 | 0.223020099 |
| C002 | 4 | 5 | 1 | 1 | 1 | 0.851634012 | 0.029673198 | 0.946941671 | 0.014553829 | 0.166886957 | 0.091428712 | 0 | 0.896698529 |
| C003 | 4 | 5 | 1 | 1 | 1 | 0.524533008 | 0.095093398 | 0.566336521 | 0.319571943 | 0.440713738 | 0.271647996 | 0 | 0.320737055 |
| C004 | 4 | 5 | 1 | 1 | 1 | 0.818767538 | 0.036246492 | 0.929546722 | 0.022209666 | 0.18750248 | 0.104681106 | 0 | 0.864057109 |
| C001 | 5 | 7 | 1 | 1 | 1 | 1.382371895 | -0.054624556 | 0.595664688 | 0.158168445 | 0.8336422 | 0.321227431 | 0 | 0.354816421 |
| C002 | 5 | 7 | 1 | 1 | 1 | 1.10912044 | -0.015588634 | 0.74306485 | 0.055649207 | 0.446720129 | 0.168630585 | 0 | 0.552145372 |
| C003 | 5 | 7 | 1 | 1 | 1 | 1.400266294 | -0.057180899 | 0.61258048 | 0.143636351 | 0.808004777 | 0.320355228 | 0 | 0.375254844 |
| C004 | 5 | 7 | 1 | 1 | 1 | 1.127369673 | -0.018195668 | 0.736864011 | 0.058852967 | 0.462558891 | 0.175629044 | 0 | 0.542968571 |
| C001 | 6 | 8 | 1 | 0.875 | 0.875 | 0.358466929 | 0.071281452 | 0.213689608 | 0.580901864 | 0.61939472 | 0.425227431 | 0.222222222 | 0.045663248 |
| C002 | 6 | 8 | 1 | 0.875 | 0.875 | 0.459148545 | 0.060094606 | 0.384600701 | 0.306762438 | 0.416519073 | 0.272630585 | 0.222222222 | 0.147917699 |
| C003 | 6 | 8 | 1 | 0.875 | 0.875 | 0.316897871 | 0.075900237 | 0.195233044 | 0.61469188 | 0.601697684 | 0.424355228 | 0.222222222 | 0.038115941 |
| C004 | 6 | 8 | 1 | 0.875 | 0.875 | 0.424765106 | 0.063914988 | 0.360729945 | 0.340222387 | 0.415093343 | 0.279629044 | 0.222222222 | 0.130126093 |
| C001 | 7 | 8 | 1 | 0.125 | 0.125 | -0.386441112 | 0.092429407 | -0.294330054 | 0.286939845 | 0.348016925 | 0.816 | 0.933333333 | 0.086630181 |
| C002 | 7 | 8 | 1 | 0.125 | 0.125 | -0.251584632 | 0.083438975 | -0.232622168 | 0.404102 | 0.291729901 | 0.816 | 0.933333333 | 0.054113073 |
| C003 | 7 | 8 | 1 | 0.125 | 0.125 | -0.322480879 | 0.088165392 | -0.250877702 | 0.3671117 | 0.345107199 | 0.816 | 0.933333333 | 0.062939621 |
| C004 | 7 | 8 | 1 | 0.125 | 0.125 | -0.26714477 | 0.084476318 | -0.248655979 | 0.371511131 | 0.28861368 | 0.816 | 0.933333333 | 0.061829796 |

# Linear regression between observed and expected abundances

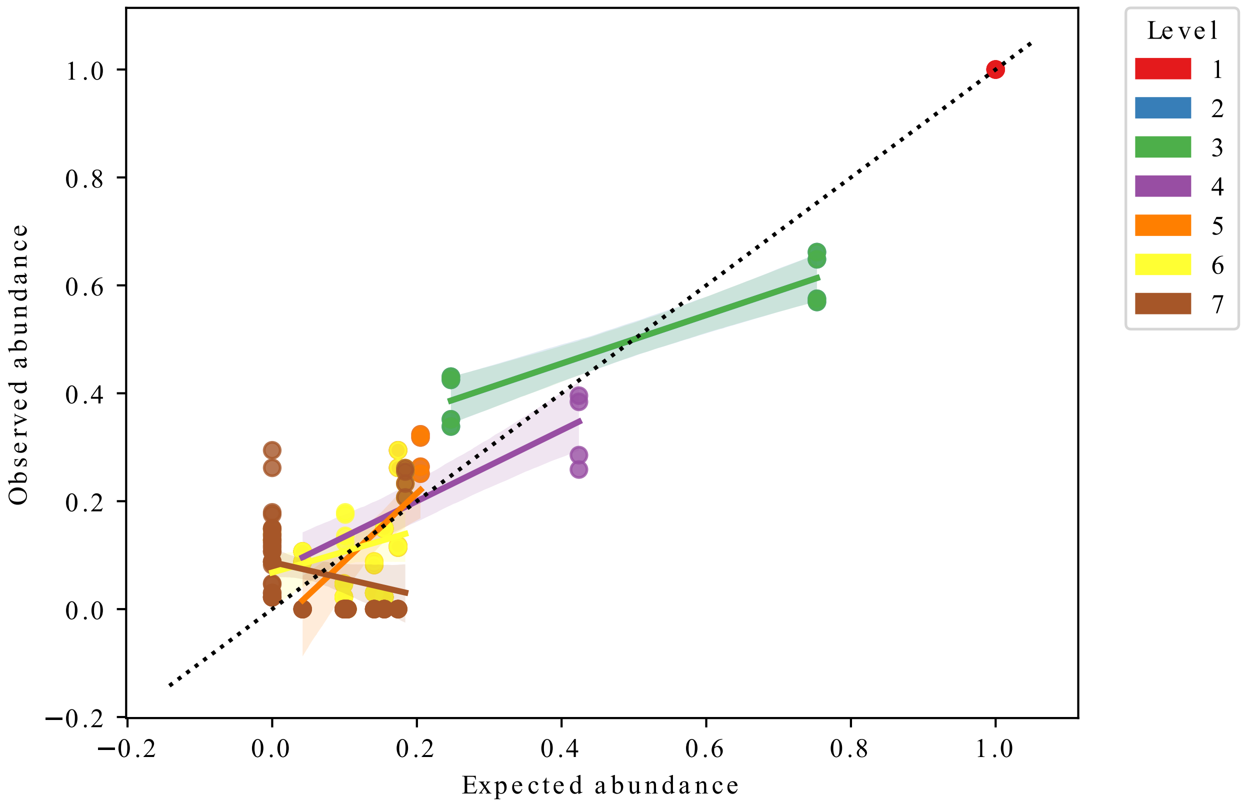


## 5.2 False positives: misclassifications

No misclassifications detected.

## 5.3 False positives: underclassifications

| Taxon | C001 | C002 | C003 | C004 |
|---|---|---|---|---|
| d_Bacteria;p_Firmicutes;c_Bacilli;o_Bacillales;f_Bacillaceae;g_Bacillus;__ | 0.294298385 | 0.117544027 | 0.261875305 | 0.114060885 |
| d_Bacteria;p_Firmicutes;c_Bacilli;o_Lactobacillales;f_Enterococcaceae;g_Enterococcus;__ | 0.02172949 | 0.046049489 | 0.022757095 | 0.04749225 |
| d_Bacteria;p_Firmicutes;c_Bacilli;o_Lactobacillales;f_Listeriaceae;g_Listeria;__ | 0.029933481 | 0.088277443 | 0.029880403 | 0.081670773 |
| d_Bacteria;p_Firmicutes;c_Bacilli;o_Staphylococcales;f_Staphylococcaceae;g_Staphylococcus;__ | 0.022109598 | 0.148498455 | 0.022007273 | 0.150147047 |
| d_Bacteria;p_Proteobacteria;c_Gammaproteobacteria;o_Enterobacterales;f_Enterobacteriaceae;;__ | 0.138866012 | 0.127766632 | 0.148502231 | 0.128089977 |
| d_Bacteria;p_Proteobacteria;c_Gammaproteobacteria;o_Enterobacterales;f_Enterobacteriaceae;g_Escherichia-Shigella;__ | 0.179315806 | 0.123308175 | 0.175345855 | 0.135720531 |
| d_Bacteria;p_Proteobacteria;c_Gammaproteobacteria;o_Pseudomonadales;f_Pseudomonadaceae;gPseudomonas;__ | 0.106746912 | 0.087353906 | 0.106924605 | 0.087870599 |

## 5.4 False negatives

The taxonomy classifier can accurately assign taxonomy only up to the genus level. Since, all community standards are a priory identified at species level, this leads to the classification of every feature as a false negative.

| Taxon | C001 | C002 | C003 | C004 |
|---|---|---|---|---|
| d_Bacteria;p_Firmicutes;c_Bacilli;o_Bacillales;f_Bacillaceae;g_Bacillus;s__Bacillus_subtilis | 0.174 | 0.174 | 0.174 | 0.174 |
| d_Bacteria;p_Firmicutes;c_Bacilli;o_Lactobacillales;f_Enterococcaceae;g_Enterococcus;s__Enterococcus_faecalis | 0.099 | 0.099 | 0.099 | 0.099 |
| d_Bacteria;p_Firmicutes;c_Bacilli;o_Lactobacillales;f_Listeriaceae;g_Listeria;s__Listeria_monocytogenes | 0.141 | 0.141 | 0.141 | 0.141 |
| d_Bacteria;p_Firmicutes;c_Bacilli;o_Staphylococcales;f_Staphylococcaceae;g_Staphylococcus;s__Staphylococcus_aureus | 0.155 | 0.155 | 0.155 | 0.155 |
| d_Bacteria;p_Proteobacteria;c_Gammaproteobacteria;o_Enterobacterales;f_Enterobacteriaceae;g_Escherichia-Shigella;s__Escherichia_coli | 0.101 | 0.101 | 0.101 | 0.101 |
| d_Bacteria;p_Proteobacteria;c_Gammaproteobacteria;o_Enterobacterales;f_Enterobacteriaceae;g_Salmonella;s__Salmonella_enterica | 0.104 | 0.104 | 0.104 | 0.104 |
| d_Bacteria;p_Proteobacteria;c_Gammaproteobacteria;o_Pseudomonadales;f_Pseudomonadaceae;g_Pseudomonas;s__Pseudomonas_aeruginosa | 0.042 | 0.042 | 0.042 | 0.042 |

# B) 28S rRNA workflow

## 1. Activate Qiime2

```
# Activate base env
. ~/.bashrc

# Activate qiime2
conda activate qiime2-2022.11
```

## 2. Import expected taxonomic composition of mock samples

Composition of comunity standard available here: [https://www.zymoresearch.de/collections/zymobiomics-microbial-community-standards/products/zymobiomics-microbial-community-standard](https://www.zymoresearch.de/collections/zymobiomics-microbial-community-standards/products/zymobiomics-microbial-community-standard)

```
biom convert -i mock_expected.tsv -o mock-expected.biom --table-type="OTU table" --to-hdf5

qiime tools import --input-path mock-expected.biom --type 'FeatureTable[RelativeFrequency]' --input-format
BIOMV210Format --output-path mock-expected.qza
```

## 2. Get the observed taxonomic composition of mock samples

### 2.1 Import results after decontam pipeline

```
qiime tools import --input-path table-nocontam.biom --type 'FeatureTable[Frequency]' --input-format
BIOMV100Format --output-path table-nocontam.qza
```

### 2.2 Subset community standard samples (positive controls)

```
qiime feature-table filter-samples --i-table table-nocontam.qza --m-metadata-file positive-samples.tsv --o-
filtered-table mock-observed.qza
```

### 2.3 Go through the filtering steps

```
#Filter taxonomy
qiime taxa filter-table --i-table mock-observed.qza --i-taxonomy taxonomy.qza --p-exclude
mitochondria,chloroplast,Unassigned,Vertebrata --p-include p_ --o-filtered-table mock-observed.qza

# Filter features present in less than 2 samples
qiime feature-table filter-features --i-table mock-observed.qza --p-min-samples 2 --o-filtered-table mock-
observed.qza
qiime feature-table summarize --i-table mock-observed.qza --o-visualization mock-observed.qzv --m-sample-
metadata-file buzzard_meta.tsv

# Filter samples with less than 500 reads
qiime feature-table filter-samples --i-table mock-observed.qza --p-min-frequency 500 --o-filtered-table mock-
observed.qza
```

### 2.4 Inspect the taxonomic composition of the community standards (taxa bar plots only for positive controls)

```
qiime taxa barplot --i-table mock-observed.qza --i-taxonomy taxonomy.qza --m-metadata-file buzzard_meta.tsv --
o-visualization mock-observed-bar-plot.qzv
```

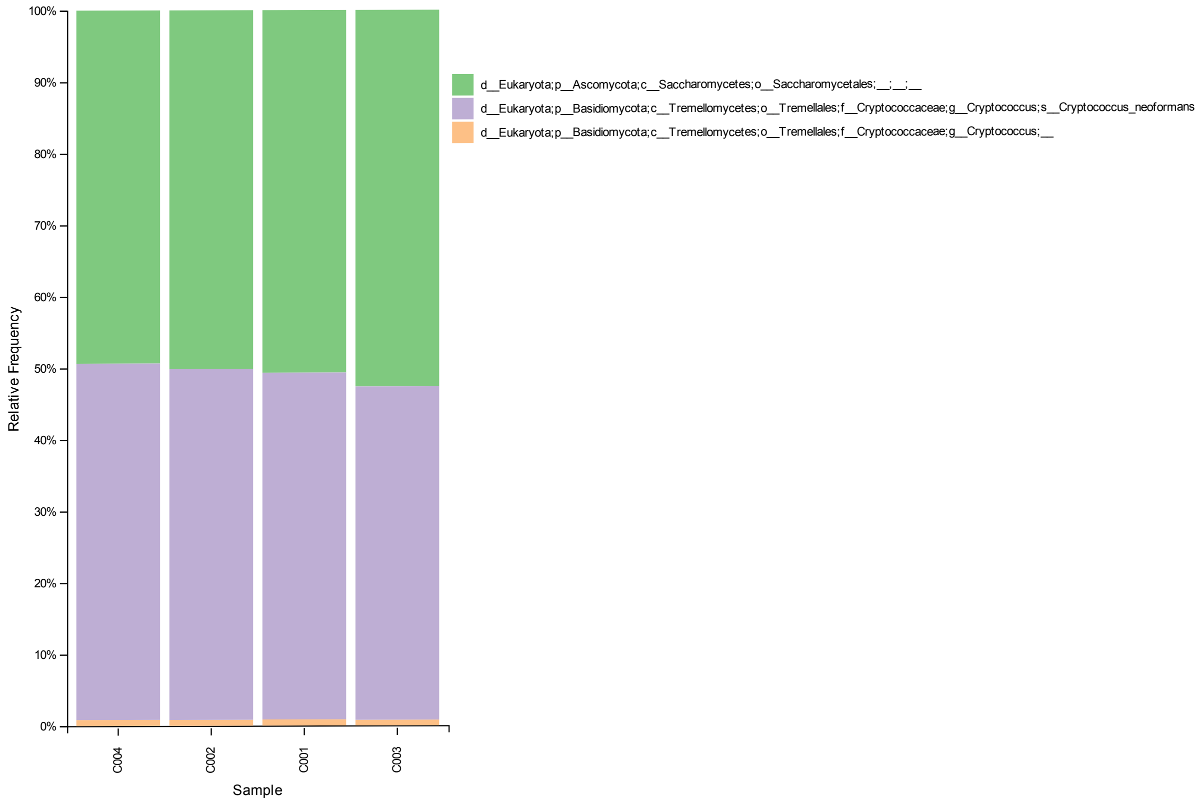


**C001 and C003** are replicates of the ZymoBIOMICS Microbial Community Standard (D6300) used for validation of the extraction method.

**C002 and C004** are replicates of the ZymoBIOMICS Microbial Community Standard (D6305) used for validation of the PCR procedure.

## 3. Agglomerate taxa at species level

```
qiime taxa collapse --i-table mock-observed.qza --i-taxonomy taxonomy.qza --p-level 7 --o-collapsed-table mock-
observed-l7.qza
```

### 3.1 Convert sequence counts into relative abundances

```
qiime feature-table relative-frequency --i-table mock-observed-l7.qza --o-relative-frequency-table mock-observed-l7-rel.qza
```

## 4. Compare observed and expected taxonomic composition of mock samples

```
qiime quality-control evaluate-composition --i-expected-features mock-expected.qza --i-observed-features mock-observed-l7-rel.qza --o-visualization mock-comparison.qzv
```

## 5. Feature evaluation results

Taxon accuracy rate (**TAR**) and taxon detection rate (**TDR**) are used for qualitative compositional analyses of mock communities.

At a given taxonomic level, a classification is a:

**True positive (TP)**, if that taxon is both observed and expected.

**False positive (FP)**, if that taxon is observed but not expected.

**False negative (FN)**, if a taxon is expected but not observed.

These are used to calculate TAR and TDR as:

**TAR = TP/(TP + FP)** Fraction of observed taxa that were expected at level L.

**TDR = TP/(TP + FN)** Fraction of expected taxa that are observed at level L.

### 5.1 Per-level accuracy

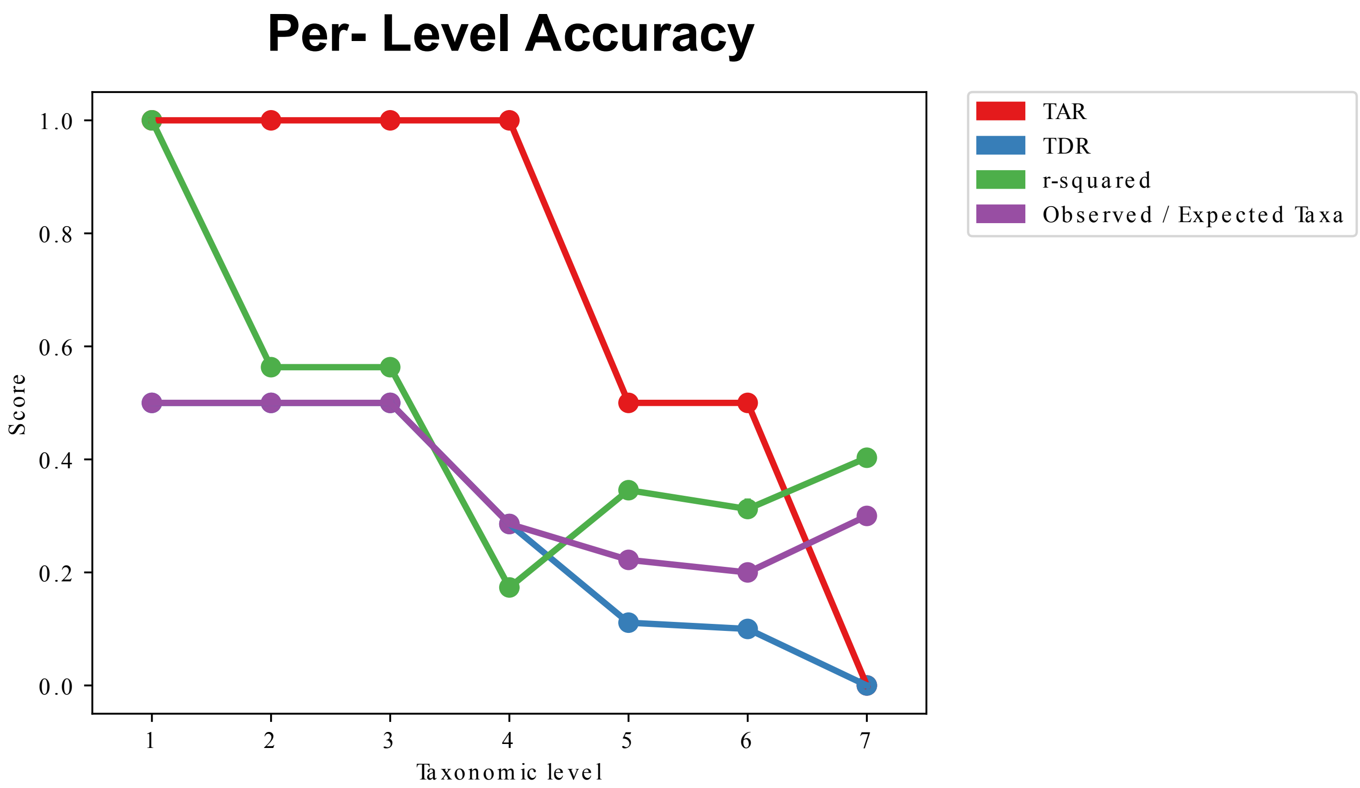

| Sample | level | Observed Taxa | Observed / Expected Taxa | TAR | TDR | Slope | Intercept | r-value | P value | Std Err | Bray-Curtis | Jaccard | r-squared |
|---|---|---|---|---|---|---|---|---|---|---|---|---|---|
| C001 | 1 | 1 | 0.5 | 1 | 0.5 | -1.228501229 | 1.228501229 | -1 | 0 | 0 | 0.829826167 | 0.5 | 1 |
| C002 | 1 | 1 | 0.5 | 1 | 0.5 | -1.228501229 | 1.228501229 | -1 | 0 | 0 | 0.829826167 | 0.5 | 1 |
| C003 | 1 | 1 | 0.5 | 1 | 0.5 | -1.228501229 | 1.228501229 | -1 | 0 | 0 | 0.829826167 | 0.5 | 1 |
| C004 | 1 | 1 | 0.5 | 1 | 0.5 | -1.228501229 | 1.228501229 | -1 | 0 | 0 | 0.829826167 | 0.5 | 1 |
| C001 | 2 | 2 | 0.5 | 1 | 0.5 | -0.692961756 | 0.455463161 | -0.750911307 | 0.249088693 | 0.430937915 | 0.829826167 | 0.5 | 0.56386779 |
| C002 | 2 | 2 | 0.5 | 1 | 0.5 | -0.692961756 | 0.455463161 | -0.751039507 | 0.248960493 | 0.430769232 | 0.829826167 | 0.5 | 0.564060341 |
| C003 | 2 | 2 | 0.5 | 1 | 0.5 | -0.692961756 | 0.455463161 | -0.748975684 | 0.251024316 | 0.433487255 | 0.829826167 | 0.5 | 0.560964575 |
| C004 | 2 | 2 | 0.5 | 1 | 0.5 | -0.692961756 | 0.455463161 | -0.750918677 | 0.249081323 | 0.430928216 | 0.829826167 | 0.5 | 0.56387886 |
| C001 | 3 | 2 | 0.5 | 1 | 0.5 | -0.692961756 | 0.455463161 | -0.750911307 | 0.249088693 | 0.430937915 | 0.829826167 | 0.5 | 0.56386779 |
| C002 | 3 | 2 | 0.5 | 1 | 0.5 | -0.692961756 | 0.455463161 | -0.751039507 | 0.248960493 | 0.430769232 | 0.829826167 | 0.5 | 0.564060341 |
| C003 | 3 | 2 | 0.5 | 1 | 0.5 | -0.692961756 | 0.455463161 | -0.748975684 | 0.251024316 | 0.433487255 | 0.829826167 | 0.5 | 0.560964575 |
| C004 | 3 | 2 | 0.5 | 1 | 0.5 | -0.692961756 | 0.455463161 | -0.750918677 | 0.249081323 | 0.430928216 | 0.829826167 | 0.5 | 0.56387886 |
| C001 | 4 | 2 | 0.285714286 | 1 | 0.285714286 | -0.811156664 | 0.280290258 | -0.416585855 | 0.352516264 | 0.791635291 | 0.829826167 | 0.714285714 | 0.173543774 |
| C002 | 4 | 2 | 0.285714286 | 1 | 0.285714286 | -0.811156664 | 0.280290258 | -0.416635642 | 0.352452763 | 0.791520826 | 0.829826167 | 0.714285714 | 0.173585258 |
| C003 | 4 | 2 | 0.285714286 | 1 | 0.285714286 | -0.811156664 | 0.280290258 | -0.415833219 | 0.353476794 | 0.793368651 | 0.829826167 | 0.714285714 | 0.172917266 |
| C004 | 4 | 2 | 0.285714286 | 1 | 0.285714286 | -0.811156664 | 0.280290258 | -0.416588717 | 0.352512613 | 0.791628709 | 0.829826167 | 0.714285714 | 0.17354616 |
| C001 | 5 | 2 | 0.222222222 | 0.5 | 0.111111111 | -1.9004661 | 0.325395279 | -0.587746513 | 0.073958823 | 0.924906485 | 0.914913083 | 0.9 | 0.345445964 |
| C002 | 5 | 2 | 0.222222222 | 0.5 | 0.111111111 | -1.887871681 | 0.323901581 | -0.58391381 | 0.076334357 | 0.927974742 | 0.914913083 | 0.9 | 0.340955337 |
| C003 | 5 | 2 | 0.222222222 | 0.5 | 0.111111111 | -1.948099664 | 0.33104462 | -0.601505711 | 0.065828218 | 0.914748529 | 0.914913083 | 0.9 | 0.36180912 |
| C004 | 5 | 2 | 0.222222222 | 0.5 | 0.111111111 | -1.868303657 | 0.321580814 | -0.57780336 | 0.080222914 | 0.933052825 | 0.914913083 | 0.9 | 0.333856722 |
| C001 | 6 | 2 | 0.2 | 0.5 | 0.1 | -2.061603124 | 0.313187391 | -0.558647764 | 0.074043375 | 1.020263616 | 0.914913083 | 0.909090909 | 0.312087325 |
| C002 | 6 | 2 | 0.2 | 0.5 | 0.1 | -2.045562756 | 0.311457948 | -0.554359022 | 0.076772337 | 1.023690473 | 0.914913083 | 0.909090909 | 0.307313925 |
| C003 | 6 | 2 | 0.2 | 0.5 | 0.1 | -2.122269671 | 0.319728348 | -0.574179623 | 0.06470559 | 1.008722946 | 0.914913083 | 0.909090909 | 0.32968224 |
| C004 | 6 | 2 | 0.2 | 0.5 | 0.1 | -2.020640739 | 0.308770901 | -0.547551171 | 0.081240752 | 1.02931993 | 0.914913083 | 0.909090909 | 0.299812284 |
| C001 | 7 | 3 | 0.3 | 0 | 0 | -1.834953229 | 0.244327271 | -0.635226632 | 0.019652847 | 0.672667579 | 1 | 1 | 0.403512874 |
| C002 | 7 | 3 | 0.3 | 0 | 0 | -1.834953229 | 0.244327271 | -0.635128987 | 0.019677555 | 0.672840946 | 1 | 1 | 0.40338883 |
| C003 | 7 | 3 | 0.3 | 0 | 0 | -1.834953229 | 0.244327271 | -0.633465836 | 0.020101959 | 0.675799268 | 1 | 1 | 0.401278965 |
| C004 | 7 | 3 | 0.3 | 0 | 0 | -1.834953229 | 0.244327271 | -0.635306293 | 0.019632707 | 0.672526169 | 1 | 1 | 0.403614085 |

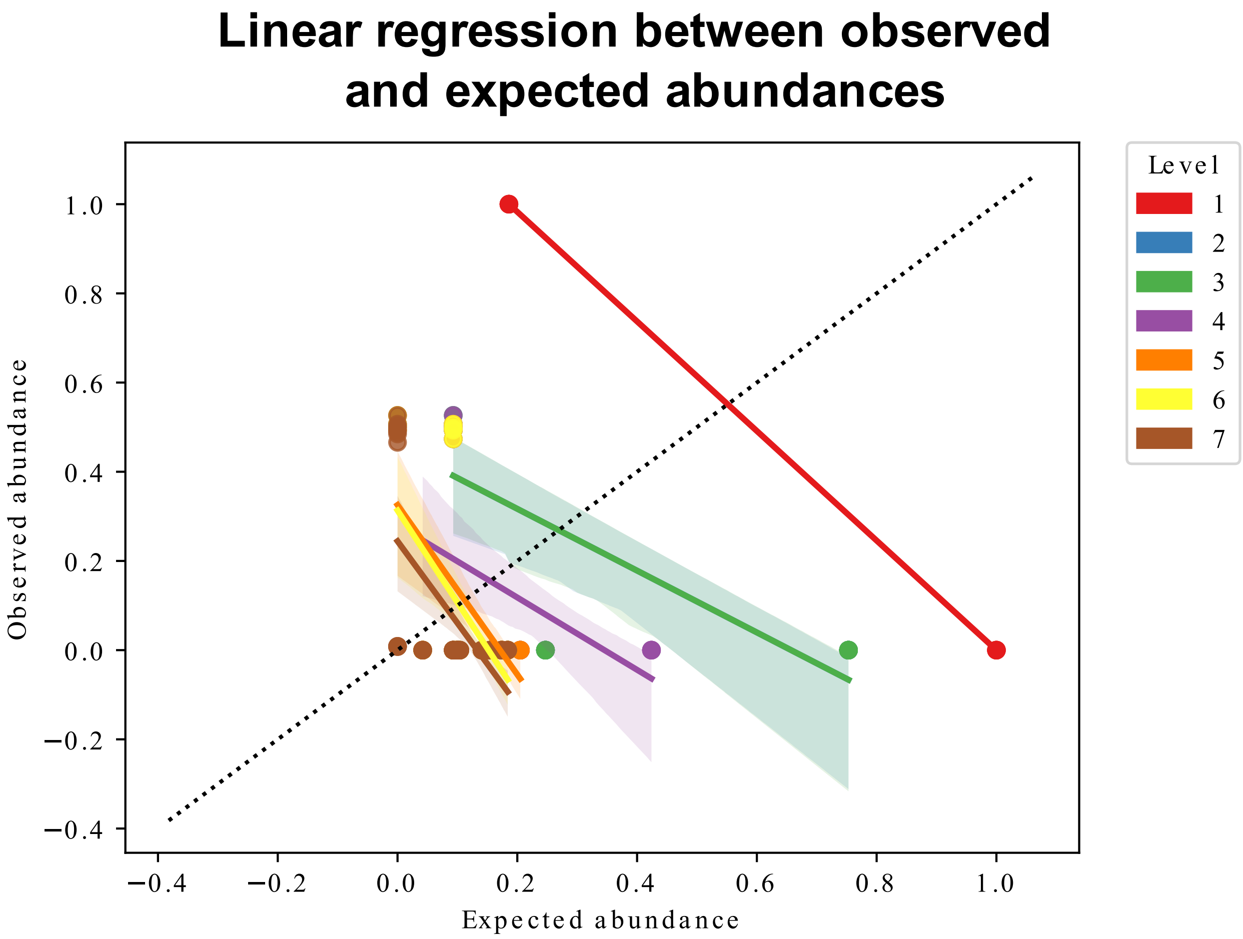


## 5.2 False positives: misclassifications

| Taxon | C001 | C002 | C003 | C004 |
|---|---|---|---|---|
| dEukaryota;p_Basidiomycota;c_Tremellomycetes;o_Tremellales;f_Cryptococcaceae;g_Cryptococcus;s__Cryptococcus_neoformans | 0.484728969 | 0.490224317 | 0.465872425 | 0.498092944 |

## 5.3 False positives: underclassifications

| Taxon | C001 | C002 | C003 | C004 |
|---|---|---|---|---|
| dEukaryota;p_Ascomycota;c_Saccharomycetes;o_Saccharomycetales;;;__ | 0.506709634 | 0.50152745 | 0.526309258 | 0.493475861 |
| dEukaryota;p_Basidiomycota;c_Tremellomycetes;o_Tremellales;f_Cryptococcaceae;g_Cryptococcus;__ | 0.008561397 | 0.008248233 | 0.007818317 | 0.008431195 |

## 5.4 False negatives

The taxonomy classifier can accurately assign taxonomy only up to the genus level. Since, all community standards are a priory identified at species level, this leads to the classification of every feature as a false negative.

| Taxon | C001 | C002 | C003 | C004 |
|---|---|---|---|---|
| d_Bacteria;p_Firmicutes;c_Bacilli;o_Bacillales;f_Bacillaceae;g_Bacillus;s__Bacillus_subtilis | 0.174 | 0.174 | 0.174 | 0.174 |
| d_Bacteria;p_Firmicutes;c_Bacilli;o_Lactobacillales;f_Enterococcaceae;g_Enterococcus;s__Enterococcus_faecalis | 0.099 | 0.099 | 0.099 | 0.099 |
| d_Bacteria;p_Firmicutes;c_Bacilli;o_Lactobacillales;f_Lactobacillaceae;g_Limosilactobacillus;s__Lactobacillus_fermentum | 0.184 | 0.184 | 0.184 | 0.184 |
| d_Bacteria;p_Firmicutes;c_Bacilli;o_Lactobacillales;f_Listeriaceae;g_Listeria;s__Listeria_monocytogenes | 0.141 | 0.141 | 0.141 | 0.141 |
| d_Bacteria;p_Firmicutes;c_Bacilli;o_Staphylococcales;f_Staphylococcaceae;g_Staphylococcus;s__Staphylococcus_aureus | 0.155 | 0.155 | 0.155 | 0.155 |
| d_Bacteria;p_Proteobacteria;c_Gammaproteobacteria;o_Enterobacterales;f_Enterobacteriaceae;g_Escherichia-Shigella;s__Escherichia_coli | 0.101 | 0.101 | 0.101 | 0.101 |
| d_Bacteria;p_Proteobacteria;c_Gammaproteobacteria;o_Enterobacterales;f_Enterobacteriaceae;g_Salmonella;s__Salmonella_enterica | 0.104 | 0.104 | 0.104 | 0.104 |
| d_Bacteria;p_Proteobacteria;c_Gammaproteobacteria;o_Pseudomonadales;f_Pseudomonadaceae;g_Pseudomonas;s__Pseudomonas_aeruginosa | 0.042 | 0.042 | 0.042 | 0.042 |
| d_Eukaryota;p_Ascomycota;c_Saccharomycetes;o_Saccharomycetales;f_Saccharomycetaceae;g_Saccharomyces;s__Saccharomyces_cerevisiae | 0.093 | 0.093 | 0.093 | 0.093 |

| Taxon | C001 | C002 | C003 | C004 |
| --- | --- | --- | --- | --- |
| d_Eukaryota;p_Basidiomycota;c_Tremellomycetes;o_Tremellales;f_Cryptococcaceae;g_Cryptococcus;s__Cryptococcus_gattii | 0.093 | 0.093 | 0.093 | 0.093 |
