# Supplementary material for: Early-life factors shaping the gut microbiota of Common buzzard nestlings
Source: Anim Microbiome. 2024 May 14;6:27. doi: 10.1186/s42523-024-00313-8 (PMC11092241; doi:10.1186/s42523-024-00313-8)
Supplement: Supplementary file 4 — Supplementary file1 (PDF 1093 kb) [file 42523_2024_313_MOESM4_ESM.pdf]

Sequence data processing

Table of Contents

Sequence data processing
- Table of Contents

A) 16s rRNA sequence data processing
- 1. Activate Qiime2
- 2. Import sequences
- 3. Visualize quality plots
  - 3.1. Summary of raw reads
  - 3.2. Quality plots
- 4. Run dada2
  - 4.1. DADA2 results
    - 4.1.1. Table summary
    - 4.1.2. Summary reads per samples
    - 4.1.2. Sequence Length Statistics
    - 4.1.3. Denoising statistics
- 5. Taxonomy assignment
  - 5.1. Taxonomy visualisation
- 6. Exit qiime2
- 7. In R run decontam
  - 7.1. Read in the data
  - 7.2. Run decontam
  - 7.3. Export feature table as biom file
- 8. Import biom table from R to qiime2
- 9. Remove control samples from dataset
  - 9.1. Table summary after decontam
- 10. Taxonomy based filtering
  - 10.1. Table summary after taxonomic filtering
- 11. Filter unique features
- 12. Filter samples with less than 500 reads
  - 12.1 Table summary after filtering unique features and samples with less than 500 reads
- 13. Filter representative sequences
- 14. Building a phylogenetic tree
- 15. Rarefaction curves
- 16. Taxa bar plots
  - 16.1. Plot core phylum (present in 70% of samples)
  - 16.2. Plot core families (present in 70% of samples)
- 17. Calculate alpha diversity metrics and rarefy the data-set
- 18. Filter samples with less than 4000 reads (unrarefied table for beta analysis)
  - 18.1. Table Summary
  - 18.2. Summary of final reads per sample
  - 18.3. Sequence length statistics

B) 28s rRNA sequence data processing
- 1. Activate Qiime2
- 2. Import sequences
- 3. Visualize quality plots
  - 3.1 Summary of raw reads
  - 3.2 Quality plots
- 4. Exit qiime
- 5. Run R pipeline to concatenate reads
- 6. Import data from R to qiime2
  - 6.1. Visualising results from R pipeline
    - 6.1.1 Summary of primer trimmed reads
    - 6.1.2. Primer trimmed quality plots
    - 6.1.3 Denoising statistics
    - 6.1.4. Table summary (after denoising)

6.1.5 Summary reads per sample
6.1.6. Sequence Length Statistics
7. Taxonomy assignment
8. Exit qiime
8. In R run decontam
8.1. Read the data
8.2. Run decontam
8.3. Export feature table as biom file
9. Import biom table from R to qiime2
10. Remove control samples from dataset
10.1 Table summary after decontam
11. Taxonomy based filtering
11.1. Table summary after taxonomic filtering
12. Filter unique features
13. Filter samples with less than 500 reads
13.1 Table summary after filtering unique features and samples with less than 500 reads
14. Filter representative sequences
15. Build a phylogenetic tree
16. Rarefaction curves
16.1 Rarefaction plots (sampling depth 20,000)
16.2 Rarefaction plots (sampling depth 2000)
17. Taxa bar plots
17.1. Plot core phylum (present in 70% of samples)
17.2. Plot core families (present in 70% of samples)
18. Calculate alpha diversity metrics and rarefy the data-set
19. Filter samples with less than 2000 reads (unrarefied table for beta analysis)
19.1. Table Summary
19.2. Summary of final reads per sample
24.2. Sequence length statistics

A) 16s rRNA sequence data processing

1. Activate Qiime2

```
# Activate base env
. ~/.bashrc

# Activate qiime2
conda activate qiime2-2022.11
```

2. Import sequences

```
qiime tools import --type 'SampleData[PairedEndSequencesWithQuality]'  --input-path seqs --input-format
CasavaOneEightSingleLanePerSampleDirFmt --output-path demux-paired-end.qza
```

3. Visualize quality plots

```
qiime demux summarize --i-data demux-paired-end.qza --o-visualization demux-quality-plots.qzv
```

3.1. Summary of raw reads

	Forward reads	Reverse reads
**Minimum**	77	77
**Median**	59119	59119
**Mean**	57956.0754	57956.0754
**Maximum**	96300	96300
**Total**	14604931	14604931

3.2. Quality plots

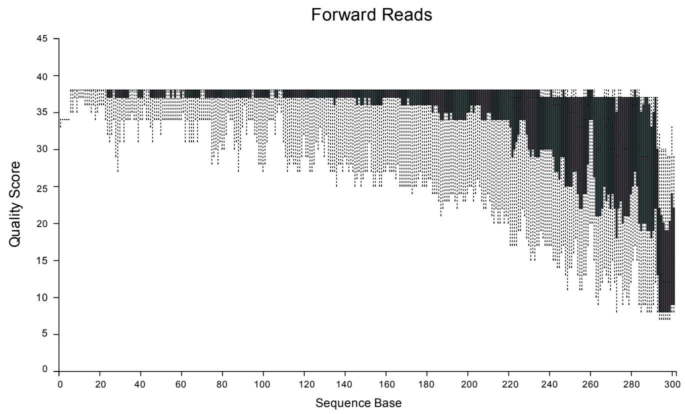

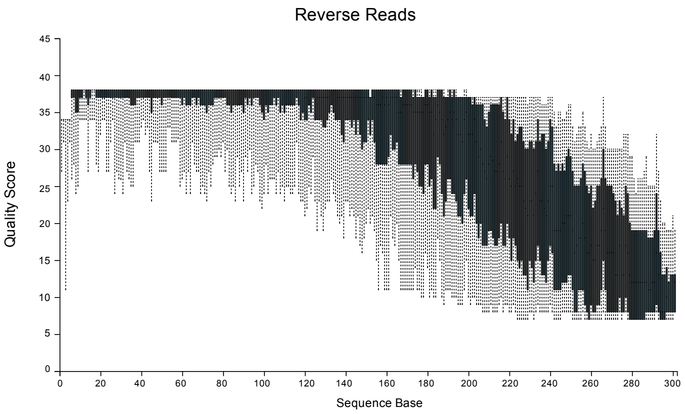

4. Run dada2

```
qiime dada2 denoise-paired --i-demultiplexed-seqs demux-paired-end.qza --p-trim-left-f 20 --p-trim-left-r 20 --
p-trunc-len-f 253 --p-trunc-len-r 185  --p-trunc-q 2 --o-table table.qza --o-representative-sequences rep-
seqs.qza --o-denoising-stats denoising-stats.qza
```

4.1. DADA2 results

```
qiime metadata tabulate --m-input-file denoising-stats.qza --o-visualization denoising-stats.qzv |

qiime feature-table tabulate-seqs --i-data rep-seqs.qza --o-visualization rep-seqs.qzv |

qiime feature-table summarize --i-table table.qza --o-visualization table.qzv --m-sample-metadata-file
buzzard_meta.tsv
```

4.1.1. Table summary

	Sample
**Number of samples**	252
**Number of features (ASVs)**	10,065
**Total frequency**	6,319,664

4.1.2. Summary reads per samples

	Frequency
**Minimum frequency**	0
**1st quartile**	19,110.25
**Median frequency**	25,091.50
**3rd quartile**	31,849.25
**Maximum frequency**	50,886.00
**Mean frequency**	25,078.03

4.1.2. Sequence Length Statistics

Sequence count	Min Lenght	Max Lenght	Mean Lenght	Range	Standard Deviation
10065	233	383	254.814	150	17.408

4.1.3. Denoising statistics

sample-id	input	filtered	% passed filter	denoised	merged	% merged	non-chimeric	% non-chimeric
S003	96300	58404	60.65	56348	50585	52.53	45952	47.72
S038	92164	48317	52.43	47278	43590	47.3	38298	41.55
S217	90239	49933	55.33	49013	46227	51.23	38079	42.2
S221	87443	55218	63.15	53518	48437	55.39	37075	42.4
S205	87439	59977	68.59	58853	55632	63.62	50886	58.2
S164	87271	43790	50.18	43042	40948	46.92	37559	43.04
S030	85926	4638	5.4	4508	4401	5.12	4342	5.05
S212	85532	56121	65.61	54638	50880	59.49	37847	44.25
S213	84629	58903	69.6	57329	54191	64.03	49144	58.07
S019	83332	50069	60.08	49187	45332	54.4	38807	46.57
S037	82624	17935	21.71	17598	16724	20.24	15902	19.25
S220	82614	43457	52.6	42528	39851	48.24	33789	40.9
S059	82183	30690	37.34	29999	27654	33.65	20931	25.47
S211	80977	52883	65.31	51410	47277	58.38	40568	50.1
S040	80597	48181	59.78	47110	43274	53.69	36857	45.73
S209	80562	53648	66.59	52551	50213	62.33	46545	57.78
S022	80114	50013	62.43	49134	44668	55.76	36144	45.12
S015	79621	48466	60.87	47365	42616	53.52	34222	42.98
S007	79600	52155	65.52	51053	46045	57.85	35038	44.02
S215	79463	50041	62.97	49024	45789	57.62	37356	47.01
S036	79365	50393	63.5	49629	45458	57.28	37053	46.69
S083	78879	61369	77.8	59699	55470	70.32	45273	57.4
S181	78712	42310	53.75	41285	38594	49.03	31808	40.41
S232	77656	50973	65.64	49738	46126	59.4	40121	51.67

sample-id	input	filtered	% passed filter	denoised	merged	% merged	non-chimeric	% non-chimeric
S179	77634	47088	60.65	46362	43072	55.48	33997	43.79
S063	77524	44579	57.5	43500	39391	50.81	31859	41.1
S034	77442	58069	74.98	56893	52726	68.08	42574	54.98
S075	76606	36828	48.07	35739	31863	41.59	23219	30.31
S216	75827	50239	66.25	49506	47252	62.32	41985	55.37
S056	75800	50044	66.02	49019	44828	59.14	37003	48.82
S163	75721	2505	3.31	2314	2226	2.94	2203	2.91
S078	75649	1482	1.96	1332	1307	1.73	1292	1.71
S201	74881	44613	59.58	43719	41161	54.97	30433	40.64
S004	74031	48362	65.33	47434	43757	59.11	35262	47.63
S204	74004	49523	66.92	48501	45422	61.38	41307	55.82
S023	73945	41690	56.38	40881	37554	50.79	29927	40.47
S196	73922	50374	68.14	47874	42102	56.95	36975	50.02
S116	73882	46214	62.55	45101	40587	54.93	33822	45.78
S165	73640	42918	58.28	42142	39050	53.03	35362	48.02
S021	73578	48044	65.3	47013	43961	59.75	39050	53.07
S024	73463	50915	69.31	49304	44494	60.57	37493	51.04
S197	73361	44421	60.55	43204	39797	54.25	29948	40.82
S062	73249	41450	56.59	40521	35925	49.05	29699	40.55
S203	73233	45331	61.9	44361	40658	55.52	32239	44.02
S180	73074	30038	41.11	29002	26553	36.34	23114	31.63
S200	72937	50991	69.91	50125	47723	65.43	42203	57.86
S171	72883	36414	49.96	35582	32120	44.07	27687	37.99
S155	72777	47482	65.24	45938	41021	56.37	31846	43.76
S154	72589	47452	65.37	46364	42661	58.77	32509	44.79
C010	72576	46168	63.61	45603	44709	61.6	42101	58.01
S233	72551	42679	58.83	41510	37189	51.26	31916	43.99
S028	72430	45992	63.5	45188	41707	57.58	32070	44.28
S014	72125	46933	65.07	45845	40939	56.76	33443	46.37
S147	72124	39861	55.27	38649	34999	48.53	30439	42.2
C002	71473	56446	78.98	55874	51033	71.4	33041	46.23
S051	71384	47623	66.71	46791	42973	60.2	32722	45.84
S219	71176	48473	68.1	47297	42988	60.4	29703	41.73
S178	71036	26365	37.11	25821	24415	34.37	22331	31.44
S189	70915	40772	57.49	40089	37141	52.37	29715	41.9
S077	70538	39494	55.99	38315	34245	48.55	29182	41.37
S093	70217	23402	33.33	22816	20619	29.36	16777	23.89
S005	70073	35633	50.85	34245	31083	44.36	26955	38.47
S208	69546	43644	62.76	42701	38509	55.37	35221	50.64
S092	69518	37010	53.24	36187	33140	47.67	24280	34.93

sample-id	input	filtered	% passed filter	denoised	merged	% merged	non-chimeric	% non-chimeric
S054	69214	42018	60.71	40821	36245	52.37	31817	45.97
S101	69051	40651	58.87	39642	37111	53.74	34198	49.53
S035	68697	48326	70.35	47697	44675	65.03	34856	50.74
S002	68603	38702	56.41	37938	34948	50.94	29477	42.97
S157	68541	44408	64.79	43433	39929	58.26	33042	48.21
S140	68281	26759	39.19	26224	24430	35.78	21169	31
S172	68069	46526	68.35	46126	43114	63.34	34885	51.25
S060	67868	43185	63.63	42092	38334	56.48	28284	41.68
S020	67737	41752	61.64	40818	38103	56.25	33070	48.82
S117	67651	118	0.17	71	71	0.1	71	0.1
C001	67583	54456	80.58	54034	49960	73.92	32475	48.05
S223	67531	44635	66.1	43631	40767	60.37	31378	46.46
S110	67060	40417	60.27	39763	36565	54.53	30909	46.09
S043	66900	42302	63.23	40949	36396	54.4	25895	38.71
S044	66815	32352	48.42	31621	28936	43.31	24573	36.78
S008	66781	31219	46.75	30569	28037	41.98	24338	36.44
S089	66561	36900	55.44	36163	34085	51.21	30515	45.85
S193	66415	41938	63.15	41171	38398	57.82	30852	46.45
S094	65947	36235	54.95	34997	31409	47.63	25977	39.39
S188	65939	35875	54.41	35229	33501	50.81	27919	42.34
S102	65649	40897	62.3	39965	36737	55.96	30904	47.07
S124	65641	33538	51.09	32755	29361	44.73	26939	41.04
S125	65468	27392	41.84	26706	24548	37.5	22545	34.44
S148	65338	40952	62.68	40033	37832	57.9	35724	54.68
S146	65229	30050	46.07	29052	26791	41.07	24220	37.13
S085	64527	44937	69.64	44028	40807	63.24	33317	51.63
S195	64504	41501	64.34	40661	37819	58.63	32932	51.05
S039	64471	41716	64.71	40566	35872	55.64	31448	48.78
S016	64279	39442	61.36	38545	35627	55.43	30191	46.97
S052	64148	38722	60.36	37623	34831	54.3	27101	42.25
S156	63812	43756	68.57	42716	39253	61.51	33183	52
S138	63789	38577	60.48	37633	33823	53.02	27040	42.39
S076	63731	48192	75.62	47106	42443	66.6	33499	52.56
S084	63722	50327	78.98	49383	46244	72.57	37967	59.58
C009	63651	41897	65.82	40971	38631	60.69	35354	55.54
S218	63447	27470	43.3	27033	25760	40.6	22637	35.68
S050	63419	23585	37.19	22937	21380	33.71	18520	29.2
S012	63351	33428	52.77	31881	29437	46.47	23021	36.34
S149	63222	29203	46.19	28565	26894	42.54	25135	39.76
S170	63131	30979	49.07	30289	28171	44.62	24093	38.16

sample-id	input	filtered	% passed filter	denoised	merged	% merged	non-chimeric	% non-chimeric
S198	62859	38887	61.86	38016	34972	55.64	25531	40.62
S018	62645	45777	73.07	44905	41444	66.16	33413	53.34
S123	62071	32263	51.98	31622	28991	46.71	25048	40.35
S031	62063	48032	77.39	47145	43216	69.63	32325	52.08
S011	62020	42200	68.04	41428	38682	62.37	30336	48.91
S055	62011	40629	65.52	39808	36427	58.74	28048	45.23
S207	61931	33778	54.54	32740	30068	48.55	25909	41.84
S086	61921	47016	75.93	46068	42815	69.14	30163	48.71
S139	61677	39097	63.39	38033	33959	55.06	27965	45.34
S091	61632	28436	46.14	27601	25279	41.02	18961	30.76
S032	61619	38819	63	38041	34668	56.26	26692	43.32
S192	61606	38185	61.98	37371	33823	54.9	27251	44.23
S067	61356	32004	52.16	31024	26614	43.38	16008	26.09
C017	61140	41244	67.46	39148	35980	58.85	35354	57.82
S088	60849	40560	66.66	39149	35769	58.78	25862	42.5
C011	60263	40108	66.55	39766	38899	64.55	36217	60.1
S107	59800	39848	66.64	38651	34916	58.39	31322	52.38
C003	59638	47299	79.31	46849	43109	72.28	27531	46.16
S227	59577	36938	62	36038	33056	55.48	23990	40.27
S162	59332	33802	56.97	32402	30047	50.64	26075	43.95
S071	59242	31726	53.55	30674	26390	44.55	20999	35.45
S026	59123	47092	79.65	46329	43367	73.35	35223	59.58
S194	59115	39552	66.91	38530	35590	60.2	28613	48.4
S118	58881	35601	60.46	34899	31670	53.79	25147	42.71
S108	58804	43886	74.63	43061	40104	68.2	32019	54.45
S042	58785	29320	49.88	28645	26345	44.82	23246	39.54
S006	58519	25753	44.01	25075	22907	39.14	19310	33
S082	58476	44102	75.42	43079	39403	67.38	29101	49.77
S234	58412	31191	53.4	30485	27984	47.91	22800	39.03
S222	58259	39575	67.93	38120	34080	58.5	24943	42.81
S199	58241	37551	64.48	36690	33646	57.77	25849	44.38
S235	58185	31809	54.67	30891	27700	47.61	21644	37.2
S087	58143	16650	28.64	16125	14987	25.78	11854	20.39
C004	57703	45212	78.35	44599	40329	69.89	26539	45.99
S047	57336	37692	65.74	37050	34060	59.4	25263	44.06
S141	57084	32259	56.51	31695	29648	51.94	25484	44.64
S184	56998	33014	57.92	32056	28064	49.24	21996	38.59
S079	56676	34007	60	33128	29771	52.53	22276	39.3
S070	56597	24543	43.36	23803	21055	37.2	17656	31.2
S130	56495	37346	66.1	36609	33889	59.99	26441	46.8

sample-id	input	filtered	% passed filter	denoised	merged	% merged	non-chimeric	% non-chimeric
S115	56465	38458	68.11	37670	33803	59.87	28761	50.94
S202	56287	34221	60.8	33591	31462	55.9	25432	45.18
S100	56278	40774	72.45	39177	35359	62.83	28488	50.62
S058	56080	34132	60.86	33218	28300	50.46	15427	27.51
C016	55808	36029	64.56	35187	33922	60.78	32822	58.81
S068	55570	40690	73.22	39177	34225	61.59	22518	40.52
S114	55550	38876	69.98	37828	33465	60.24	27304	49.15
S191	55545	27872	50.18	26000	21355	38.45	17235	31.03
C015	55534	36852	66.36	36318	35378	63.71	35096	63.2
S214	55367	30430	54.96	29945	27740	50.1	22413	40.48
S033	55365	37205	67.2	36381	33486	60.48	26239	47.39
S228	55260	28462	51.51	27515	24988	45.22	20701	37.46
S225	55198	31323	56.75	30149	27216	49.31	23944	43.38
S013	55085	25527	46.34	24954	22983	41.72	18768	34.07
S122	54679	39444	72.14	38654	35549	65.01	28746	52.57
S053	54494	39020	71.6	37762	33527	61.52	24989	45.86
S069	54228	33037	60.92	32233	27848	51.35	20217	37.28
S133	54053	28992	53.64	28244	25859	47.84	20407	37.75
S187	54029	23820	44.09	23188	21631	40.04	17101	31.65
S049	53865	39965	74.19	39059	35301	65.54	28122	52.21
S229	53641	31220	58.2	29976	26944	50.23	19536	36.42
S046	53484	34556	64.61	33599	30713	57.42	25141	47.01
S185	53420	23392	43.79	21808	18787	35.17	17918	33.54
S098	52975	33418	63.08	31964	28020	52.89	22915	43.26
S081	52893	38540	72.86	37438	33189	62.75	24417	46.16
S132	52815	33167	62.8	32701	30707	58.14	24459	46.31
S073	52711	30915	58.65	29873	25726	48.81	21135	40.1
S061	52687	35326	67.05	34537	31044	58.92	22268	42.26
S210	52626	33580	63.81	32399	29747	56.53	24376	46.32
S045	52482	32704	62.31	31986	29553	56.31	22554	42.97
S010	52453	31465	59.99	30428	26657	50.82	22012	41.97
S226	52086	34611	66.45	33137	29801	57.21	27040	51.91
S190	52020	31966	61.45	30968	28873	55.5	22173	42.62
S017	51988	38445	73.95	37541	34875	67.08	27280	52.47
S131	51799	34107	65.84	33271	30826	59.51	24353	47.01
S064	51696	31238	60.43	30381	26331	50.93	18831	36.43
S027	51674	32145	62.21	31397	29508	57.1	24432	47.28
S048	51632	24701	47.84	24093	22283	43.16	17952	34.77
S099	51271	35917	70.05	34883	31839	62.1	24977	48.72
S224	50549	30847	61.02	30031	27363	54.13	24880	49.22

sample-id	input	filtered	% passed filter	denoised	merged	% merged	non-chimeric	% non-chimeric
S161	50335	34900	69.34	34032	32154	63.88	29714	59.03
S109	50224	39865	79.37	38543	34341	68.38	28447	56.64
S142	49862	33840	67.87	32772	30004	60.17	27518	55.19
S106	49768	36071	72.48	35481	32968	66.24	24962	50.16
S074	49550	26199	52.87	25301	23023	46.46	17714	35.75
S186	49221	18227	37.03	17755	16807	34.15	15058	30.59
S166	48913	32191	65.81	31315	29270	59.84	24528	50.15
S167	48262	32737	67.83	32133	30403	63	28343	58.73
S009	47415	24582	51.84	23633	21134	44.57	17777	37.49
C007	47327	23615	49.9	23317	22948	48.49	21032	44.44
S095	47187	27854	59.03	26974	24475	51.87	21046	44.6
S231	46898	32149	68.55	31550	29331	62.54	24558	52.36
S090	46878	16767	35.77	15686	13947	29.75	9250	19.73
S183	46438	23444	50.48	22742	21503	46.3	20029	43.13
S168	45965	27853	60.6	27320	25701	55.91	22290	48.49
S182	45552	25367	55.69	24566	22688	49.81	19160	42.06
S072	45242	32659	72.19	31814	29122	64.37	23105	51.07
S175	45176	22909	50.71	22237	20751	45.93	17863	39.54
S025	44563	31044	69.66	30372	27529	61.78	23043	51.71
S057	43890	25200	57.42	24523	22155	50.48	14507	33.05
S230	43713	28340	64.83	27885	25592	58.55	19614	44.87
S041	43673	28751	65.83	27901	25469	58.32	18760	42.96
S160	43186	25375	58.76	24419	22553	52.22	19431	44.99
S126	42603	23367	54.85	22541	19824	46.53	16537	38.82
S158	41818	26843	64.19	26336	24859	59.45	23494	56.18
C012	41792	25605	61.27	25317	24704	59.11	21687	51.89
C013	41164	24331	59.11	24063	23642	57.43	21431	52.06
S119	40284	23045	57.21	22219	20265	50.31	15888	39.44
S103	40189	26638	66.28	25856	23892	59.45	19681	48.97
S150	39799	23316	58.58	22808	21214	53.3	20261	50.91
S173	39422	20629	52.33	19701	18272	46.35	15495	39.31
S174	39265	24170	61.56	23630	22106	56.3	19340	49.26
S176	38519	23775	61.72	23196	21391	55.53	18352	47.64
S151	38092	25199	66.15	24833	23516	61.73	20076	52.7
S134	37618	18784	49.93	18062	16546	43.98	15863	42.17
S096	37497	19313	51.51	18785	16978	45.28	13712	36.57
S177	36442	22686	62.25	22076	20497	56.25	17023	46.71
S065	35931	21253	59.15	20539	18040	50.21	12046	33.53
S143	35842	21937	61.2	21101	19553	54.55	17377	48.48
S080	35712	23199	64.96	22581	19934	55.82	14464	40.5

sample-id	input	filtered	% passed filter	denoised	merged	% merged	non-chimeric	% non-chimeric
S111	35263	17483	49.58	16903	15165	43.01	14287	40.52
S120	34869	20893	59.92	20368	18545	53.18	14197	40.72
S144	34592	20788	60.09	20334	18480	53.42	15473	44.73
S128	34529	21501	62.27	20895	19213	55.64	17834	51.65
S169	34280	21224	61.91	20727	19359	56.47	17966	52.41
S127	34043	17895	52.57	17400	15907	46.73	14789	43.44
S206	33959	999	2.94	881	880	2.59	880	2.59
S153	33516	20715	61.81	20263	18490	55.17	17126	51.1
S135	33004	16792	50.88	16014	14566	44.13	13442	40.73
C014	32172	19068	59.27	18905	18593	57.79	17217	53.52
S001	32034	10157	31.71	9704	8604	26.86	7132	22.26
S121	31957	20153	63.06	19420	17609	55.1	15347	48.02
S104	31423	18042	57.42	17528	16357	52.05	13687	43.56
S152	31416	20558	65.44	19947	18174	57.85	15751	50.14
S145	31277	18670	59.69	18369	17491	55.92	15961	51.03
S137	30416	13499	44.38	12918	11966	39.34	10758	35.37
S136	30170	19486	64.59	19035	17489	57.97	15655	51.89
S112	29839	19096	64	18471	16216	54.34	13275	44.49
S129	28991	7840	27.04	7597	7314	25.23	7159	24.69
S097	25677	17216	67.05	15799	13436	52.33	11189	43.58
S159	25671	78	0.3	47	43	0.17	43	0.17
S113	25054	14399	57.47	13765	12048	48.09	11649	46.5
S029	22180	9748	43.95	9418	8260	37.24	7819	35.25
S066	21855	15714	71.9	14297	11083	50.71	5583	25.55
S105	17877	9138	51.12	8673	7576	42.38	7216	40.36
C005	264	34	12.88	2	0	0	0	0
C006	224	26	11.61	1	0	0	0	0
C008	77	7	9.09	1	0	0	0	0

5. Taxonomy assignment

```
qiime feature-classifier classify-sklearn --i-classifier silva-138.1-SSU-nr99-515F-806R-classifier.qza --i-reads rep-seqs.qza --o-classification taxonomy.qza
```

5.1. Taxonomy visualisation

```
qiime metadata tabulate --m-input-file taxonomy.qza --o-visualization taxonomy.qzv
```

6. Exit qiime2

```
conda deactivate
```

7. In R run decontam

7.1. Read in the data

```
# Load Libraries
library(decontam)
library(qiime2R)
library(phyloseq)
library(biomformat)

# Make a phyloseq object
ps <- qza_to_phyloseq(features = "table.qza", taxonomy = "taxonomy.qza", metadata = "buzzard_meta.tsv")

# Choose which samples are the negative controls
sample_data(ps)$is.neg <- sample_data(ps)$type == "negative"
```

7.2. Run decontam

```
# Identify contaminants based on prevalence method (treshold 0.1 is the standard)
contamdf.prev <- isContaminant(ps, method="prevalence", neg="is.neg", threshold=0.1)

table(contamdf.prev$contaminant)
head(which(contamdf.prev$contaminant))

# Remove contaminants from the phyloseq object
ps.nocontam <- prune_taxa(!contamdf.prev$contaminant,ps)
```

7.3. Export feature table as biom file

```
# Extract asv table from the phyloseq object
table_nocontam <- as(otu_table(ps.nocontam),"matrix",)

#'t' to transform if taxa_are_rows=FALSE table_nocontam<- t(as(otu_table(ps.nocontam),"matrix",))#if
taxa_are_rows=TRUE

# Make a biom table
table_nocontam_biom <- make_biom(data=table_nocontam)
write_biom(table_nocontam_biom,"table-nocontam.biom")
```

8. Import biom table from R to qiime2

```
conda activate qiime2-2022.11

qiime tools import --input-path table-nocontam.biom --type 'FeatureTable[Frequency]' --input-format
BIOMV100Format --output-path table-nocontam.qza
```

9. Remove control samples from dataset

```
qiime feature-table filter-samples --i-table table-nocontam.qza --m-metadata-file buzzard_meta.tsv --p-where
"type='positive'" --p-exclude-ids --o-filtered-table table-nocontam.qza

qiime feature-table filter-samples --i-table table-nocontam.qza --m-metadata-file buzzard_meta.tsv --p-where
"type='negative'" --p-exclude-ids --o-filtered-table table-nocontam.qza
```

9.1. Table summary after decontam

	Sample
**Number of samples**	235
**Number of features (ASVs)**	9,487
**Total frequency**	5,640,334

10. Taxonomy based filtering

Filter out mitochondrial, chloroplast, unassigned, Vertebrata, Eukaryote and taxa not assigned to phylum

```
qiime taxa filter-table --i-table table-nocontam.qza --i-taxonomy taxonomy.qza --p-exclude
mitochondria,chloroplast,Unassigned,Vertebrata,Eukaryota --p-include p_ --o-filtered-table table-taxa-
filter.qza

qiime feature-table summarize --i-table table-taxa-filter.qza --o-visualization table-taxa-filter.qzv --m-
sample-metadata-file buzzard_meta.tsv
```

10.1. Table summary after taxonomic filtering

	Sample
**Number of samples**	235
**Number of features (ASVs)**	7,473
**Total frequency**	5,352,052

11. Filter unique features

```
qiime feature-table filter-features --i-table table-taxa-filter.qza --p-min-samples 2 --o-filtered-table table-
taxa-filter-no_singles.qza

qiime feature-table summarize --i-table table-taxa-filter-no_singles.qza --o-visualization table-taxa-filter-
no_singles.qzv --m-sample-metadata-file buzzard_meta.tsv
```

12. Filter samples with less than 500 reads

```
qiime feature-table filter-samples --i-table table-taxa-filter-no_singles.qza --p-min-frequency 500 --o-
filtered-table table-taxa-filter-no_singles.qza

qiime feature-table summarize --i-table table-taxa-filter-no_singles.qza --o-visualization table-taxa-filter-
no_singles.qzv --m-sample-metadata-file buzzard_meta.tsv
```

12.1 Table summary after filtering unique features and samples with less than 500 reads

	Sample
**Number of samples**	233
**Number of features (ASVs)**	2,078
**Total frequency**	5,125,527

13. Filter representative sequences

```
qiime feature-table filter-seqs --i-data rep-seqs.qza --i-table filtered-table.qza --o-filtered-data filter-seqs.qza
qiime feature-table tabulate-seqs --i-data filter-seqs.qza --o-visualization filter-seqs.qzv
```

14. Building a phylogenetic tree

```
qiime phylogeny align-to-tree-mafft-fasttree --i-sequences filter-seqs.qza  --o-alignment aligned-seqs.qza --o-masked-alignment masked-aligned-seqs.qza --o-tree unrooted-tree.qza  --o-rooted-tree rooted-tree.qza
```

15. Rarefaction curves

```
qiime diversity alpha-rarefaction --i-table filtered-table.qza --i-phylogeny rooted-tree.qza --p-max-depth 20000 --m-metadata-file buzzard_meta.tsv  --o-visualization alpha-rarefaction.qzv

qiime diversity alpha-rarefaction --i-table filtered-table.qza --i-phylogeny rooted-tree.qza --p-max-depth 4000 --m-metadata-file buzzard_meta.tsv  --o-visualization alpha-rarefaction.qzv
```

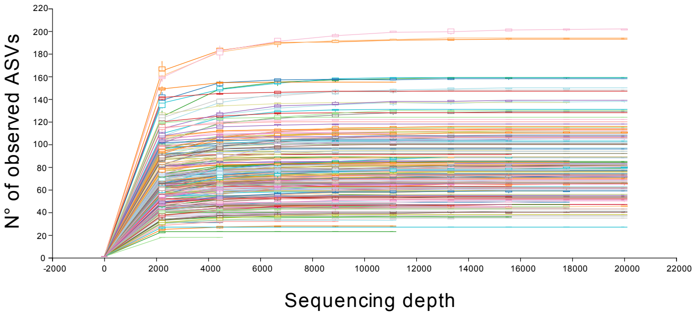

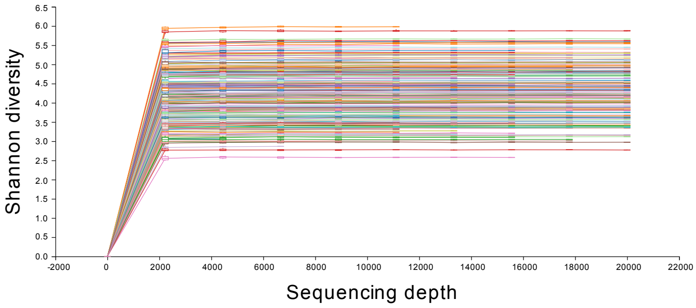

16. Taxa bar plots

```
qiime taxa barplot --i-table filtered-table.qza --i-taxonomy taxonomy.qza --m-metadata-file buzzard_meta.tsv --o-visualization taxa-bar-plots.qzv
```

16.1. Plot core phylum (present in 70% of samples)

```
qiime taxa collapse --i-table filtered-table.qza --i-taxonomy taxonomy.qza --o-collapsed-table phylum-table.qza
 --p-level 2

qiime feature-table summarize --i-table phylum-table.qza --o-visualization phylum-table.qzv --m-sample-
metadata-file buzzard_meta.tsv

qiime feature-table core-features --i-table phylum-table.qza --o-visualization phylum-core-table.qzv
```

From the visualization file download the tsv relative to features present in 70% of the samples (or any other % that I want)

```
qiime metadata tabulate --m-input-file core-features-0.700.tsv --o-visualization core-features-0.700.qzv

qiime feature-table filter-features --i-table filtered-table.qza --o-filtered-table table-phylum-core-0.700.qza
--m-metadata-file core-features-0.700.tsv

qiime taxa barplot --i-table table-phylum-core-0.700.qza --i-taxonomy taxonomy.qza --m-metadata-file
buzzard_meta.tsv --o-visualization core-phylum-bar-plots.qzv
```

**Feature ID**	**2%**	**9%**	**25%**	**50%**	**75%**	**91%**	**98%**
dBacteria;p_Firmicutes	2184.9	3847.59	6083.25	8120.5	11548.5	14100.62	17146.1
dBacteria;p_Actinobacteriota	1985.22	3404.05	4962.5	7219	9077.5	10892.29	13493.08
dBacteria;p_Proteobacteria	757.18	1443.83	2297.75	3928.5	5697.5	7565.78	10199.84
dBacteria;p_Bacteroidota	0	15.61	79	260	674	2138.07	8270.38
dBacteria;p_Campylobacterota	0	0	17.5	177.5	526.25	1400.73	3014.1

16.2. Plot core families (present in 70% of samples)

```
qiime taxa collapse --i-table filtered-table.qza --i-taxonomy taxonomy.qza --o-collapsed-table family-table.qza
 --p-level 5

qiime feature-table summarize --i-table -table.qza --o-visualization family-table.qzv --m-sample-metadata-file
buzzard_meta.tsv

qiime feature-table core-features --i-table family-table.qza --o-visualization family-core-table.qzv
```

From the visualization file download the tsv relative to features present in 70% of the samples (or any other % that I want)

```
qiime metadata tabulate --m-input-file core-features-0.700.tsv --o-visualization core-features-0.700.qzv

qiime feature-table filter-features --i-table filtered-table.qza --o-filtered-table table-family-core-0.700.qza
--m-metadata-file core-features-0.700.tsv

qiime taxa barplot --i-table table-family-core-0.700.qza --i-taxonomy taxonomy.qza --m-metadata-file
buzzard_meta.tsv --o-visualization core-family-bar-plots.qzv
```

**Feature ID**	**2%**	**9%**	**25%**	**50%**	**75%**	**91%**	**98%**
dBacteria;pActinobacteriota;cActinobacteria;oCorynebacteriales;f__Corynebacteriaceae	350.74	872.57	1722.75	3061	4669.25	6330.39	8077.36
dBacteria;pFirmicutes;cClostridia;oPeptostreptococcales-Tissierellales;f__Peptostreptococcaceae	44.64	221.27	977.5	2371.5	3711.5	5851.84	8581.56

Feature ID	2%	9%	25%	50%	75%	91%	98%
dBacteria;pActinobacteriota;cActinobacteria;oActinomycetales;f__Actinomycetaceae	0	9.61	128.5	1542	3403.25	5059.41	6521.82
dBacteria;pProteobacteria;cGammaproteobacteria;oEnterobacterales;f__Enterobacteriaceae	17.16	85.37	290.5	747	1892	3955.99	7019.76
dBacteria;pActinobacteriota;cActinobacteria;oPropionibacteriales;f__Propionibacteriaceae	0	39.61	116.75	344.5	803	1854.51	3679.56
dBacteria;pFirmicutes;cBacilli;oStaphylococcales;f__Gemellaceae	0	0	49.25	302	1325	3811.18	7817.5
dBacteria;pFirmicutes;cBacilli;oStaphylococcales;f__Staphylococcaceae	0	25.61	92.25	246.5	596.25	1271.9	2761.72
dBacteria;pFirmicutes;cBacilli;oLactobacillales;f__Lactobacillaceae	0	18	67	232	684	1414.26	3788.36
dBacteria;pFirmicutes;cBacilli;oLactobacillales;f__Enterococcaceae	0	0	64.5	208	569	1411.95	3548.1
dBacteria;pProteobacteria;cGammaproteobacteria;oXanthomonadales;f__Xanthomonadaceae	0	0	49.25	199	445	821.73	1358.34
dBacteria;pFirmicutes;cBacilli;oBacillales;f__Bacillaceae	0	0	50.75	182	399.75	837.68	1659.94
dBacteria;pCampylobacterota;cCampylobacteria;oCampylobacterales;f__Campylobacteraceae	0	0	12.25	173.5	505	1294.68	2993.46
dBacteria;pProteobacteria;cGammaproteobacteria;oBurkholderiales;f__Comamonadaceae	0	13.61	61.25	171.5	411	827	1608.6
dBacteria;pFirmicutes;cClostridia;oClostridiales;f__Clostridiaceae	0	0	6	87.5	482	1778.88	3636.3
dBacteria;pProteobacteria;cGammaproteobacteria;oPseudomonadales;f__Moraxellaceae	0	0	16.5	75	272.5	758.23	2257.12
dBacteria;pActinobacteriota;cActinobacteria;oBifidobacteriales;f__Bifidobacteriaceae	0	0	8.25	74.5	185.5	372.34	1114.06
dBacteria;pProteobacteria;cAlphaproteobacteria;oRhizobiales;f__Rhizobiaceae	0	0	14	50.5	174	424.24	827.8
dBacteria;pBacteroidota;cBacteroidia;oFlavobacteriales;f__Weeksellaceae	0	0	5	31.5	117.75	467.9	1931.38
dBacteria;pProteobacteria;cAlphaproteobacteria;oSphingomonadales;f__Sphingomonadaceae	0	0	4.25	27	79.75	189.07	461.82

17. Calculate alpha diversity metrics and rarefy the data-set

```
# Calculates observed features, shannon diversity, Faith PD and retrieves rarefied table
qiime diversity core-metrics-phylogenetic --i-phylogeny rooted-tree.qza --i-table filtered-table.qza --p-sampling-depth 4000 --m-metadata-file buzzard_meta.tsv --output-dir alpha-metrics-results

#Add alpha diversity metrics to the metadata
qiime metadata tabulate --m-input-file buzzard_metadata.tsv --m-input-file shannon_vector.qza --m-input-file observed_features.qza --m-input-file faith_pd_vector.qza --o-visualization buzzard_meta_alpha.qzv
```

From the "buzzard_meta_alpha.qzv" one can extract the tsv file with the metadata + alpha metrics.

18. Filter samples with less than 4000 reads (unrarefied table for beta analysis)

```
qiime feature-table filter-samples --i-table filtered-table.qza --p-min-frequency 4000 --o-filtered-table beta-table.qza

qiime feature-table summarize --i-table beta-table.qza --o-visualization beta-table.qzv --m-sample-metadata-file buzzard_meta_alpha.tsv
```

18.1. Table Summary

	Sample
**Number of samples**	230
**Number of features (ASVs)**	2,078
**Total frequency**	5,121,868

18.2. Summary of final reads per sample

	Frequency
**Minimum frequency**	4,295.00
**1st quartile**	16,586.25
**Median frequency**	22,162.00
**3rd quartile**	27,903.50
**Maximum frequency**	43,074.00
**Mean frequency**	22,268.99

18.3. Sequence length statistics

Sequence Count	Min Length	Max Length	Mean Length	Range	Standard Deviation
2078	247	291	251.96	44	1.34

B) 28s rRNA sequence data processing

1. Activate Qiime2

```
# Activate base env
. ~/.bashrc

# Activate qiime2
conda activate qiime2-2022.11
```

2. Import sequences

```
qiime tools import --type 'SampleData[PairedEndSequencesWithQuality]'  --input-path seqs --input-format
CasavaOneEightSingleLanePerSampleDirFmt --output-path demux-paired-end.qza
```

3. Visualize quality plots

```
qiime demux summarize --i-data demux-paired-end.qza --o-visualization demux-quality-plots.qzv
```

3.1 Summary of raw reads

	Forward reads	Reverse reads
**Minimum**	311	311
**Median**	55034.5	55034.5
**Mean**	83582.93254	83582.93254
**Maximum**	475700	475700
**Total**	21062899	21062899

3.2 Quality plots

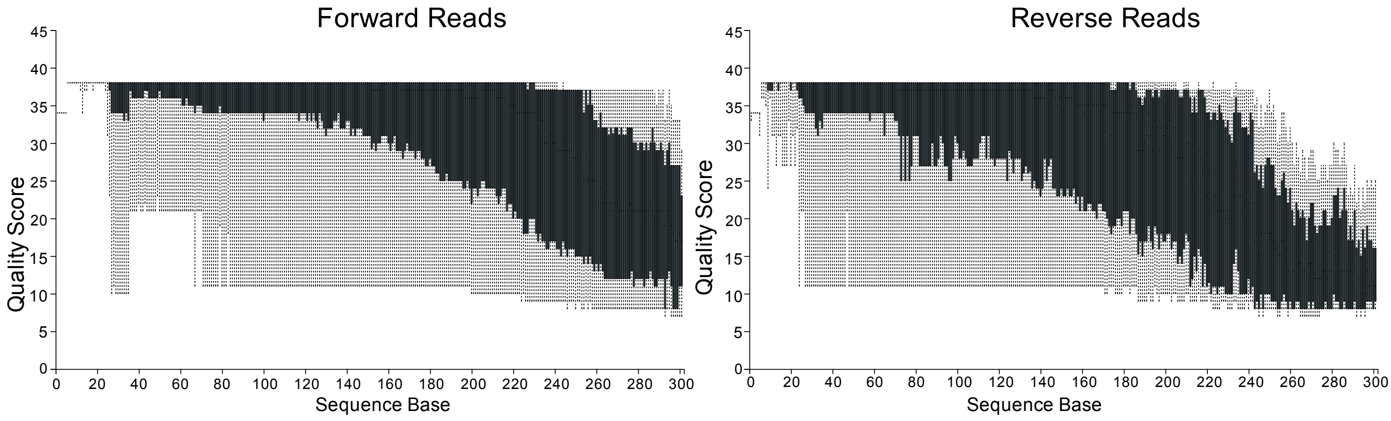

4. Exit qiime

```
conda deactivate
```

5. Run R pipeline to concatenate reads

```
Rscript 28s-concatenate-reads.R
```

6. Import data from R to qiime2

```
#Activate qiime2
conda activate qiime2-2022.11

# Import biom table (final result from DADA2 pipeline)
qiime tools import --input-path table.biom --type 'FeatureTable[Frequency]' --input-format BIOMV100Format --
output-path table.qza

# Import ASV sequences (fasta file)
qiime tools import --input-path ASV_nochim.fasta --output-path rep-seqs.qza --type 'FeatureData[Sequence]'
```

6.1. Visualising results from R pipeline

```
qiime feature-table tabulate-seqs --i-data rep-seqs.qza --o-visualization rep-seqs.qzv

qiime feature-table summarize --i-table table.qza --o-visualization table.qzv --m-sample-metadata-file
buzzard_meta.tsv
```

6.1.1 Summary of primer trimmed reads

	Forward reads	Reverse reads
**Minimum**	173	173
**Median**	30195.5	30195.5
**Mean**	34482.53571	34482.53571
**Maximum**	251247	251247
**Total**	8689599	8689599

6.1.2. Primer trimmed quality plots

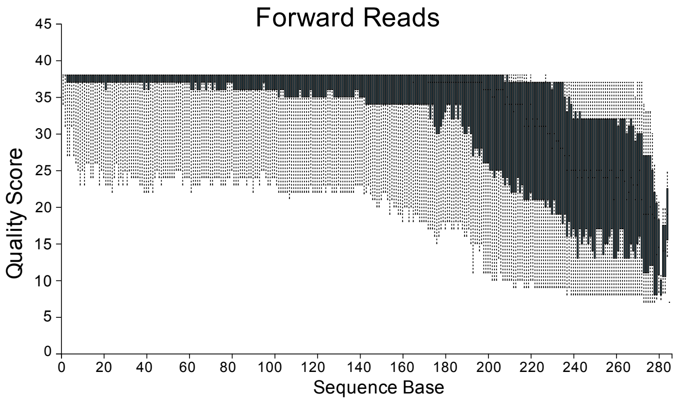

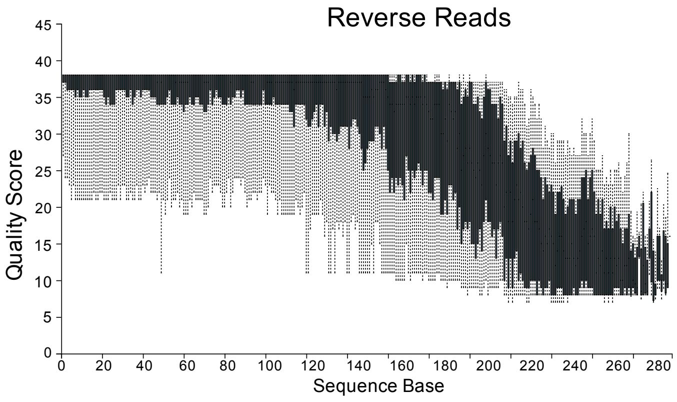

6.1.3 Denoising statistics

	input	filtered	denoisedF	denoisedR	merged	nonchim	perc-reads-retained
C017	35371	29638	29625	29632	29619	29371	83
S163	22696	17163	17154	17161	17152	17099	75
C001	28246	21402	21384	21400	21384	20803	74
C002	31108	23522	23504	23520	23503	22924	74
S008	6511	4904	4884	4903	4884	4801	74
C004	27244	20423	20412	20420	20412	19925	73
C003	22699	16546	16512	16543	16511	16149	71
S113	26199	18615	18589	18603	18578	16640	64
S170	20616	15256	15213	15250	15208	12918	63
S148	31151	23200	23096	23176	23073	18647	60
S112	22850	15774	15697	15746	15671	13520	59
S109	38911	23127	23025	23105	23014	22601	58
S123	23197	16543	16529	16543	16529	13561	58
S090	16251	11885	11870	11885	11870	9272	57

	input	filtered	denoisedF	denoisedR	merged	nonchim	perc-reads-retained
S068	33318	24445	24294	24443	24294	18476	55
S069	30704	22932	22806	22912	22788	16979	55
S132	6708	4262	4257	4234	4229	3657	55
S195	55896	40389	39953	40244	39810	30492	55
S071	18093	11814	11769	11792	11749	9759	54
S225	40220	29015	28789	28997	28775	21544	54
S003	30440	22844	22508	22735	22402	16214	53
S101	18178	12992	12940	12991	12940	9575	53
S025	32883	24044	23938	23942	23841	16890	51
S233	49268	37197	36850	37080	36733	24905	51
S021	25585	17860	17780	17857	17779	12755	50
S204	20324	12813	12781	12813	12781	10212	50
S226	41235	31258	30843	31179	30767	20597	50
S006	21252	15142	15091	15132	15081	10391	49
S032	23522	18061	18017	18057	18017	11612	49
S194	10759	6140	6074	6140	6074	5277	49
C010	33597	17576	17568	17576	17568	16019	48
S041	33204	24968	24720	24942	24695	16070	48
S060	32486	22731	22552	22684	22507	15633	48
S066	34840	25389	25013	25366	24993	16779	48
S076	35424	26248	26084	26151	25990	16942	48
S119	42073	24864	24737	24792	24667	20383	48
S017	30634	21799	21383	21488	21085	14446	47
S018	55890	29792	29428	29645	29316	26303	47
S014	32902	24305	23975	24219	23894	15037	46
S061	34639	26504	26310	26441	26247	15932	46
S079	37377	27736	27584	27641	27490	17362	46
S093	24130	16566	16519	16557	16510	11108	46
S103	26187	15671	15448	15667	15444	11967	46
S128	26380	16644	16587	16624	16567	12145	46
S138	22789	16834	16740	16809	16716	10465	46
S038	61961	31045	30959	30985	30899	27712	45
S108	24337	18071	17918	18071	17918	10833	45
S159	26904	13202	13047	13171	13018	11976	45
S192	40451	29571	29163	29408	29002	18285	45
S231	28874	21243	21185	21175	21117	12945	45
S020	28870	20791	20491	20629	20334	12668	44
S047	39766	24976	24827	24922	24775	17565	44
S067	40991	30786	30709	30750	30675	17981	44
S088	20604	15760	15438	15726	15407	9071	44

	input	filtered	denoisedF	denoisedR	merged	nonchim	perc-reads-retained
S140	26942	20024	19983	19939	19899	11745	44
S187	48396	21525	21517	21520	21513	21513	44
S013	38425	25491	25096	25377	24992	16362	43
S023	39463	26857	26702	26846	26694	16908	43
S043	72620	31283	31255	31272	31247	31212	43
S073	55052	27780	27543	27720	27483	23889	43
S083	31053	22965	22283	22913	22234	13428	43
S203	12218	6707	6670	6677	6640	5260	43
S227	36382	27473	27282	27451	27260	15714	43
S002	22783	16078	15949	16027	15901	9457	42
S052	56109	31723	31549	31586	31416	23790	42
S098	22890	16228	16056	16179	16008	9657	42
S102	21488	15842	15780	15839	15777	9131	42
S134	16772	9385	9334	9383	9333	7086	42
C015	41097	23188	23105	23187	23104	16852	41
S062	32866	22186	21928	22053	21798	13626	41
S155	28650	11720	11702	11714	11698	11683	41
S172	23896	16951	16838	16950	16838	9764	41
S202	59112	42421	42090	42316	41987	24340	41
S234	44740	33820	33520	33663	33368	18514	41
C016	45807	31968	31825	31944	31801	18266	40
S049	73142	35960	35572	35709	35330	29240	40
S080	19145	10772	10642	10769	10641	7710	40
S147	28326	21480	21197	21377	21096	11434	40
S150	24557	15543	15454	15543	15454	9849	40
S205	36977	23578	23453	23573	23450	14792	40
S216	42300	28771	28667	28770	28666	17091	40
S028	31811	22761	22544	22717	22502	12269	39
S065	38142	27515	27148	27460	27104	14952	39
S139	22203	16704	16625	16666	16588	8745	39
S146	22672	16440	16227	16148	15945	8889	39
S211	33956	23693	23531	23691	23530	13365	39
S228	31975	21433	21191	21350	21110	12533	39
S046	29702	18493	18334	18492	18334	11412	38
S057	27762	20973	20626	20969	20623	10686	38
S149	21868	15991	15933	15967	15909	8249	38
S212	33967	21786	21380	21683	21289	12927	38
S224	48814	33638	33557	33618	33537	18733	38
S026	29089	21980	21658	21940	21620	10896	37
S035	44428	27677	27479	27533	27341	16256	37

	input	filtered	denoisedF	denoisedR	merged	nonchim	perc-reads-retained
S104	21011	13749	13629	13745	13625	7723	37
S105	31837	12710	12635	12710	12635	11819	37
S153	21421	12693	12672	12692	12672	7982	37
S157	34706	24884	24573	24759	24450	12895	37
S075	36175	27058	26609	26970	26522	12891	36
S107	20000	14349	14324	14348	14324	7292	36
S110	31960	20130	20042	19991	19903	11515	36
S141	24448	17924	17635	17854	17565	8833	36
S156	26975	19934	19689	19870	19627	9741	36
S165	29464	21086	20848	20994	20757	10747	36
S167	36325	22416	22306	22333	22226	12909	36
S173	25247	16715	16403	16641	16333	9016	36
S189	24093	15081	14920	15002	14846	8632	36
S191	27743	16377	16270	16248	16141	10059	36
S217	47628	30762	30532	30740	30510	17038	36
C012	31718	19006	18978	18972	18944	11190	35
S016	18251	13541	13291	13499	13250	6326	35
S027	44061	18982	18582	18814	18423	15482	35
S106	25721	18656	18540	18577	18463	9085	35
S116	29984	22017	21619	21936	21546	10572	35
S144	30527	19798	19562	19659	19426	10592	35
S179	38453	16802	16701	16743	16644	13495	35
S180	31982	22145	21741	22072	21674	11256	35
S181	26645	18990	18485	18966	18463	9214	35
S190	28313	18761	18277	18661	18185	9803	35
S199	42169	27618	27116	27497	26999	14847	35
C013	26712	16110	16086	16104	16081	9160	34
S007	24637	18351	18219	18190	18062	8278	34
S054	31017	21940	21819	21896	21776	10507	34
S125	8137	3061	3058	3060	3058	2794	34
S131	25607	17594	17304	17472	17188	8593	34
S001	15561	10263	9995	10210	9946	5166	33
S085	50809	21565	21396	21542	21375	16832	33
S092	13478	9105	8901	9072	8869	4513	33
S121	23286	12861	12661	12758	12560	7614	33
S168	29227	19448	19326	19381	19260	9705	33
S171	29050	21553	21199	21499	21148	9711	33
S230	27796	18250	18077	18249	18076	9088	33
S232	59484	42012	41748	41872	41608	19827	33
S235	43166	30871	30561	30738	30431	14451	33

	input	filtered	denoisedF	denoisedR	merged	nonchim	perc-reads-retained
C011	32364	19846	19776	19822	19754	10207	32
S005	31953	23226	22805	23106	22695	10369	32
S045	38061	23452	23274	23394	23216	12012	32
S084	40364	16541	16364	16533	16359	12835	32
S091	17468	11066	10860	11007	10806	5585	32
S137	24996	16727	16353	16659	16286	7914	32
S029	22828	8502	8487	8500	8487	7036	31
S051	30907	22511	22263	22329	22085	9705	31
S058	45537	23210	23043	23196	23029	13995	31
S063	32269	24260	24077	24099	23917	9959	31
S122	24134	17923	17754	17844	17676	7443	31
S124	21227	15187	14941	15153	14907	6499	31
S126	29506	17252	17043	17221	17016	9034	31
S161	26267	16540	16443	16510	16415	8102	31
S196	28791	19165	18628	19062	18530	8786	31
S213	28232	16795	16600	16710	16520	8759	31
S219	47003	33969	33502	33792	33331	14733	31
S039	31498	22838	22653	22748	22566	9456	30
S040	48616	16403	16350	16380	16327	14793	30
S072	19538	14200	13927	14150	13882	5902	30
S082	26375	17851	17502	17793	17447	7921	30
S094	22734	16391	16220	16325	16156	6921	30
S130	20361	15236	15026	15219	15010	6196	30
S215	18162	12006	11708	11971	11677	5504	30
S221	43854	15450	15122	15413	15087	13130	30
S223	54361	16814	16738	16790	16718	16212	30
C014	54247	38730	38699	38729	38698	15950	29
S004	33755	24124	23948	24047	23875	9831	29
S142	35542	23221	22838	23127	22748	10430	29
S166	42896	24579	24271	24498	24196	12548	29
S009	18863	13241	13014	13154	12938	5229	28
S010	31671	23507	23068	23475	23042	8940	28
S011	29498	20491	20221	20418	20152	8251	28
S015	26507	19754	19553	19737	19536	7396	28
S036	64696	22419	22363	22411	22358	18402	28
S055	27449	20365	20165	20309	20109	7730	28
S074	85770	24261	24241	24256	24237	24208	28
S077	64814	21389	21298	21354	21268	18209	28
S097	25053	16525	16242	16434	16154	6965	28
S135	31390	20409	19828	20307	19730	8681	28

	input	filtered	denoisedF	denoisedR	merged	nonchim	perc-reads-retained
S177	29235	18438	18258	18333	18153	8138	28
S220	49357	26825	26727	26698	26601	13595	28
S034	36189	23049	22788	22944	22686	9943	27
S042	31005	22751	22469	22642	22362	8457	27
S053	47524	18386	18321	18240	18178	12701	27
S056	22123	16432	16194	16355	16118	5911	27
S099	21053	12115	11811	12110	11806	5768	27
S118	34170	21592	21321	21518	21249	9196	27
S133	32919	9929	9882	9919	9873	8906	27
S081	57521	20433	20066	20399	20036	14811	26
S152	34076	20030	19719	19942	19632	8717	26
S222	14463	4142	4087	4140	4086	3703	26
S111	44194	19433	19364	19410	19341	11051	25
S031	40396	22966	22616	22712	22374	9864	24
S120	24707	16100	15815	16000	15717	5963	24
S143	31693	19753	19392	19652	19294	7707	24
S012	31048	21738	21186	21629	21102	7135	23
S059	90778	20718	20682	20714	20681	20614	23
S136	20330	13731	13641	13651	13562	4642	23
S044	38056	17995	17905	17888	17799	8533	22
S214	7724	1979	1963	1975	1961	1708	22
S019	58483	17368	17104	17251	16992	12057	21
S022	42703	16350	16158	16295	16105	9116	21
S114	24650	10337	10227	10305	10196	5296	21
S145	38950	15601	15543	15548	15490	8278	21
S207	60787	12486	12467	12446	12432	12425	20
S096	45128	14889	14799	14861	14771	7958	18
S089	64206	10503	10417	10496	10415	10413	16
S095	70870	29143	28789	28880	28530	10693	15
S185	2104	285	283	285	283	283	13
S100	42218	11945	11762	11883	11703	4902	12
S218	30407	3306	3300	3305	3299	3195	11
S048	58900	5898	5876	5898	5876	5876	10
S024	69919	5537	5483	5511	5466	5390	8
S064	81415	5675	5622	5646	5595	5524	7
S188	36747	2491	2477	2479	2474	2474	7
S182	12749	800	784	799	784	781	6
S210	1697	93	85	93	85	85	5
S037	149434	5649	5600	5632	5588	5510	4
S160	14288	583	576	581	574	572	4

	input	filtered	denoisedF	denoisedR	merged	nonchim	perc-reads-retained
S176	6022	205	196	202	196	190	3
S193	28660	955	951	951	950	950	3
S198	82105	2321	2294	2297	2292	2238	3
C006	173	7	3	7	3	3	2
S070	163737	2837	2831	2836	2831	2773	2
S175	40368	996	984	993	984	984	2
C005	275	6	3	6	3	3	1
S086	3036	45	30	42	30	30	1
S162	22760	267	241	260	241	241	1
C007	290	6	1	5	1	1	0
C008	1165	4	1	3	1	1	0
C009	3375	1	1	1	1	1	0
S030	20564	13	4	1	0	0	0
S033	49673	25	5	22	5	5	0
S050	158711	84	71	73	60	60	0
S078	175800	10	2	1	0	0	0
S087	251247	1269	1192	1266	1190	1185	0
S115	6065	7	1	1	0	0	0
S117	3721	14	7	14	7	7	0
S127	85253	172	161	172	161	156	0
S129	5141	5	1	5	1	0	0
S151	8409	8	1	6	1	1	0
S154	10651	21	12	18	10	10	0
S158	87207	4	1	4	1	1	0
S164	5107	8	1	1	0	0	0
S169	5345	34	19	31	16	14	0
S174	63928	253	249	251	249	249	0
S178	1840	7	1	7	1	1	0
S183	10884	1	1	1	1	1	0
S184	1494	7	1	7	1	1	0
S186	4889	8	5	5	5	5	0
S197	57132	7	1	7	1	0	0
S200	13718	8	1	8	1	1	0
S201	22193	4	1	4	1	1	0
S206	11523	19	11	19	11	3	0
S208	2904	7	2	7	2	2	0
S209	4127	2	1	2	1	1	0
S229	7168	9	1	8	1	1	0

6.1.4. Table summary (after denoising)

	Sample
**Number of samples**	252
**Number of features (ASVs)**	14,143
**Total frequency**	2,710,821

6.1.5 Summary reads per sample

	Frequency
**Minimum frequency**	0
**1st quartile**	6,773.50
**Median frequency**	9,894.00
**3rd quartile**	15,024.00
**Maximum frequency**	33,905.00
**Mean frequency**	10,757.23

6.1.6. Sequence Length Statistics

Sequence Count	Min Length	Max Length	Mean Length	Range	Standard Deviation
14143	230	415	410.82	185	20.7

7. Taxonomy assignment

```
qiime feature-classifier classify-sklearn --i-classifier silva-138.1-LSU-nr99-GA20F-RM9R-classifier.qza --i-
reads rep-seqs.qza --o-classification taxonomy.qza

# Taxonomy visualisation
qiime metadata tabulate --m-input-file taxonomy.qza --o-visualization taxonomy.qzv
```

8. Exit qiime

```
conda deactivate
```

8. In R run decontam

8.1. Read the data

```
# Load Libraries
library(decontam)
library(qiime2R)
library(phyloseq)
library(biomformat)

# Make a phyloseq object
ps <- qza_to_phyloseq(features = "table.qza", taxonomy = "taxonomy.qza", metadata = "buzzard_meta.tsv")

# Choose which samples are the negative controls
sample_data(ps)$is.neg <- sample_data(ps)$type == "negative"
```

8.2. Run decontam

```
# Identify contaminants based on prevalence method (treshold 0.1 is the standard)
contamdf.prev <- isContaminant(ps, method="prevalence", neg="is.neg", threshold=0.1)

table(contamdf.prev$contaminant)
head(which(contamdf.prev$contaminant))

# Remove contaminants from the phyloseq object
ps.nocontam <- prune_taxa(!contamdf.prev$contaminant,ps)
```

8.3. Export feature table as biom file

```
# Extract asv table from the phyloseq object
table_nocontam <- as(otu_table(ps.nocontam),"matrix",)

#'t' to transform if taxa_are_rows=FALSE table_nocontam<- t(as(otu_table(ps.nocontam),"matrix",))#if
taxa_are_rows=TRUE

# Make a biom table
table_nocontam_biom <- make_biom(data=table_nocontam)
write_biom(table_nocontam_biom,"table-nocontam.biom")
```

9. Import biom table from R to qiime2

```
conda activate qiime2-2022.11

qiime tools import --input-path table-nocontam.biom --type 'FeatureTable[Frequency]' --input-format
BIOMV100Format --output-path table-nocontam.qza
```

10. Remove control samples from dataset

```
qiime feature-table filter-samples --i-table table-nocontam.qza --m-metadata-file buzzard_meta.tsv --p-where
"type='positive'" --p-exclude-ids --o-filtered-table table-nocontam.qza

qiime feature-table filter-samples --i-table table-nocontam.qza --m-metadata-file buzzard_meta.tsv --p-where
"type='negative'" --p-exclude-ids --o-filtered-table table-nocontam.qza
```

10.1 Table summary after decontam

	Sample
**Number of samples**	235
**Number of features (ASVs)**	13,925
**Total frequency**	2,499,109

11. Taxonomy based filtering

Filter out mitochondrial, Chloroplast, Unassigned, Vertebrata, Bacteria and taxa not assigned to phylum

```
qiime taxa filter-table --i-table table-nocontam.qza --i-taxonomy taxonomy.qza --p-exclude
mitochondria,chloroplast,Unassigned,Vertebrata --p-include p_ --o-filtered-table table-taxa-filter.qza

qiime feature-table summarize --i-table table-taxa-filter.qza --o-visualization table-taxa-filter.qzv --m-
sample-metadata-file buzzard_meta.tsv
```

11.1. Table summary after taxonomic filtering

	Sample
**Number of samples**	217
**Number of features (ASVs)**	10,970
**Total frequency**	1,738,094

12. Filter unique features

```
qiime feature-table filter-features --i-table table-taxa-filter.qza --p-min-samples 2 --o-filtered-table table-
taxa-filter-no_singles.qza

qiime feature-table summarize --i-table table-taxa-filter-no_singles.qza --o-visualization table-taxa-filter-
no_singles.qzv --m-sample-metadata-file buzzard_meta.tsv
```

13. Filter samples with less than 500 reads

```
qiime feature-table filter-samples --i-table table-taxa-filter-no_singles.qza --p-min-frequency 500 --o-
filtered-table table-taxa-filter-no_singles.qza

qiime feature-table summarize --i-table table-taxa-filter-no_singles.qza --o-visualization table-taxa-filter-
no_singles.qzv --m-sample-metadata-file buzzard_meta.tsv
```

13.1 Table summary after filtering unique features and samples with less than 500 reads

	Sample
**Number of samples**	191
**Number of features (ASVs)**	1,770
**Total frequency**	1,494,767

14. Filter representative sequences

```
qiime feature-table filter-seqs --i-data rep-seqs.qza --i-table filtered-table.qza --o-filtered-data filter-seqs.qza
qiime feature-table tabulate-seqs --i-data filter-seqs.qza --o-visualization filter-seqs.qzv
```

15. Build a phylogenetic tree

```
qiime phylogeny align-to-tree-mafft-fasttree --i-sequences filter-seqs.qza  --o-alignment aligned-seqs.qza --o-masked-alignment masked-aligned-seqs.qza --o-tree unrooted-tree.qza  --o-rooted-tree rooted-tree.qza
```

16. Rarefaction curves

```
qiime diversity alpha-rarefaction --i-table filtered-table.qza --i-phylogeny rooted-tree.qza --p-max-depth 20000 --m-metadata-file buzzard_meta.tsv  --o-visualization alpha-rarefaction.qzv

qiime diversity alpha-rarefaction --i-table filtered-table.qza --i-phylogeny rooted-tree.qza --p-max-depth 2000 --m-metadata-file buzzard_meta.tsv  --o-visualization alpha-rarefaction.qzv
```

16.1 Rarefaction plots (sampling depth 20,000)

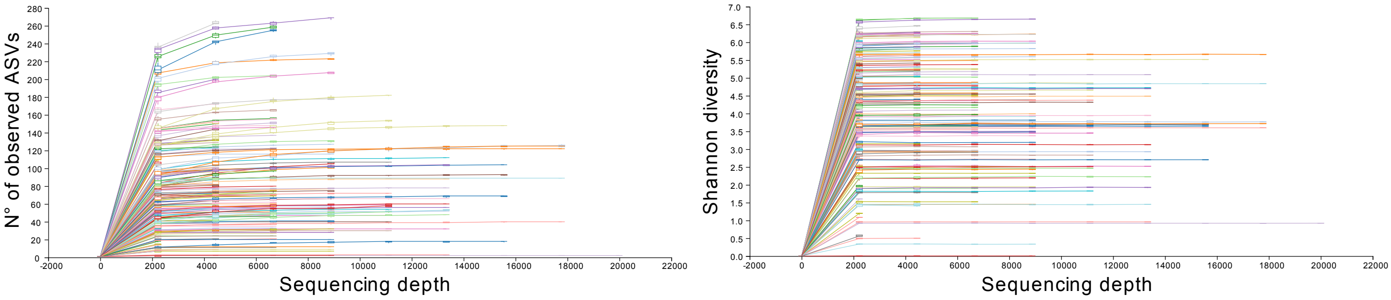

16.2 Rarefaction plots (sampling depth 2000)

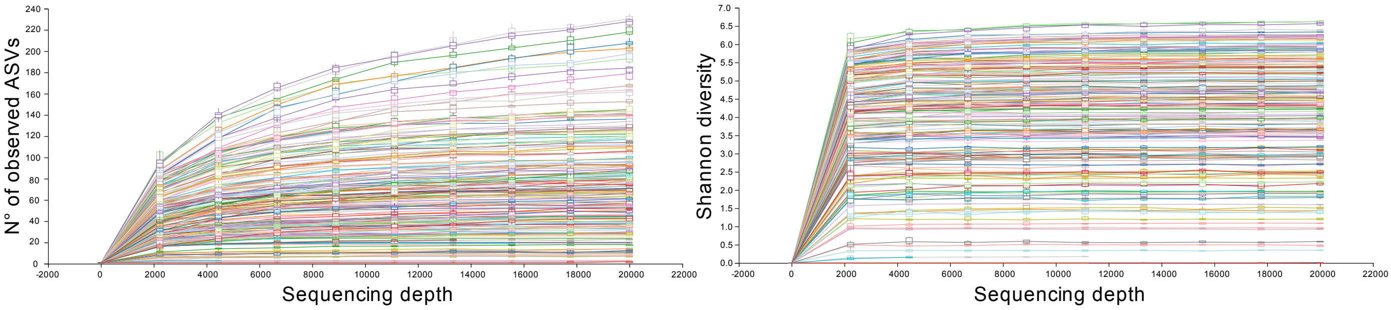

17. Taxa bar plots

```
qiime taxa barplot --i-table filtered-table.qza --i-taxonomy taxonomy.qza --m-metadata-file buzzard_meta.tsv --o-visualization taxa-bar-plots.qzv
```

17.1. Plot core phylum (present in 70% of samples)

```
qiime taxa collapse --i-table filtered-table.qza --i-taxonomy taxonomy.qza --o-collapsed-table phylum-table.qza
 --p-level 2

qiime feature-table summarize --i-table phylum-table.qza --o-visualization phylum-table.qzv --m-sample-metadata-file buzzard_meta.tsv

qiime feature-table core-features --i-table phylum-table.qza --o-visualization phylum-core-table.qzv
```

From the visualization file download the tsv relative to features present in 70% of the samples (or any other % that I want)

```
qiime metadata tabulate --m-input-file core-features-0.700.tsv --o-visualization core-features-0.700.qzv

qiime feature-table filter-features --i-table filtered-table.qza --o-filtered-table table-phylum-core-0.700.qza
--m-metadata-file core-features-0.700.tsv

qiime taxa barplot --i-table table-phylum-core-0.700.qza --i-taxonomy taxonomy.qza --m-metadata-file
buzzard_meta.tsv --o-visualization core-phylum-bar-plots.qzv
```

Feature ID	2%	9%	25%	50%	75%	91%	98%
dEukaryota;pAscomycota	194.02	1249.7	2510	3538.5	5763.75	10004.59	13126.14
dEukaryota;pBasidiomycota	19.08	250.31	771.5	1338.5	2443	5386.57	8451.72
dEukaryota;pPhragmoplastophyta	0	0	1.5	168	549	1439.67	4006.36

17.2. Plot core families (present in 70% of samples)

```
qiime taxa collapse --i-table filtered-table.qza --i-taxonomy taxonomy.qza --o-collapsed-table family-table.qza
 --p-level 5

qiime feature-table summarize --i-table -table.qza --o-visualization family-table.qzv --m-sample-metadata-file
buzzard_meta.tsv

qiime feature-table core-features --i-table family-table.qza --o-visualization family-core-table.qzv
```

From the visualization file download the tsv relative to features present in 70% of the samples (or any other % that I want)

```
qiime metadata tabulate --m-input-file core-features-0.700.tsv --o-visualization core-features-0.700.qzv

qiime feature-table filter-features --i-table filtered-table.qza --o-filtered-table table-family-core-0.700.qza
--m-metadata-file core-features-0.700.tsv

qiime taxa barplot --i-table table-family-core-0.700.qza --i-taxonomy taxonomy.qza --m-metadata-file
buzzard_meta.tsv --o-visualization core-family-bar-plots.qzv
```

Feature ID	2%	9%	25%	50%	75%	91%	98%
dEukaryota;pBasidiomycota;cExobasidiomycetes;oExobasidiomycetes;f__Exobasidiomycetes	0	105.44	293	775	1621.5	3861.9	7110.5
dEukaryota;pAscomycota;cDothideomycetes;oCapnodiales;f__Capnodiales	0	17.66	220	457	797	1134.9	1368.08
dEukaryota;pAscomycota;cDothideomycetes;oCapnodiales;__	0	14.44	111.5	236.5	520.25	1088.58	2432.76
dEukaryota;pAscomycota;cDothideomycetes;oCapnodiales;f__Cladosporiaceae	0	0	29.75	161	466.25	1123.67	3475.32
dEukaryota;pAscomycota;;;__	0	0	10.75	144	354.5	587.78	1235.62
dEukaryota;pPhragmoplastophyta;cPhragmoplastophyta;oPhragmoplastophyta;f__Phragmoplastophyta	0	0	0	106.5	416.75	1247.84	3978.52

18. Calculate alpha diversity metrics and rarefy the data-set

```
# Calculates observed features, shannon diversity, Faith PD and retrieves rarefied table
qiime diversity core-metrics-phylogenetic --i-phylogeny rooted-tree.qza --i-table filtered-table.qza --p-sampling-depth 2000 --m-metadata-file buzzard_meta.tsv --output-dir alpha-metrics-results

#Add alpha diversity metrics to the metadata
qiime metadata tabulate --m-input-file buzzard_metadata.tsv --m-input-file shannon_vector.qza --m-input-file observed_features.qza --m-input-file faith_pd_vector.qza --o-visualization buzzard_meta_alpha.qzv
```

From the "buzzard_meta_alpha.qzv" one can extract the tsv file with the metadata + alpha metrics.

19. Filter samples with less than 2000 reads (unrarefied table for beta analysis)

```
qiime feature-table filter-samples --i-table filtered-table.qza --p-min-frequency 4000 --o-filtered-table beta-table.qza

qiime feature-table summarize --i-table beta-table.qza --o-visualization beta-table.qzv --m-sample-metadata-file buzzard_meta_alpha.tsv
```

19.1. Table Summary

	Sample
**Number of samples**	180
**Number of features (ASVs)**	1,770
**Total frequency**	1,478,279

19.2. Summary of final reads per sample

	Frequency
**Minimum frequency**	2,036.00
**1st quartile**	5,388.25
**Median frequency**	7,138.50
**3rd quartile**	9,985.75
**Maximum frequency**	33,249.00
**Mean frequency**	8,212.66

24.2. Sequence length statistics

Sequence Count	Min Length	Max Length	Mean Length	Range	Standard Deviation
1770	230	415	413.84	185	11.08
