# Supplementary material for: Early-life factors shaping the gut microbiota of Common buzzard nestlings
Source: Anim Microbiome. 2024 May 14;6:27. doi: 10.1186/s42523-024-00313-8 (PMC11092241; doi:10.1186/s42523-024-00313-8)
Supplement: Supplementary file 5 — Supplementary file1 (PDF 1408 kb) [file 42523_2024_313_MOESM5_ESM.pdf]

Alpha diversity statistical analysis

Table of Contents

Alpha diversity statistical analysis

Table of Contents

A) 16S rRNA alpha diversity statistical analysis

1. Shannon diversity Index
   - 1.1 Check correlation between variables
   - 1.2 Model Shannon
   - 1.3 Check Normality
   - 1.4 Model Diagnostics
   - 1.5 Model Summary
   - 1.6 Significance values
   - 1.7 Marginal and conditional R-squared
   - 1.8 Plot model effects
   - 1.9 Significance of random effects
2. Faith phylogenetic diversity
   - 2.1 Log transform Faith
   - 2.2 Check correlation between variables
   - 2.3 Model Faith PD
   - 2.4 Check Normality
   - 2.5 Model Diagnostics
   - 2.6 Model Summary
   - 2.7 Significance values
   - 2.8 Marginal and Conditional R-squared
   - 2.9 Plot model effects
   - 2.10 Significance of random effects

B) 28S rRNA alpha diversity statistical analysis

1. Shannon diversity Index
   - 1.1 Check correlation between variables
   - 1.2. Transform Shannon
   - 1.3. Model Faith PD
   - 1.4. Check Normality
   - 1.5. Model Diagnostics
   - 1.6. Model Summary
   - 1.7. Significance values
   - 1.8. Marginal and conditional R-squared
   - 1.9. Plot model effects
   - 1.10. Significance of random effects
2. Faith phylogenetic diversity
   - 2.1 Check correlation between variables
   - 2.2. Log transform Faith
   - 2.2. Model Faith PD
   - 2.3. Check Normality
   - 2.4. Model Diagnostics
   - 2.5. Model Summary
   - 2.6. Significance values
     - 2.6.1. Multiple comparison test for "Habitat"
   - 2.7. Marginal and Conditional R-squared
   - 2.8. Plot model effects
   - 2.9. Significance of random effects

A) 16S rRNA alpha diversity statistical analysis

1. Shannon diversity Index

```
#Load libraries
library(tidyverse)
library(lme4)
library(MuMIn)
library (performance)
library(datawizard)
library(car)
library(effects)
library(ggpubr)
library(jtools)
library(correlation)
library(lmerTest)

#Load dataset
metadata <- readRDS("16S_metadata.rds")

# Calculate age and body condition index
metadata$bci_two<-resid(glm(weight~ log10(wing) + sex, gaussian, metadata, na.action="na.exclude")) #calculate
body condition
metadata$std_bci <- scale(metadata$bci_two) # scale bci values

metadata$age_days <- buteo_age(df = metadata, wing = "wing", sex = TRUE, unit = c("cm"), .plot = F, decimals =
2,.show_model = T)$fit
metadata$std_age <- scale(metadata$age_days) # scale age values

saveRDS(metadata,"16S_metadata.rds")
```

1.1 Check correlation between variables

```
#check correlation between variables from the model
test_cor_data <- metadata[, c("habitat", "rank", "year", "lbinom", "sex", "std_age", "std_bci_two",
"shannon_entropy")]

correl <- correlation(test_cor_data, include_factors = TRUE)
correl <- cor_sort(as.matrix(correl)) # as matrix

#Plot matrix
corr_matrix <- visualisation_recipe(correl)
plot(corr_matrix)
```

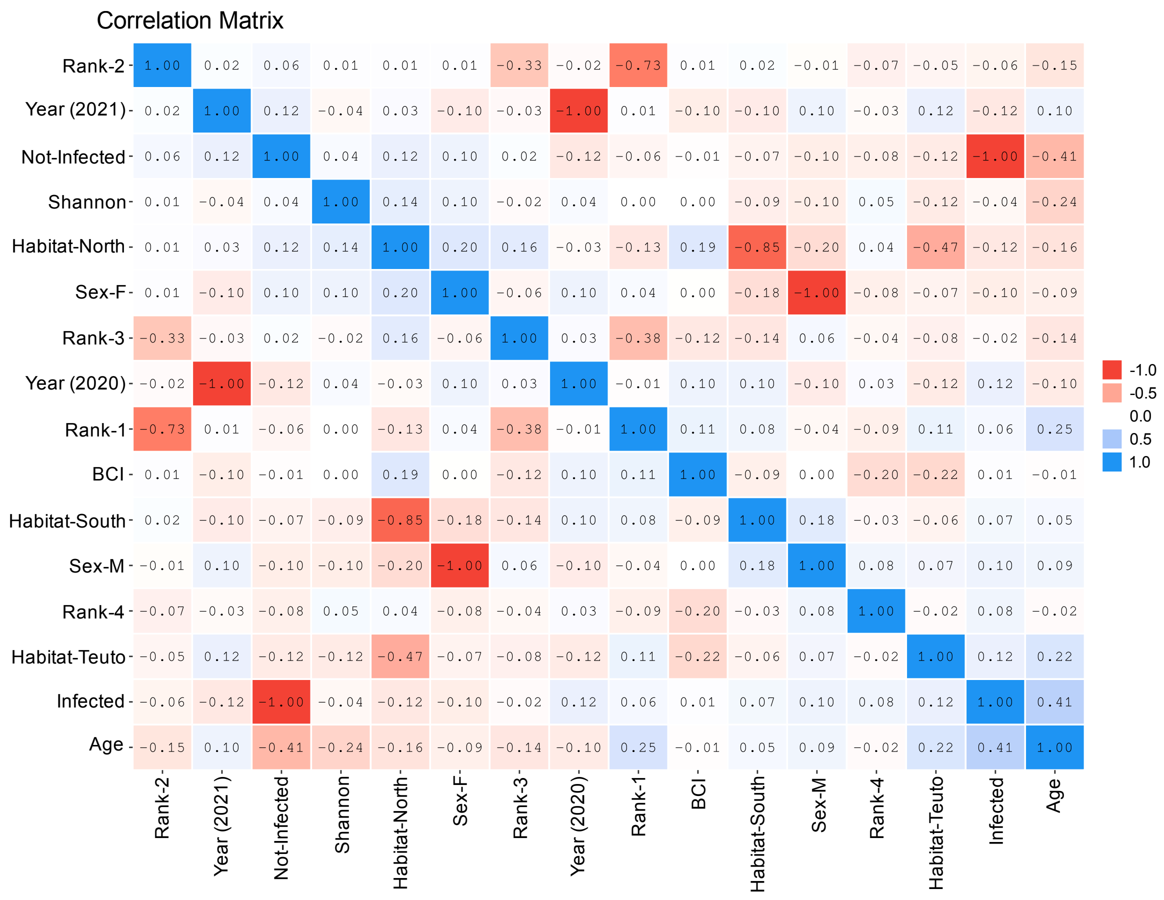

1.2 Model Shannon

```
model_shannon <- lmer(shannon_entropy ~ std_age + std_bci + rank + sex + year + habitat + lbinom +
(1|nest/ring_number), data = metadata)
```

1.3 Check Normality

```
> check_normality(model_shannon)
OK: residuals appear as normally distributed (p = 0.394).
```

1.4 Model Diagnostics

```
check_model(model_shannon)
```

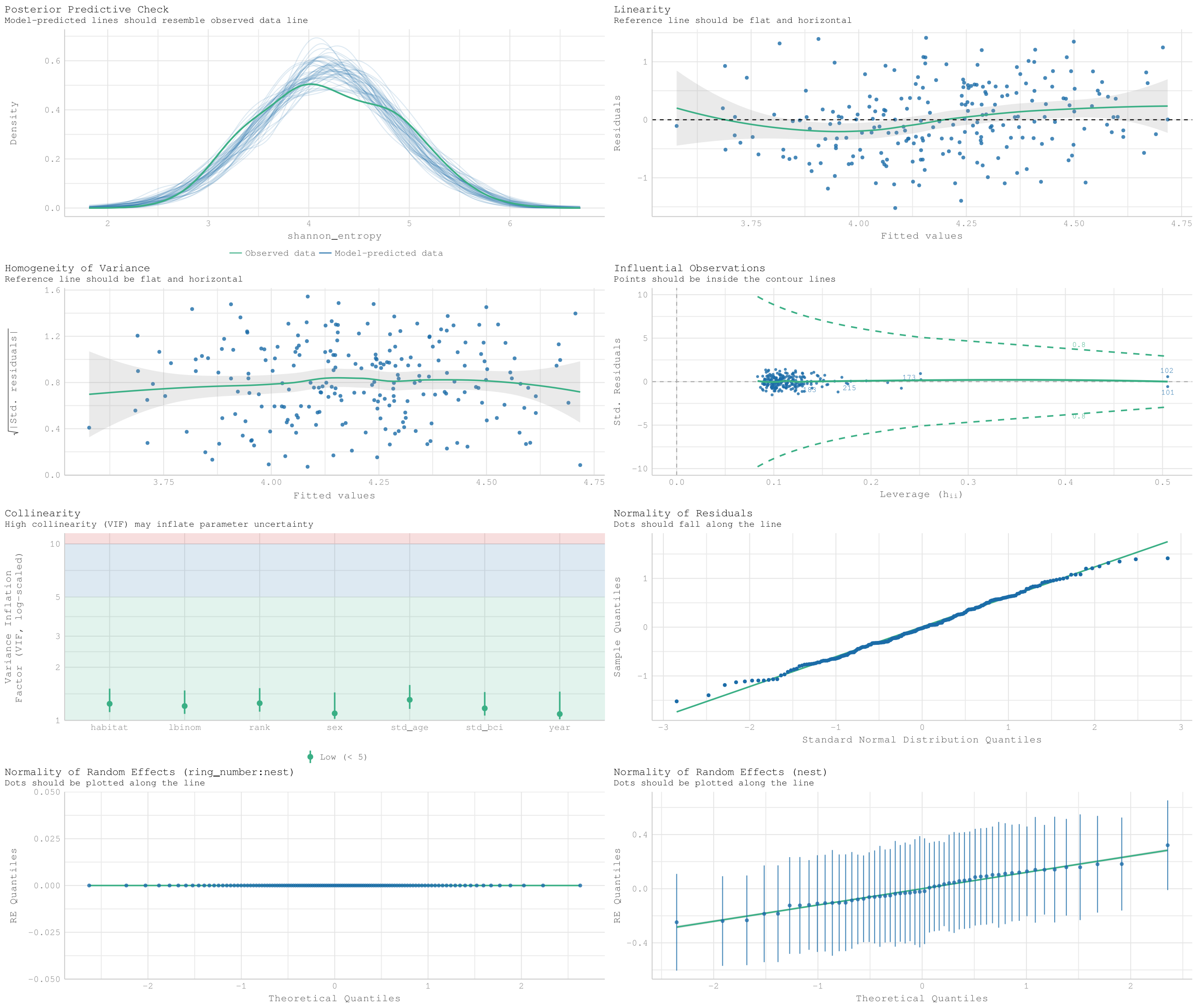

1.5 Model Summary

```
> summary(model_shannon_final)

Linear mixed model fit by REML ['lmerMod']
Formula: shannon_entropy ~ std_age + std_bci + rank + sex + year + habitat + lbinom + (1 | nest/ring_number)
   Data: metadata

REML criterion at convergence: 475.7

Scaled residuals:
     Min       1Q     Median       3Q      Max
-2.38433  -0.63636  -0.03356  0.65990  2.21304

Random effects:
 Groups           Name        Variance  Std.Dev.
 ring_number:nest (Intercept) 0.00000   0.0000
 nest             (Intercept) 0.04932   0.2221
 Residual                     0.40710   0.6380
Number of obs: 226, groups:  ring_number:nest, 117; nest, 54

Fixed effects:
               Estimate   Std. Error   t value
(Intercept)   4.3068106   0.1058171    40.701
std_age      -0.1652697   0.0529112    -3.124
std_bci       0.0001679   0.0511950     0.003
```

```
rank2        -0.0925053     0.0964370     -0.959
rank3        -0.1613338     0.1423461     -1.133
rank4         0.2139437     0.5077520      0.421
sexM         -0.1144732     0.0963586     -1.188
year2021      0.0683771     0.1981118      0.345
habitatsouth -0.1657539     0.1744792     -0.950
habitatteuto -0.2976098     0.2869742     -1.037
lbinom1       0.0787340     0.1036844      0.759

Correlation of Fixed Effects:
            (Intr) std_ag std_b_ rank2  rank3  rank4  sexM   yr2021 hbttst hbtttt
std_age      0.143
std_bci     -0.087  0.023
rank2       -0.404  0.196  0.101
rank3       -0.246  0.216  0.210  0.378
rank4       -0.025  0.112  0.240  0.132  0.167
sexM        -0.388 -0.043 -0.088 -0.035 -0.146 -0.138
year2021    -0.177 -0.106  0.100 -0.028  0.029  0.041 -0.138
habitatsoth -0.143 -0.013  0.156  0.048  0.168  0.094 -0.207  0.134
habitatteut -0.087 -0.137  0.239  0.074  0.114  0.077 -0.082 -0.086  0.124
lbinom1     -0.521 -0.371 -0.036 -0.032 -0.079 -0.082 -0.043  0.142 -0.038 -0.065
optimizer (nloptwrap) convergence code: 0 (OK)
boundary (singular) fit: see help('isSingular')
```

1.6 Significance values

```
> Anova(model_shannon)

Analysis of Deviance Table (Type II Wald chisquare tests)

Response: shannon_entropy
             Chisq    Df    Pr(>Chisq)
std_age      9.7564    1     0.001787 **
std_bci      0.0000    1     0.997383
rank         2.0552    3     0.561028
sex          1.4113    1     0.234836
year         0.1191    1     0.729986
habitat      1.7600    2     0.414783
lbinom       0.5766    1     0.447636
---
Signif. codes:  0 '***' 0.001 '**' 0.01 '*' 0.05 '.' 0.1 ' ' 1
```

1.7 Marginal and conditional R-squared

```
 r.squaredGLMM(model_shannon)
           R2m         R2c
[1,]   0.0758452    0.1757042
```

1.8 Plot model effects

```
plot(allEffects(model_shannon))
```

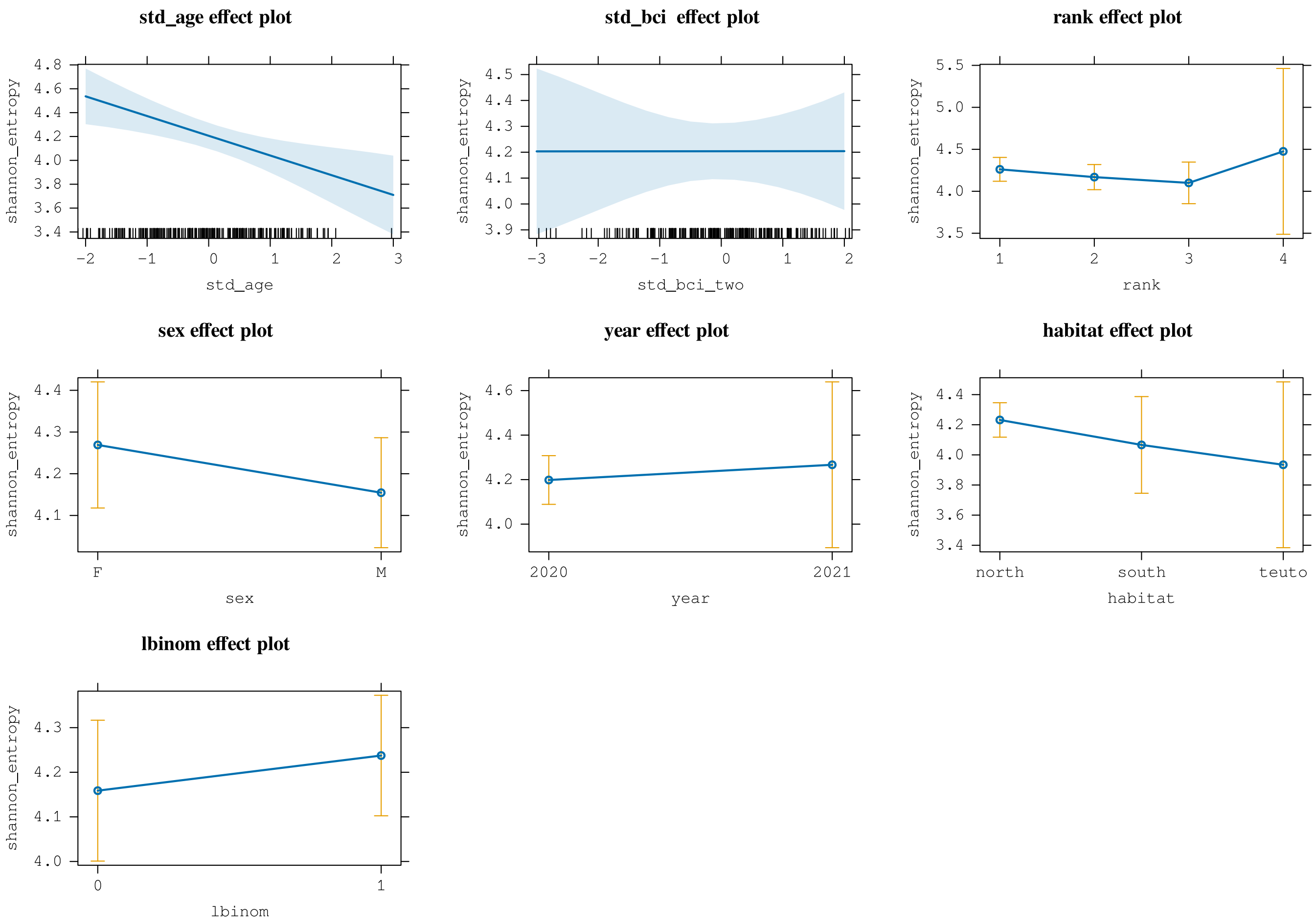

1.9 Significance of random effects

```
>ranova(model_shannon)
ANOVA-like table for random-effects: Single term deletions

Model:
shannon_entropy ~ std_age + std_bci + rank + sex + year + habitat + lbinom + (1 | ring_number:nest) + (1 |
nest)
                       npar  logLik    AIC    LRT Df Pr(>Chisq)
<none>                   14 -237.86 503.71
(1 | ring_number:nest)   13 -237.86 501.71 0.0000  1    1.00000
(1 | nest)               13 -239.56 505.12 3.4089  1    0.06485 .
---
Signif. codes:  0 '***' 0.001 '**' 0.01 '*' 0.05 '.' 0.1 ' ' 1
```

2. Faith phylogenetic diversity

2.1 Log transform Faith

(model residuals not normal distributed)

```
model_faith <- lmer(faith_pd ~ std_age + std_bci + rank + sex + year + habitat + lbinom + (1|nest/ring_number),
data = metadata)

> check_normality(model_faith)
Warning: Non-normality of residuals detected (p = 0.003).

#Log transform faith
metadata$log_faith <- log10(metadata$faith_pd)
```

2.2 Check correlation between variables

```
#check correlation between variables from the model
test_cor_data <- metadata[, c("habitat", "rank", "year", "lbinom", "sex", "std_age", "std_bci_two",
"faith_pd")]

correl <- correlation(test_cor_data, include_factors = TRUE)
correl <- cor_sort(as.matrix(correl)) # as matrix

#Plot matrix
corr_matrix <- visualisation_recipe(correl)
plot(corr_matrix)
```

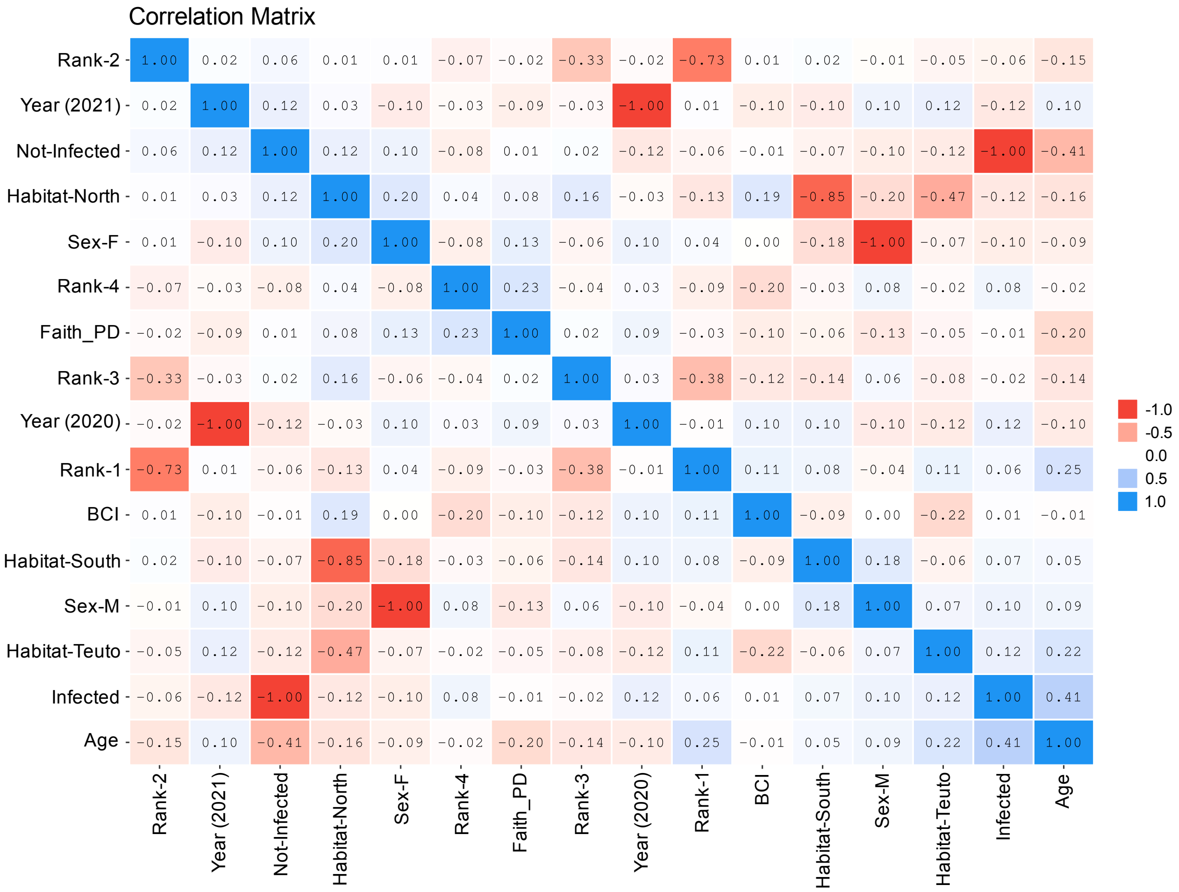

2.3 Model Faith PD

```
model_faith <- lmer(log_faith ~ std_age + std_bci + rank + sex + year + habitat + lbinom +
(1|nest/ring_number), data = metadata)
```

2.4 Check Normality

```
> check_normality(model_faith)
OK: residuals appear as normally distributed (p = 0.081).
```

2.5 Model Diagnostics

```
check_model(model_faith)
```

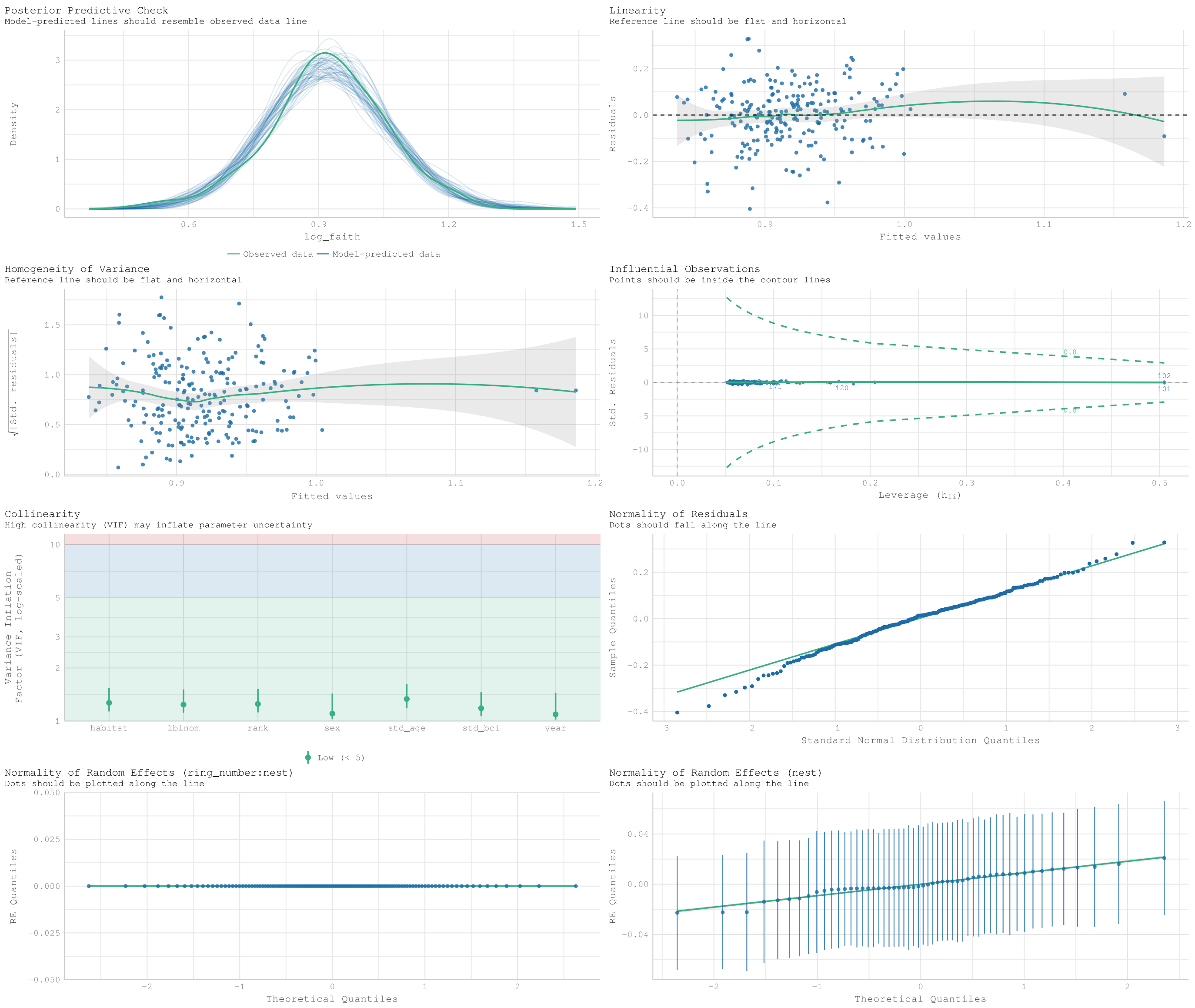

2.6 Model Summary

```
> summary(model_faith)

Linear mixed model fit by REML ['lmerMod']
Formula: log_faith ~ std_age + std_bci + rank + sex + year + habitat + lbinom + (1 | nest/ring_number)
   Data: metadata

REML criterion at convergence: -225.5

Scaled residuals:
    Min      1Q    Median     3Q     Max
-3.1497  -0.5645  0.1041   0.6145  2.5580

Random effects:
 Groups           Name        Variance  Std.Dev.
 ring_number:nest (Intercept) 0.0000000 0.00000
 nest             (Intercept) 0.0006666 0.02582
 Residual                     0.0164990 0.12845
```

```
Number of obs: 226, groups:  ring_number:nest, 117; nest, 54

Fixed effects:
               Estimate   Std. Error    t value
(Intercept)   0.9407717    0.0199731    47.102
std_age      -0.0285771    0.0102204    -2.796
std_bci      -0.0067600    0.0097165    -0.696
rank2        -0.0189343    0.0192559    -0.983
rank3        -0.0154191    0.0279228    -0.552
rank4         0.2347500    0.0988584     2.375
sexM         -0.0360834    0.0186700    -1.933
year2021     -0.0134265    0.0354902    -0.378
habitatsouth -0.0029790    0.0315251    -0.094
habitatteuto  0.0008674    0.0526736     0.016
lbinom1       0.0146748    0.0200224     0.733

Correlation of Fixed Effects:
            (Intr) std_ag std_b_ rank2  rank3  rank4  sexM   yr2021 hbttst hbtttt
std_age      0.164
std_bci     -0.079  0.018
rank2       -0.428  0.188  0.090
rank3       -0.280  0.200  0.210  0.374
rank4       -0.027  0.097  0.246  0.127  0.155
sexM        -0.394 -0.052 -0.097 -0.037 -0.137 -0.129
year2021    -0.166 -0.115  0.105 -0.031  0.035  0.045 -0.148
habitatsoth -0.124 -0.011  0.172  0.049  0.187  0.104 -0.220  0.136
habitatteut -0.086 -0.142  0.251  0.076  0.125  0.087 -0.082 -0.071  0.130
lbinom1     -0.525 -0.394 -0.043 -0.034 -0.070 -0.095 -0.049  0.152 -0.041 -0.063
optimizer (nloptwrap) convergence code: 0 (OK)
boundary (singular) fit: see help('isSingular')
```

2.7 Significance values

```
> Anova(model_faith)
Analysis of Deviance Table (Type II Wald chisquare tests)

Response: log_faith
             Chisq   Df   Pr(>Chisq)
std_age     7.8181   1     0.005173 **
std_bci     0.4840   1     0.486605
rank        7.5620   3     0.055986 .
sex         3.7353   1     0.053274 .
year        0.1431   1     0.705197
habitat     0.0098   2     0.995125
lbinom      0.5372   1     0.463609
---
Signif. codes:  0 '***' 0.001 '**' 0.01 '*' 0.05 '.' 0.1 ' ' 1
```

2.8 Marginal and Conditional R-squared

```
> r.squaredGLMM(model_faith)
            R2m        R2c
[1,]   0.09268855   0.1279241
```

2.9 Plot model effects

```
plot(allEffects(model_faith))
```

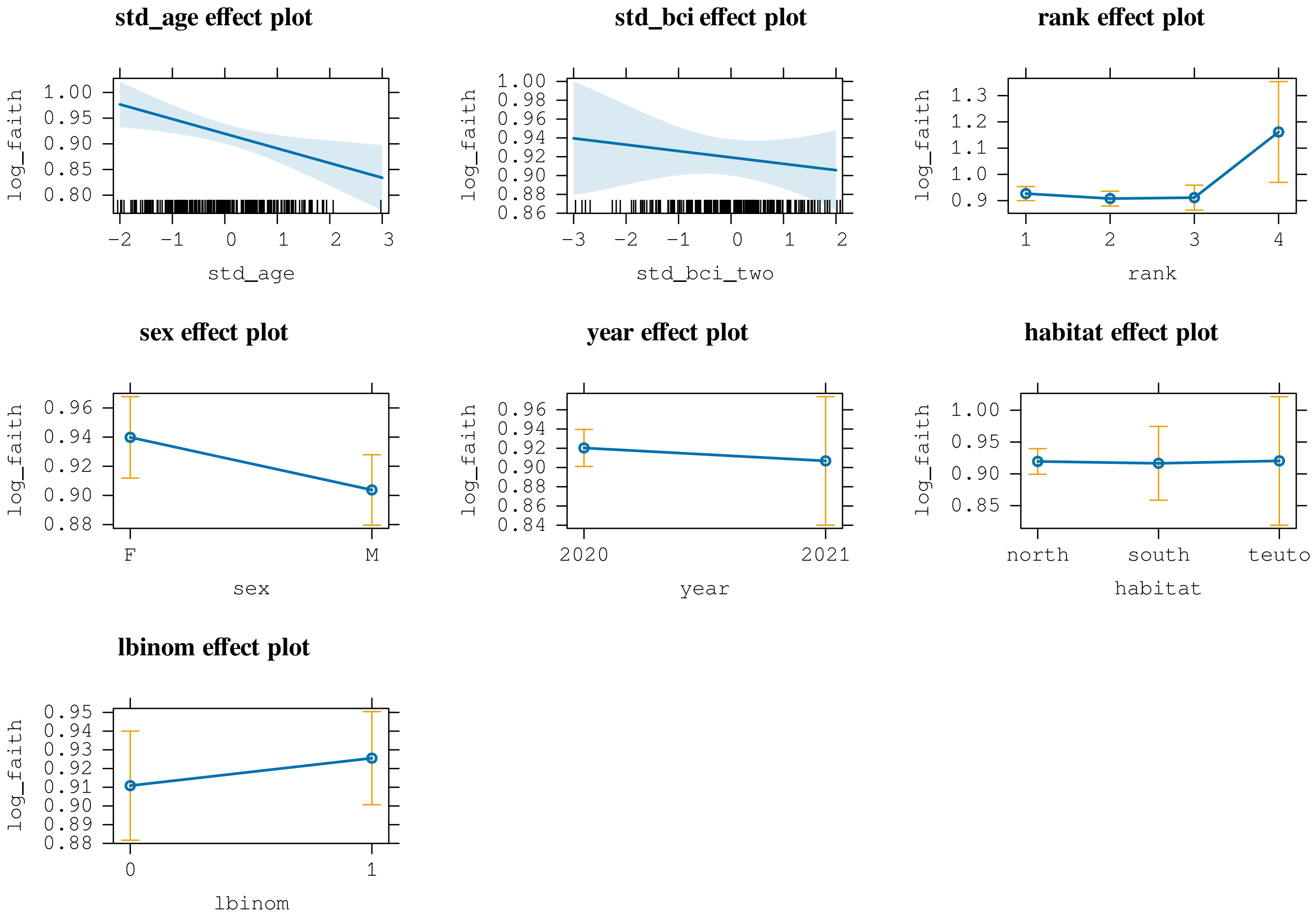

2.10 Significance of random effects

```
> ranova(model_faith)
boundary (singular) fit: see help('isSingular')
ANOVA-like table for random-effects: Single term deletions

Model:
log_faith ~ std_age + std_bci + rank + sex + year + habitat + lbinom + (1 | ring_number:nest) + (1 | nest)
                       npar logLik     AIC     LRT Df Pr(>Chisq)
<none>                   14 112.74 -197.49
(1 | ring_number:nest)   13 112.74 -199.49 0.00000  1     1.0000
(1 | nest)               13 112.45 -198.91 0.58057  1     0.4461
```

B) 28S rRNA alpha diversity statistical analysis

1. Shannon diversity Index

```
#Load libraries
library(tidyverse)
library(lme4)
library(MuMIn)
library (performance)
library(datawizard)
library(car)
library(effects)
library(ggpubr)
library(multcomp)
library(jtools)

#Load dataset
metadata <- readRDS("28S_metadata.rds")

# Calculate age and body condition index
metadata$bci_two<-resid(glm(weight~ log10(wing) + sex, gaussian, metadata, na.action="na.exclude")) #calculate
body condition
metadata$std_bci <- scale(metadata$bci_two) # scale bci values

metadata$age_days <- buteo_age(df = metadata, wing = "wing", sex = TRUE, unit = c("cm"), .plot = F, decimals =
2,.show_model = T)$fit
metadata$std_age <- scale(metadata$age_days) # scale age values

saveRDS(metadata,"28S_metadata.rds")
```

1.1 Check correlation between variables

```
#check correlation between variables from the model
test_cor_data <- metadata[, c("habitat", "rank", "year", "lbinom", "sex", "std_age", "std_bci_two",
"shannon_entropy")]

correl <- correlation(test_cor_data, include_factors = TRUE)
correl <- cor_sort(as.matrix(correl)) # as matrix

#Plot matrix
corr_matrix <- visualisation_recipe(correl)
plot(corr_matrix)
```

1.2. Transform Shannon

```
> model_shannon <- lmer(shannon_entropy ~ std_age + std_bci + rank + sex + year + habitat + lbinom +
(1|nest/ring_number), data = metadata)

> check_normality(model_shannon)
Warning: Non-normality of residuals detected (p < .001).

# Reflect and log transform shannon
metadata$log_shannon <- log10(max(metadata$shannon_entropy+1) - metadata$shannon_entropy)

# Data reflection changes direction of relationships
metadata$log_shannon <- -metadata$log_shannon # change directions of relatioships again
```

1.3. Model Faith PD

```
model_shannon <- lmer(log_shannon ~ std_age + std_bci + rank + sex + year + habitat + lbinom +
(1|nest/ring_number), data = metadata)
```

1.4. Check Normality

```
> check_normality(model_shannon_final_prev)
Warning: Non-normality of residuals detected (p = 0.046). # residuals still not normal distributed
```

1.5. Model Diagnostics

```
check_model(model_shannon)# normality of residuals identified by visual inspection
```

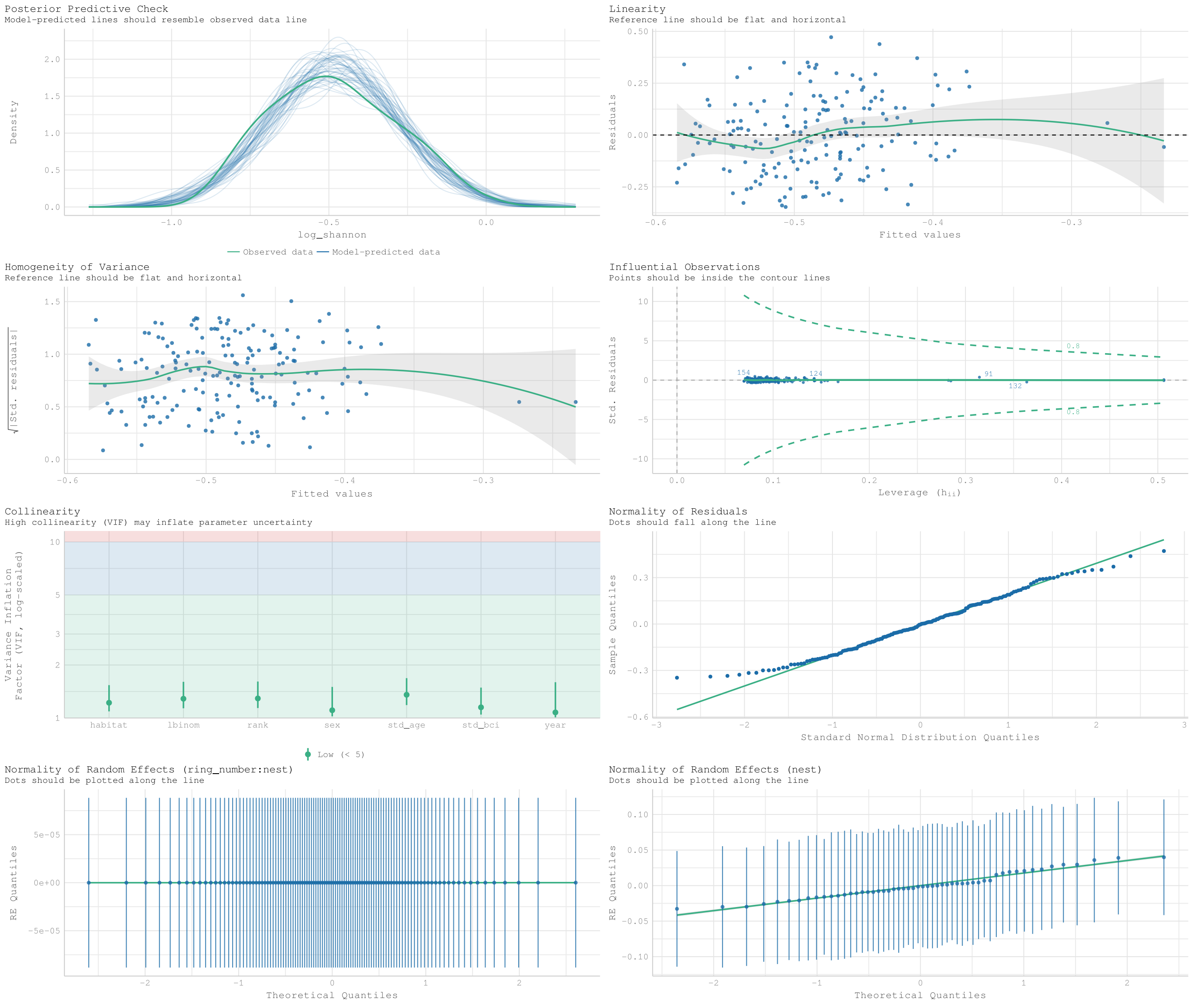

1.6. Model Summary

```
> summary(model_shannon)
Linear mixed model fit by REML ['lmerMod']
Formula: log_shannon ~ std_age + std_bci + sex + rank + habitat + year + lbinom + (1 | nest/ring_number)
   Data: metadata

REML criterion at convergence: -28.9

Scaled residuals:
    Min      1Q    Median      3Q      Max
-1.7910  -0.7103  -0.0168   0.6698   2.4352

Random effects:
 Groups           Name        Variance  Std.Dev.
 ring_number:nest (Intercept) 2.032e-09 4.508e-05
 nest             (Intercept) 2.236e-03 4.729e-02
 Residual                     3.754e-02 1.938e-01
Number of obs: 177, groups:  ring_number:nest, 108; nest, 54

Fixed effects:
              Estimate   Std. Error   t value
(Intercept)  -0.520591    0.034071    -15.280
std_age      -0.035594    0.017628    -2.019
std_bci      -0.004052    0.016615    -0.244
sexM          0.016542    0.032398     0.511
rank2        -0.004258    0.033979    -0.125
rank3         0.029871    0.045397     0.658
rank4         0.210610    0.152755     1.379
habitatsouth  0.068654    0.053602     1.281
habitatteuto  0.112459    0.108764     1.034
year2021      0.003454    0.061376     0.056
lbinom1       0.019645    0.034963     0.562

Correlation of Fixed Effects:
            (Intr) std_ag std_b_ sexM   rank2  rank3  rank4  hbttst hbtttt yr2021
std_age      0.186
std_bci     -0.023  0.002
sexM        -0.408 -0.103 -0.112
rank2       -0.426  0.143  0.086 -0.062
rank3       -0.265  0.231  0.206 -0.101  0.372
rank4       -0.014  0.114  0.251 -0.136  0.123  0.178
habitatsoth -0.111  0.047  0.138 -0.164  0.026  0.183  0.106
habitatteut -0.123 -0.151  0.184  0.036  0.123  0.113  0.066  0.078
year2021    -0.109 -0.060  0.095 -0.155 -0.078  0.009  0.049  0.125 -0.091
lbinom1     -0.504 -0.398 -0.024 -0.099  0.032 -0.120 -0.105 -0.079 -0.070  0.107
```

1.7. Significance values

```
> Anova(model_shannon)
Analysis of Deviance Table (Type II Wald chisquare tests)

Response: log_shannon
            Chisq  Df  Pr(>Chisq)
std_age     4.0769  1    0.04347 *
std_bci     0.0595  1    0.80735
sex         0.2607  1    0.60965
rank        2.3064  3    0.51129
habitat     2.5173  2    0.28404
year        0.0032  1    0.95512
lbinom      0.3157  1    0.57421
---
```

```
Signif. codes:  0 '***' 0.001 '**' 0.01 '*' 0.05 '.' 0.1 ' ' 1
```

1.8. Marginal and conditional R-squared

```
> r.squaredGLMM(model_shannon)
            R2m          R2c
[1,]    0.05739074    0.1103858
```

1.9. Plot model effects

```
plot(allEffects(model_shannon))
```

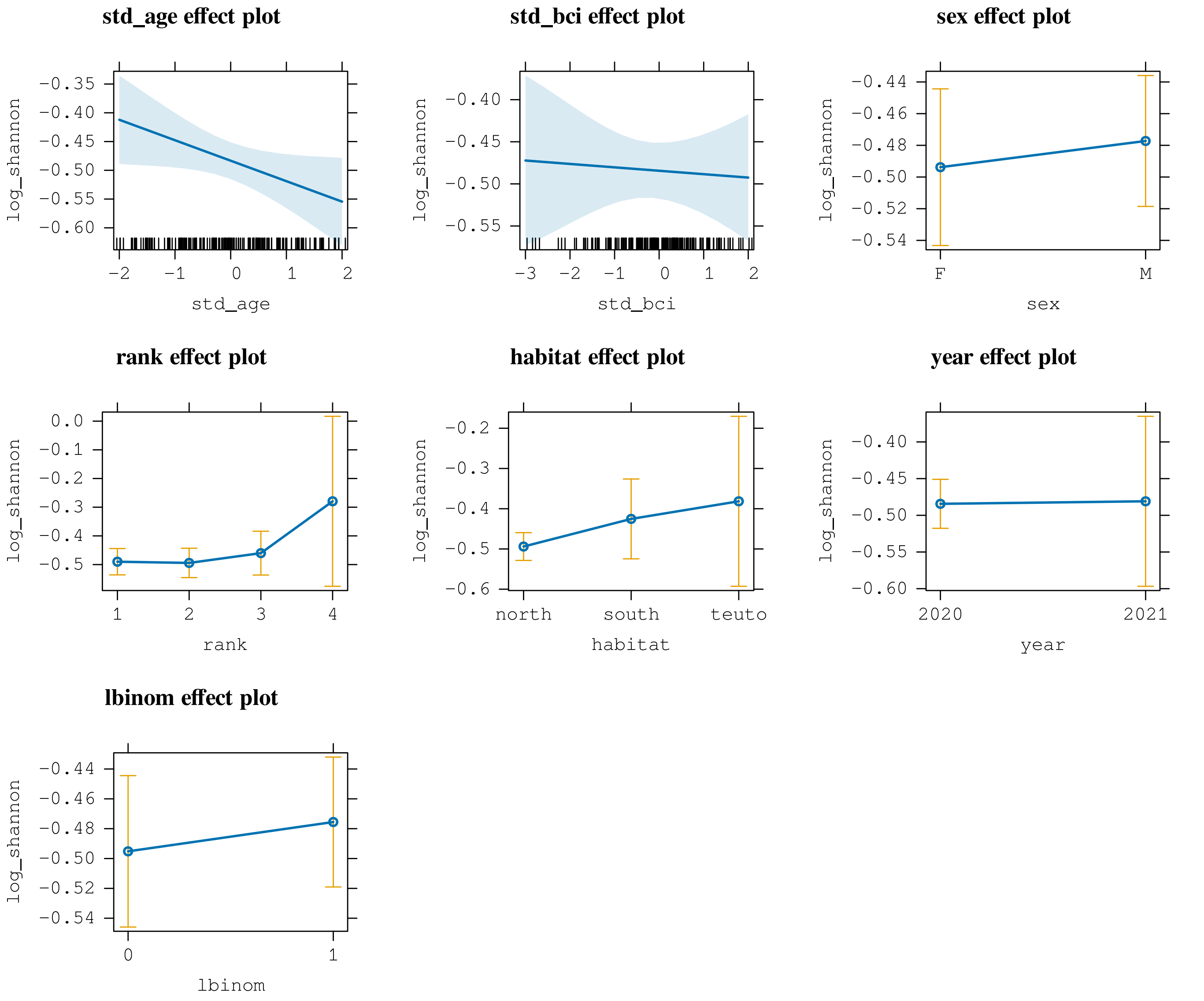

1.10. Significance of random effects

```
> ranova(model_shannon)
boundary (singular) fit: see help('isSingular')
ANOVA-like table for random-effects: Single term deletions

Model:
log_shannon ~ std_age + std_bci + sex + rank + habitat + year + lbinom + (1 | ring_number:nest) + (1 | nest)
                       npar logLik      AIC     LRT Df Pr(>Chisq)
<none>                   14 14.434 -0.86813
(1 | ring_number:nest)   13 14.434 -2.86813 0.00000  1     1.0000
(1 | nest)               13 14.145 -2.28934 0.57879  1     0.4468
```

2. Faith phylogenetic diversity

2.1 Check correlation between variables

```
#check correlation between variables from the model
test_cor_data <- metadata[, c("habitat", "rank", "year", "lbinom", "sex", "std_age", "std_bci_two",
"faith_pd")]

correl <- correlation(test_cor_data, include_factors = TRUE)
correl <- cor_sort(as.matrix(correl)) # as matrix

#Plot matrix
corr_matrix <- visualisation_recipe(correl)
plot(corr_matrix)
```

2.2. Log transform Faith

(model residuals not normal distributed)

```
model_faith <- lmer(faith_pd ~ std_age + std_bci + rank + sex + year + habitat + lbinom + (1|nest/ring_number),
data = metadata)

> check_normality(model_faith_final_lbinom)
Warning: Non-normality of residuals detected (p < .001).

#Log transform faith
metadata$log_faith <- log10(metadata$faith_pd)
```

2.2. Model Faith PD

```
model_faith <- lmer(log_faith ~ std_age + std_bci + rank + sex + year + habitat + lbinom +
(1|nest/ring_number), data = metadata)
```

2.3. Check Normality

```
> check_normality(model_faith)
OK: residuals appear as normally distributed (p = 0.661).
```

2.4. Model Diagnostics

```
check_model(model_faith)
```

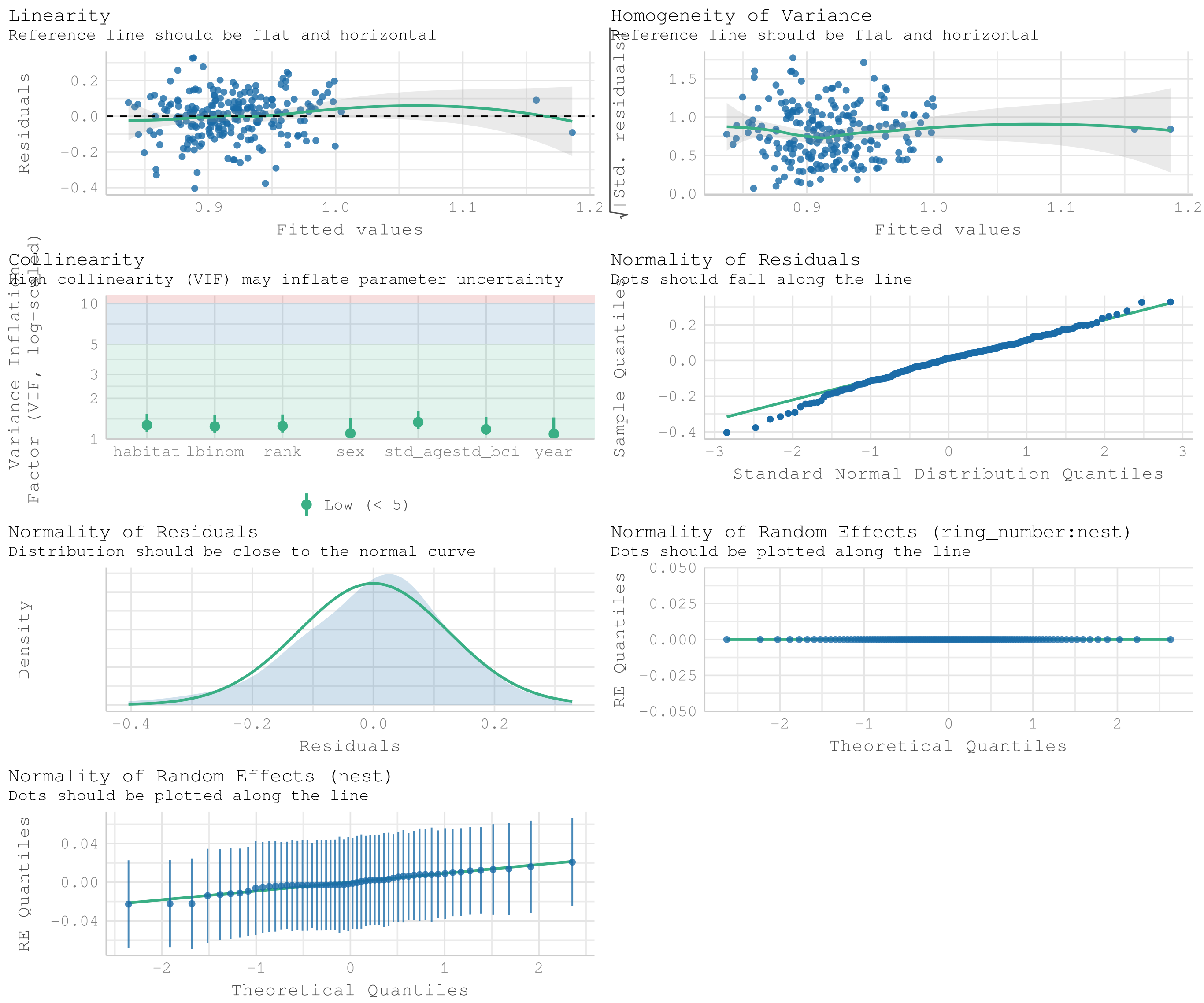

2.5. Model Summary

```
> summary(model_faith)

Linear mixed model fit by REML ['lmerMod']
Formula: log_faith ~ std_age + std_bci + rank + sex + year + habitat +      lbinom + (1 | nest/ring_number)
   Data: metadata

REML criterion at convergence: -10

Scaled residuals:
     Min       1Q    Median      3Q      Max
-2.62275  -0.65997  0.01234  0.63677  2.32240
```

```
Random effects:
 Groups           Name        Variance Std.Dev.
 ring_number:nest (Intercept) 0.00000  0.0000
 nest             (Intercept) 0.00000  0.0000
 Residual                     0.04422  0.2103
Number of obs: 177, groups:  ring_number:nest, 108; nest, 54

Fixed effects:
              Estimate  Std. Error    t value
(Intercept)   0.403635   0.035173     11.476
std_age      -0.020558   0.018479     -1.113
std_bci      -0.019744   0.017132     -1.152
rank2         0.007209   0.036431      0.198
rank3        -0.014333   0.048106     -0.298
rank4         0.315343   0.158692      1.987
sexM          0.008349   0.033891      0.246
year2021      0.004901   0.060742      0.081
habitatsouth  0.094678   0.053631      1.765
habitatteuto  0.219712   0.113001      1.944
lbinom1       0.043000   0.036659      1.173

Correlation of Fixed Effects:
            (Intr) std_ag std_b_ rank2  rank3  rank4  sexM   yr2021 hbttst hbtttt
std_age      0.205
std_bci     -0.013 -0.006
rank2       -0.443  0.137  0.072
rank3       -0.293  0.224  0.204  0.372
rank4       -0.014  0.101  0.266  0.125  0.165
sexM        -0.414 -0.117 -0.111 -0.065 -0.093 -0.128
year2021    -0.098 -0.065  0.096 -0.083  0.007  0.049 -0.153
habitatsoth -0.095  0.048  0.147  0.025  0.196  0.112 -0.169  0.123
habitatteut -0.125 -0.151  0.184  0.127  0.119  0.073  0.042 -0.085  0.078
lbinom1     -0.504 -0.418 -0.036  0.030 -0.116 -0.118 -0.099  0.111 -0.087 -0.070
optimizer (nloptwrap) convergence code: 0 (OK)
boundary (singular) fit: see help('isSingular')
```

2.6. Significance values

```
> Anova(model_faith_final)
Analysis of Deviance Table (Type II Wald chisquare tests)

Response: log_faith
             Chisq Df  Pr(>Chisq)
std_age     1.2377  1    0.26591
std_bci     1.3281  1    0.24914
rank        4.3856  3    0.22272
sex         0.0607  1    0.80540
year        0.0065  1    0.93569
habitat     6.3988  2    0.04079 *
lbinom      1.3759  1    0.24081
---
Signif. codes:  0 '***' 0.001 '**' 0.01 '*' 0.05 '.' 0.1 ' ' 1
```

2.6.1. Multiple comparison test for "Habitat"

```
> library(multcomp)

> multicomp <- glht(model_faith, linfct = mcp(habitat="Tukey")) #multicomparisson for linear models

> confint(glht(model_faith_final_lbinom, mcp(habitat="Tukey")))

     Simultaneous Confidence Intervals

Multiple Comparisons of Means: Tukey Contrasts


Fit: lmer(formula = log_faith ~ std_age + std_bci + rank + sex +
    year + habitat + lbinom + (1 | nest/ring_number), data = metadata)

Quantile = 2.3042
95% family-wise confidence level


Linear Hypotheses:
                   Estimate       lwr       upr
south - north == 0  0.09468    -0.02890   0.21826
teuto - north == 0  0.21971    -0.04067   0.48009
teuto - south == 0  0.12503    -0.15431   0.40438

> summary(multicomp, test = adjusted("holm"))

 Simultaneous Tests for General Linear Hypotheses

Multiple Comparisons of Means: Tukey Contrasts


Fit: lmer(formula = log_faith ~ std_age + std_bci_two + rank + sex +
    year + habitat + lbinom + (1 | nest/ring_number), data = metadata)

Linear Hypotheses:
                   Estimate   Std. Error  z value  Pr(>|z|)
south - north == 0  0.09468     0.05363    1.765    0.116
teuto - north == 0  0.21971     0.11300    1.944    0.116
teuto - south == 0  0.12503     0.12123    1.031    0.302
(Adjusted p values reported -- BH method)
```

2.7. Marginal and Conditional R-squared

```
> r.squaredGLMM(model_faith)
           R2m          R2c
[1,]    0.09305071   0.09305071
```

2.8. Plot model effects

```
plot(allEffects(model_faith))
```

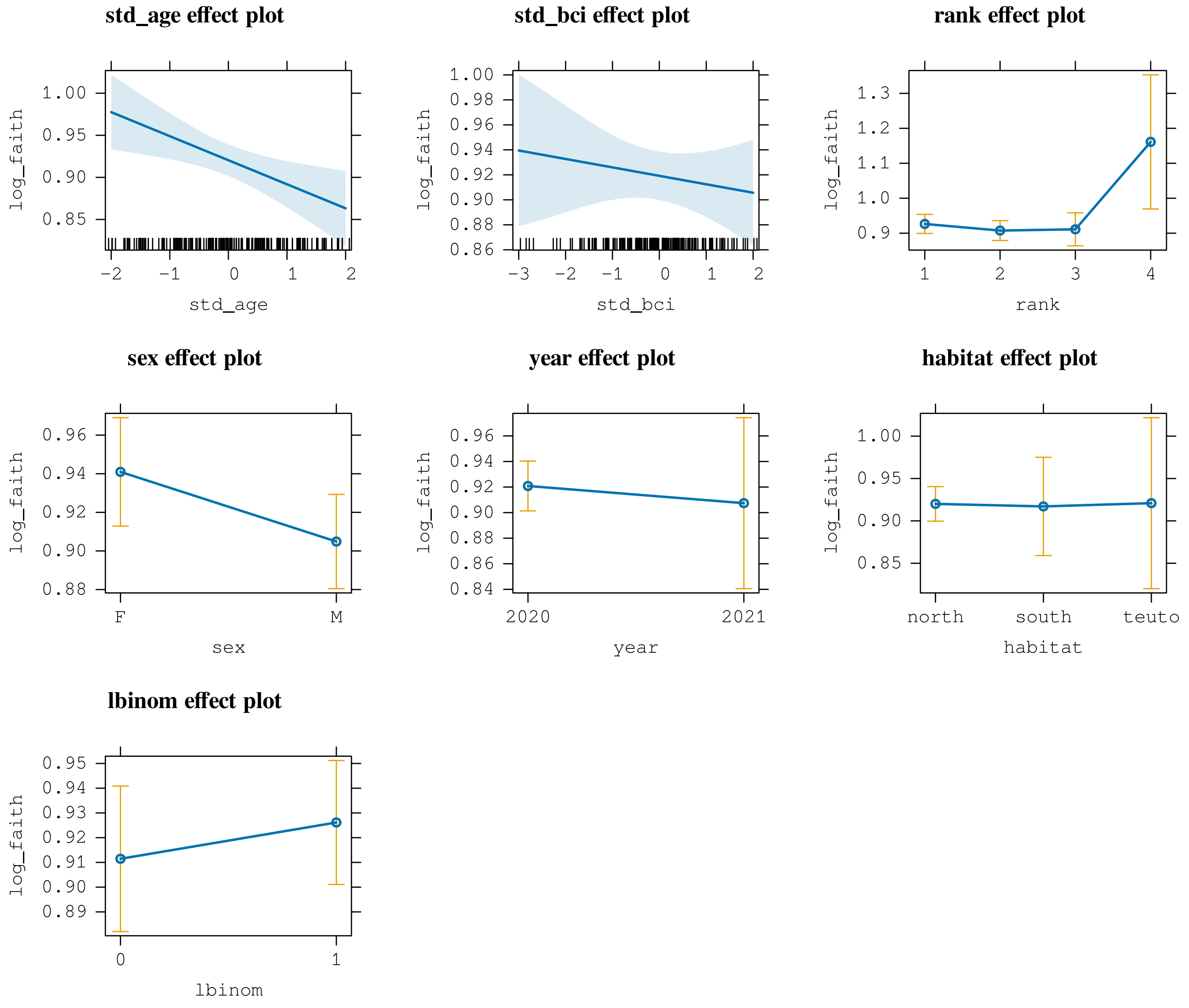

2.9. Significance of random effects

```
> lmerTest::ranova(model_faith_final_lbinom)
boundary (singular) fit: see help('isSingular')
boundary (singular) fit: see help('isSingular')
ANOVA-like table for random-effects: Single term deletions

Model:
log_faith ~ std_age + std_bci_two + rank + sex + year + habitat + lbinom + (1 | ring_number:nest) + (1 | nest)
                       npar logLik    AIC LRT Df Pr(>Chisq)
<none>                   14 5.0039 17.992
(1 | ring_number:nest)   13 5.0039 15.992   0  1          1
(1 | nest)               13 5.0039 15.992   0  1          1
```
