# Supplementary material for: Early-life factors shaping the gut microbiota of Common buzzard nestlings
Source: Anim Microbiome. 2024 May 14;6:27. doi: 10.1186/s42523-024-00313-8 (PMC11092241; doi:10.1186/s42523-024-00313-8)
Supplement: Supplementary file 6 — Supplementary file1 (PDF 175 kb) [file 42523_2024_313_MOESM6_ESM.pdf]

Constructing dyadic data from non-independent observations

Based on the workflow described here https://github.com/nuorenarra/Analysing-dyadic-data-with-brms

Table of Contents

Constructing dyadic data from non-independent observations
- Table of Contents

A) 16S rRNA dyadic data set
- 1. Cumulative Sum Scaling (CSS) normalization
- 2. Read in the data
- 3. Construct matrices to create the dayadic dataset
  - 3.1. Microbiome dissimilarity/distance matrices (BC, WU)
  - 3.2. SEX similarity matrix
  - 3.2.1 SEX combination matrix
  - 3.3. HABITAT similarity matrix
  - 3.3.1 HABITAT combination-factor matrix
  - 3.4. NEST similarity matrix
  - 3.5. YEAR similarity matrices
  - 3.6. INFECTION status combination-factor matrix
  - 3.7. AGE diference matrix
  - 3.8. BCI diference matrix
- 4. Unravel matrices into one dyadic data frame
  - 4.1 Build dyadic dataset
  - 4.2 Add Individual ID and Sample ID combinations to the data set

B) 28S rRNA dyadic data set
- 1. Cumulative Sum Scaling (CSS) normalization
- 2. Read in the data
- 3. Construct matrices to create the dayadic dataset
  - 3.1. Microbiome dissimilarity/distance matrices (BC, WU)
  - 3.2. SEX similarity matrix
  - 3.2.1 SEX combination matrix
  - 3.3. HABITAT similarity matrix
  - 3.3.1 HABITAT combination-factor matrix
  - 3.4. NEST similarity matrix
  - 3.5. YEAR similarity matrices
  - 3.6. INFECTION status combination-factor matrix
  - 3.7. AGE diference matrix
  - 3.8. BCI diference matrix
- 4. Unravel matrices into one dyadic data frame
  - 4.1. Build dyadic dataset
  - 4.2. Add Individual ID and Sample ID combinations to the data set

---

A) 16S rRNA dyadic data set

1. Cumulative Sum Scaling (CSS) normalization

```
# Load libraries
library(qiime2R)
library(phyloseq)
library(tidyverse)
library(microbiome)
library(metagenomeSeq)


# Create phyloseq object

ps <- qza_to_phyloseq(
  features="beta-table.qza",
  taxonomy="taxonomy.qza",
  tree = "rooted-tree.qza",
  metadata = "metadata.tsv")

#Extract taxonomy
taxonomy <- as.data.frame(tax_table(ps))

#Edit taxonomy file (for some reason Kingdom name comes with "d_" before)
taxonomy$Kingdom <- gsub("d__","",as.character(taxonomy$Kingdom))
taxonomy <- as.matrix(taxonomy)

#Extract phylogeny file
tree <- phy_tree(ps) # its easier to get the tree file in the correct format this way. first build the ps
object and then extract the tree


# CSS data transformation

## First Convert the phyloseq object to a metagenomeSeq object (MRexperiment)
meta.obj <- phyloseq_to_metagenomeSeq(ps)

## Normalise counts
meta.obj <- cumNorm(meta.obj, p = cumNormStatFast(meta.obj))

## Convert CSS data into data.frame-formatted OTU table (log transformed data)
asv_table_css <- MRcounts(meta.obj, norm = TRUE, log = TRUE)


# Make a new phyloseq object with with the new CSS transformed ASV table
asv_table_css <- otu_table(asv_table_css, taxa_are_rows = TRUE)
taxonomy <- tax_table(taxonomy)
metadata <- sample_data(metadata)
tree <- phy_tree(tree)

ps_css <- phyloseq(asv_table_css, taxonomy, metadata, tree)

otu <- as.data.frame(otu_table(ps_css))

# Explore phyloseq object
summarize_phyloseq(ps_css)
sample_names(ps_css) # looks at the sample names on the phyloseq object
meta(ps_css) # retrieves the metadata file
sample_data(ps_css) # retrieves the metadata file
taxa(ps) # retrieves taxa name (ASV_1, ASV_2...etc)
abundances(ps_css) # retrieves ASV counts table
abundances(ps_css, "compositional") # computes relative abundaces
readcount(ps_css) # number of reads per sample

# Save phyloseq object as rds file for dyatic data construction

saveRDS(ps_css,"phyloseq_css.rds")
```

2. Read in the data

```
# Load Libraries

library(phyloseq)
library(tidyverse)
library(janitor)

#Read in microbiome data and associated sample data in phyloseq format.
micdata<-readRDS( "phyloseq_css.rds")

#See:
micdata

# Extract metadata file from Phyloseq object
sample_data<-sample_data(micdata)

# Edit metadata file
sample_data$ring_number <- as.factor(sample_data$ring_number)
sample_data$identifier <- as.factor(sample_data$identifier)
sample_data$date <- as.Date(sample_data$date, "%m/%d/%Y")
sample_data$std_age <- as.numeric(sample_data$std_age)
sample_data$std_BCI_two <- as.numeric(sample_data$std_BCI_two)
sample_data$sampling_point <- as.factor(sample_data$sampling_point)
sample_data$nest <- as.factor(sample_data$nest)
sample_data$habitat <- as.factor(sample_data$habitat)
sample_data$year <- as.factor(sample_data$year)
sample_data$lbinom <- as.factor(sample_data$lbinom)
sample_data<- clean_names(sample_data)

#make a key for the order of sample names and their associated individual IDs.
key <- data.frame(ID=sample_data(micdata)$ring_number, Sample_name=sample_data(micdata)$identifier)
```

3. Construct matrices to create the dayadic dataset

3.1. Microbiome dissimilarity/distance matrices (BC, WU)

```
#Make Bray curtis matrix from microbiome data using vegdist function embedded in phyloseq::distance
BCM<- as.matrix(phyloseq::distance(micdata, method = "bray", type = "samples"))

#Make unweighted unifrac matrix
UUM <- as.matrix(phyloseq::distance(micdata, method = "unifrac", type = "samples"))

#Make unweighted unifrac matrix
WUM <- as.matrix(phyloseq::distance(micdata, method = "wunifrac", type = "samples"))

# Assign individual names to rownames based on the key constructed before
all(rownames(BCM)==key$Sample_name)
all(rownames(UUM)==key$Sample_name)
all(rownames(WUM)==key$Sample_name)

#Eyeball  dissimilarities across individuals
BCM
WUM
```

```
#Save matrices
saveRDS(BCM,"ready-matrices/BCM.rds")
saveRDS(WUM,"ready-matrices/WUM.rds")
```

3.2. SEX similarity matrix

```
# The resulting matrix will have for each individual pair a value of "1"= same sex or "0"=different sex

#Create data frame with each Individual name (character) and their nest ID (Character)
sex_frame<-sample_data[,c("ring_number","sex")]
sex_frame$ring_number<-as.character(sex_frame$ring_number)
sex_frame$sex<-as.character(sex_frame$sex)

#Create an empty numeric matrix to fill with distances
sexM<-array(0,c(nrow(sex_frame),nrow(sex_frame)))

#Derive matrix with binary Age similarity between each sample
for(i in 1:nrow(sex_frame)){
  for(j in 1:nrow(sex_frame)){
    if(sex_frame$sex[i]==sex_frame$sex[j]){
      sexM[i,j]= 1 #same sex
    } else{
      sexM[i,j]= 0 #differente sex
    }
  }
}

#Name rown amd colnames with individual names
all(rownames(sexM)==key$ID)
rownames(sexM)<-key$Sample_name
colnames(sexM)<-key$Sample_name

sexM

#Save matrix to ready matrices folder
saveRDS(sexM,"SEXM.rds")
```

3.2.1 SEX combination matrix

```
#The resulting matrix will have for each individual pair a value of "FF"= both female or "FM"=male and a female "MM"=both male.

#Create data frame with each Individual name (character) and their Age (Character)
Sex_frame<-sample_data[,c("ring_number","sex")]
Sex_frame$ring_number<-as.character(Sex_frame$ring_number)
Sex_frame$sex<-as.character(Sex_frame$sex)

#Create an empty character matrix to fill with characters
SEXM<-array(as.character(NA),c(nrow(Sex_frame),nrow(Sex_frame)))

for(i in 1:nrow(Sex_frame)){
  for(j in 1:nrow(Sex_frame)){
    if(Sex_frame$sex[i]=="F" & Sex_frame$sex[i]==Sex_frame$sex[j]){
      SEXM[i,j]= "FF"}
    if(Sex_frame$sex[i]=="M" & Sex_frame$sex[i]==Sex_frame$sex[j]){
      SEXM[i,j]= "MM"}
    if( Sex_frame$sex[i]!=Sex_frame$sex[j]){
      SEXM[i,j]= "FM"}
  }
```

```
}


#Name rown amd colnames with individual names
rownames(SEXM)<-key$Sample_name
colnames(SEXM)<-key$Sample_name

#Save matrix to ready matrices folder
saveRDS(SEXM,"SEXM_comb.rds")
```

3.3. HABITAT similarity matrix

```
# The resulting matrix will have for each individual pair a value of "1"= same habitat or "0"=different habitat

#Create data frame with each Individual name (character) and their nest ID (Character)
habitat_frame<-sample_data[,c("ring_number","habitat")]
habitat_frame$ring_number<-as.character(habitat_frame$ring_number)
habitat_frame$habitat<-as.character(habitat_frame$habitat)

#Create an empty numeric matrix to fill with distances
habitatM<-array(0,c(nrow(habitat_frame),nrow(habitat_frame)))

#Derive matrix with binary Age similarity between each sample
for(i in 1:nrow(habitat_frame)){
  for(j in 1:nrow(habitat_frame)){
    if(habitat_frame$habitat[i]==habitat_frame$habitat[j]){
      habitatM[i,j]= 1 #same habitat
    } else{
      habitatM[i,j]= 0 #differente habitat
    }
  }
}

#Name rown amd colnames with individual names
all(rownames(habitatM)==key$ID)
rownames(habitatM)<-key$Sample_name
colnames(habitatM)<-key$Sample_name

habitatM

#Save matrix to ready matrices folder
saveRDS(habitatM,"habitatM.rds")
```

3.3.1 HABITAT combination-factor matrix

```
#The resulting matrix will have for each individual pair a value of "NN"= both North or "SS"=both south, "TT=
both Teuto and all the interations.
#This type of variables are better than binary variables in revealing how some trends may be affected by
individual-level factors.

#Create data frame with each Individual name (character) and their Age (Character)
habitat_frame<-sample_data[,c("ring_number","habitat")]
habitat_frame$ring_number<-as.character(habitat_frame$ring_number)
habitat_frame$habitat<-as.character(habitat_frame$habitat)

# Change habitat levels from south,north teuto to S,N,T
```

```
habitat_frame$habitat <- with(habitat_frame, factor(habitat, levels = c('north', 'south', 'teuto'), labels = 
c("N", "S", "T")))


#Create an empty character matrix to fill with characters
habitatM<-array(as.character(NA),c(nrow(habitat_frame),nrow(habitat_frame)))

for(i in 1:nrow(habitat_frame)){
  for(j in 1:nrow(habitat_frame)){
    if(habitat_frame$habitat[i]=="N" & habitat_frame$habitat[i]==habitat_frame$habitat[j]){
      habitatM[i,j]= "NN"}
    if(habitat_frame$habitat[i]=="S" & habitat_frame$habitat[i]==habitat_frame$habitat[j]){
      habitatM[i,j]= "SS"}
    if(habitat_frame$habitat[i]=="T" & habitat_frame$habitat[i]==habitat_frame$habitat[j]){
      habitatM[i,j]= "TT"}
    if( habitat_frame$habitat[i]=="N" & habitat_frame$habitat[j]=="S"){
      habitatM[i,j]= "NS"}
    if( habitat_frame$habitat[i]=="S" & habitat_frame$habitat[j]=="N"){
      habitatM[i,j]= "NS"}
    if( habitat_frame$habitat[i]=="N" & habitat_frame$habitat[j]=="T"){
      habitatM[i,j]= "NT"}
    if( habitat_frame$habitat[i]=="T" & habitat_frame$habitat[j]=="N"){
      habitatM[i,j]= "NT"}
    if( habitat_frame$habitat[i]=="S" & habitat_frame$habitat[j]=="T"){
      habitatM[i,j]= "ST"}
    if( habitat_frame$habitat[i]=="T" & habitat_frame$habitat[j]=="S"){
      habitatM[i,j]= "ST"}
  }
}

#Name rown amd colnames with individual names
rownames(habitatM)<-key$Sample_name
colnames(habitatM)<-key$Sample_name

#Save matrix to ready matrices folder
saveRDS(habitatM,"habitatM_comb.rds")
```

3.4. NEST similarity matrix

```
# The resulting matrix will have for each individual pair a value of "1"= same nest or "0"=different nest

#Create data frame with each Individual name (character) and their nest ID (Character)
nest_frame<-sample_data[,c("ring_number","nest")]
nest_frame$ring_number<-as.character(nest_frame$ring_number)
nest_frame$nest<-as.character(nest_frame$nest)

#Create an empty numeric matrix to fill with distances
nestM<-array(0,c(nrow(nest_frame),nrow(nest_frame)))

#Derive matrix with binary nest similarity between each sample
for(i in 1:nrow(nest_frame)){
  for(j in 1:nrow(nest_frame)){
    if(nest_frame$nest[i]==nest_frame$nest[j]){
      nestM[i,j]= 1
    } else{
      nestM[i,j]= 0
    }
  }
}

#Name rown amd colnames with individual names
all(rownames(nestM)==key$ID)
rownames(nestM)<-key$Sample_name
```

```
colnames(nestM)<-key$Sample_name

nestM

#Save matrix to ready matrices folder
saveRDS(nestM,"nestM.rds")
```

3.5. YEAR similarity matrices

```
#The resulting matrix will have for each individual pair a value of "1"= same year or "0"=different year

#Create data frame with each Individual name (character) and their Age (Character)
year_frame<-sample_data[,c("ring_number","year")]
year_frame$ring_number<-as.character(year_frame$ring_number)
year_frame$year<-as.character(year_frame$year)

#Create an empty numeric matrix to fill with distances
yearM<-array(0,c(nrow(year_frame),nrow(year_frame)))

#Derive matrix with binary Age similarity between each sample
for(i in 1:nrow(year_frame)){
  for(j in 1:nrow(year_frame)){
    if(year_frame$year[i]==year_frame$year[j]){
      yearM[i,j]= 1
    } else{
      yearM[i,j]= 0
    }
  }
}

#Name rown amd colnames with individual names
all(rownames(yearM)==key$ID)
rownames(yearM)<-key$Sample_name
colnames(yearM)<-key$Sample_name

yearM

#Save matrix to ready matrices folder
saveRDS(yearM,"yearM.rds")
```

3.6. INFECTION status combination-factor matrix

```
#The resulting matrix will have for each individual pair a value of "II"= both infected or "NiNi"=both uninfected, "NiI= non-infected vs infected.


#Create data frame with each Individual name (character) and their infection status (Character)
lbinom_frame<-sample_data[,c("ring_number","lbinom")]
lbinom_frame$ring_number<-as.character(lbinom_frame$ring_number)
lbinom_frame$lbinom<-as.character(lbinom_frame$lbinom)

#lbinom_frame <- replace(lbinom_frame, is.na(lbinom_frame), "NA")
lbinom_frame <- replace(lbinom_frame, lbinom_frame=="", "NA") #  some missing data replaced by NA

lbinom_frame$lbinom <- with(lbinom_frame, factor(lbinom, levels = c('1', '0', "NA"), labels = c("I", "Ni", "NA")))
```

```
#Create an empty character matrix to fill with characters
lbinomM<-array(as.character(NA),c(nrow(lbinom_frame),nrow(lbinom_frame)))

for(i in 1:nrow(lbinom_frame)){
  for(j in 1:nrow(lbinom_frame)){
    if(lbinom_frame$lbinom[i]=="I" & lbinom_frame$lbinom[i]==lbinom_frame$lbinom[j]){
      lbinomM[i,j]= "II"}
    if(lbinom_frame$lbinom[i]=="Ni" & lbinom_frame$lbinom[i]==lbinom_frame$lbinom[j]){
      lbinomM[i,j]= "NiNi"}
    if( lbinom_frame$lbinom[i]=="I" & lbinom_frame$lbinom[j]=="Ni"){
      lbinomM[i,j]= "NiI"}
    if( lbinom_frame$lbinom[i]=="Ni" & lbinom_frame$lbinom[j]=="I"){
      lbinomM[i,j]= "NiI"}
}}

#Name rown amd colnames with individual names
rownames(lbinomM)<-key$Sample_name
colnames(lbinomM)<-key$Sample_name

#Save matrix to ready matrices folder
saveRDS(lbinomM,"lbinomM.rds")
```

3.7. AGE diference matrix

```
#This matrix will describe the distance in days between age of microbiome samples

#Transform dates into a numeric variable
sample_data$age_days<-as.numeric(sample_data$age_days)

#Create data frame with each sample name (character) and age (numeric)
AgeTime_frame<-sample_data[,c("ring_number","age_days")]
AgeTime_frame$ring_number<-as.character(AgeTime_frame$ring_number)

#Create an empty matrix to fill with distances
AGEM<-array(0,c(nrow(AgeTime_frame),nrow(AgeTime_frame)))

#Derive matrix with time distances between each sample using abs()-function
for (i in 1:nrow(AgeTime_frame)){
  for (j in 1:nrow(AgeTime_frame))
  {AGEM[i,j]=abs(AgeTime_frame$age_days[i] -AgeTime_frame$age_days[j])
  }
}

#Note that age diference matrix has rownames and colnames in the same order as key
all(rownames(AGEM)==key$Sample_name)

#Name rown amd colnames with individual names
rownames(AGEM)<-key$Sample_name
colnames(AGEM)<-key$Sample_name

AGEM

#Save matrix to ready matrices folder
saveRDS(AGEM,AGEM.rds")
```

3.8. BCI diference matrix

```
#This matrix will describe the diference in body condition between the microbiome samples

#Transform body condition into a numeric variable
sample_data$bci_two<-as.numeric(sample_data$bci_two)

#Create data frame with each sample name (character) and sampling time (numeric)
BC_frame<-sample_data[,c("ring_number","bci_two")]
BC_frame$ring_number<-as.character(BC_frame$ring_number)

#Create an empty matrix to fill with distances
BCondM<-array(0,c(nrow(BC_frame),nrow(BC_frame)))

#Derive matrix with time distances between each sample using abs()-function
for (i in 1:nrow(BC_frame)){
  for (j in 1:nrow(BC_frame))
  {BCondM[i,j]=abs(BC_frame$bci_two[i] -BC_frame$bci_two[j])
  }
}

#Note that Temporal distance matrix has rownames and colnames in the same order as key
all(rownames(AGEM)==key$Sample_name)

# So we can just call the rownames and colnames with the names of individuals since there is just one sample
per individual in this data set

#Name rown amd colnames with individual names
rownames(BCondM)<-key$Sample_name
colnames(BCondM)<-key$Sample_name

BCondM

#Save matrix to ready matrices folder
saveRDS(BCondM,"BCondM.rds")
```

4. Unravel matrices into one dyadic data frame

4.1 Build dyadic dataset

```
#Read in microbial distance matrices if not in already
BCM <- readRDS("BCM.rds") # bray-curtis
WUM <- readRDS("WUM.rds") # weighted unifrac

# Ready in matrices of other variables

AGEM<-readRDS("AGEM.rds") # age
BCI_twoM <-readRDS("BCI_two_M.rds") #body condition
yearM <- readRDS("yearM.rds") # year
nestM <- readRDS("nestM.rds") # nest

SEXM <- readRDS("SEXM.rds") # sex similarity
SEXM_comb <- readRDS("SEXM_comb.rds") # sex combination

habitatM <- readRDS("habitatM.rds") # habitat similarity
habitatM <- readRDS("habitatM_comb.rds") # habitat combination

lbinomM <- readRDS("lbinomM.rds") # infection status
```

```
#First unravel the matrices into vectors matching the lower quantile of each matrix.
#From numeric matrices, this can be done by making a list (c()) of the distance object (dist()) derived from
the matrix.
#as.dist() by default includes only the lower quantile of the matrix and excludes the diagonal.

BC <- c(as.dist(BCM))
UU <- c(as.dist(UUM))
WU <- c(as.dist(WUM))
age <- c(as.dist(AGEM))
bci_two <-c(as.dist(BCI_twoM))
nest <- c(as.dist(nestM))
year <- c(as.dist(yearM))
sex <- c(as.dist(SEXM))

#From categorical matrices, this can be done by making a list (c()) of the lower quantile of the matrix with
lower.tri() -function.

sex_comb <- c(SEXM_comb[lower.tri(SEXM_comb)])
habitat_comb <- c(TREATMM[lower.tri(habitatM_comb)])
lbinom <- c(lbinomM[lower.tri(lbinomM)])



#Combine these vectors into a data frame
data.dyad<-data.frame(BC_dissim=BC , UU_distance=UU, WU_distance=WU, age_diference=age,
                       bci_one = bci_one, bci_diference = bci_two, lbinom_comp = lbinom,
                       nest_sim=nest, habitat_sim=habitat,,habitat_comb=habitat_comb,
sex_sim=sex,sex_comb=sex_comb year_sim = year)
```

4.2 Add Individual ID and Sample ID combinations to the data set

```
#Add the identities of both individuals in each dyad as separate columns into the data frame and exclude self-
comparisons (as these are not meaningful).

# extracting Individual-combinations present in the matrices
list<-expand.grid(key$Sample_name,key$Sample_name)

# This created individual-to-same-individual pairs as well. Get rid of these:
list<-list[which(list$Var1!=list$Var2),]

# this still has both quantiles in--> add 'unique' key
list$key <- apply(list, 1, function(x)paste(sort(x), collapse=''))
list<-subset(list, !duplicated(list$key))

# sanity check that the Individual name combinations are in the same exact order as the lower quantile value
vector of the matrices
i=34

BCM[which(rownames(BCM)==list$Var1[i]),which(colnames(BCM)==list$Var2[i])]==BC[i]
UUM[which(rownames(UUM)==list$Var1[i]),which(colnames(UUM)==list$Var2[i])]==UU[i]
WUM[which(rownames(WUM)==list$Var1[i]),which(colnames(WUM)==list$Var2[i])]==WU[i]

AGEM[which(rownames(AGEM)==list$Var1[i]),which(colnames(AGEM)==list$Var2[i])]==age[i]
SEXM[which(rownames(SEXM)==list$Var1[i]),which(colnames(SEXM)==list$Var2[i])]==sex[i]
TREATMM[which(rownames(TREATMM)==list$Var1[i]),which(colnames(TREATMM)==list$Var2[i])]==treatm[i]
yearM[which(rownames(yearM)==list$Var1[i]),which(colnames(yearM)==list$Var2[i])]==year[i]
nestM[which(rownames(nestM)==list$Var1[i]),which(colnames(nestM)==list$Var2[i])]==nest[i]


# add the names of both individuals participating in each dyad into the data frame
data.dyad$sampleA<-list$Var2
```

```
data.dyad$sampleB<-list$Var1

# make a new key for the order of sample names and their associated individual IDs.
key2 <- data.frame(ID=sample_data(micdata)$ring_number, sampleA =sample_data(micdata)$identifier, sampleB
=sample_data(micdata)$identifier )

# merge individual ID to each sample name (sampleA -> IDA; sampleB -> IDB)
listdf = list(data.dyad, key2) # built list
data.dyad <- listdf %>% reduce(left_join, by ="sampleA", keep = FALSE) # merge based on sampleA column

data.dyad <- data.dyad[,-19] # delete column duplicate
data.dyad <- rename(data.dyad, IDA = ID, sampleB = sampleB.x) # change column names

listdf = list(data.dyad, key2) # update list
data.dyad <- listdf %>% reduce(left_join, by ="sampleB", keep = FALSE) # merge by sample B
data.dyad <- data.dyad[,-20] # delete column duplicate

data.dyad <- rename(data.dyad, IDB = ID, sampleA = sampleA.x) # change column names


# Make sure you have got rid of all self comparisons
data.dyad<-data.dyad[which(data.dyad$sampleA!=data.dyad$sampleB),]

#Save dyadic data file
saveRDS(data.dyad,"data_dyad.rds")
```

Example of the data dyad (first 20 rows)

sampleA	sampleB	IDA	IDB	BC_dissim	WU_distance	age_diference	bci_diference	lbinom_comp	nest_sim	habitat_sim	habitat_comb	sex_sim	sex_comb	year_sim
S001	S002	3419291	3419288	0.708478913	0.048377791	0.174242424		II	0	1	NN	0	FM	1
S001	S003	3419291	3419287	0.742968667	0.06477989	0.178030303	0.159085291	II	0	1	NN	1	MM	1
S001	S004	3419291	3419286	0.621086265	0.063355594	0.071969697	0.232891266	II	0	1	NN	1	MM	1
S001	S005	3419291	3419285	0.728804856	0.056634477	0.185606061	0.043899636	NiI	0	1	NN	0	FM	1
S001	S006	3419291	3419284	0.72086191	0.04764569	0.15530303	0.057296175	NiI	0	1	NN	0	FM	1
S001	S007	3419291	3419283	0.58844846	0.081624471	0.026515152	0.219643831	NiI	0	1	NN	1	MM	1
S001	S008	3419291	3419282	0.638061537	0.058416954	0.03030303	0.192283125	NiI	0	1	NN	1	MM	1
S001	S009	3419291	3419281	0.554663665	0.058029588	0.018939394	0.223148272	NiI	0	1	NN	0	FM	1
S001	S010	3419291	3419279	0.574530808	0.056723361	0.170454546	0.003992333	NiI	0	1	NN	1	MM	1
S001	S011	3419291	3419278	0.580341291	0.042190368	0.068181818	0.045747277	II	0	1	NN	1	MM	1
S001	S012	3419291	3419276	0.566648959	0.067971619	0.079545454	0.180183235	NiI	0	1	NN	1	MM	1
S001	S013	3419291	3419275	0.442679036	0.053512563	0.060606061	0.046341041	NiI	0	1	NN	1	MM	1
S001	S014	3419291	3419280	0.560013748	0.043511334	0.196969697	0.098652122	II	0	1	NN	0	FM	1
S001	S015	3419291	3419297	0.671730803	0.066848843	0.246212121	0.046668815	NiI	0	1	NN	0	FM	1
S001	S016	3419291	3419294	0.666217633	0.052062348	0.231060606	0.201903615	NiI	0	0	NS	1	MM	1
S001	S017	3419291	3121005	0.631848671	0.065660102	0.034090909	0.172042132	NiI	0	1	NN	0	FM	1
S001	S018	3419291	3121004	0.656930239	0.063652836	0.011363636	0.101811651	II	0	1	NN	0	FM	1
S001	S019	3419291	3121001	0.731432769	0.066831972	0.071969697	0.02611092	II	0	1	NN	0	FM	1
S001	S020	3419291	3121006	0.650345451	0.061676228	0.193181818	0.140552659	NiI	0	1	NN	0	FM	1
S001	S021	3419291	3419299	0.712390555	0.050166339	0.049242424	0.0043404	NiI	0	1	NN	0	FM	1

B) 28S rRNA dyadic data set

1. Cumulative Sum Scaling (CSS) normalization

```
# Load libraries
library(qiime2R)
library(phyloseq)
library(tidyverse)
library(microbiome)
library(metagenomeSeq)


# Create phyloseq object

ps <- qza_to_phyloseq(
  features="beta-table.qza",
  taxonomy="taxonomy.qza",
  tree = "rooted-tree.qza",
  metadata = "metadata.tsv")

#Extract taxonomy
taxonomy <- as.data.frame(tax_table(ps))

#Edit taxonomy file (for some reason Kingdom name comes with "d_" before)
taxonomy$Kingdom <- gsub("d__","",as.character(taxonomy$Kingdom))
taxonomy <- as.matrix(taxonomy)

#Extract phylogeny file
tree <- phy_tree(ps) # its easier to get the tree file in the correct format this way. first build the ps
object and then extract the tree


# CSS data transformation

## First Convert the phyloseq object to a metagenomeSeq object (MRexperiment)
meta.obj <- phyloseq_to_metagenomeSeq(ps)

## Normalise counts
meta.obj <- cumNorm(meta.obj, p = cumNormStatFast(meta.obj))

## Convert CSS data into data.frame-formatted OTU table (log transformed data)
asv_table_css <- MRcounts(meta.obj, norm = TRUE, log = TRUE)


# Make a new phyloseq object with with the new CSS transformed ASV table
asv_table_css <- otu_table(asv_table_css, taxa_are_rows = TRUE)
taxonomy <- tax_table(taxonomy)
metadata <- sample_data(metadata)
tree <- phy_tree(tree)

ps_css <- phyloseq(asv_table_css, taxonomy, metadata, tree)

otu <- as.data.frame(otu_table(ps_css))


# Explore phyloseq object
summarize_phyloseq(ps_css)
sample_names(ps_css) # looks at the sample names on the phyloseq object
meta(ps_css) # retrieves the metadata file
sample_data(ps_css) # retrieves the metadata file
taxa(ps) # retrieves taxa name (ASV_1, ASV_2...etc)
abundances(ps_css) # retrieves ASV counts table
abundances(ps_css, "compositional") # computes relative abundaces
readcount(ps_css) # number of reads per sample


# Save phyloseq object as rds file for dyatic data construction
```

```
saveRDS(ps_css,"phyloseq_css.rds")
```

2. Read in the data

```
# Load Libraries

library(phyloseq)
library(tidyverse)
library(janitor)

#Read in microbiome data and associated sample data in phyloseq format.
micdata<-readRDS( "phyloseq_css.rds")

# remove sample that seems to be outlier. Distance measures always equal to 1 for this sample

micdata <- subset_samples(micdata,  identifier != "S024" & identifier != "S029" & identifier != "S043" &
identifier != "S174" &
                            identifier != "S207")

#See:
micdata

# Extract metadata file from Phyloseq object
sample_data<-sample_data(micdata)

# Edit metadata file
sample_data$ring_number <- as.factor(sample_data$ring_number)
sample_data$identifier <- as.factor(sample_data$identifier)
sample_data$date <- as.Date(sample_data$date, "%m/%d/%Y")
sample_data$std_age <- as.numeric(sample_data$std_age)
sample_data$std_BCI_two <- as.numeric(sample_data$std_BCI_two)
sample_data$sampling_point <- as.factor(sample_data$sampling_point)
sample_data$nest <- as.factor(sample_data$nest)
sample_data$habitat <- as.factor(sample_data$habitat)
sample_data$year <- as.factor(sample_data$year)
sample_data$lbinom <- as.factor(sample_data$lbinom)
sample_data$sex <- as.factor(sample_data$sex)
sample_data<- clean_names(sample_data)

#make a key for the order of sample names and their associated individual IDs.
key <- data.frame(ID=sample_data(micdata)$ring_number, Sample_name=sample_data(micdata)$identifier)
```

3. Construct matrices to create the dayadic dataset

3.1. Microbiome dissimilarity/distance matrices (BC, WU)

```
#Make Bray curtis matrix from microbiome data using vegdist function embedded in phyloseq::distance
BCM<- as.matrix(phyloseq::distance(micdata, method = "bray", type = "samples"))

#Make unweighted unifrac matrix
UUM <- as.matrix(phyloseq::distance(micdata, method = "unifrac", type = "samples"))

#Make unweighted unifrac matrix
WUM <- as.matrix(phyloseq::distance(micdata, method = "wunifrac", type = "samples"))

# Assign individual names to rownames based on the key constructed before
```

```
all(rownames(BCM)==key$Sample_name)
all(rownames(UUM)==key$Sample_name)
all(rownames(WUM)==key$Sample_name)

#Eyeball  dissimilarities across individuals
BCM
WUM

#Save matrices
saveRDS(BCM,"ready-matrices/BCM.rds")
saveRDS(WUM,"ready-matrices/WUM.rds")
```

3.2. SEX similarity matrix

```
# The resulting matrix will have for each individual pair a value of "1"= same sex or "0"=different sex

#Create data frame with each Individual name (character) and their nest ID (Character)
sex_frame<-sample_data[,c("ring_number","sex")]
sex_frame$ring_number<-as.character(sex_frame$ring_number)
sex_frame$sex<-as.character(sex_frame$sex)

#Create an empty numeric matrix to fill with distances
sexM<-array(0,c(nrow(sex_frame),nrow(sex_frame)))

#Derive matrix with binary Age similarity between each sample
for(i in 1:nrow(sex_frame)){
  for(j in 1:nrow(sex_frame)){
    if(sex_frame$sex[i]==sex_frame$sex[j]){
      sexM[i,j]= 1 #same sex
    } else{
      sexM[i,j]= 0 #differente sex
}}}

#Name rown amd colnames with individual names
all(rownames(sexM)==key$ID)
rownames(sexM)<-key$Sample_name
colnames(sexM)<-key$Sample_name

sexM

#Save matrix to ready matrices folder
saveRDS(sexM,"SEXM.rds")
```

3.2.1 SEX combination matrix

```
#The resulting matrix will have for each individual pair a value of "FF"= both female or "FM"=male and a female
"MM"=both male.

#Create data frame with each Individual name (character) and their Age (Character)
Sex_frame<-sample_data[,c("ring_number","sex")]
Sex_frame$ring_number<-as.character(Sex_frame$ring_number)
Sex_frame$sex<-as.character(Sex_frame$sex)

#Create an empty character matrix to fill with characters
SEXM<-array(as.character(NA),c(nrow(Sex_frame),nrow(Sex_frame)))

for(i in 1:nrow(Sex_frame)){
  for(j in 1:nrow(Sex_frame)){
    if(Sex_frame$sex[i]=="F" & Sex_frame$sex[i]==Sex_frame$sex[j]){
```

```
      SEXM[i,j]= "FF"}
    if(Sex_frame$sex[i]=="M" & Sex_frame$sex[i]==Sex_frame$sex[j]){
      SEXM[i,j]= "MM"}
    if( Sex_frame$sex[i]!=Sex_frame$sex[j]){
      SEXM[i,j]= "FM"}
  }}

#Name rown amd colnames with individual names
rownames(SEXM)<-key$Sample_name
colnames(SEXM)<-key$Sample_name

#Save matrix to ready matrices folder
saveRDS(SEXM,"SEXM_comb.rds")
```

3.3. HABITAT similarity matrix

```
# The resulting matrix will have for each individual pair a value of "1"= same habitat or "0"=different habitat

#Create data frame with each Individual name (character) and their nest ID (Character)
habitat_frame<-sample_data[,c("ring_number","habitat")]
habitat_frame$ring_number<-as.character(habitat_frame$ring_number)
habitat_frame$habitat<-as.character(habitat_frame$habitat)

#Create an empty numeric matrix to fill with distances
habitatM<-array(0,c(nrow(habitat_frame),nrow(habitat_frame)))

#Derive matrix with binary Age similarity between each sample
for(i in 1:nrow(habitat_frame)){
  for(j in 1:nrow(habitat_frame)){
    if(habitat_frame$habitat[i]==habitat_frame$habitat[j]){
      habitatM[i,j]= 1 #same habitat
    } else{
      habitatM[i,j]= 0 #differente habitat
    }}}

#Name rown amd colnames with individual names
all(rownames(habitatM)==key$ID)
rownames(habitatM)<-key$Sample_name
colnames(habitatM)<-key$Sample_name

habitatM

#Save matrix to ready matrices folder
saveRDS(habitatM,"habitatM.rds")
```

3.3.1 HABITAT combination-factor matrix

```
#The resulting matrix will have for each individual pair a value of "NN"= both North or "SS"=both south, "TT=
both Teuto and all the interations.
#This type of variables are better than binary variables in revealing how some trends may be affected by
individual-level factors.

#Create data frame with each Individual name (character) and their Age (Character)
habitat_frame<-sample_data[,c("ring_number","habitat")]
habitat_frame$ring_number<-as.character(habitat_frame$ring_number)
habitat_frame$habitat<-as.character(habitat_frame$habitat)
```

```
# Change habitat levels from south,north teuto to S,N,T
habitat_frame$habitat <- with(habitat_frame, factor(habitat, levels = c('north', 'south', 'teuto'), labels =
c("N", "S", "T")))


#Create an empty character matrix to fill with characters
habitatM<-array(as.character(NA),c(nrow(habitat_frame),nrow(habitat_frame)))

for(i in 1:nrow(habitat_frame)){
  for(j in 1:nrow(habitat_frame)){
    if(habitat_frame$habitat[i]=="N" & habitat_frame$habitat[i]==habitat_frame$habitat[j]){
      habitatM[i,j]= "NN"}
    if(habitat_frame$habitat[i]=="S" & habitat_frame$habitat[i]==habitat_frame$habitat[j]){
      habitatM[i,j]= "SS"}
    if(habitat_frame$habitat[i]=="T" & habitat_frame$habitat[i]==habitat_frame$habitat[j]){
      habitatM[i,j]= "TT"}
    if( habitat_frame$habitat[i]=="N" & habitat_frame$habitat[j]=="S"){
      habitatM[i,j]= "NS"}
    if( habitat_frame$habitat[i]=="S" & habitat_frame$habitat[j]=="N"){
      habitatM[i,j]= "NS"}
    if( habitat_frame$habitat[i]=="N" & habitat_frame$habitat[j]=="T"){
      habitatM[i,j]= "NT"}
    if( habitat_frame$habitat[i]=="T" & habitat_frame$habitat[j]=="N"){
      habitatM[i,j]= "NT"}
    if( habitat_frame$habitat[i]=="S" & habitat_frame$habitat[j]=="T"){
      habitatM[i,j]= "ST"}
    if( habitat_frame$habitat[i]=="T" & habitat_frame$habitat[j]=="S"){
      habitatM[i,j]= "ST"}
  }}

#Name rown amd colnames with individual names
rownames(habitatM)<-key$Sample_name
colnames(habitatM)<-key$Sample_name

#Save matrix to ready matrices folder
saveRDS(habitatM,"habitatM_comb.rds")
```

3.4. NEST similarity matrix

```
# The resulting matrix will have for each individual pair a value of "1"= same nest or "0"=different nest

#Create data frame with each Individual name (character) and their nest ID (Character)
nest_frame<-sample_data[,c("ring_number","nest")]
nest_frame$ring_number<-as.character(nest_frame$ring_number)
nest_frame$nest<-as.character(nest_frame$nest)

#Create an empty numeric matrix to fill with distances
nestM<-array(0,c(nrow(nest_frame),nrow(nest_frame)))

#Derive matrix with binary nest similarity between each sample
for(i in 1:nrow(nest_frame)){
  for(j in 1:nrow(nest_frame)){
    if(nest_frame$nest[i]==nest_frame$nest[j]){
      nestM[i,j]= 1
    } else{
      nestM[i,j]= 0
    }}}

#Name rown amd colnames with individual names
all(rownames(nestM)==key$ID)
rownames(nestM)<-key$Sample_name
colnames(nestM)<-key$Sample_name
```

```
nestM

#Save matrix to ready matrices folder
saveRDS(nestM,"nestM.rds")
```

3.5. YEAR similarity matrices

```
#The resulting matrix will have for each individual pair a value of "1"= same year or "0"=different year

#Create data frame with each Individual name (character) and their Age (Character)
year_frame<-sample_data[,c("ring_number","year")]
year_frame$ring_number<-as.character(year_frame$ring_number)
year_frame$year<-as.character(year_frame$year)

#Create an empty numeric matrix to fill with distances
yearM<-array(0,c(nrow(year_frame),nrow(year_frame)))

#Derive matrix with binary Age similarity between each sample
for(i in 1:nrow(year_frame)){
  for(j in 1:nrow(year_frame)){
    if(year_frame$year[i]==year_frame$year[j]){
      yearM[i,j]= 1
    } else{
      yearM[i,j]= 0
    }}}

#Name rown amd colnames with individual names
all(rownames(yearM)==key$ID)
rownames(yearM)<-key$Sample_name
colnames(yearM)<-key$Sample_name

yearM

#Save matrix to ready matrices folder
saveRDS(yearM,"yearM.rds")
```

3.6. INFECTION status combination-factor matrix

```
#The resulting matrix will have for each individual pair a value of "II"= both infected or "NiNi"=both uninfected, "NiI= non-infected vs infected.


#Create data frame with each Individual name (character) and their infection status (Character)
lbinom_frame<-sample_data[,c("ring_number","lbinom")]
lbinom_frame$ring_number<-as.character(lbinom_frame$ring_number)
lbinom_frame$lbinom<-as.character(lbinom_frame$lbinom)

#lbinom_frame <- replace(lbinom_frame, is.na(lbinom_frame), "NA")
lbinom_frame <- replace(lbinom_frame, lbinom_frame=="", "NA") #  some missing data replaced by NA

lbinom_frame$lbinom <- with(lbinom_frame, factor(lbinom, levels = c('1', '0', "NA"), labels = c("I", "Ni", "NA")))



#Create an empty character matrix to fill with characters
lbinomM<-array(as.character(NA),c(nrow(lbinom_frame),nrow(lbinom_frame)))
```

```
for(i in 1:nrow(lbinom_frame)){
  for(j in 1:nrow(lbinom_frame)){
    if(lbinom_frame$lbinom[i]=="I" & lbinom_frame$lbinom[i]==lbinom_frame$lbinom[j]){
      lbinomM[i,j]= "II"}
    if(lbinom_frame$lbinom[i]=="Ni" & lbinom_frame$lbinom[i]==lbinom_frame$lbinom[j]){
      lbinomM[i,j]= "NiNi"}
    if( lbinom_frame$lbinom[i]=="I" & lbinom_frame$lbinom[j]=="Ni"){
      lbinomM[i,j]= "NiI"}
    if( lbinom_frame$lbinom[i]=="Ni" & lbinom_frame$lbinom[j]=="I"){
      lbinomM[i,j]= "NiI"}
}}

#Name rown amd colnames with individual names
rownames(lbinomM)<-key$Sample_name
colnames(lbinomM)<-key$Sample_name

#Save matrix to ready matrices folder
saveRDS(lbinomM,"lbinomM.rds")
```

3.7. AGE diference matrix

```
#This matrix will describe the distance in days between age of microbiome samples

#Transform dates into a numeric variable
sample_data$age_days<-as.numeric(sample_data$age_days)

#Create data frame with each sample name (character) and age (numeric)
AgeTime_frame<-sample_data[,c("ring_number","age_days")]
AgeTime_frame$ring_number<-as.character(AgeTime_frame$ring_number)

#Create an empty matrix to fill with distances
AGEM<-array(0,c(nrow(AgeTime_frame),nrow(AgeTime_frame)))

#Derive matrix with time distances between each sample using abs()-function
for (i in 1:nrow(AgeTime_frame)){
  for (j in 1:nrow(AgeTime_frame))
  {AGEM[i,j]=abs(AgeTime_frame$age_days[i] -AgeTime_frame$age_days[j])
  }}

#Note that age diference matrix has rownames and colnames in the same order as key
all(rownames(AGEM)==key$Sample_name)

#Name rown amd colnames with individual names
rownames(AGEM)<-key$Sample_name
colnames(AGEM)<-key$Sample_name

AGEM

#Save matrix to ready matrices folder
saveRDS(AGEM,AGEM.rds")
```

3.8. BCI diference matrix

```
#This matrix will describe the diference in body condition between the microbiome samples

#Transform body condition into a numeric variable
sample_data$bci_two<-as.numeric(sample_data$bci_two)

#Create data frame with each sample name (character) and sampling time (numeric)
BC_frame<-sample_data[,c("ring_number","bci_two")]
BC_frame$ring_number<-as.character(BC_frame$ring_number)

#Create an empty matrix to fill with distances
BCondM<-array(0,c(nrow(BC_frame),nrow(BC_frame)))

#Derive matrix with time distances between each sample using abs()-function
for (i in 1:nrow(BC_frame)){
  for (j in 1:nrow(BC_frame))
  {BCondM[i,j]=abs(BC_frame$bci_two[i] -BC_frame$bci_two[j])
  }}

#Note that Temporal distance matrix has rownames and colnames in the same order as key
all(rownames(AGEM)==key$Sample_name)

# So we can just call the rownames and colnames with the names of individuals since there is just one sample
per individual in this data set

#Name rown amd colnames with individual names
rownames(BCondM)<-key$Sample_name
colnames(BCondM)<-key$Sample_name

BCondM

#Save matrix to ready matrices folder
saveRDS(BCondM,"BCondM.rds")
```

4. Unravel matrices into one dyadic data frame

4.1. Build dyadic dataset

```
#Read in microbial distance matrices if not in already
BCM <- readRDS("BCM.rds") # bray-curtis
WUM <- readRDS("WUM.rds") # weighted unifrac

# Ready in matrices of other variables

AGEM<-readRDS("AGEM.rds") # age
BCI_twoM <-readRDS("BCI_two_M.rds") #body condition
yearM <- readRDS("yearM.rds") # year
nestM <- readRDS("nestM.rds") # nest

SEXM <- readRDS("SEXM.rds") # sex similarity
SEXM_comb <- readRDS("SEXM_comb.rds") # sex combination

habitatM <- readRDS("habitatM.rds") # habitat similarity
habitatM <- readRDS("habitatM_comb.rds") # habitat combination

lbinomM <- readRDS("lbinomM.rds") # infection status
```

```
#First unravel the matrices into vectors matching the lower quantile of each matrix.
#From numeric matrices, this can be done by making a list (c()) of the distance object (dist()) derived from
the matrix.
#as.dist() by default includes only the lower quantile of the matrix and excludes the diagonal.

BC <- c(as.dist(BCM))
UU <- c(as.dist(UUM))
WU <- c(as.dist(WUM))
age <- c(as.dist(AGEM))
bci_two <-c(as.dist(BCI_twoM))
nest <- c(as.dist(nestM))
year <- c(as.dist(yearM))
sex <- c(as.dist(SEXM))

#From categorical matrices, this can be done by making a list (c()) of the lower quantile of the matrix with
lower.tri() -function.

sex_comb <- c(SEXM_comb[lower.tri(SEXM_comb)])
habitat_comb <- c(TREATMM[lower.tri(habitatM_comb)])
lbinom <- c(lbinomM[lower.tri(lbinomM)])



#Combine these vectors into a data frame
data.dyad<-data.frame(BC_dissim=BC , UU_distance=UU, WU_distance=WU, age_diference=age,
                       bci_diference = bci_two, lbinom_comp = lbinom,
                       nest_sim=nest, habitat_sim=habitat, habitat_comb=habitat_comb, sex_sim=sex,
sex_comb=sex_comb year_sim = year)
```

4.2. Add Individual ID and Sample ID combinations to the data set

```
#Add the identities of both individuals in each dyad as separate columns into the data frame and exclude self-
comparisons (as these are not meaningful).

# extracting Individual-combinations present in the matrices
list<-expand.grid(key$Sample_name,key$Sample_name)

# This created individual-to-same-individual pairs as well. Get rid of these:
list<-list[which(list$Var1!=list$Var2),]

# this still has both quantiles in--> add 'unique' key
list$key <- apply(list, 1, function(x)paste(sort(x), collapse=''))
list<-subset(list, !duplicated(list$key))

# sanity check that the Individual name combinations are in the same exact order as the lower quantile value
vector of the matrices
i=34

BCM[which(rownames(BCM)==list$Var1[i]),which(colnames(BCM)==list$Var2[i])]==BC[i]
UUM[which(rownames(UUM)==list$Var1[i]),which(colnames(UUM)==list$Var2[i])]==UU[i]
WUM[which(rownames(WUM)==list$Var1[i]),which(colnames(WUM)==list$Var2[i])]==WU[i]

AGEM[which(rownames(AGEM)==list$Var1[i]),which(colnames(AGEM)==list$Var2[i])]==age[i]
SEXM[which(rownames(SEXM)==list$Var1[i]),which(colnames(SEXM)==list$Var2[i])]==sex[i]
TREATMM[which(rownames(TREATMM)==list$Var1[i]),which(colnames(TREATMM)==list$Var2[i])]==treatm[i]
yearM[which(rownames(yearM)==list$Var1[i]),which(colnames(yearM)==list$Var2[i])]==year[i]
nestM[which(rownames(nestM)==list$Var1[i]),which(colnames(nestM)==list$Var2[i])]==nest[i]


# add the names of both individuals participating in each dyad into the data frame
data.dyad$sampleA<-list$Var2
data.dyad$sampleB<-list$Var1
```

```r
# make a new key for the order of sample names and their associated individual IDs.
key2 <- data.frame(ID=sample_data(micdata)$ring_number, sampleA =sample_data(micdata)$identifier, sampleB
=sample_data(micdata)$identifier )

# merge individual ID to each sample name (sampleA -> IDA; sampleB -> IDB)
listdf = list(data.dyad, key2) # built list
data.dyad <- listdf %>% reduce(left_join, by ="sampleA", keep = FALSE) # merge based on sampleA column

data.dyad <- data.dyad[,-19] # delete column duplicate
data.dyad <- rename(data.dyad, IDA = ID, sampleB = sampleB.x) # change column names

listdf = list(data.dyad, key2) # update list
data.dyad <- listdf %>% reduce(left_join, by ="sampleB", keep = FALSE) # merge by sample B
data.dyad <- data.dyad[,-20] # delete column duplicate

data.dyad <- rename(data.dyad, IDB = ID, sampleA = sampleA.x) # change column names


# Make sure you have got rid of all self comparisons
data.dyad<-data.dyad[which(data.dyad$sampleA!=data.dyad$sampleB),]

#Save dyadic data file
saveRDS(data.dyad,"data_dyad.rds")
```
