# Supplementary material for: Early-life factors shaping the gut microbiota of Common buzzard nestlings
Source: Anim Microbiome. 2024 May 14;6:27. doi: 10.1186/s42523-024-00313-8 (PMC11092241; doi:10.1186/s42523-024-00313-8)
Supplement: Supplementary file 7 — Supplementary file1 (PDF 13894 kb) [file 42523_2024_313_MOESM7_ESM.pdf]

Beta diversity statistical analysis

Based on the workflow described here https://github.com/nuorenarra/Analysing-dyadic-data-with-brms

Table of Contents

Beta diversity statistical analysis
- Table of Contents

A) 16S rRNA beta diversity statistical analysis
- 1. Bray-Curtis dissimilarities
  - 1.1 Scale predictors between 0-1
  - 1.2 Model Bray-Curtis
  - 1.3 Model Diagnostics
    - 1.3.1 Compare distribution of response variable to distributions of predicted response variable values
  - 1.5 Model Summary
  - 1.4 Plot model posterior and credible intervals
  - 1.5 Plot model effects
- 2. Weighted UniFrac distances
  - 2.1 Model WU distances
  - 1.3 Model Diagnostics
    - 1.3.1 Compare distribution of response variable to distributions of predicted response variable values
  - 1.5 Model Summary
  - 1.4 Plot model posterior and credible intervals
  - 1.5 Plot model effects

B) 28S rRNA beta diversity statistical analysis
- 1. Bray-Curtis dissimilarities
  - 1.1. Scale predictors between 0-1
  - 1.2. Model Bray-Curtis
  - 1.3. Model Diagnostics
    - 1.3.1. Compare distribution of response variable to distributions of predicted response variable values
  - 1.4. Model Summary
  - 1.5. Plot model posterior and credible intervals
  - 1.6. Plot model effects
- 2. Weighted UniFrac distances
  - 2.1. Scale predictors between 0-1
  - 2.2. Model WU distances
  - 2.3. Model Diagnostics
    - 2.3.1. Compare distribution of response variable to distributions of predicted response variable values
  - 2.4. Model Summary
  - 2.5. Plot model posterior and credible intervals
  - 2.6. Plot model effects

A) 16S rRNA beta diversity statistical analysis

1. Bray-Curtis dissimilarities

```
#Load Packages

library(brms)
library(rstan)
library(parallel)
library(bayesplot)
library(ggplot2)

#Read in the data
data.dyad <- readRDS("data_dyad.rds")
```

Data-dyad is composed of 26,335 pairwise comparisons.

1.1 Scale predictors between 0-1

```
#scale all predictors to range between 0-1 if they are not already naturally on that scale

#define scaling function:

range.use <- function(x,min.use,max.use){ (x - min(x,na.rm=T))/(max(x,na.rm=T)-min(x,na.rm=T)) * (max.use -
min.use) + min.use }

scalecols<-c("age_diference","bci_diference")

for(i in 1:ncol(data.dyad[,which(colnames(data.dyad)%in%scalecols)])){
  data.dyad[,which(colnames(data.dyad)%in%scalecols)][,i]<-
range.use(data.dyad[,which(colnames(data.dyad)%in%scalecols)][,i],0,1)
}

data.dyad$sex_sim <-factor(data.dyad$sex_sim, levels=c("0","1"))
data.dyad$nest_sim <-factor(data.dyad$nest_sim, levels=c("0","1"))
data.dyad$year_sim <-factor(data.dyad$year_sim, levels=c("0","1"))
data.dyad$lbinom_comp <-factor(data.dyad$lbinom_comp, levels=c("II","NiNi","NiI"))
data.dyad$habitat_sim<-factor(data.dyad$habitat_sim, levels=c("0","1"))
data.dyad$sampleA <-as.factor(data.dyad$sampleA)
data.dyad$sampleB <-as.factor(data.dyad$sampleB)
data.dyad$IDA <-as.factor(data.dyad$IDA)
data.dyad$IDB <-as.factor(data.dyad$IDB)
```

1.2 Model Bray-Curtis

```
ncores = detectCores()
options(mc.cores = parallel::detectCores())

model_BC <- brm(BC_dissim~1+ age_diference + bci_diference + nest_sim + year_sim + sex_sim + habitat_sim +
lbinom_comp (1|mm(sampleA,sampleB)) + (1|mm(IDA,IDB)),
            data = data.dyad,
            family= "Beta",
            warmup = 10000, iter = 20000,
            cores = ncores, chains = 4,
            init=0)

saveRDS(model_BC, "model_BC.rds")

# Read in model
model_BC <- readRDS("model_final.rds")
```

1.3 Model Diagnostics

```
plot(model_BC)
```

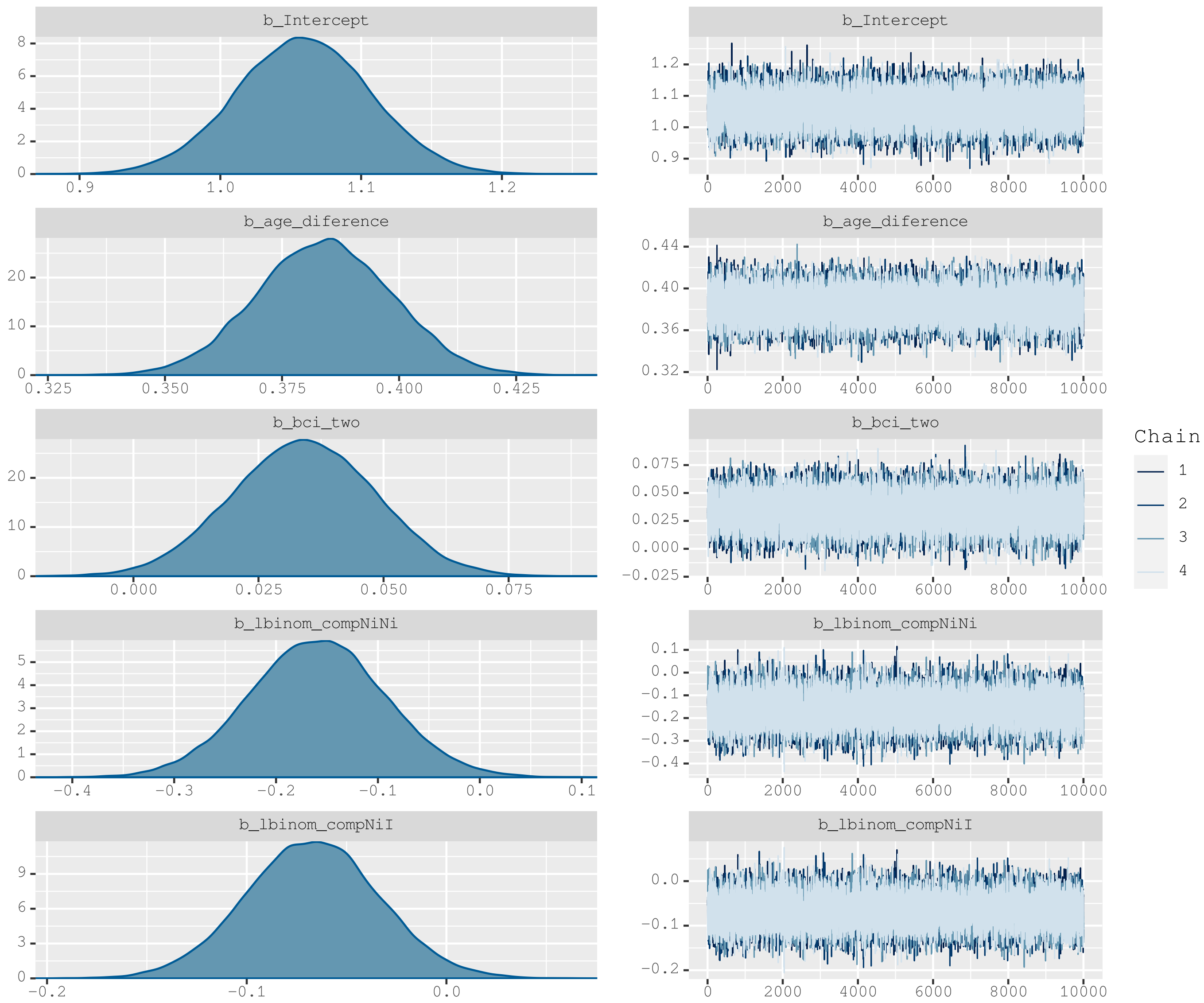

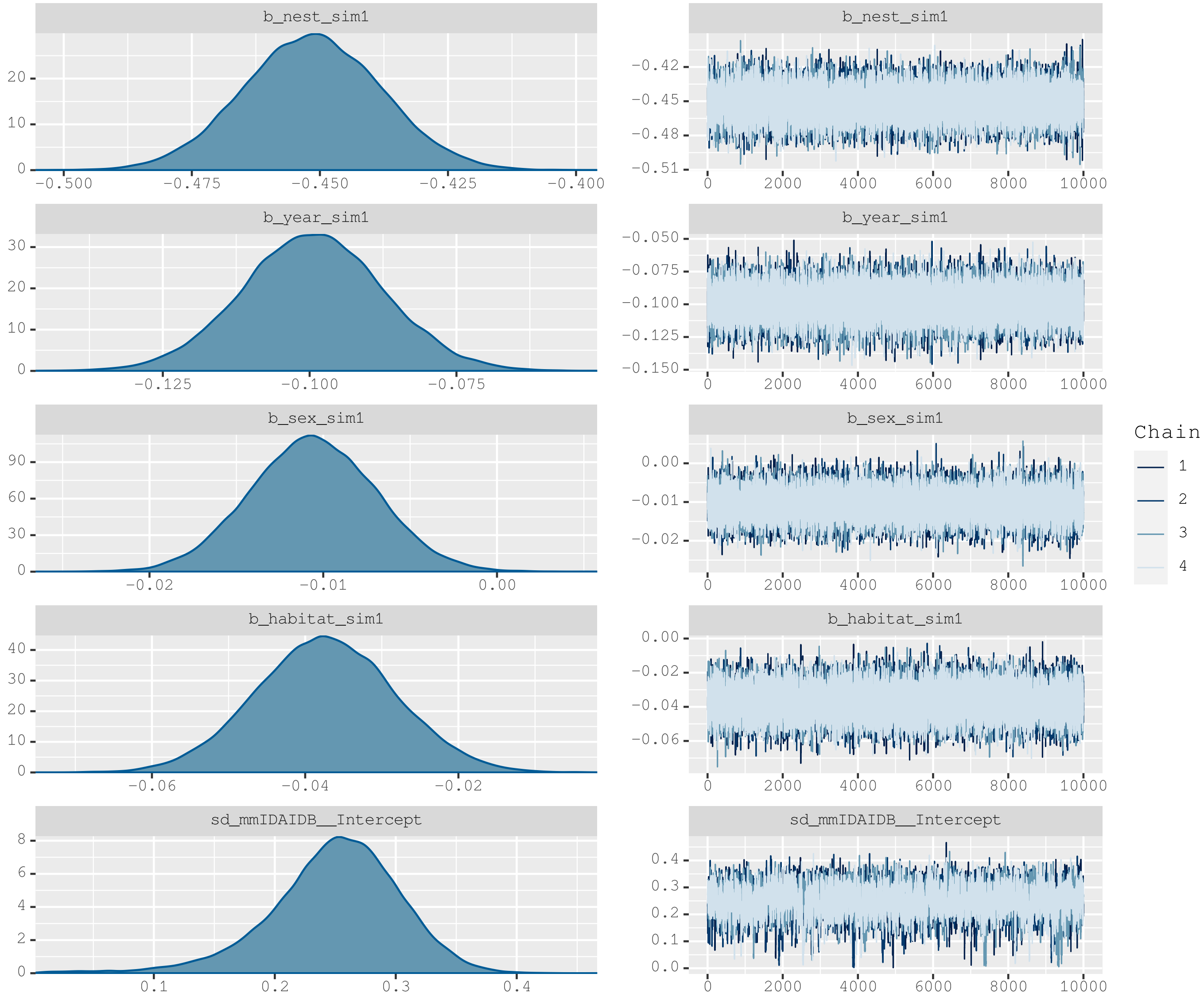

b_nest_sim1
b_year_sim1
b_sex_sim1
b_habitat_sim1
sd_mmIDAIDB__Intercept
Chain
1
2
3
4
-0.500
-0.475
-0.450
-0.425
-0.400
-0.125
-0.100
-0.075
-0.02
-0.01
0.00
-0.06
-0.04
-0.02
0.1
0.2
0.3
0.4
0
2000
4000
6000
8000
10000

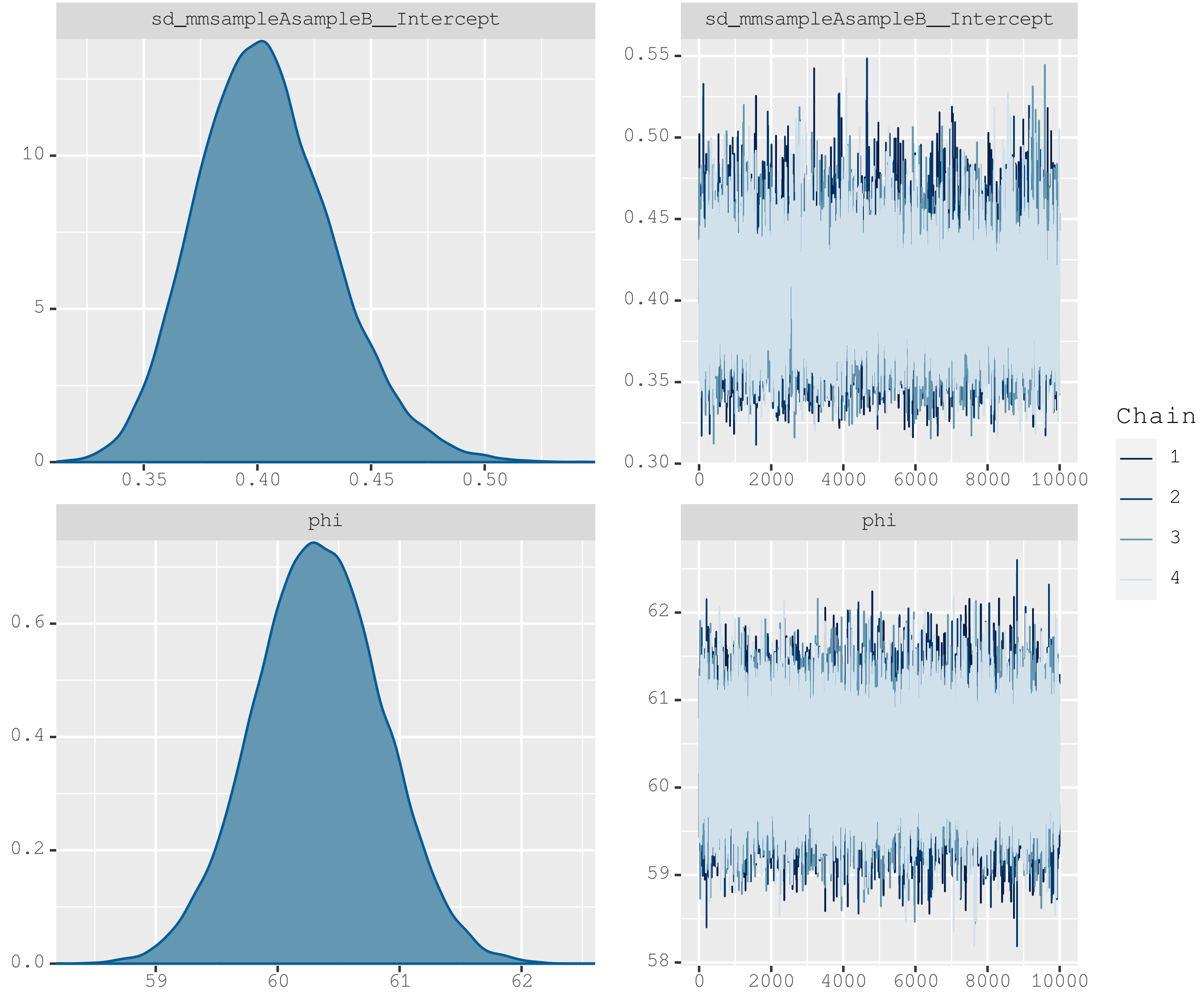

1.3.1 Compare distribution of response variable to distributions of predicted response variable values

```
pp_model_BC <-pp_check(model_BC, ndraws = 100)
pp_model_final
```

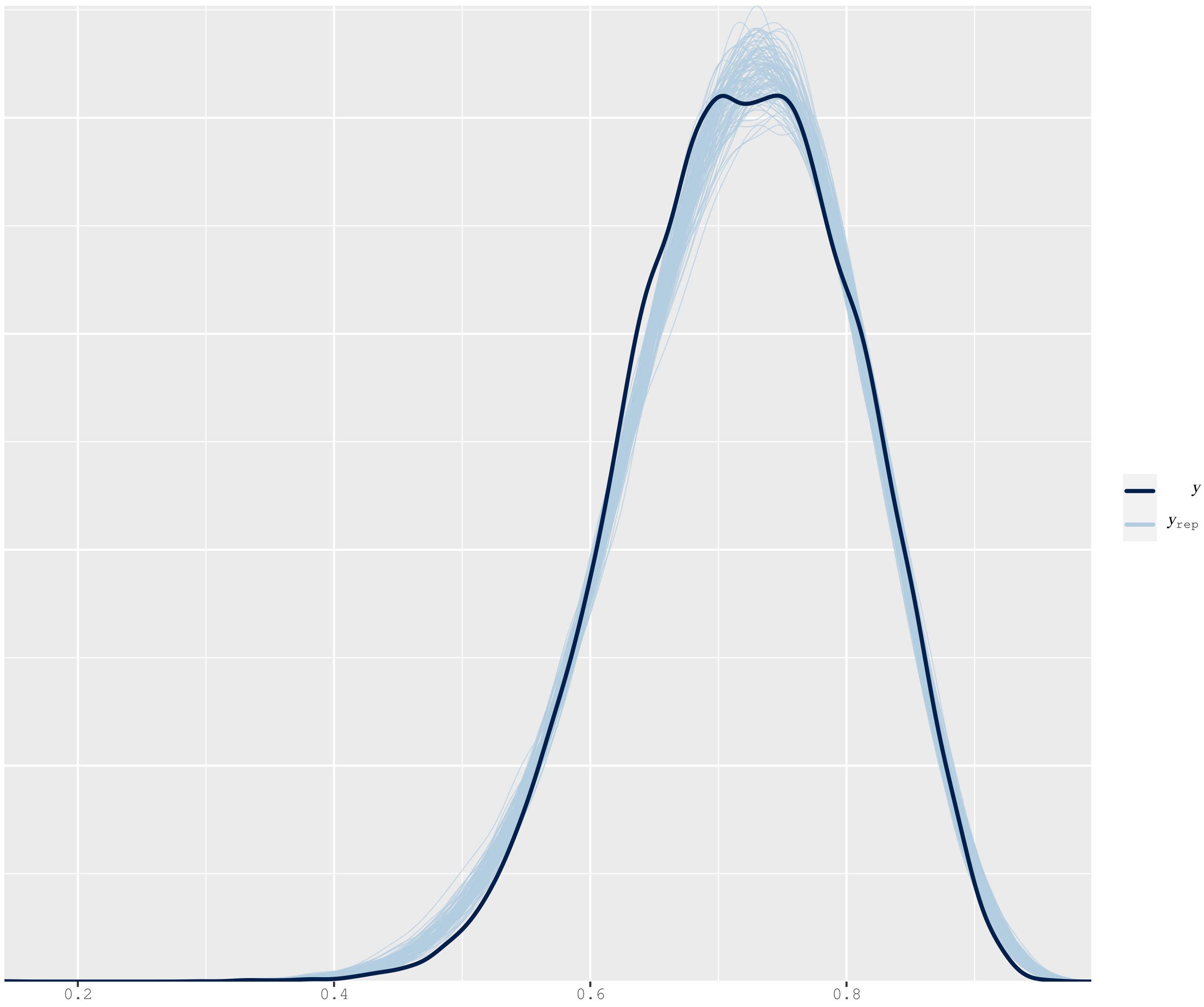

1.5 Model Summary

```
summary(model_BC)

Family: beta
  Links: mu = logit; phi = identity
Formula: BC_dissim ~ 1 + age_diference + bci_diference + lbinom_comp + nest_sim + year_sim + sex_sim +
habitat_sim + (1 | mm(sampleA, sampleB)) + (1 | mm(IDA, IDB))
   Data: data.dyad (Number of observations: 25425)
  Draws: 4 chains, each with iter = 20000; warmup = 10000; thin = 1;
         total post-warmup draws = 40000

Group-Level Effects:
~mmIDAIDB (Number of levels: 117)
              Estimate Est.Error   l-95% CI  u-95% CI   Rhat   Bulk_ESS  Tail_ESS
sd(Intercept)     0.25      0.05     0.13       0.34     1.00     2990     2652

~mmsampleAsampleB (Number of levels: 226)
              Estimate Est.Error  l-95% CI   u-95% CI   Rhat   Bulk_ESS  Tail_ESS
sd(Intercept)     0.40     0.03      0.35       0.47     1.00     5918      8058

Population-Level Effects:
                Estimate   Est.Error  l-95% CI   u-95% CI    Rhat   Bulk_ESS    Tail_ESS
Intercept           1.06       0.05     0.97        1.15      1.00    35066       30616
```

```
age_diference        0.38      0.01     0.36        0.41      1.00    58577       25412
bci_diference        0.03      0.01     0.01        0.06      1.00    59493       25555
lbinom_compNiNi     -0.16      0.07    -0.29       -0.03      1.00    21670       26641
lbinom_compNiI      -0.07      0.03    -0.13       -0.00      1.00    21753       26841
nest_sim1           -0.45      0.01    -0.48       -0.43      1.00    59044       27921
year_sim1           -0.10      0.01    -0.12       -0.08      1.00    59803       26444
sex_sim1            -0.01      0.00    -0.02       -0.00      1.00    58233       26359
habitat_sim1        -0.04      0.01    -0.05       -0.02      1.00    55160       26715

Family Specific Parameters:
    Estimate Est.Error   l-95% CI  u-95% CI   Rhat   Bulk_ESS   Tail_ESS
phi    60.34      0.53     59.30      61.37     1.00     55129      25683

Draws were sampled using sampling(NUTS). For each parameter, Bulk_ESS
and Tail_ESS are effective sample size measures, and Rhat is the potential
scale reduction factor on split chains (at convergence, Rhat = 1).
```

1.4 Plot model posterior and credible intervals

```
plot1 <-mcmc_plot(model_final_lbinom, type = "intervals", prob = 0.90,
                  variable = c("b_age_diference","b_bci_diference", "b_lbinom_compNiNi", "b_lbinom_compNiI",
"b_nest_sim1", "b_year_sim1",                         "b_sex_sim1", "b_habitat_sim1"))

plot1 + theme_minimal() + geom_vline(xintercept = 0, linetype="dotted", color="blue")
```

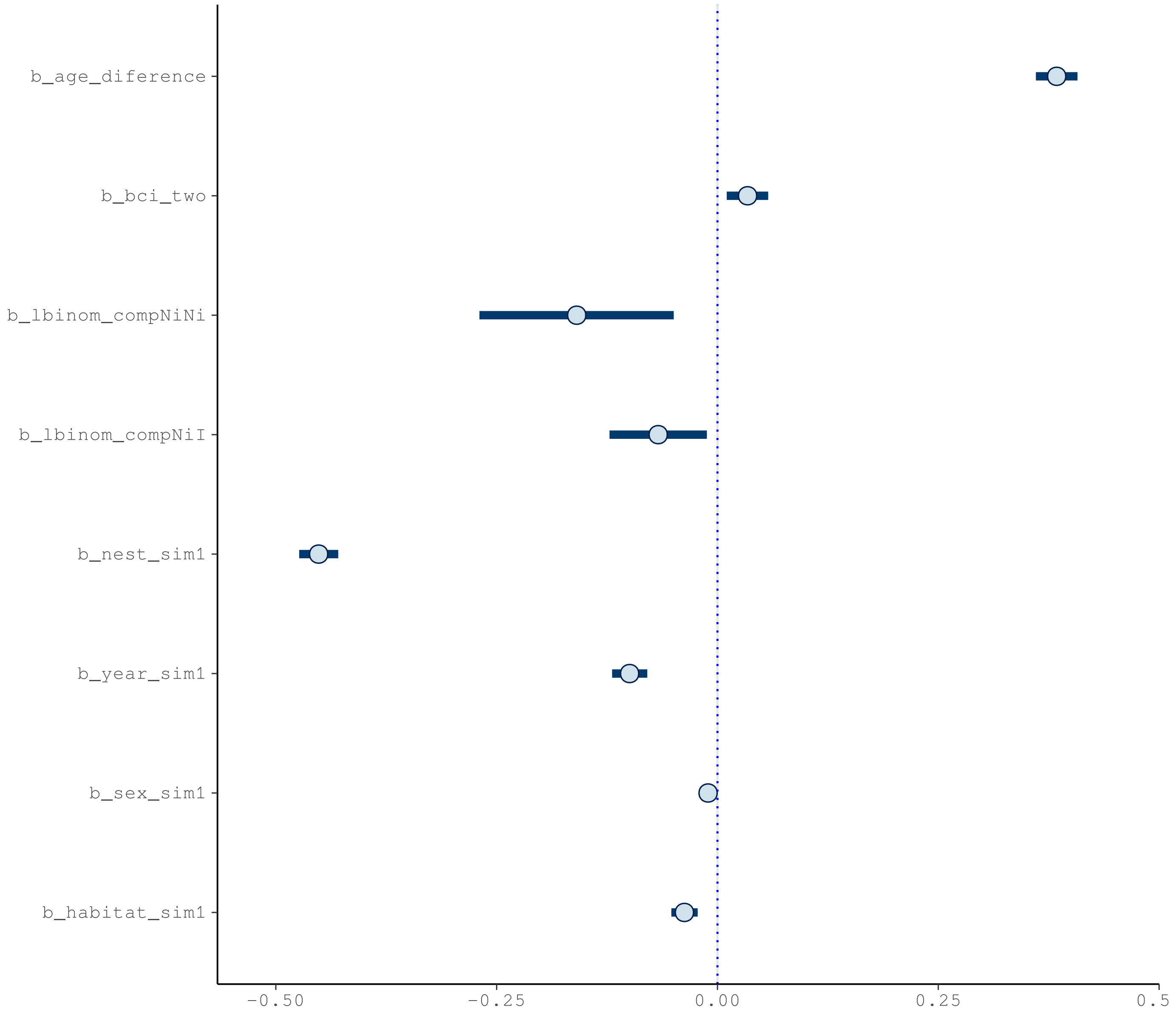

1.5 Plot model effects

Depicts the range of predicted Bray-Curtis dissimilarity values, does not depict confidence intervals.

```
conditional_effects(model_BC) # all effect plots for BC_dissmilarity
```

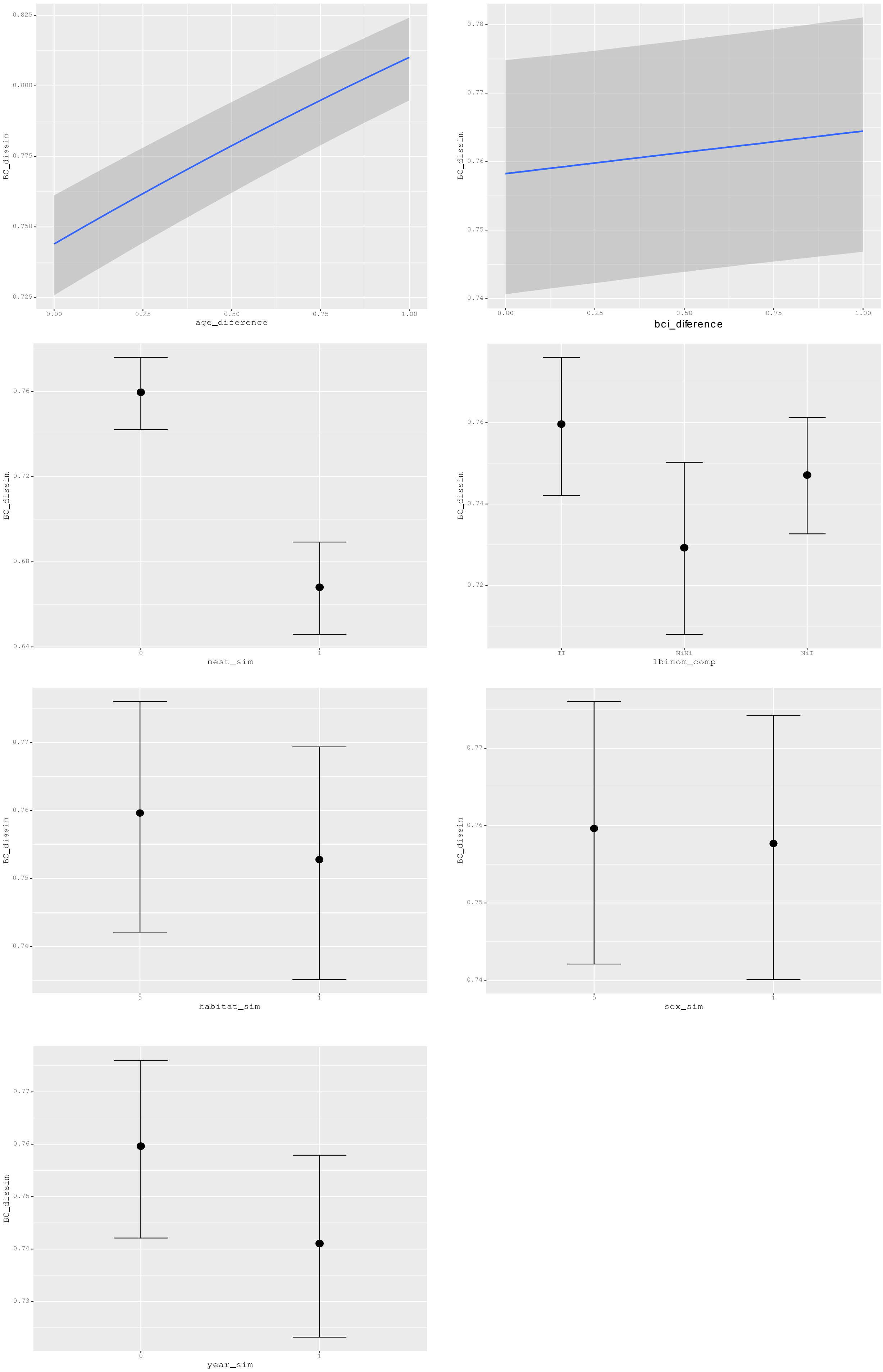

BC_dissim
0.825
0.800
0.775
0.750
0.725
0.00
0.25
0.50
0.75
1.00
age_diference
0.78
0.77
0.76
0.75
0.74
bci_diference
0.72
0.68
0.64
0
1
nest_sim
II
NiNi
NiI
lbinom_comp
0.73
habitat_sim
sex_sim
year_sim

2. Weighted UniFrac distances

2.1 Model WU distances

```
ncores = detectCores()
options(mc.cores = parallel::detectCores())

# In order to enhance model convergence, a uniform prior was applied
prior1 <- c(set_prior("normal(0,1)", class = "b", coef = "age_diference"),
            set_prior("normal(0,1)", class = "b", coef = "bci_diference"),
            set_prior("normal(0,1)", class = "b", coef = "nest_sim1"),
            set_prior("normal(0,1)", class = "b", coef = "year_sim1"),
            set_prior("normal(0,1)", class = "b", coef = "sex_sim1"),
            set_prior("normal(0,1)", class = "b", coef = "habitat_sim"),
            set_prior("normal(0,1)", class = "b", coef = "lbinom_compNiI"),
            set_prior("normal(0,1)", class = "b", coef = "lbinom_compNiNi"))

model_WU <- brm(WU_distance~1+ age_diference + bci_diference + nest_sim + year_sim + sex_sim + habitat_sim +
(1|mm(sampleA,sampleB)) + (1|mm(IDA,IDB)),
              data = data.dyad,
              family= "Beta",
              prior = prior1,
              warmup = 10000, iter = 20000,
              control = list(adapt_delta = 0.99, max_treedepth = 15),
              cores = ncores, chains = 4, init=0)

saveRDS(model_WU, "model_WU.rds")

# Read in the model
model_WU <- readRDS("model_WU.rds")
```

1.3 Model Diagnostics

```
plot(model_WU)
```

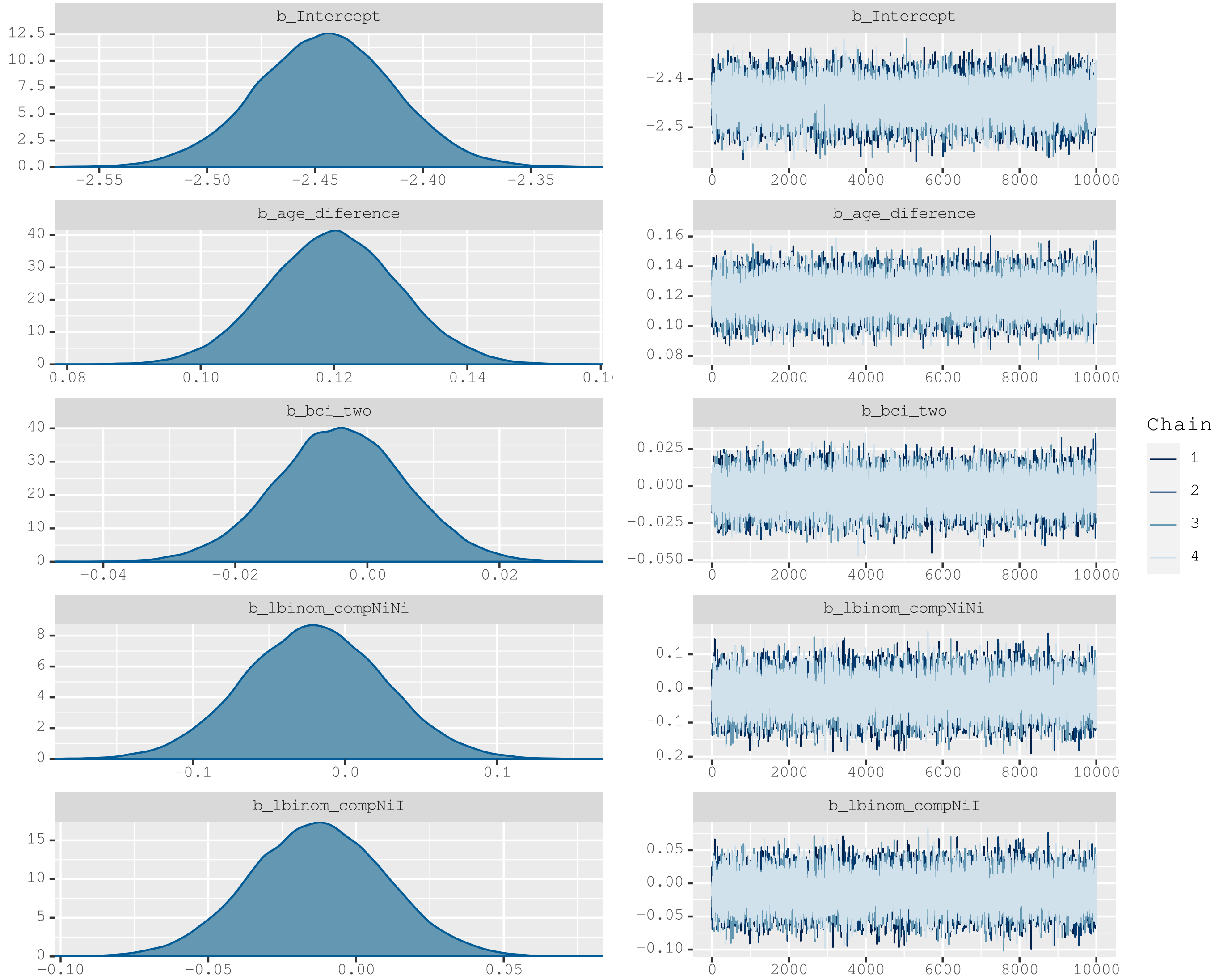

b_Intercept
b_age_diference
b_bci_two
b_lbinom_compNiNi
b_lbinom_compNiI
Chain
1
2
3
4

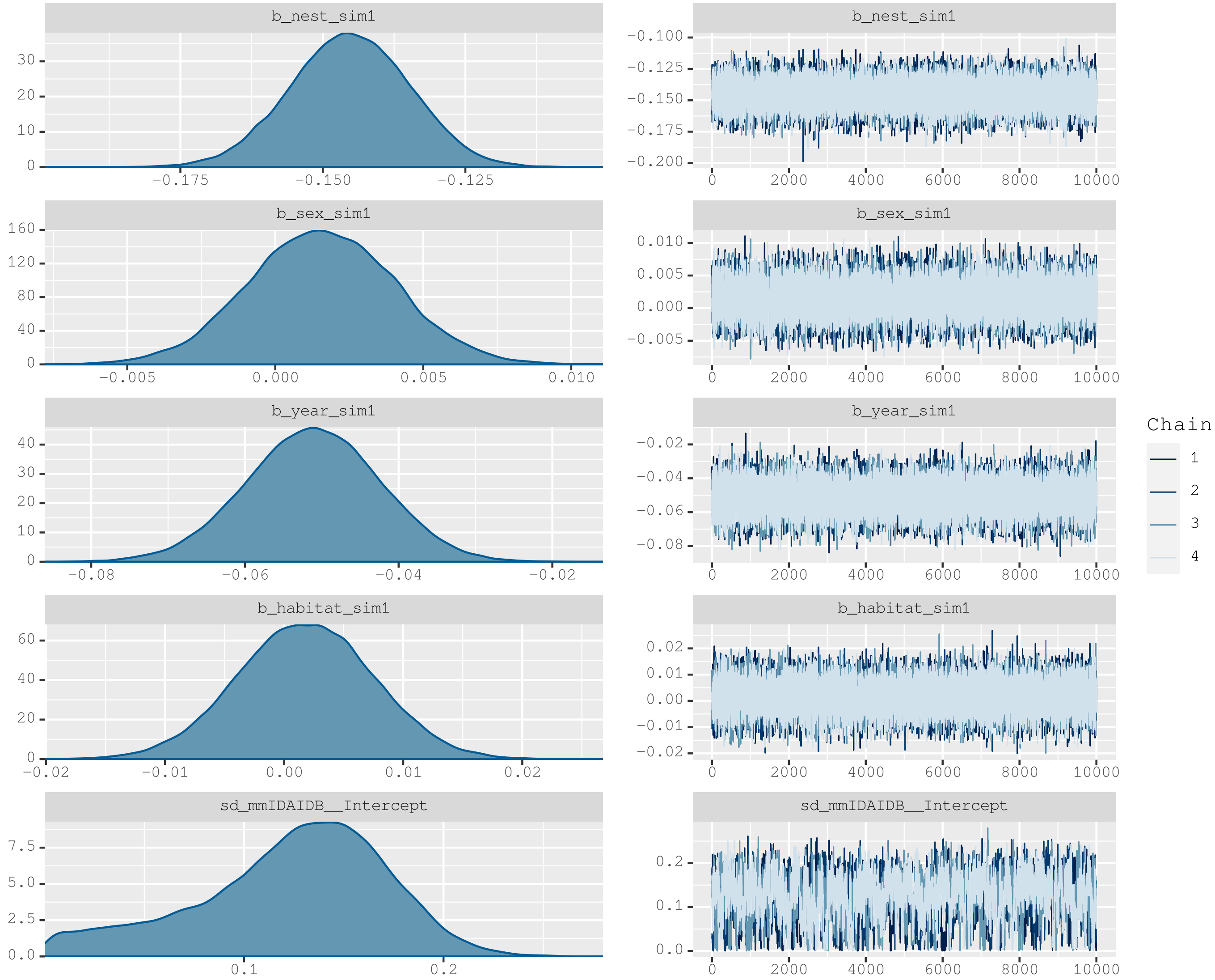

b_nest_sim1
b_sex_sim1
b_year_sim1
b_habitat_sim1
sd_mmIDAIDB__Intercept
Chain
1
2
3
4

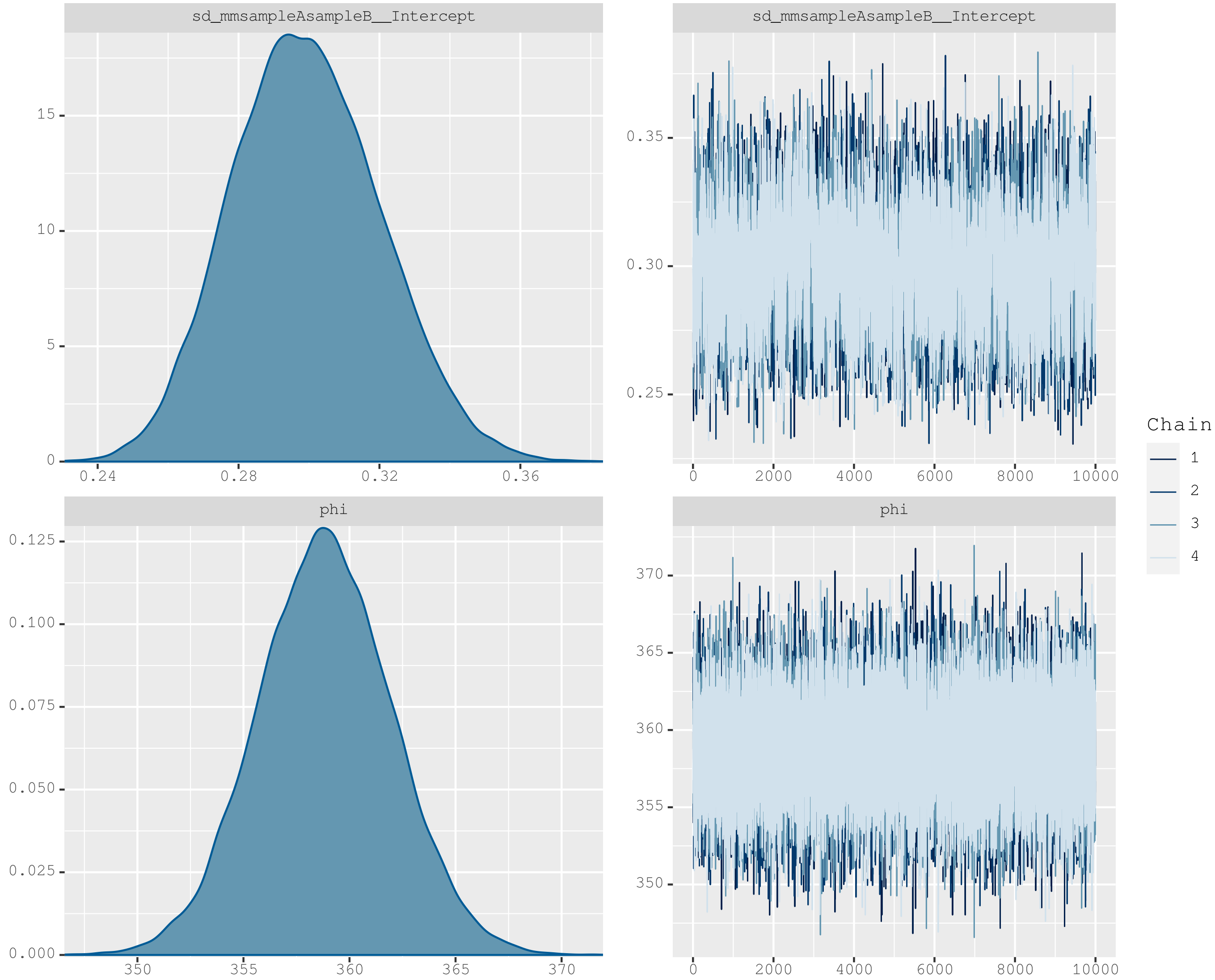

1.3.1 Compare distribution of response variable to distributions of predicted response variable values

```
pp_model_BC <-pp_check(model_WU, ndraws = 100)
pp_model_WU
```

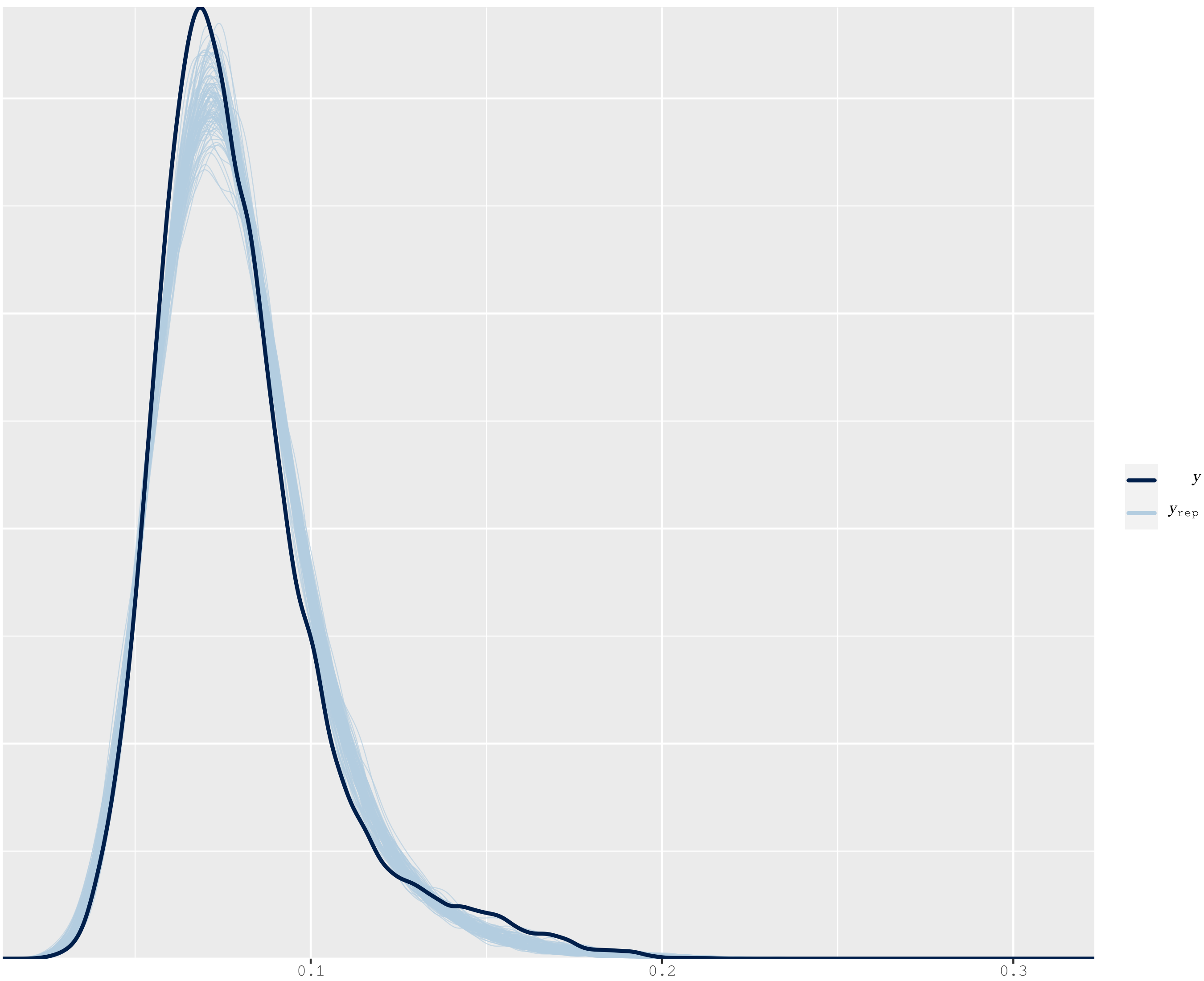

1.5 Model Summary

```
summary(model_WU)

Family: beta
  Links: mu = logit; phi = identity
Formula: WU_distance ~ 1 + age_diference + bci_diference + lbinom_comp + nest_sim + sex_sim + year_sim +
habitat_sim + (1 | mm(sampleA, sampleB)) + (1 | mm(IDA, IDB))
   Data: data.dyad (Number of observations: 25425)
  Draws: 4 chains, each with iter = 20000; warmup = 10000; thin = 1;
         total post-warmup draws = 40000

Group-Level Effects:
~mmIDAIDB (Number of levels: 117)
              Estimate  Est.Error  l-95% CI  u-95% CI  Rhat  Bulk_ESS  Tail_ESS
sd(Intercept)     0.12       0.05      0.01      0.20  1.00      1090      1738

~mmsampleAsampleB (Number of levels: 226)
              Estimate  Est.Error  l-95% CI  u-95% CI  Rhat  Bulk_ESS  Tail_ESS
sd(Intercept)     0.30       0.02      0.26      0.34  1.00      2450      7741

Population-Level Effects:
                Estimate  Est.Error  l-95% CI  u-95% CI  Rhat  Bulk_ESS  Tail_ESS
Intercept          -2.45       0.03     -2.51     -2.38  1.00     17900     25545
age_diference       0.12       0.01      0.10      0.14  1.00     67396     27034
bci_diference      -0.00       0.01     -0.02      0.02  1.00     66325     26781
lbinom_compNiNi    -0.02       0.05     -0.11      0.07  1.00     10353     17451
lbinom_compNiI     -0.01       0.02     -0.06      0.03  1.00     10461     17112
nest_sim1          -0.15       0.01     -0.17     -0.13  1.00     66733     28983
```

```
sex_sim1          0.00      0.00     -0.00      0.01     1.00     60472      27160
year_sim1        -0.05      0.01     -0.07     -0.03     1.00     69858      29156
habitat_sim1      0.00      0.01     -0.01      0.01     1.00     66748      28899

Family Specific Parameters:
    Estimate   Est.Error   l-95% CI   u-95% CI   Rhat   Bulk_ESS   Tail_ESS
phi   358.85      3.16      352.67     365.09    1.00     64829      27654

Draws were sampled using sampling(NUTS). For each parameter, Bulk_ESS
and Tail_ESS are effective sample size measures, and Rhat is the potential
scale reduction factor on split chains (at convergence, Rhat = 1).
```

1.4 Plot model posterior and credible intervals

```
plot1 <-mcmc_plot(model_WU, type = "intervals", prob = 0.95, pars= rownames(fixef(model_final))
[2:nrow(fixef(model_final))])
plot1 + theme_minimal() + geom_vline(xintercept = 0, linetype="dotted", color="blue")
```

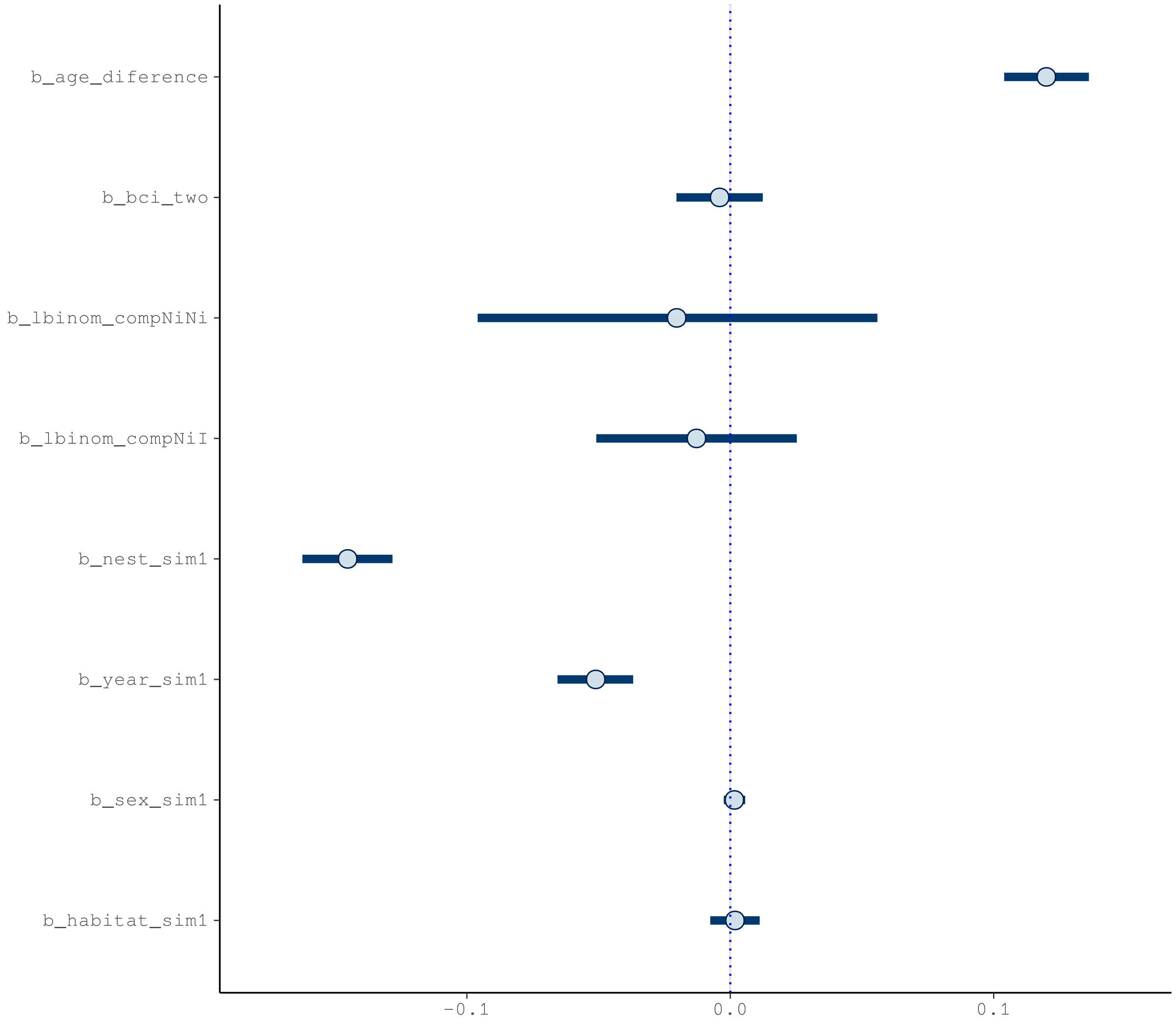

1.5 Plot model effects

Depicts the range of predicted Bray-Curtis dissimilarity values, does not depict confidence intervals.

```
conditional_effects(model_WU) # all effect plots for BC_dissmilarity
```

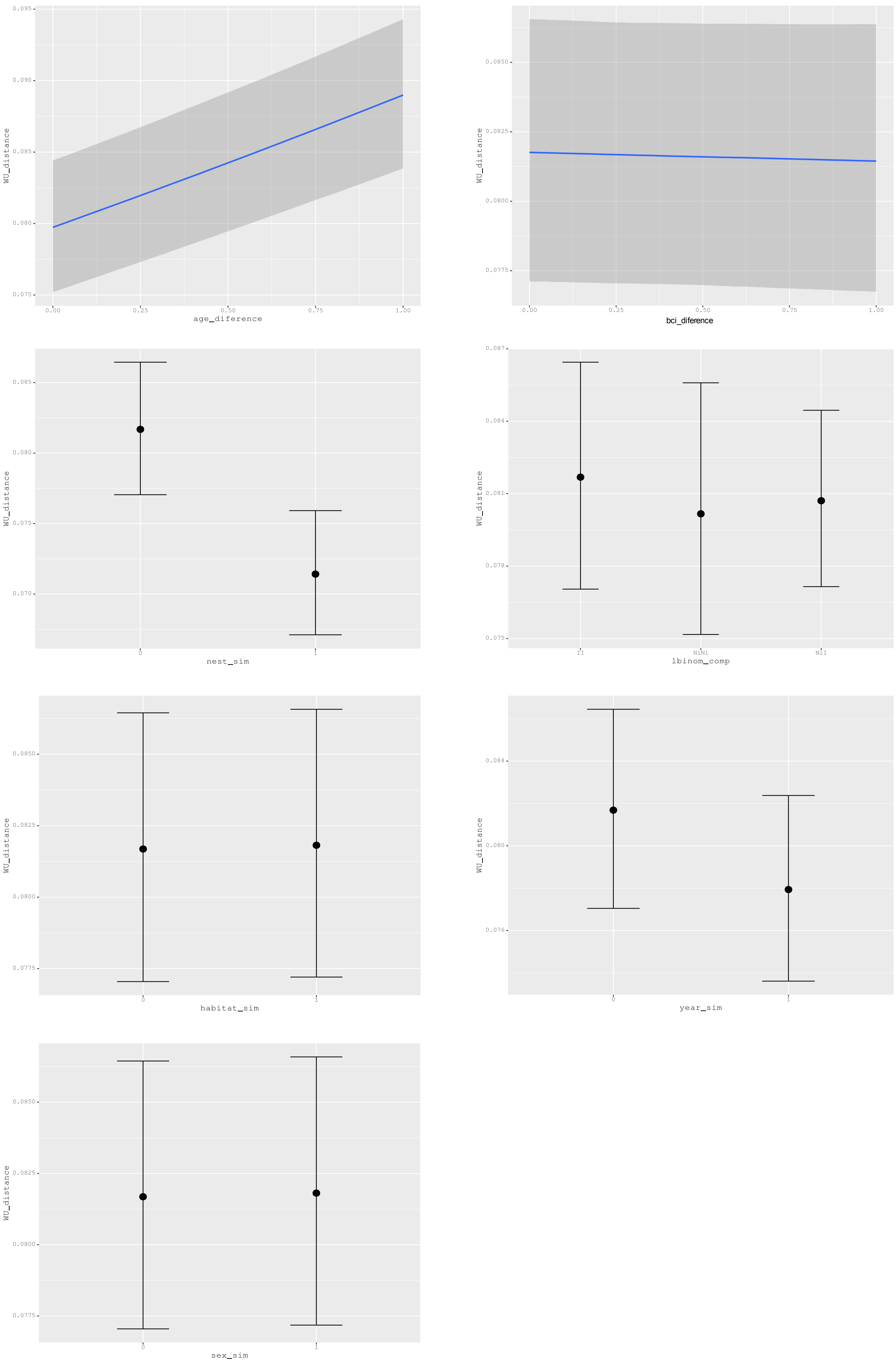

WU_distance
age_diference
0.075
0.080
0.085
0.090
0.095
0.00
0.25
0.50
0.75
1.00
bci_diference
0.0775
0.0800
0.0825
0.0850
nest_sim
0.070
0.075
0.080
0.085
0
1
lbinom_comp
0.075
0.078
0.081
0.084
0.087
II
NiNi
NiI
habitat_sim
year_sim
0.076
0.080
0.084
sex_sim

B) 28S rRNA beta diversity statistical analysis

1. Bray-Curtis dissimilarities

```
#Load Packages

library(brms)
library(rstan)
library(parallel)
library(bayesplot)
library(ggplot2)

#Read in the data
data.dyad <- readRDS("data_dyad.rds")
```

1.1. Scale predictors between 0-1

```
#scale all predictors to range between 0-1 if they are not already naturally on that scale

#define scaling function:

range.use <- function(x,min.use,max.use){ (x - min(x,na.rm=T))/(max(x,na.rm=T)-min(x,na.rm=T)) * (max.use -
min.use) + min.use }

scalecols<-c("age_diference","bci_diference")

for(i in 1:ncol(data.dyad[,which(colnames(data.dyad)%in%scalecols)])){
  data.dyad[,which(colnames(data.dyad)%in%scalecols)][,i]<-
range.use(data.dyad[,which(colnames(data.dyad)%in%scalecols)][,i],0,1)
}

data.dyad$sex_sim <-factor(data.dyad$sex_sim, levels=c("0","1"))
data.dyad$nest_sim <-factor(data.dyad$nest_sim, levels=c("0","1"))
data.dyad$year_sim <-factor(data.dyad$year_sim, levels=c("0","1"))
data.dyad$lbinom_comp <-factor(data.dyad$lbinom_comp, levels=c("II","NiNi","NiI"))
data.dyad$habitat_sim<-factor(data.dyad$habitat_sim, levels=c("0","1"))
data.dyad$sampleA <-as.factor(data.dyad$sampleA)
data.dyad$sampleB <-as.factor(data.dyad$sampleB)
data.dyad$IDA <-as.factor(data.dyad$IDA)
data.dyad$IDB <-as.factor(data.dyad$IDB)
```

1.2. Model Bray-Curtis

```
ncores = detectCores()
options(mc.cores = parallel::detectCores())


prior1 <- c(set_prior("normal(0,1)", class = "b", coef = "age_diference"),
            set_prior("normal(0,1)", class = "b", coef = "bci_diference"),
            set_prior("normal(0,1)", class = "b", coef = "nest_sim1"),
            set_prior("normal(0,1)", class = "b", coef = "year_sim1"),
            set_prior("normal(0,1)", class = "b", coef = "sex_sim1"),
            set_prior("normal(0,1)", class = "b", coef = "habitat_sim1"),
            set_prior("normal(0,1)", class = "b", coef = "lbinom_compNiI"),
```

```
            set_prior("normal(0,1)", class = "b", coef = "lbinom_compNiNi"))

model_final <- brm(BC_dissim~1+ age_diference + bci_diference + nest_sim + year_sim + sex_sim + habitat_sim +
lbinom_comp + (1|mm(sampleA,sampleB)) + (1|mm(IDA,IDB)),
              data = data.dyad,
              family= "zero_one_inflated_beta",
              prior = prior1,
              warmup = 10000, iter = 20000,
              control = list(adapt_delta = 0.99, max_treedepth = 15),
              cores = ncores, chains = 4, init=0)

saveRDS(model_final, "model_final.rds")

#Read in the model
model_final <- readRDS("model_final.rds")
```

1.3. Model Diagnostics

```
plot(model_BC)
```

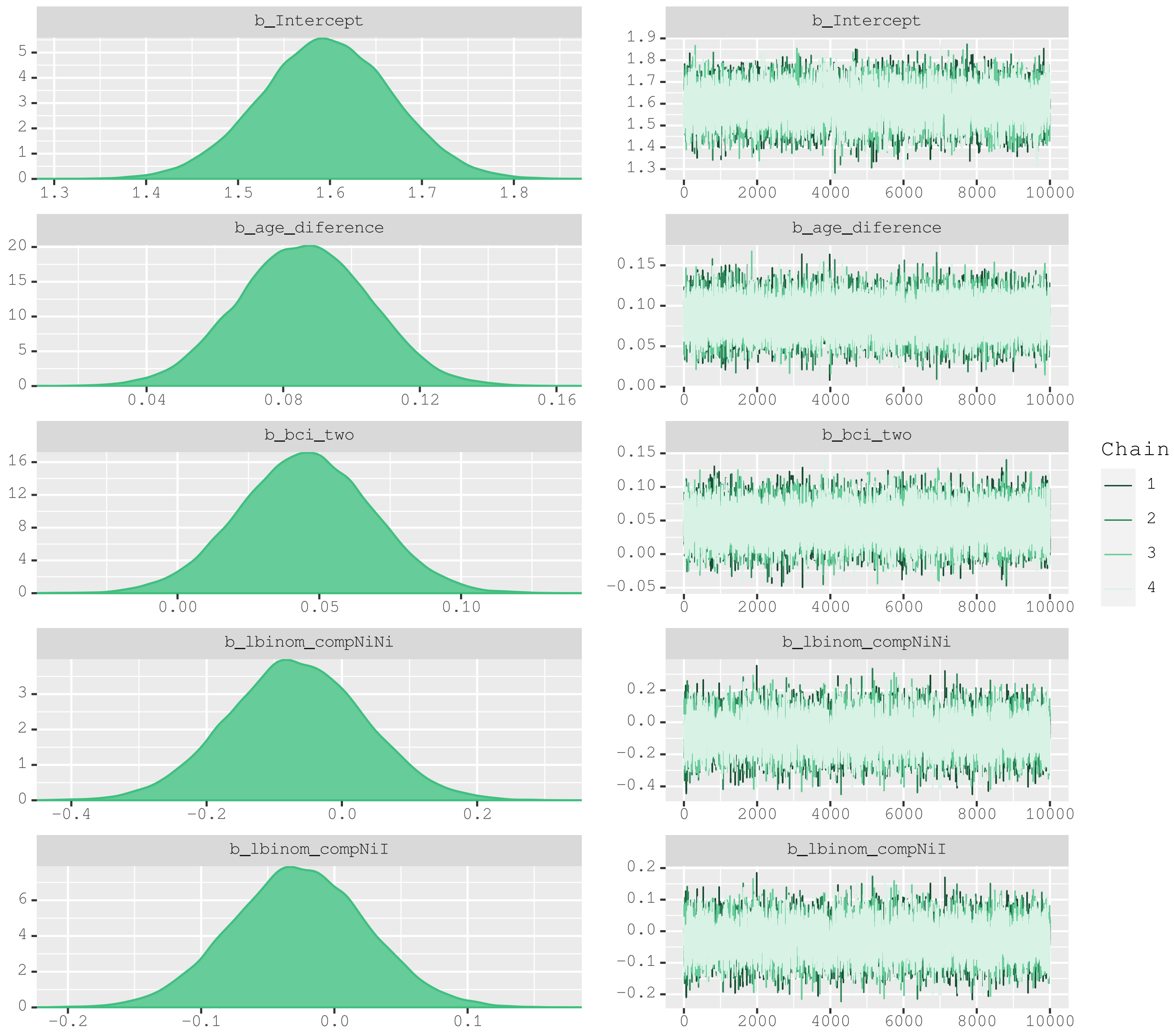

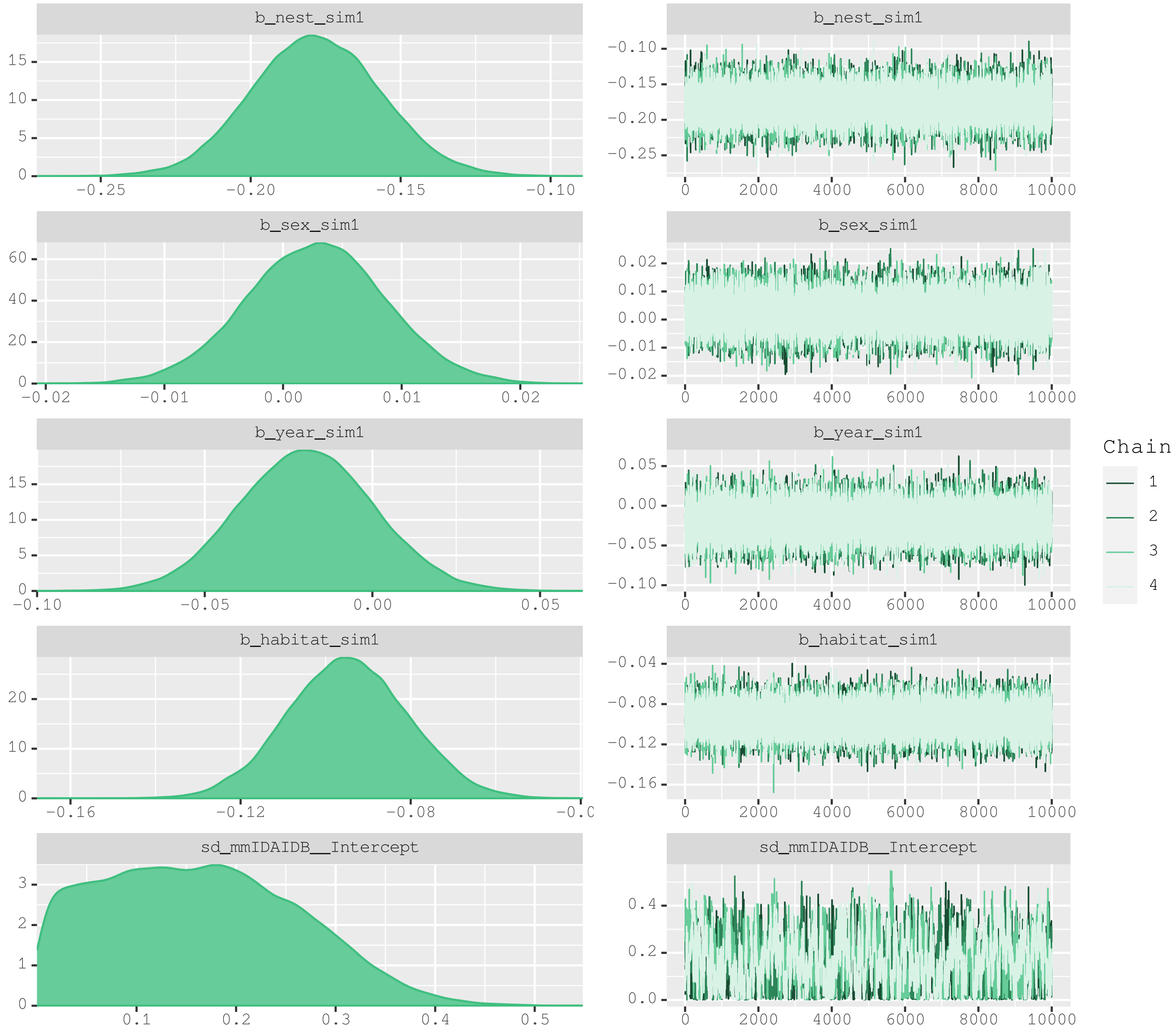
b_nest_sim1
b_sex_sim1
b_year_sim1
b_habitat_sim1
sd_mmIDAIDB__Intercept
Chain
1
2
3
4
0
2000
4000
6000
8000
10000

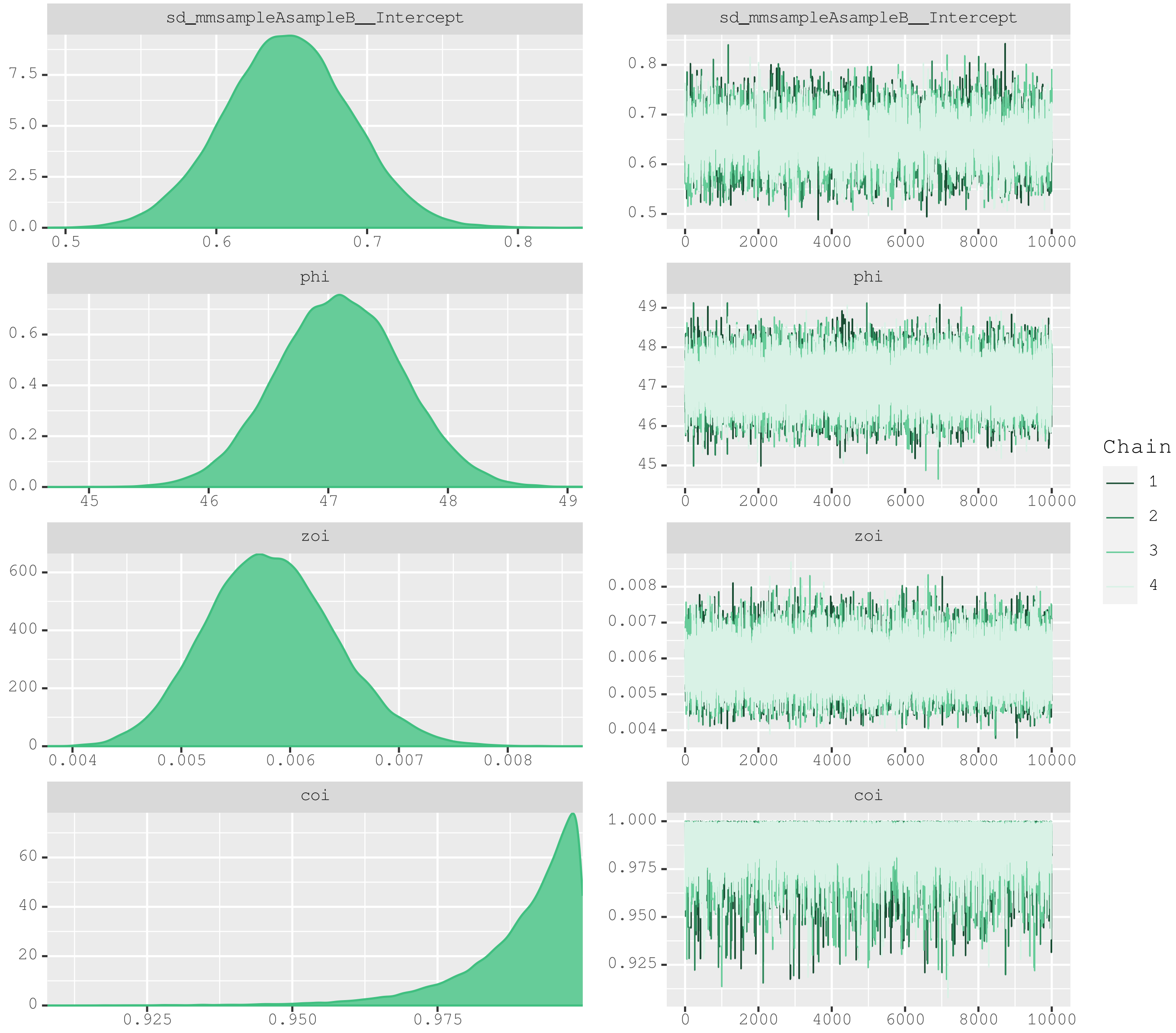

1.3.1. Compare distribution of response variable to distributions of predicted response variable values

```
pp_model_BC <-pp_check(model_BC, ndraws = 50)

pp_model_final
```

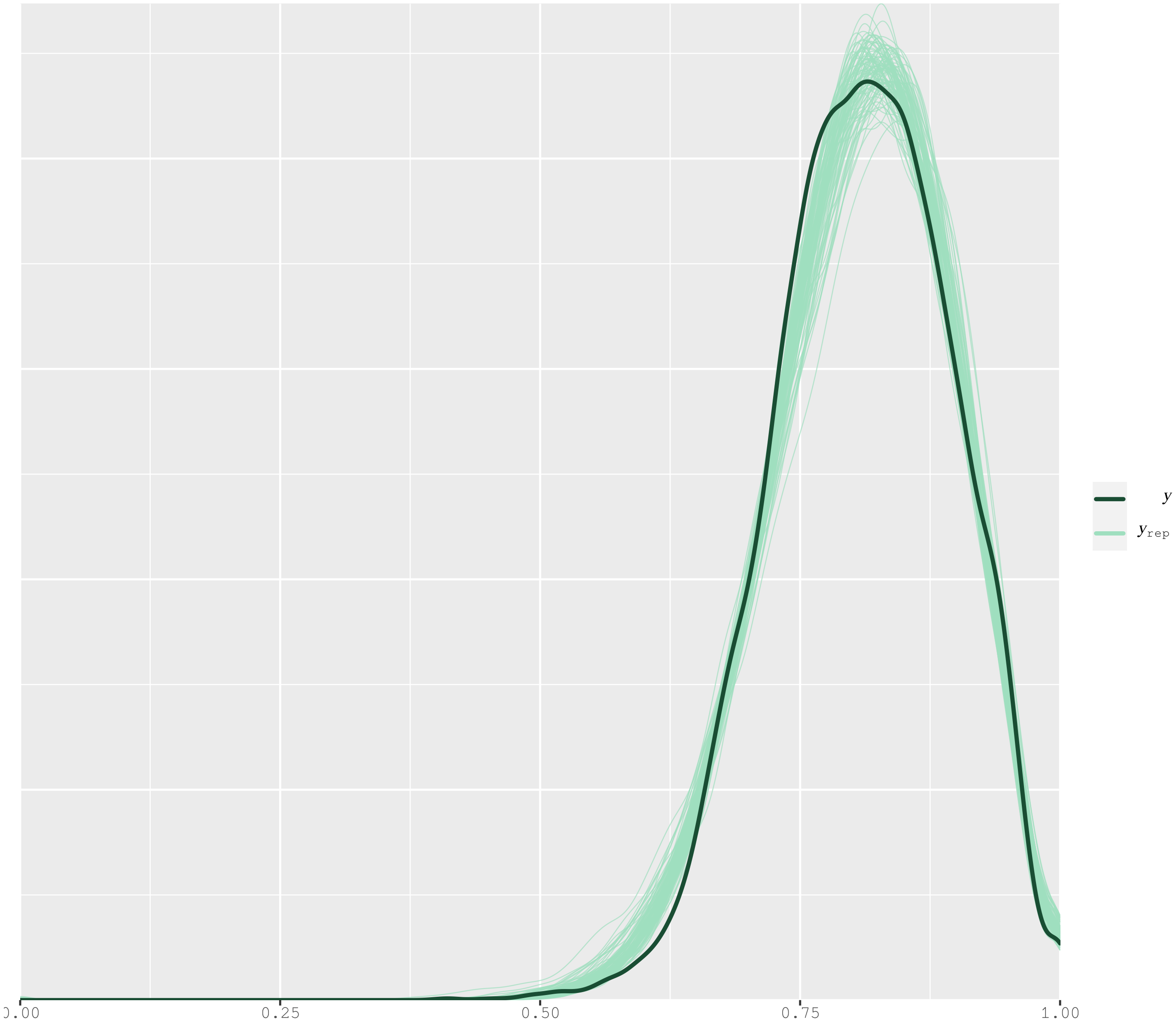

1.4. Model Summary

```
summary(model_BC)

Family: zero_one_inflated_beta
  Links: mu = logit; phi = identity; zoi = identity; coi = identity
Formula: BC_dissim ~ 1 + age_diference + bci_diference + lbinom_comp + nest_sim + sex_sim + year_sim +
habitat_sim + (1 | mm(sampleA, sampleB)) + (1 | mm(IDA, IDB))
   Data: data.dyad (Number of observations: 16471)
  Draws: 4 chains, each with iter = 20000; warmup = 10000; thin = 1;
         total post-warmup draws = 40000

Group-Level Effects:
~mmIDAIDB (Number of levels: 110)
              Estimate     Est.Error    l-95% CI     u-95% CI    Rhat    Bulk_ESS     Tail_ESS
sd(Intercept)     0.16         0.10        0.01         0.36      1.01      783          2381

~mmsampleAsampleB (Number of levels: 182)
              Estimate    Est.Error    l-95% CI     u-95% CI    Rhat    Bulk_ESS    Tail_ESS
sd(Intercept)     0.65        0.04         0.57         0.73     1.00      3000        6849

Population-Level Effects:
                Estimate     Est.Error    l-95% CI     u-95% CI     Rhat     Bulk_ESS     Tail_ESS
Intercept           1.60         0.07         1.45         1.74      1.00     10560          19316
```

```
age_diference       0.09      0.02       0.05       0.12      1.00     80195          27859
bci_two             0.05      0.02      -0.00       0.09      1.00     85005          26947
lbinom_compNiNi    -0.07      0.10      -0.27       0.13      1.00      8718          15364
lbinom_compNiI     -0.03      0.05      -0.13       0.07      1.00      8771          15815
nest_sim1          -0.18      0.02      -0.22      -0.14      1.00     79725          29045
sex_sim1            0.00      0.01      -0.01       0.01      1.00     78734          26540
year_sim1          -0.02      0.02      -0.06       0.02      1.00     72869          31147
habitat_sim1       -0.09      0.01      -0.12      -0.07      1.00     71270          28517

Family Specific Parameters:
    Estimate    Est.Error    l-95% CI    u-95% CI    Rhat    Bulk_ESS    Tail_ESS
phi    47.10      0.52         46.09       48.12      1.00       81005       27166
zoi     0.01      0.00          0.00        0.01      1.00       74765       26599
coi     0.99      0.01          0.96        1.00      1.00       46936       21236

Draws were sampled using sampling(NUTS). For each parameter, Bulk_ESS
and Tail_ESS are effective sample size measures, and Rhat is the potential
scale reduction factor on split chains (at convergence, Rhat = 1).
```

1.5. Plot model posterior and credible intervals

```
plot1 <-mcmc_plot(model_WU, type = "intervals", prob = 0.90, variable = c("b_age_diference","b_bci_diference",
"b_lbinom_compNiNi",
                                                         "b_lbinom_compNiI", "b_nest_sim1", "b_year_sim1",
"b_sex_sim1", "b_habitat_sim1"))

plot1 + theme_default() + geom_vline(xintercept = 0, linetype="dotted", color="black")
```

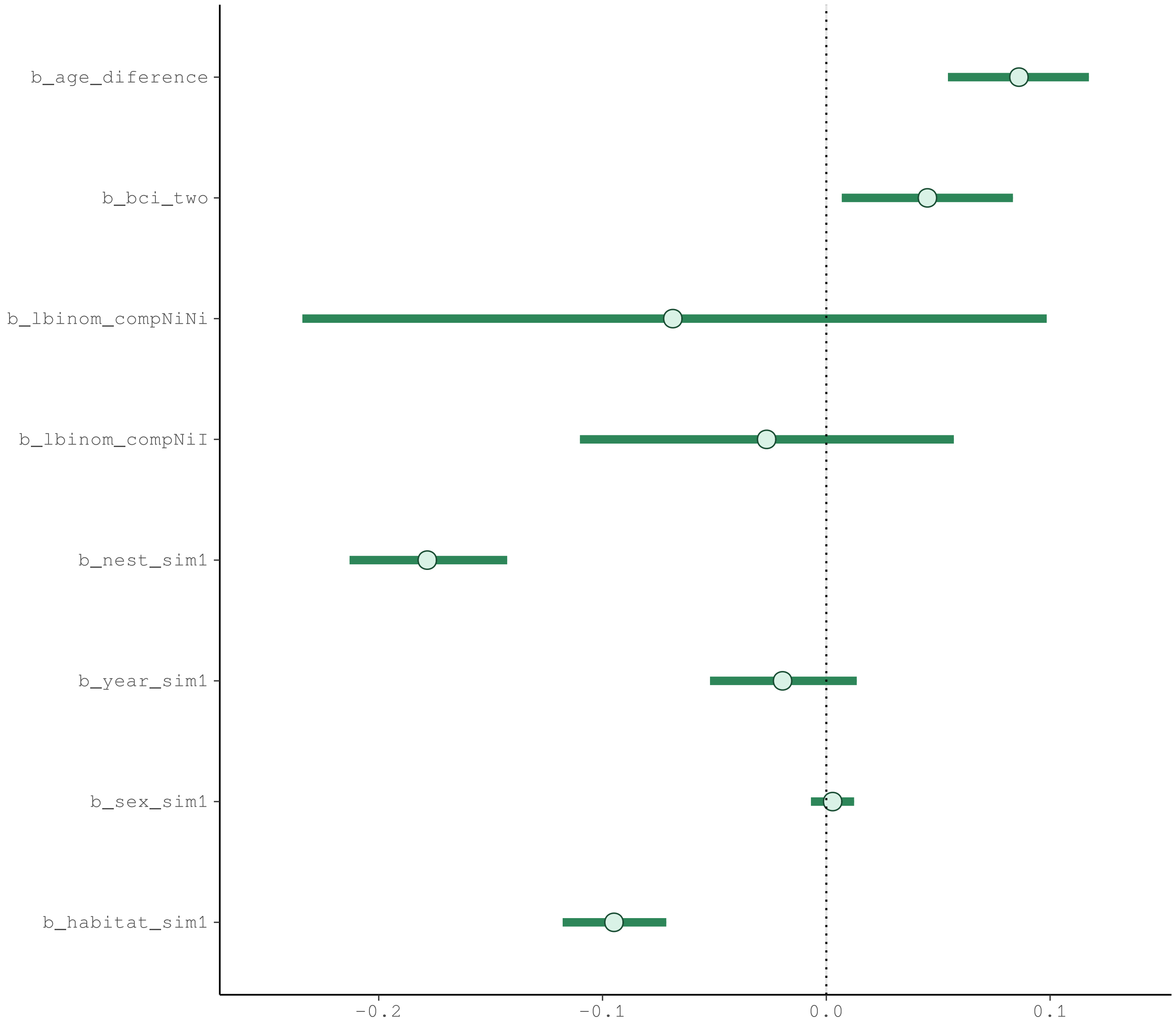

1.6. Plot model effects

Depicts the range of predicted Bray-Curtis dissimilarity values, does not depict confidence intervals.

```
conditional_effects(model_BC)
```

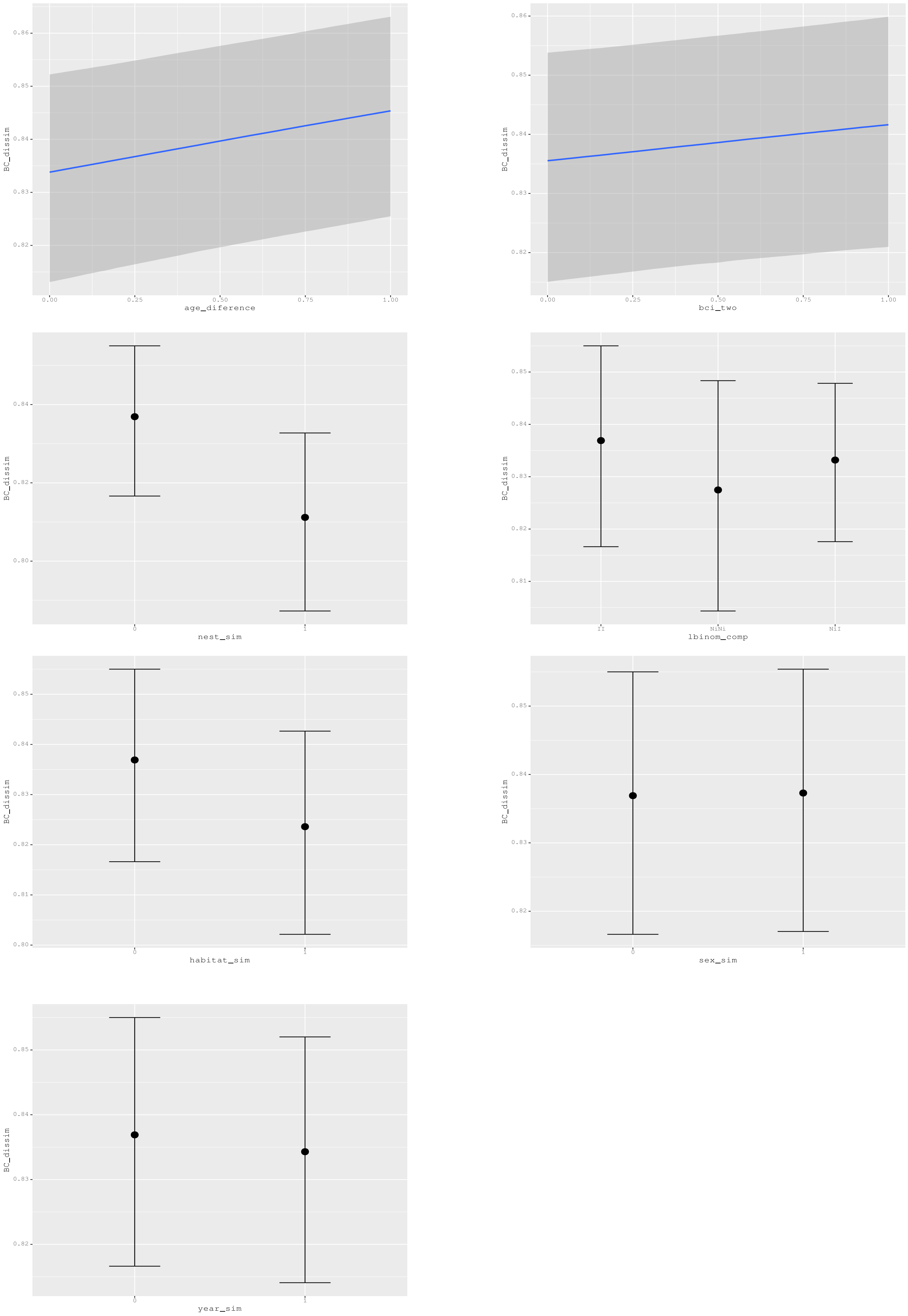

BC_dissim
0.86
0.85
0.84
0.83
0.82
0.00
0.25
0.50
0.75
1.00
age_diference
bci_two
0.80
0.81
0
1
nest_sim
II
NiNi
NiI
lbinom_comp
habitat_sim
sex_sim
year_sim

2. Weighted UniFrac distances

```
#Load Packages

library(brms)
library(rstan)
library(parallel)
library(bayesplot)
library(ggplot2)

#Read in the data
data.dyad <- readRDS("data_dyad.rds")
```

Data dyad composed of 17,020 pairwise comparisons.

2.1. Scale predictors between 0-1

```
#scale all predictors to range between 0-1 if they are not already naturally on that scale

#define scaling function:

range.use <- function(x,min.use,max.use){ (x - min(x,na.rm=T))/(max(x,na.rm=T)-min(x,na.rm=T)) * (max.use -
min.use) + min.use }

scalecols<-c("age_diference","bci_diference","sampling_time")

for(i in 1:ncol(data.dyad[,which(colnames(data.dyad)%in%scalecols)])){
  data.dyad[,which(colnames(data.dyad)%in%scalecols)][,i]<-
range.use(data.dyad[,which(colnames(data.dyad)%in%scalecols)][,i],0,1)
}

data.dyad$sex_sim <-factor(data.dyad$sex_sim, levels=c("0","1"))
data.dyad$nest_sim <-factor(data.dyad$nest_sim, levels=c("0","1"))
data.dyad$year_sim <-factor(data.dyad$year_sim, levels=c("0","1"))
data.dyad$lbinom_comp <-factor(data.dyad$lbinom_comp, levels=c("II","NiNi","NiI"))
data.dyad$habitat_sim<-factor(data.dyad$habitat_sim, levels=c("0","1"))
data.dyad$sampleA <-as.factor(data.dyad$sampleA)
data.dyad$sampleB <-as.factor(data.dyad$sampleB)
data.dyad$IDA <-as.factor(data.dyad$IDA)
data.dyad$IDB <-as.factor(data.dyad$IDB)
```

2.2. Model WU distances

```
ncores = detectCores()
options(mc.cores = parallel::detectCores())

# In order to enhance model convergence, a uniform prior was applied
prior1 <- c(set_prior("normal(0,1)", class = "b", coef = "age_diference"),
            set_prior("normal(0,1)", class = "b", coef = "bci_diference"),
            set_prior("normal(0,1)", class = "b", coef = "nest_sim1"),
            set_prior("normal(0,1)", class = "b", coef = "year_sim1"),
            set_prior("normal(0,1)", class = "b", coef = "sex_sim1"),
            set_prior("normal(0,1)", class = "b", coef = "habitat_sim"),
            set_prior("normal(0,1)", class = "b", coef = "lbinom_compNiI"),
            set_prior("normal(0,1)", class = "b", coef = "lbinom_compNiNi"))
```

```
model_final <- brm(WU_distance~1+ age_diference + bci_diference + nest_sim + year_sim + sex_sim + habitat_sim
+lbinom_comp (1|mm(sampleA,sampleB)) + (1|mm(IDA,IDB)),
              data = data.dyad,
              family= "Beta",
              prior = prior1,
              warmup = 10000, iter = 20000,
              control = list(adapt_delta = 0.99, max_treedepth = 15),
              cores = ncores, chains = 4, init=0)

saveRDS(model_WU, "model_WU.rds")

# Read in the model
model_WU <- readRDS("model_WU.rds")
```

2.3. Model Diagnostics

```
plot(model_WU)
```

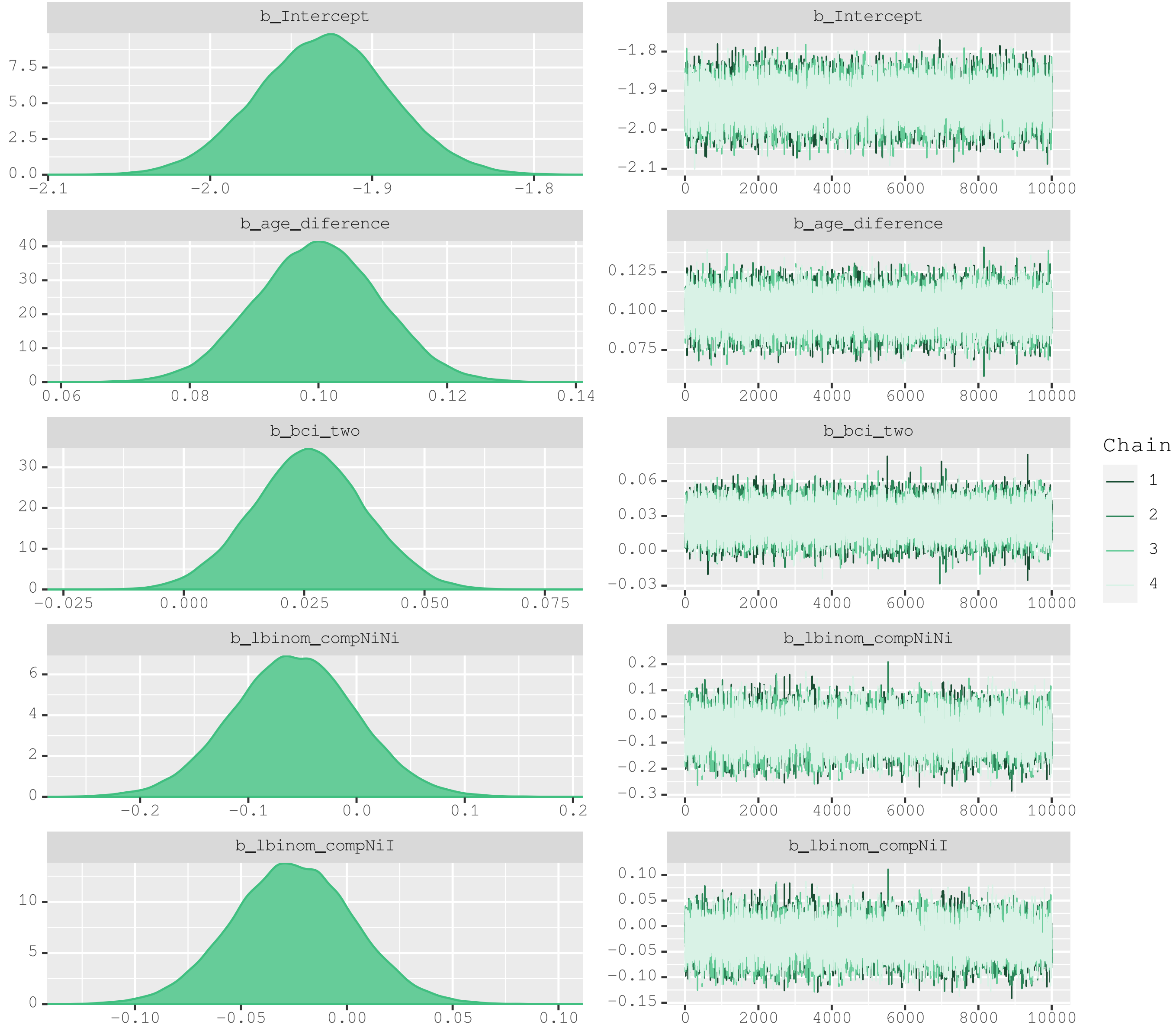

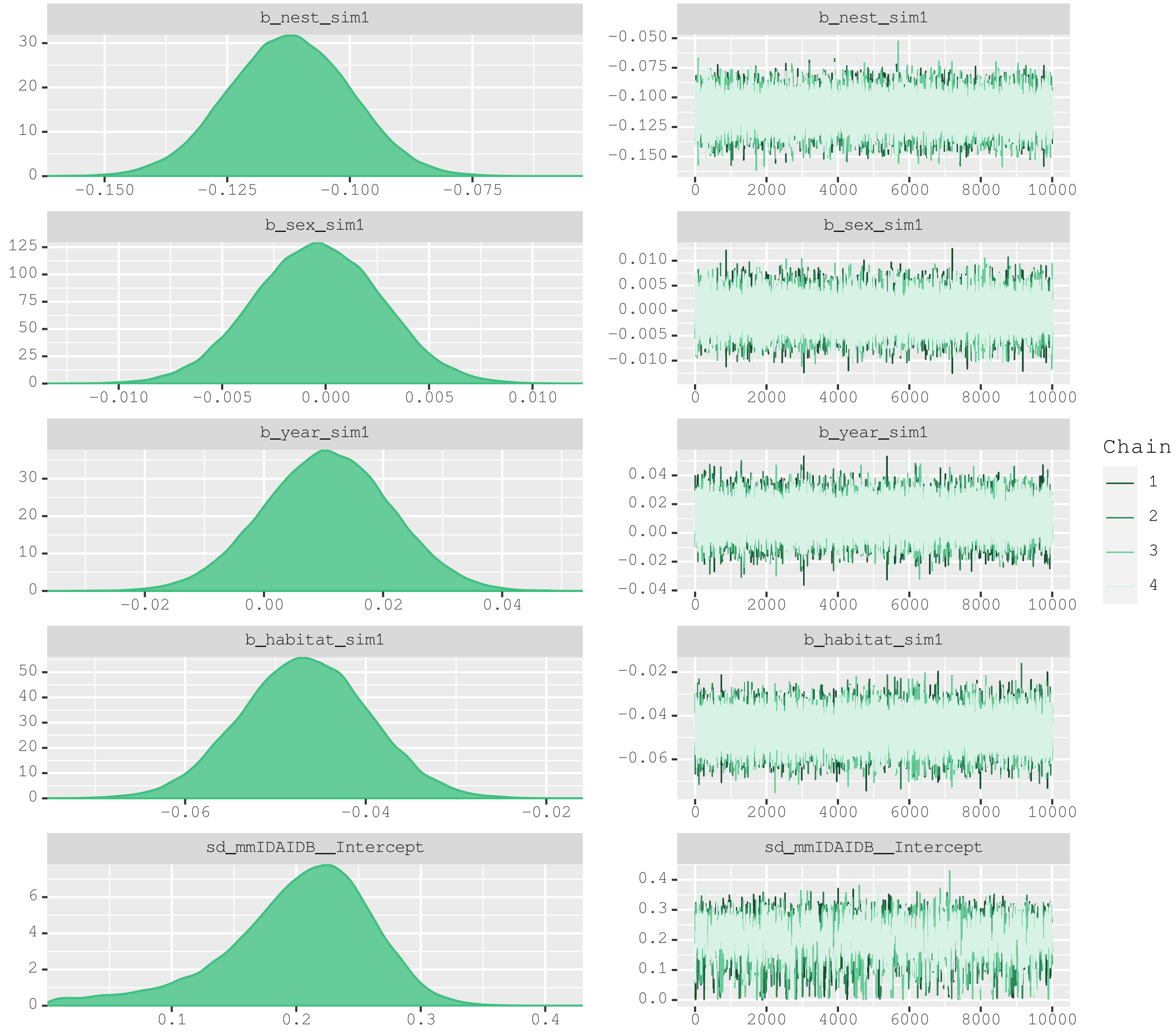
b_nest_sim1
b_sex_sim1
b_year_sim1
b_habitat_sim1
sd_mmIDAIDB__Intercept
Chain
1
2
3
4

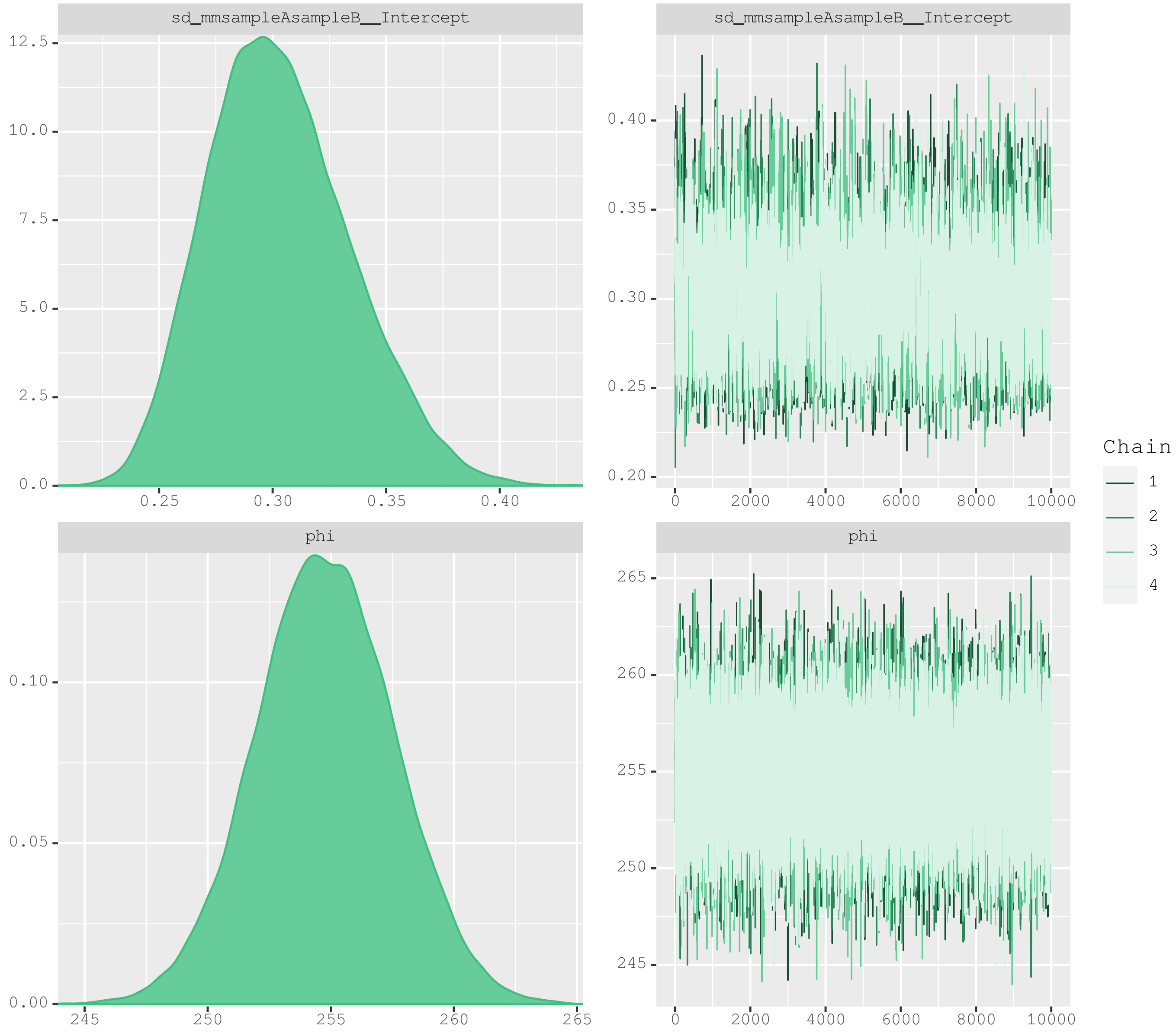

2.3.1. Compare distribution of response variable to distributions of predicted response variable values

```
pp_model_BC <-pp_check(model_WU, ndraws = 50)

pp_model_WU
```

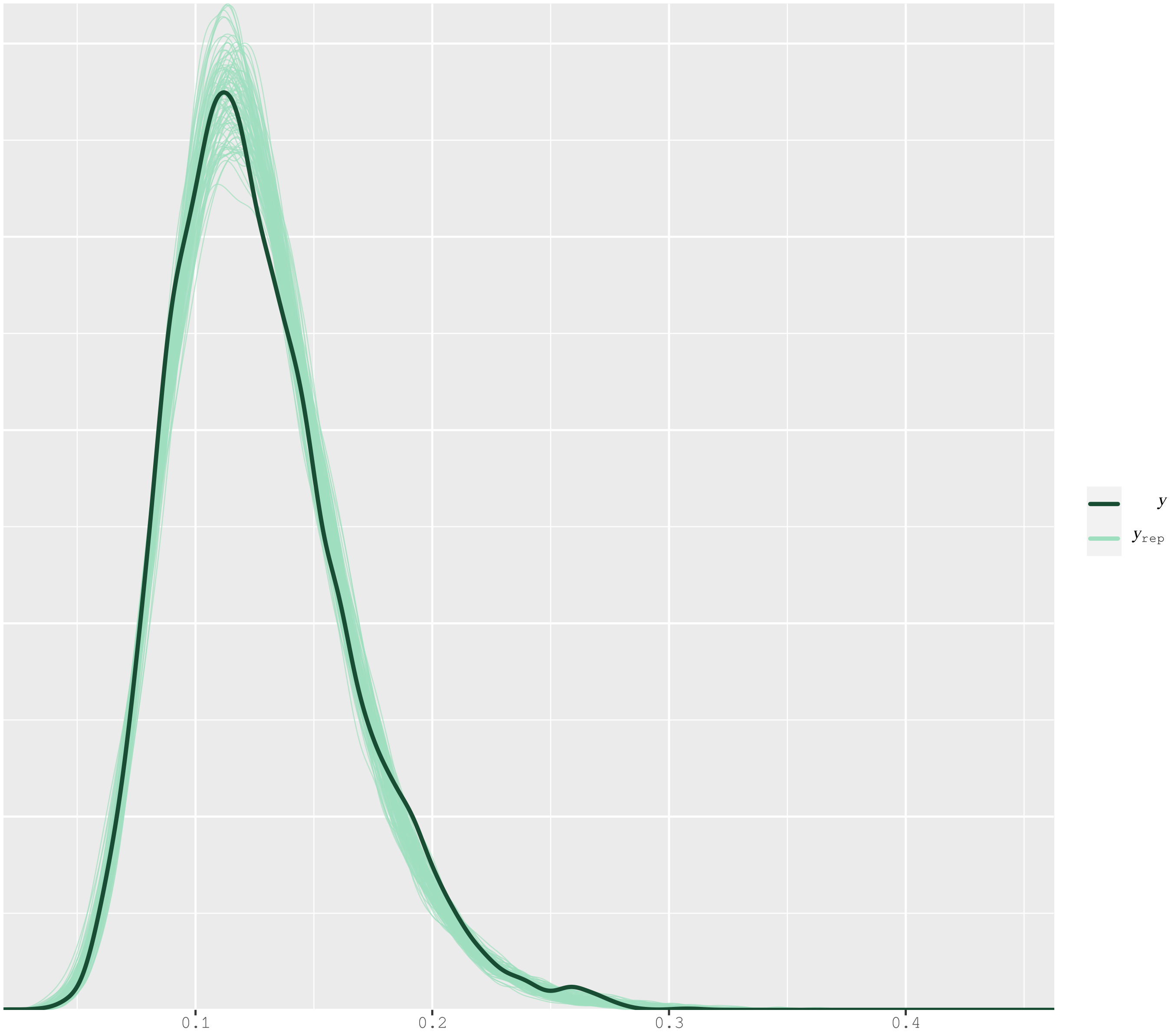

2.4. Model Summary

```
summary(model_WU)


Family: beta
  Links: mu = logit; phi = identity
Formula: WU_distance ~ 1 + age_diference + bci_diference + lbinom_comp + nest_sim + sex_sim + year_sim +
habitat_sim + (1 | mm(sampleA, sampleB)) + (1 | mm(IDA, IDB))
   Data: data.dyad (Number of observations: 16471)
  Draws: 4 chains, each with iter = 20000; warmup = 10000; thin = 1;
         total post-warmup draws = 40000

Group-Level Effects:
~mmIDAIDB (Number of levels: 110)
              Estimate     Est.Error    l-95% CI     u-95% CI    Rhat    Bulk_ESS    Tail_ESS
sd(Intercept)     0.20        0.06         0.05         0.30      1.00      2473         2493

~mmsampleAsampleB (Number of levels: 182)
              Estimate    Est.Error     l-95% CI    u-95% CI    Rhat    Bulk_ESS    Tail_ESS
sd(Intercept)     0.30        0.03         0.25        0.37      1.00      3590        5450

Population-Level Effects:
                Estimate   Est.Error   l-95% CI   u-95% CI   Rhat   Bulk_ESS   Tail_ESS
```

```
Intercept          -1.93      0.04      -2.01      -1.85     1.00     22627       27001
age_diference       0.10      0.01       0.08       0.12     1.00     94893       27585
bci_diference       0.03      0.01       0.00       0.05     1.00     93067       26189
lbinom_compNiNi    -0.06      0.06      -0.17       0.05     1.00     11669       20499
lbinom_compNiI     -0.03      0.03      -0.08       0.03     1.00     11780       21192
nest_sim1          -0.11      0.01      -0.14      -0.09     1.00     88616       27856
sex_sim1           -0.00      0.00      -0.01       0.01     1.00     89743       26096
year_sim1           0.01      0.01      -0.01       0.03     1.00     90232       28801
habitat_sim1       -0.05      0.01      -0.06      -0.03     1.00     92715       27700

Family Specific Parameters:
    Estimate    Est.Error    l-95% CI    u-95% CI    Rhat    Bulk_ESS    Tail_ESS
phi   254.78      2.81         249.27      260.30     1.00     86114        27141

Draws were sampled using sampling(NUTS). For each parameter, Bulk_ESS
and Tail_ESS are effective sample size measures, and Rhat is the potential
scale reduction factor on split chains (at convergence, Rhat = 1).
```

2.5. Plot model posterior and credible intervals

```
plot1 <-mcmc_plot(model_WU, type = "intervals", prob = 0.90, variable = c("b_age_diference","b_bci_diference",
"b_lbinom_compNiNi",
                                                         "b_lbinom_compNiI", "b_nest_sim1", "b_year_sim1",
"b_sex_sim1", "b_habitat_sim1"))

plot1 + theme_default() + geom_vline(xintercept = 0, linetype="dotted", color="black")
```

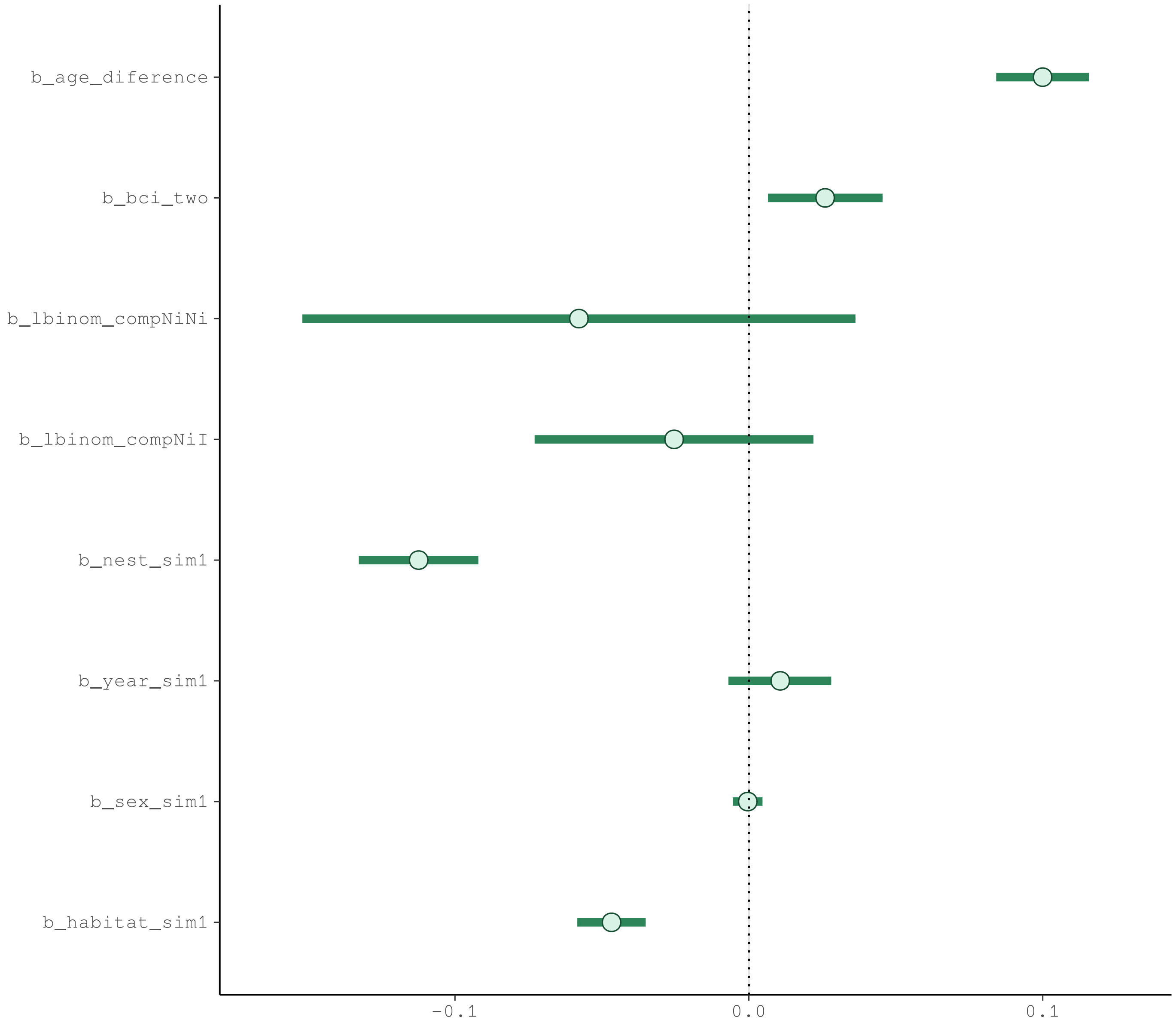

2.6. Plot model effects

Depicts the range of predicted Bray-Curtis dissimilarity values, does not depict confidence intervals.

```
conditional_effects(model_WU)
```

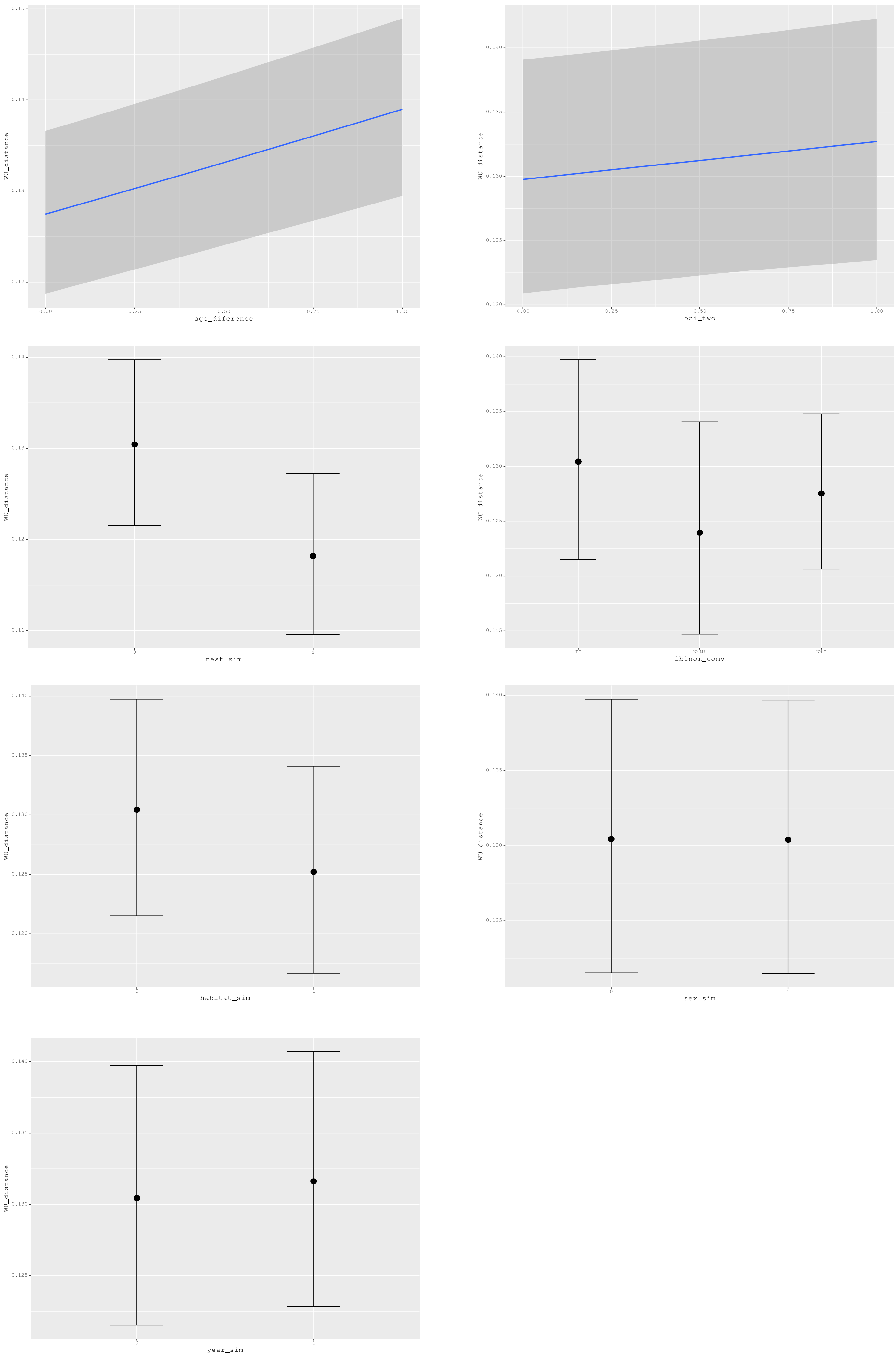
WU_distance
age_diference
0.00
0.25
0.50
0.75
1.00
bci_two
nest_sim
0
1
lbinom_comp
II
NiNi
NiI
habitat_sim
sex_sim
year_sim
