# Supplementary material for: Early-life factors shaping the gut microbiota of Common buzzard nestlings
Source: Anim Microbiome. 2024 May 14;6:27. doi: 10.1186/s42523-024-00313-8 (PMC11092241; doi:10.1186/s42523-024-00313-8)

Diferential Abundant Analysis with ANOCOM-BC2

Pipeline adapted from https://bioconductor.org/packages/release/bioc/vignettes/ANCOMBC/inst/doc/ANCOMBC2.html

Table of Contents

Diferential Abundant Analysis with ANOCOM-BC2
- Table of Contents

A) DAA 16s rRNA
- 1. Read in the data
- 2. Run ANCOM-BC2
  - 2.1 ANCOM-BC2 at ASV level
    - 2.1.1 ANCOM-BC2 primary analysis
    - 2.1.2 Sensitivity scores
    - 2.2.3 Plot ANCOM-BC2 results

B) DAA 28s rRNA
- 1. Read in the data
- 2. Run ANCOM-BC2
  - 2.1 ANCOM-BC2 at ASV level
    - 2.1.1 ANCOM-BC2 primary analysis
    - 2.1.2 Sensitivity scores
    - 2.2.3 Plot ANCOM-BC2 results

A) DAA 16s rRNA

1. Read in the data

```
# Load libraries
library(qiime2R)
library(phyloseq)
library(tidyverse)
library(janitor)
library(microbiome)
library(ANCOMBC)
library(ggrepel)

# Import data from qiime2 and create a phyloseq object

ps <- qza_to_phyloseq(
  features="unrarefied-table.qza",
  tree="rooted-tree.qza",
  taxonomy = "taxonomy.qza",
  metadata = "metadata.tsv")


# Edit metadata file

metadata <- sample_data(ps)
metadata <- clean_names(metadata)
```

```
taxonomy <- as.data.frame(tax_table(ps))
taxonomy$Kingdom <- gsub("d__","",as.character(taxonomy$Kingdom))
tree <- phy_tree(ps)
asv <- otu_table(ps)

metadata$identifier <- as.factor(metadata$identifier)
metadata$ring_number <- as.factor(metadata$ring_number)
metadata$habitat <- as.factor(metadata$habitat)
metadata$nest <- as.factor(metadata$nest)
metadata$rank <- as.factor(metadata$rank)
metadata$year <- as.factor(metadata$year)
metadata$lbinom <- as.factor(metadata$lbinom)
metadata$sex <- as.factor(metadata$sex)
metadata$age_days <- as.numeric(metadata$age_days)
metadata$std_age <- as.numeric(metadata$std_age)
metadata$bci_two <- as.numeric(metadata$bci_two)
metadata$std_bci_two <- as.numeric(metadata$std_bci_two)
metadata$faith_pd <- as.numeric(metadata$faith_pd)
metadata$shannon_entropy <- as.numeric(metadata$shannon_entropy)

# New phyloseq object
ps <- phyloseq(asv, taxonomy, metadata, tree)

# Save edited ps object as rds
saveRDS(ps,"phyloseq.rds")
```

2. Run ANCOM-BC2

2.1 ANCOM-BC2 at ASV level

```
# Diferential abundace analysis on final model. Genus level
# age + bci + rank + sex + year + habitat + lbinom


set.seed(123)
output_final = ancombc2(data = ps, assay_name = "counts", tax_level = NULL,
                        fix_formula = " std_age + std_bci_two + rank + sex  + year + habitat+lbinom", # input
model
                        rand_formula = "(1|nest/ring_number)", # input random effects
                        p_adj_method = "holm", pseudo = 0, pseudo_sens = TRUE, # multicomparison p adjustment
                        prv_cut = 0.10, lib_cut = 0, s0_perc = 0.05,
                        #group = NULL, struc_zero = TRUE, neg_lb = TRUE,
                        alpha = 0.05, n_cl = 2, verbose = TRUE, # p values cut off
                        #global = TRUE, pairwise = TRUE, dunnet = TRUE, trend = TRUE,
                        iter_control = list(tol = 1e-2, max_iter = 50, verbose = TRUE), # number of iterations
                        em_control = list(tol = 1e-5, max_iter = 100),
                        lme_control = lme4::lmerControl(optimizer ="Nelder_Mead"),
                        mdfdr_control = list(fwer_ctrl_method = "holm", B = 100),
                        trend_control = list(contrast = list(matrix(c(1, 0, -1, 1),
                        nrow = 2, byrow = TRUE), matrix(c(-1, 0, 1, -1), nrow = 2,
                        byrow = TRUE)), node = list(2, 2), solver = "ECOS", B = 100))
```

2.1.1 ANCOM-BC2 primary analysis

```
res_prim_asv = output_final_asv$res
```

Only found asvs that co-vary with age

2.1.2 Sensitivity scores

ANCOM-BC2 uses a sensitivity analysis to assess the impact of different pseudo-counts on zero counts for each taxon. The sensitivity score is determined by performing linear regression models on the bias-corrected log abundance table using various pseudo-counts and calculating the proportion of times the p-value exceeds the significance level (alpha). This helps identify taxa that are not sensitive to the pseudo-count addition, ensuring robustness in the analysis.

```
tab_sens_asv = output_asv$pseudo_sens_tab
```

2.2.3 Plot ANCOM-BC2 results

```
# Volcano plots
volc_asv <- ggplot(data=res_prim_asv, aes(x=lfc_std_age, y=-log10(p_std_age), col=diff_std_age)) + geom_point()
+
  geom_text_repel(aes(label = ifelse(diff_std_age, taxon, ""))) +
  theme_bw()
volc_asv
```

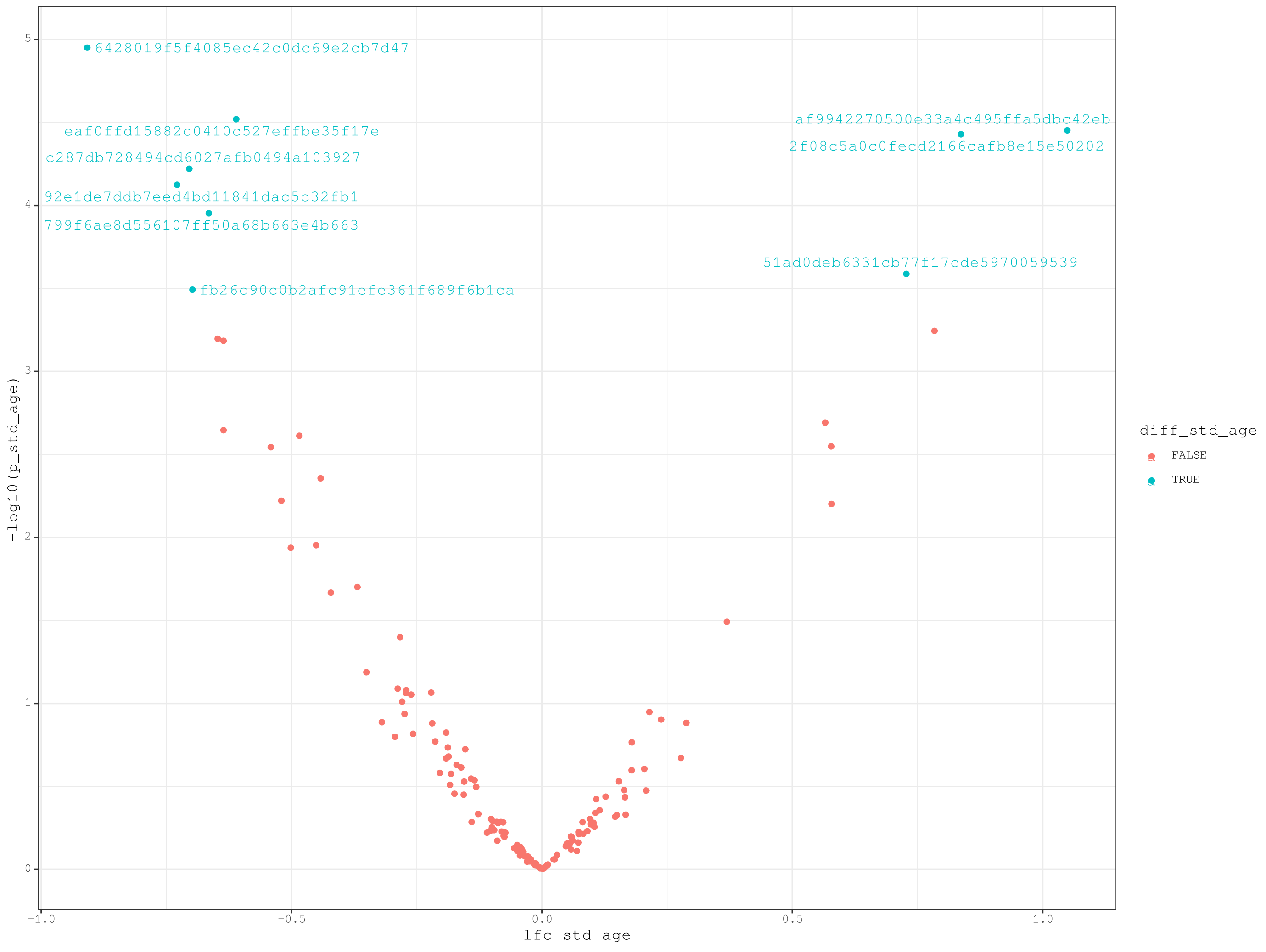

```r
# Plot log fold changes with unit of age

## Subset a dataset only for age results

df_age_asv = res_prim_asv %>% dplyr::select(taxon, ends_with("age")) # create a dataframe with values only for
age

df_fig_age_asv = df_age_asv %>%
  filter(diff_std_age == TRUE) %>%
  arrange(desc(lfc_std_age)) %>%
  mutate(direct = ifelse(lfc_std_age > 0, "Positive LFC", "Negative LFC")) # prepare the dataframe for plotting

df_fig_age_asv$taxon = factor(df_fig_age_asv$taxon, levels = df_fig_age_asv$taxon)
df_fig_age_asv$direct = factor(df_fig_age_asv$direct,
                                 levels = c("Positive LFC", "Negative LFC"))

## Make the plot

fig_age_asv = df_fig_age_asv %>%
  ggplot(aes(x = taxon, y = lfc_std_age, fill = direct)) +
  geom_bar(stat = "identity", width = 0.7, color = "black",
           position = position_dodge(width = 0.4)) +
  geom_errorbar(aes(ymin = lfc_std_age - se_std_age, ymax = lfc_std_age + se_std_age),
                width = 0.2, position = position_dodge(0.05), color = "black") +
  labs(x = NULL, y = "Log fold change", title = "Log fold changes as one unit increase of age") +
  scale_fill_discrete(name = NULL) +
  scale_color_discrete(name = NULL) +
  theme_bw() +
  theme(plot.title = element_text(hjust = 0.5),
        panel.grid.minor.y = element_blank(),
        axis.text.x = element_text(angle = 60, hjust = 1))

fig_age_asv
```

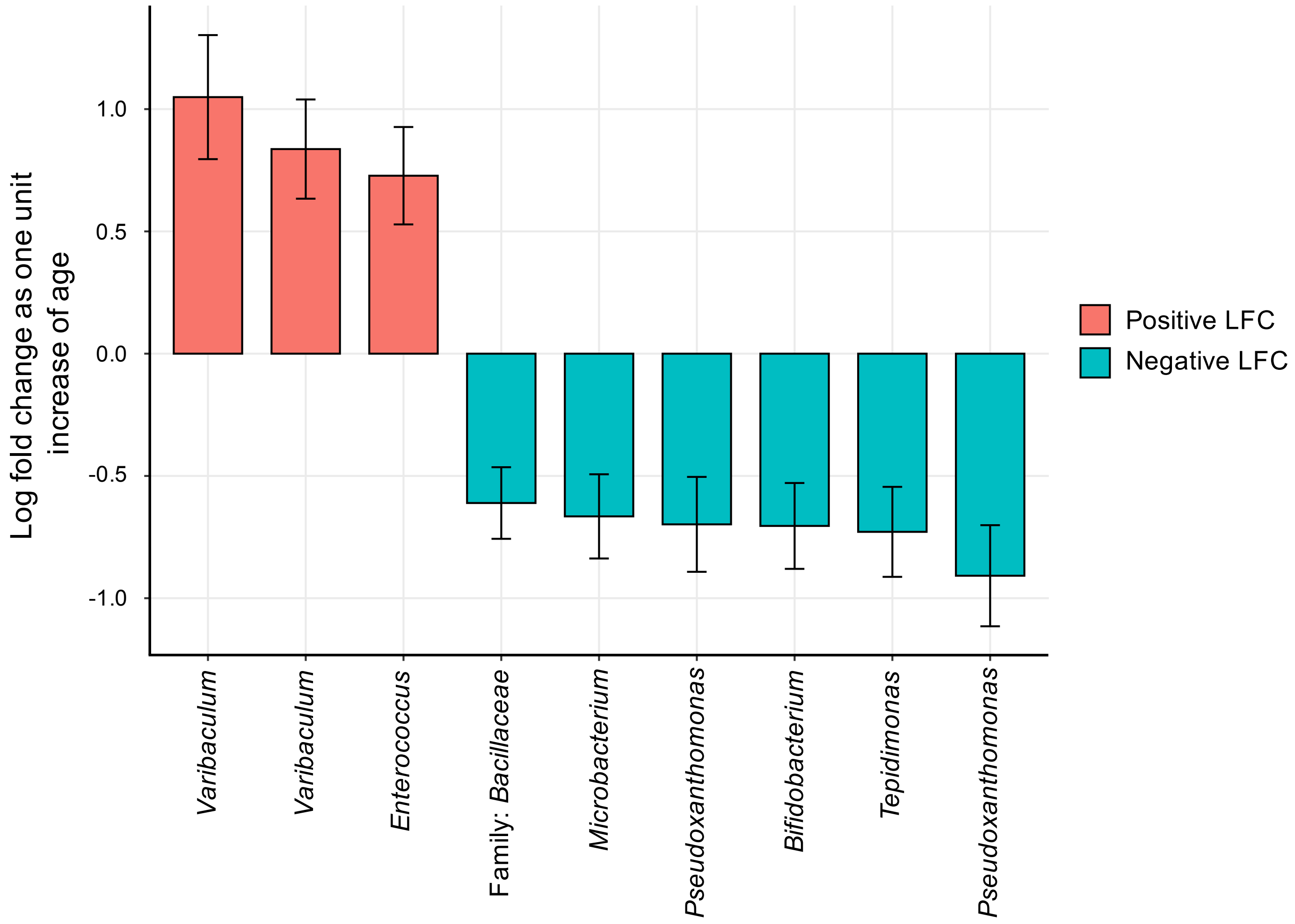


```r
# Pseudo-count sensitivity analysis for age

sens_asv_age = tab_sens_asv %>%
  transmute(taxon, sens_asv_age = std_age) %>%
  left_join(df_age_asv, by = "taxon")

sens_asv_age$diff_std_age = recode(sens_asv_age$diff_std_age * 1,
                               `1` = "Significant",
                               `0` = "Non-significant")

fig_sens_asv_age = sens_asv_age %>%
  ggplot(aes(x = taxon, y = sens_asv_age, color = diff_std_age)) +
  geom_point() +
  scale_color_brewer(palette = "Dark2", name = NULL) +
  labs(x = "ASVs", y = "Sensitivity Score") +
  theme_bw() +
  #theme(axis.text.x = element_text(angle = 60, vjust = 0.5))
theme(axis.text.x = element_blank(), panel.grid.major.x = element_blank())  # Hide the axis text on the x-axis

fig_sens_asv_age
```

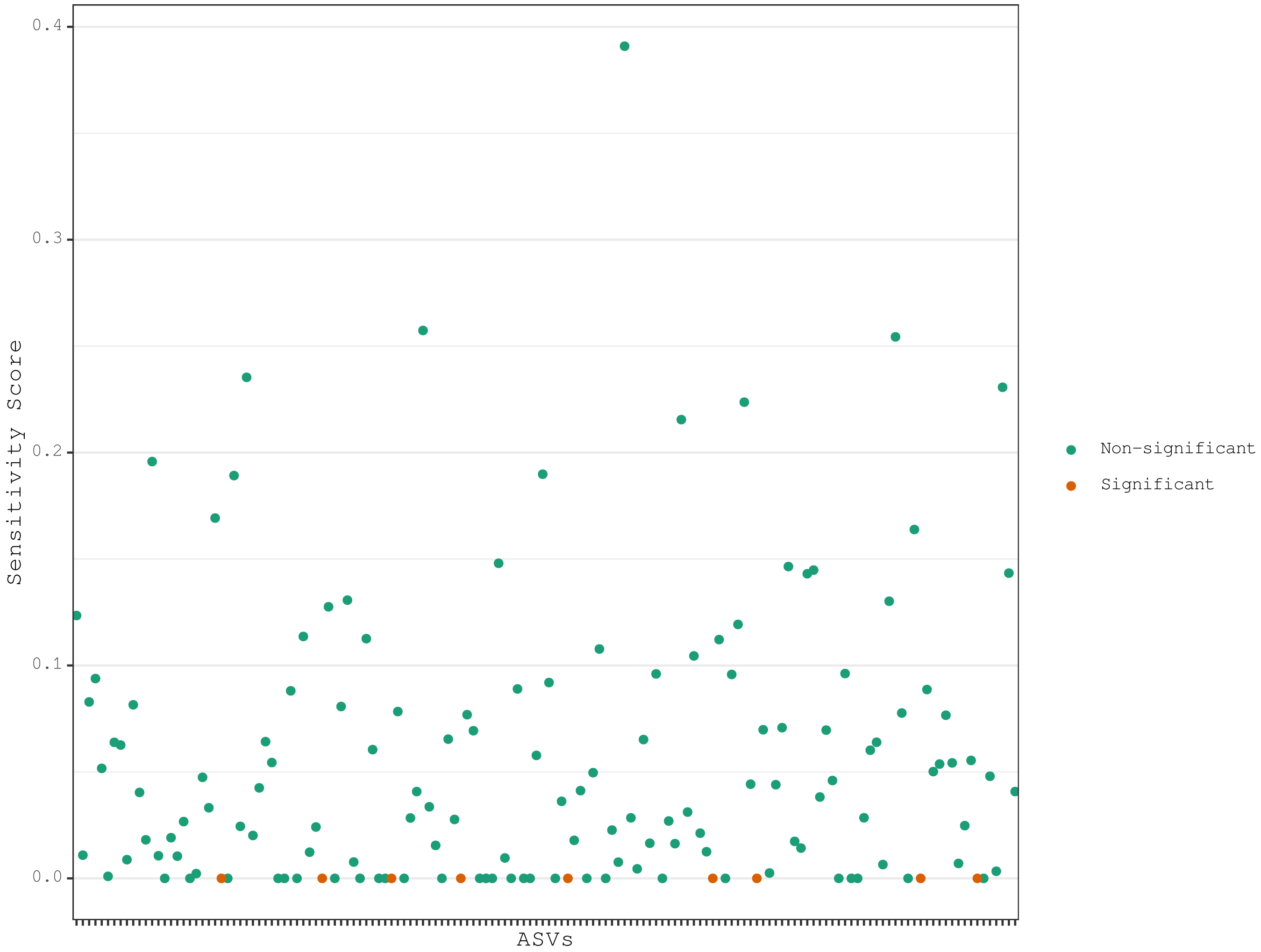

For the co-variate of age, no outlying sensitivity scores are observed. All significant taxa have low sensitivity scores.

B) DAA 28s rRNA

1. Read in the data

```
# Import data from qiime2 and create a phyloseq object

ps <- qza_to_phyloseq(
  features="unrarefied-table.qza",
  tree=".rooted-tree.qza",
  taxonomy = "taxonomy.qza",
  metadata = "metadata.tsv")


# Edit metadata file

metadata <- sample_data(ps)
metadata <- clean_names(metadata)
taxonomy <- as.data.frame(tax_table(ps))
taxonomy$Kingdom <- gsub("d__","",as.character(taxonomy$Kingdom))
tree <- phy_tree(ps)
asv <- otu_table(ps)
```

```
metadata$identifier <- as.factor(metadata$identifier)
metadata$ring_number <- as.factor(metadata$ring_number)
metadata$habitat <- as.factor(metadata$habitat)
metadata$nest <- as.factor(metadata$nest)
metadata$rank <- as.factor(metadata$rank)
metadata$year <- as.factor(metadata$year)
metadata$lbinom <- as.factor(metadata$lbinom)
metadata$sex <- as.factor(metadata$sex)
metadata$age_days <- as.numeric(metadata$age_days)
metadata$std_age <- as.numeric(metadata$std_age)
metadata$bci_two <- as.numeric(metadata$bci_two)
metadata$std_bci_two <- as.numeric(metadata$std_bci_two)
metadata$faith_pd <- as.numeric(metadata$faith_pd)
metadata$shannon_entropy <- as.numeric(metadata$shannon_entropy)

# New phyloseq object
ps <- phyloseq(asv, taxonomy, metadata, tree)

# Save edited ps object as rds
saveRDS(ps,"phyloseq.rds")
```

2. Run ANCOM-BC2

2.1 ANCOM-BC2 at ASV level

```
# Diferential abundace analysis on final model. Genus level
# age + bci + rank + sex + year + habitat + lbinom


set.seed(123)
output_final = ancombc2(data = ps, assay_name = "counts", tax_level = NULL,
                        fix_formula = " std_age + std_bci_two + rank + sex  + year + habitat+lbinom", # input
model
                        rand_formula = "(1|nest/ring_number)", # input random effects
                        p_adj_method = "holm", pseudo = 0, pseudo_sens = TRUE, # multicomparison p adjustment
                        prv_cut = 0.10, lib_cut = 0, s0_perc = 0.05,
                        #group = NULL, struc_zero = TRUE, neg_lb = TRUE,
                        alpha = 0.05, n_cl = 2, verbose = TRUE, # p values cut off
                        #global = TRUE, pairwise = TRUE, dunnet = TRUE, trend = TRUE,
                        iter_control = list(tol = 1e-2, max_iter = 50, verbose = TRUE), # number of iterations
                        em_control = list(tol = 1e-5, max_iter = 100),
                        lme_control = lme4::lmerControl(optimizer ="Nelder_Mead"),
                        mdfdr_control = list(fwer_ctrl_method = "holm", B = 100),
                        trend_control = list(contrast = list(matrix(c(1, 0, -1, 1),
                        nrow = 2, byrow = TRUE), matrix(c(-1, 0, 1, -1), nrow = 2,
                        byrow = TRUE)), node = list(2, 2), solver = "ECOS", B = 100))
```

2.1.1 ANCOM-BC2 primary analysis

```
res_prim_asv = output_final_asv$res
```

Only found asvs that co-vary with age

2.1.2 Sensitivity scores

```
tab_sens_asv = output_asv$pseudo_sens_tab
```

2.2.3 Plot ANCOM-BC2 results

```
# Volcano plots
volc_asv <- ggplot(data=res_prim_asv, aes(x=lfc_habitatsouth, y=-log10(p_habitatsouth), col=diff_habitatsouth))
+ geom_point() +
  geom_text_repel(aes(label = ifelse(diff_habitatsouth, taxon, ""))) +
  theme_bw()
volc_asv
```

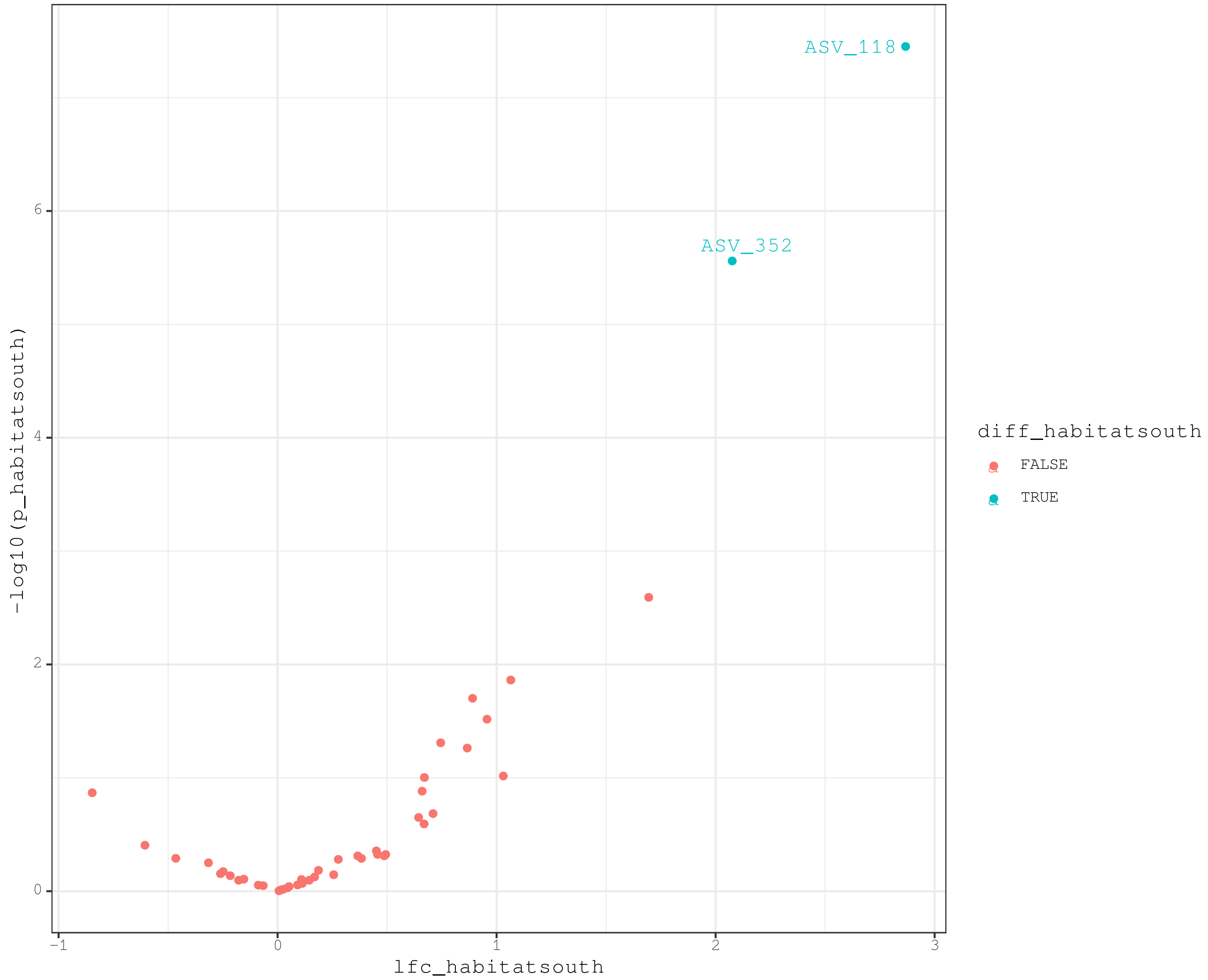


```
## Pseudo count sensitivity analysis for habitat_south

df_habitat_asv = res_prim_asv %>% dplyr::select(taxon, ends_with("south")) # create a dataframe with values
only for age

sens_asv_habitat = tab_sens_asv %>%
```

```
  transmute(taxon, sens_asv_habitat = habitatsouth) %>%
  left_join(df_habitat_asv, by = "taxon")

sens_asv_habitat$diff_habitatsouth = recode(sens_asv_habitat$diff_habitatsouth * 1,
                                   `1` = "Significant",
                                   `0` = "Non-significant")

fig_sens_asv_habitat = sens_asv_habitat %>%
  ggplot(aes(x = taxon, y = sens_asv_habitat, color = diff_habitatsouth)) +
  geom_point() +
  scale_color_brewer(palette = "Dark2", name = NULL) +
  labs(x = "ASVs", y = "Sensitivity Score") +
  theme_bw() +
  #theme(axis.text.x = element_text(angle = 60, vjust = 0.5))
  theme(axis.text.x = element_blank(), panel.grid.major.x = element_blank())  # Hide the axis text on the x-
axis

fig_sens_asv_habitat

#For the covariate of habitat no outlying sensitivity scores are observed. All significant taxa have low
sensitivity scores.
```

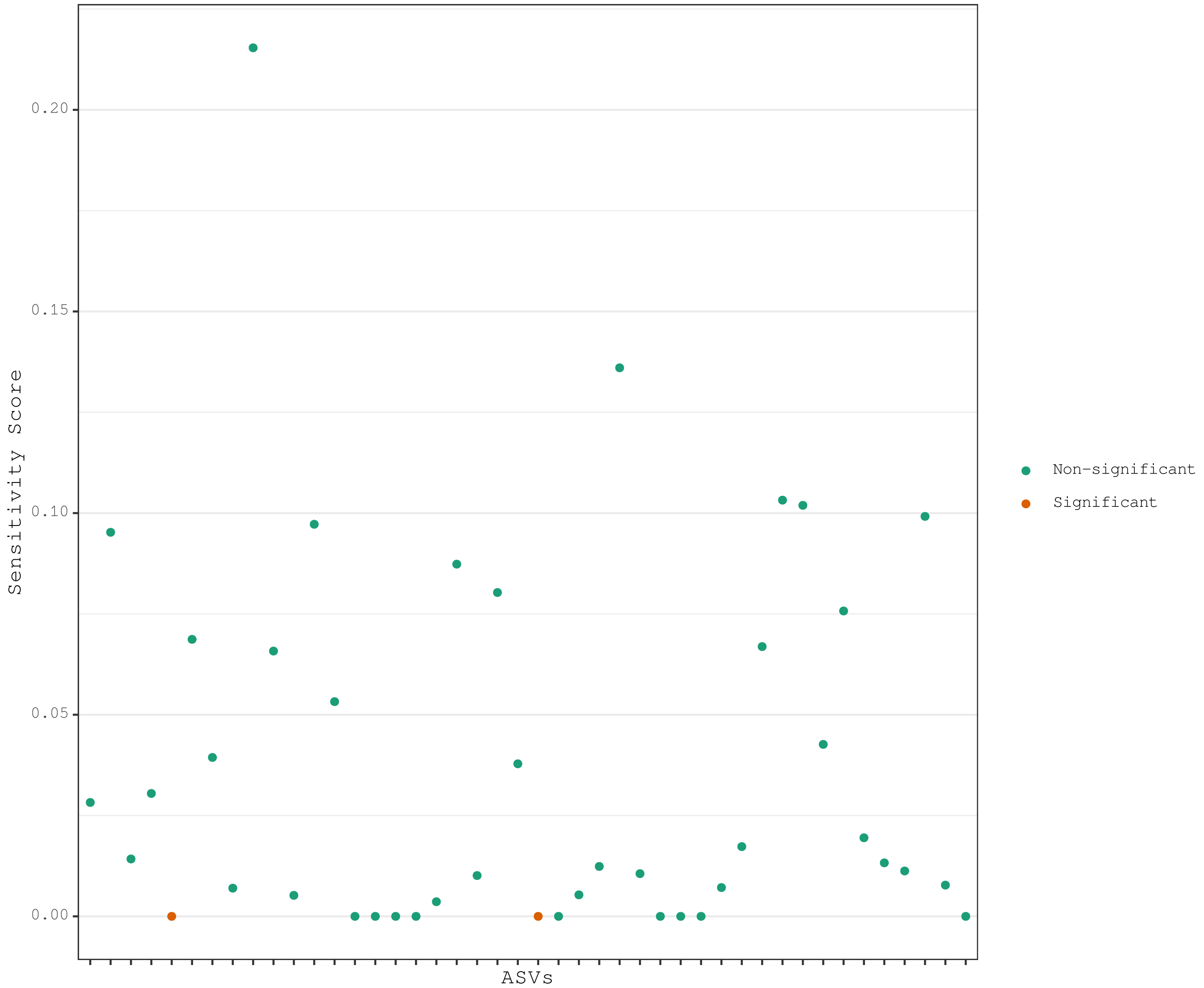

For the co-variate of habitat (south), no outlying sensitivity scores are observed. All significant taxa have low sensitivity scores.

```r
# Plot log fold changes. Pairwise comparisson with North

res_pair = output_final_asv2$res_pair

df_habitat = res_prim %>%
  dplyr::select(taxon, contains("habitat"))


df_fig_habitat = df_habitat %>%
  dplyr::filter((diff_habitatsouth == 1 | diff_habitatteuto == 1)) %>%
  dplyr::mutate(lfc_habitatteuto = ifelse(diff_habitatteuto == 1,
                                          lfc_habitatteuto, 0),
                lfc_habitatsouth = ifelse(diff_habitatsouth == 1,
                                          lfc_habitatsouth, 0)) %>%
  dplyr::transmute(taxon,
                   `Teuto vs. North` = round(lfc_habitatteuto, 2),
                   `South vs. North` = round(lfc_habitatsouth, 2)) %>%
  tidyr::pivot_longer(cols = `Teuto vs. North`:`South vs. North`,
                      names_to = "group", values_to = "value") %>%
  dplyr::arrange(taxon)

lo = floor(min(df_fig_habitat$value))
up = ceiling(max(df_fig_habitat$value))
mid = (lo + up)/2
fig_habitat = df_fig_habitat %>%
  ggplot(aes(x = group, y = taxon, fill = value)) +
  geom_tile(color = "black") +
  scale_fill_gradient2(low = "blue", high = "red", mid = "white",
                       na.value = "white", midpoint = mid, limit = c(lo, up),
                       name = NULL) +
  geom_text(aes(group, taxon, label = value), color = "black", size = 4) +
  labs(x = NULL, y = NULL, title = "Log fold changes as compared to North") +
  theme_minimal() +
  theme(plot.title = element_text(hjust = 0.5))

fig_habitat
```

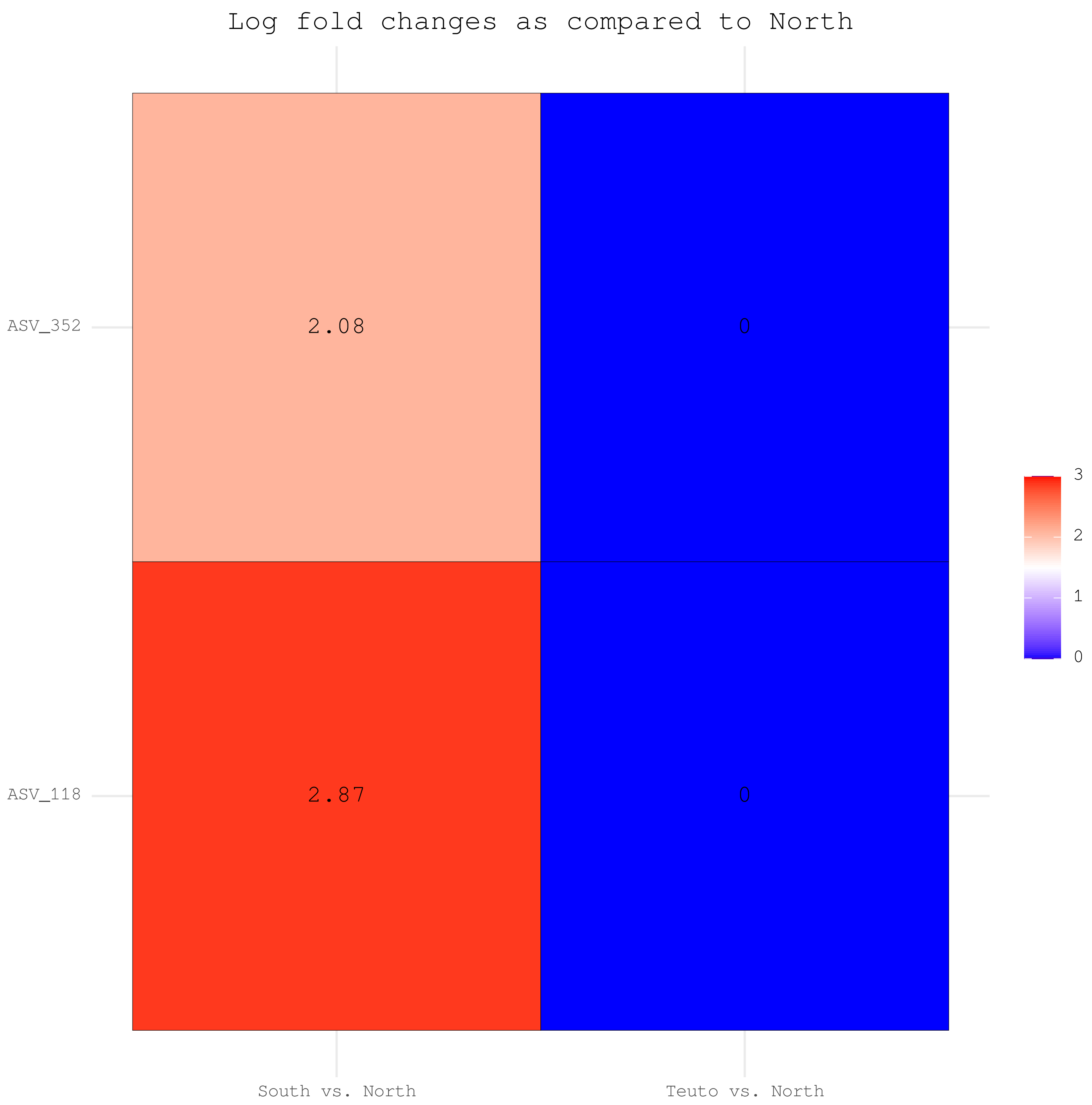


```r
# Plot log fold changes. Multiple pairwise comparisons

res_pair = output_final_asv1$res_pair

df_fig_pair = res_pair %>%
  dplyr::filter((diff_habitatsouth == 1 |diff_habitatteuto == 1 |diff_habitatteuto_habitatsouth == 1)) %>%
  dplyr::mutate(lfc_habitatsouth = ifelse(diff_habitatsouth == 1,
                                   lfc_habitatsouth, 0),
                lfc_habitatteuto = ifelse(diff_habitatteuto == 1,
                                         lfc_habitatteuto, 0),
                lfc_habitatteuto_habitatsouth = ifelse(diff_habitatteuto_habitatsouth == 1,
                                                      lfc_habitatteuto_habitatsouth, 0)) %>%
  dplyr::transmute(taxon,
                   `South vs. North` = round(lfc_habitatsouth, 2),
                   `Teuto vs. North` = round(lfc_habitatteuto, 2),
                   `South vs. Teuto` = round(lfc_habitatteuto_habitatsouth, 2)
  ) %>%
  tidyr::pivot_longer(cols = `South vs. North`:`South vs. Teuto`,
                      names_to = "group", values_to = "value") %>%
```

```
  dplyr::arrange(taxon)
df_fig_pair$group = factor(df_fig_pair$group,
                           levels = c("South vs. North",
                                      "Teuto vs. North",
                                      "South vs. Teuto"))

lo = floor(min(df_fig_pair$value))
up = ceiling(max(df_fig_pair$value))
mid = (lo + up)/2
fig_pair = df_fig_pair %>%
  ggplot(aes(x = group, y = taxon, fill = value)) +
  geom_tile(color = "black") +
  scale_fill_gradient2(low = "blue", high = "red", mid = "white",
                       na.value = "white", midpoint = mid, limit = c(lo, up),
                       name = NULL) +
  geom_text(aes(group, taxon, label = value), color = "black", size = 4) +
  labs(x = NULL, y = NULL, title = "Log fold change of pairwise comparisons") +
  theme_minimal() +
  theme(plot.title = element_text(hjust = 0.5))
fig_pair
```

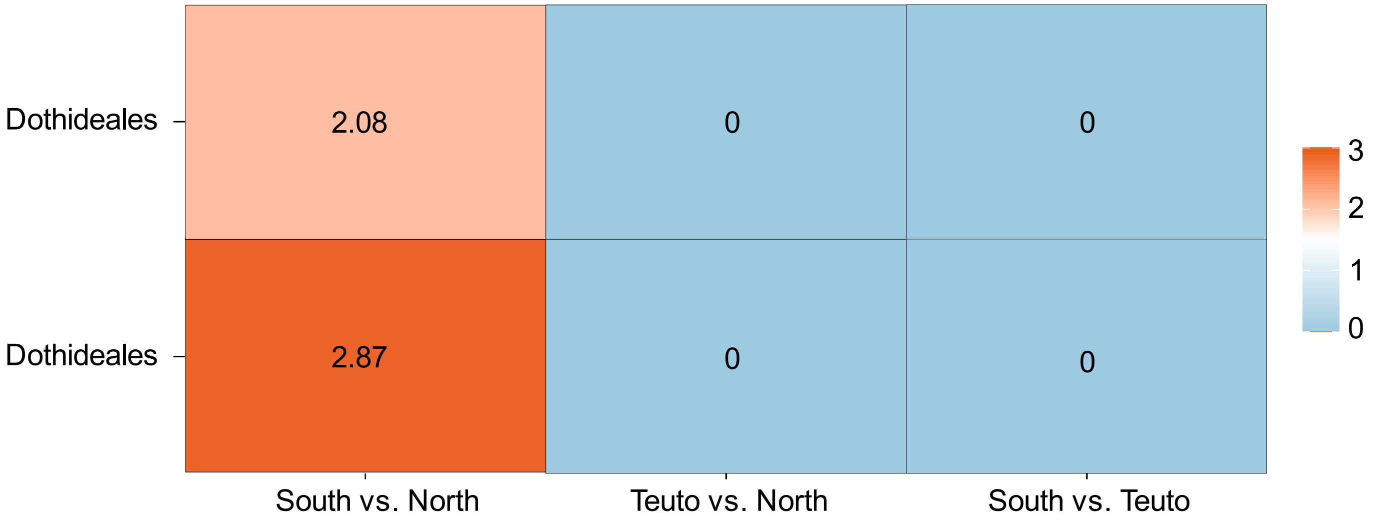

Supplement: Supplementary file 8 — Supplementary file1 (PDF 216 kb) [file 42523_2024_313_MOESM8_ESM.pdf]
